# Supplementary material for: Initial Upper Palaeolithic humans in Europe had recent Neanderthal ancestry
Source: Nature. 2021 Apr 7;592(7853):253–7. doi: 10.1038/s41586-021-03335-3 (PMC8026394; doi:10.1038/s41586-021-03335-3)
Supplement: Supplementary file 1 — This file contains Supplementary Information Sections 1-10 with Supplementary Tables S1.1-S1.2, S2.1-S2.10, S4.1, S5.1-S5.15, S7.1, S8.1, S9.1, Supplementary Figures S2.1-S2.2, S5.1-S5.14, S6.1-S6.8, S7.1-S7.17, S8.1-S8.6, S9.1-S9.4 and Supplementary References for each section. See contents page of the Supplementary Information file for more details. [file 41586_2021_3335_MOESM1_ESM.pdf]

nature portfolio

https://doi.org/10.1038/s41586-021-03335-3

## Supplementary information

# Initial Upper Palaeolithic humans in Europe had recent Neanderthal ancestry

In the format provided by the authors and unedited

# Supplementary Information

## Contents:

**Supplementary Information 1** ........ **2**
Bacho Kiro Cave and human remains ........ 2
**Supplementary Information 2** ........ **11**
Ancient DNA processing, quality controls and contamination estimates ........ 11
**Supplementary Information 3** ........ **32**
Datasets for downstream analyses ........ 32
**Supplementary Information 4** ........ **36**
Relatedness, sex determination and Y chromosome analyses ........ 36
**Supplementary Information 5** ........ **41**
Population relationships ........ 41
**Supplementary Information 6** ........ **77**
Admixture Graph Modelling ........ 77
**Supplementary Information 7** ........ **86**
Neandertal ancestry in Bacho Kiro individuals ........ 86
**Supplementary Information 8** ........ **108**
Neandertal introgressed segments in Bacho Kiro individuals and dating of Neandertal admixture ........ 108
**Supplementary information 9** ........ **123**
Sharing of Neandertal introgressed segments between Bacho Kiro and present-day individuals ........ 123
**Supplementary Information 10** ........ **130**
Neandertal deserts ........ 130

# Supplementary Information 1

## Bacho Kiro Cave and human remains

Bacho Kiro Cave is located on the northern slope of the Balkan Mountains and 5 km away from the city of Dryanovo in Bulgaria. First excavated in 1938 by D. Garrod[1] and later in 1970s by a team led by J. Kozłowski[2], Bacho Kiro Cave was recently re-opened and excavated by a research team led by the National Archaeological Institute with Museum in Sofia and the Department of Human Evolution at the Max Planck Institute for Evolutionary Anthropology in Leipzig[3,4]. New excavations focused on two sectors adjacent to the area excavated in 1970s, the Main Sector and the Niche 1 as detailed previously[3,4]. The archaeological sequence of the cave spans the late Middle Palaeolithic (MP) and the early Upper Palaeolithic (UP), with an archaeologically rich assemblage in Layer I[3,4] recognized as a variant of the Initial Upper Palaeolithic (IUP)[5].

Five human specimens from the new excavations were found in a direct association with an IUP assemblage in Bacho Kiro Cave (Fig. 1)[3]. A human lower molar (*F6-620*) was found in the upper part of the Layer J in the Main Sector of the cave and four bone fragments (*AA7-738, BB7-240*, *CC7-22*89 and *CC7-335*) were found in the Layer I in the Niche 1 sector. These specimens were directly radiocarbon dated to between 45,930 and 42,580 cal. BP[3,4] (re-calibrated with OxCal 4.4.2[6] and IntCal20[7], Tab. S1.1). Their mitochondrial genomes (mtDNA) are attributed to modern humans, suggesting that they are the oldest modern humans in Europe recovered to date[3].

Specimens from Kent's Cavern in United Kingdom[8] and Grotta del Cavallo in Italy[9] have been claimed to represent the earliest evidence of modern humans in western Europe (Fig. 1). However their dates are based on the archaeological context and the exact provenance of these specimens has been extensively debated[10,11]. Modern human remains older than 40,000 cal. BP in Europe are extremely sparse (Fig. 1)[12-14]. Moreover, genome-wide data has been retrieved for only three modern humans from Eurasia that are older than this[15-17] and each of these individuals was found outside of any archaeological context.

The archaeological sites with IUP assemblages span an area from North Africa, southwest Asia, eastern and central Europe to north China[18,19] (Fig. 1, Tab. S1.2), and have been suggested to be associated with the first wave of modern human dispersals out-of-Africa[19]. However, human remains found in the IUP context to date are relatively few[12,20-22], highlighting the relevance of Bacho Kiro Cave IUP human finds[3,4].

Two additional bone fragments were found in Layer B in the Main Sector of Bacho Kiro Cave (*F6-597*) and in the collections of the 1970s excavations in a position corresponding to the interface of Layers B/C (*BK1653*). These two specimens were directly dated to 36,320-35,600 cal. BP and 35,290-34,610 cal. BP[3,4], respectively (re-calibrated with OxCal 4.4.2[6] and IntCal20[7], Tab. S1.1). Although the lithic assemblages from these layers are sparse, they are likely to be Aurignacian[3,4]. Thus, there is ~10,000 years between the two groups of human remains found in the Bacho Kiro Cave. Even though there are more individuals that are older than 30,000 cal. BP and from whom genome-wide data has been retrieved (Fig. 1, Tab. S1.1)[23-26], this genetic record is still quite fragmentary compared to more recent timeperiods (https://reich.hms.harvard.edu/downloadable-genotypes-present-day-and-ancient-dna-data-compiled-published-papers/).

**References SI1:**


1 Garrod, D. A., Howe, B. & Gaul, J. Excavations in the cave of Bacho Kiro, north-east Bulgaria. *Bulletin of the American School of Prehistoric research* **15**, 46-76 (1939).

2 Kozłowski, J. K. & Ginter, B. Excavation in the Bacho Kiro Cave (Bulgaria): Final Report. *Pańswowe Wydawnictwo Naukowe* (1982).

3 Hublin, J.-J. et al. Initial Upper Palaeolithic Homo sapiens from Bacho Kiro Cave, Bulgaria. *Nature* **581**, 299-302, doi:10.1038/s41586-020-2259-z (2020).

4 Fewlass, H. et al. A 14 C chronology for the Middle to Upper Palaeolithic transition at Bacho Kiro Cave, Bulgaria. *Nat. Ecol. Evol.* **4**, 794-801, doi: 10.1038/s41559-020-1136-3 (2020).

5 Tsanova, T. & Borders, J. The Humanized Mineral World: Towards Social and Symbolic Evaluation of Prehistoric Technologies in South Eastern Europe. *Proceedings of the ESF workshop* Sofia 3-6 September 2003, 1-129 (2003).

6 Ramsey, C. B. Bayesian analysis of radiocarbon dates. *Radiocarbon* **51**, 337-360 (2009).

7 Reimer, P. J. *et al.* The IntCal20 Northern Hemisphere radiocarbon age calibration curve (0–55 cal kBP). *Radiocarbon* **62**, 725-757 (2020).

8 Higham, T. *et al.* The earliest evidence for anatomically modern humans in northwestern Europe. *Nature* **479**, 521-524, doi: 10.1038/nature10484 (2011).

9 Benazzi, S. *et al.* Early dispersal of modern humans in Europe and implications for Neanderthal behaviour. *Nature* **479**, 525-528, doi:10.1038/nature10617 (2011).

10 White, M. & Pettitt, P. Ancient digs and modern myths: The age and context of the Kent's Cavern 4 maxilla and the earliest Homo sapiens specimens in Europe. *European Journal of Archaeology* **15**, 392-420 (2012).

11 Zilhão, J., Banks, W. E., d'Errico, F. & Gioia, P. Analysis of site formation and assemblage integrity does not support attribution of the Uluzzian to modern humans at Grotta del Cavallo. *PLoS One* **10**, e0131181 (2015).

12 Hublin, J.-J. The modern human colonization of western Eurasia: when and where? *Quat. Sci. Rev.* **118**, 194-210, doi:10.1016/j.quascirev.2014.08.011 (2015).

13 Benazzi, S. *et al.* The makers of the Protoaurignacian and implications for Neandertal extinction. *Science* **348**, 793-796, doi:10.1126/science.aaa2773 (2015).

14 Trinkaus, E. *et al.* An early modern human from the Peştera cu Oase, Romania. *Proc. Natl. Acad. Sci. U.S.A.* **100**, 11231-11236, doi: 10.1073/pnas.2035108100 (2003).

15 Fu, Q. *et al.* Genome sequence of a 45,000-year-old modern human from western Siberia. *Nature* **514**, 445-449, doi:10.1038/nature13810 (2014).

16 Fu, Q. *et al.* An early modern human from Romania with a recent Neanderthal ancestor. *Nature* **524**, 216-219, doi:10.1038/nature14558 (2015).

17 Yang, M. A. *et al.* 40,000-Year-Old Individual from Asia Provides Insight into Early Population Structure in Eurasia. *Curr. Biol.* **27**, 3202-3208.e9, doi: 10.1016/j.cub.2017.09.030 (2017).

18 Kuhn, S. L. & Zwyns, N. Rethinking the initial Upper Paleolithic. *Quat. Int.* **347**, 29-38, doi: 10.1016/j.quaint.2014.05.040 (2014).

19 Müller, U. C. *et al.* The role of climate in the spread of modern humans into Europe. *Quat. Sci. Rev.* **30**, 273-279 (2011).

20 Kuhn, S. L. *et al.* The early upper paleolithic occupations at Üçağızlı cave (Hatay, Turkey). *Journal of human evolution* **56**, 87-113 (2009).

21 Copeland, L. & Yazbeck, C. Inventory of stone age sites in Lebanon. Part III, additions and revisions, 1967-2001. *Mélanges de l'Université Saint-Joseph* **55**, 119-325 (1997).

22 Yazbeck, C. Le Paléolithique du Liban: bilan critique. *Paléorient*, 111-126 (2004).

23 Seguin-Orlando, A. *et al.* Genomic structure in Europeans dating back at least 36,200 years. *Science* **346**, 1113-1118, doi:10.1126/science.aaa0114 (2014).

24 Fu, Q. *et al.* The genetic history of Ice Age Europe. *Nature* **534**, 200-205, doi:10.1038/nature17993 (2016).

25 Sikora, M. *et al.* Ancient genomes show social and reproductive behavior of early Upper Paleolithic foragers. *Science* **358**, 659-662, doi: 10.1126/science.aao1807 (2017).

26 Sikora, M. *et al.* The population history of northeastern Siberia since the Pleistocene. *Nature* **570**, 182-188, doi: 10.1038/s41586-019-1279-z (2019).

27 Fu, Q. *et al.* DNA analysis of an early modern human from Tianyuan Cave, China. *Proc. Natl. Acad. Sci. U.S.A.* **110**, 2223-2227, doi:10.1073/pnas.1221359110 (2013).

28 Marom, A., McCullagh, J. S., Higham, T. F., Sinitsyn, A. A. & Hedges, R. E. Single amino acid radiocarbon dating of Upper Paleolithic modern humans. *Proc. Natl. Acad. Sci. U.S.A.* **109**, 6878-6881 (2012).

29 Posth, C. *et al.* Pleistocene Mitochondrial Genomes Suggest a Single Major Dispersal of Non-Africans and a Late Glacial Population Turnover in Europe. *Curr. Biol.* **26**, 827-833, doi:10.1016/j.cub.2016.01.037 (2016).

30 Soficaru, A., Doboş, A. & Trinkaus, E. Early modern humans from the Peştera Muierii, Baia de Fier, Romania. *Proc. Natl. Acad. Sci. U.S.A.* **103**, 17196-17201 (2006).

31 Soficaru, A., Petrea, C., Doboş, A. & Trinkaus, E. The human cranium from the Peştera Cioclovina Uscată, Romania: context, age, taphonomy, morphology, and paleopathology. *Current Anthropology* **48**, 611-619 (2007).

32 Sinitsyn, A. & Hoffecker, J. Radiocarbon dating and chronology of the Early Upper Paleolithic at Kostenki. *Quaternary International* **152**, 164-174 (2006).

33 di Cesnola, A. P. L'Aurignacien et le Gravettien ancien de la grotte Paglicci au Mont Gargano. *L'anthropologie* **110**, 355-370 (2006).

34 Fewlass, H. *et al.* Direct radiocarbon dates of mid Upper Palaeolithic human remains from Dolní Věstonice II and Pavlov I, Czech Republic. *Journal of Archaeological Science: Reports* **27**, 102000 (2019).

35 Simon, U., Händel, M., Einwögerer, T. & Neugebauer-Maresch, C. The archaeological record of the Gravettian open air site Krems-Wachtberg. *Quaternary International* **351**, 5-13 (2014).

36 Richter, D., Tostevin, G. & Škrdla, P. Bohunician technology and thermoluminescence dating of the type locality of Brno-Bohunice (Czech Republic). *Journal of Human evolution* **55**, 871-885 (2008).

37 Richter, D., Tostevin, G., Škrdla, P. & Davies, W. New radiometric ages for the Early Upper Palaeolithic type locality of Brno-Bohunice (Czech Republic): comparison of OSL, IRSL, TL and 14C dating results. *Journal of Archaeological science* **36**, 708-720 (2009).

38 Nejman, L. *et al.* New chronological evidence for the Middle to Upper Palaeolithic transition in the Czech Republic and Slovakia: new optically stimulated luminescence dating results. *Archaeometry* **53**, 1044-1066 (2011).

39 Tsanova, T. Les débuts du Paléolithique supérieur dans l'Est des Balkans. *Réflexion à partir des* (2008).

40 Škrdla, P. & Nikolajev, P. Preliminary comparison of Kulychivka (lower layer) and the Moravian Bohunician. *Матеріали і дослідження з археології Прикарпаття і Волині*, 78-96 (2014).

41 Škrdla, P., Sytnyk, O. & Koropets'kyi, R. New observations concerning Kulychivka site, layer IV. *Матеріали і дослідження з археології Прикарпаття і Волині*, 15-25 (2016).

42 Gladilin, V. & Demidenko, Y. E. Upper Palaeolithic stone tool complexes from Korolevo. *Anthropologie (1962-)*, 143-178 (1989).

43 Koulakovska, L., Usik, V. & Haesaerts, P. Early paleolithic of korolevo site (Transcarpathia, Ukraine). *Quaternary International* **223**, 116-130 (2010).

44 Hoffecker, J. F. *et al.* The dating of a Middle Paleolithic blade industry in southern Russia and its relationship to the Initial Upper Paleolithic. *Journal of Paleolithic Archaeology* **2**, 381-417 (2019).

45 Kuhn, S. L. Questions of complexity and scale in explanations for cultural transitions in the Pleistocene: a case study from the early Upper Paleolithic. *Journal of Archaeological Method and Theory* **20**, 194-211 (2013).

46 Kuhn, S. L., Stiner, M. C. & Güleç, E. Initial Upper Palaeolithic in south-central Turkey and its regional context: a preliminary report. *ANTIQUITY-OXFORD* **73**, 505-517 (1999).

47 Ploux, S. & Soriano, S. Umm el Tlel, une séquence du Paléolithique supérieur en Syrie centrale. Industries lithiques et chronologie culturelle. *Paléorient*, 5-34 (2003).

48 Richter, D., Rink, W., Schwarcz, H., Julig, P. & Schroeder, H. The Middle to Upper Palaeolithic transition in the Levant and new thermoluminescence dates for a Late Mousterian assemblage from Jerf Al-Ajla Cave (Syria). *Paléorient*, 29-46 (2001).

49 Pastoors, A., Weniger, G.-C. & Kegler, J. F. The Middle-Upper Palaeolithic transition at Yabroud II (Syria). A re-evaluation of the lithic material from the Rust excavation. *Paléorient*, 47-65 (2008).

50 Leder, D. Lithic variability and techno-economy of the Initial Upper Palaeolithic in the Levant. *International Journal of Archaeology* **6**, 23-36 (2018).

51 Williams, J. & Bergman, C. The 1937–1938 and 1947–1948 Excavations of Levels XIII-VI at Ksar Akil, Lebanon. *Paléorient* **36**, 117-161 (2010).

52 Garrod, D. A. The Mugharet el-Emireh in Lower Galilee: type-station of the Emiran industry. *The Journal of the Royal Anthropological Institute of Great Britain and Ireland* **85**, 141-162 (1955).

53 Barzilai, O. & Gubenko, N. Rethinking Emireh Cave: the lithic technology perspectives. *Quaternary International* **464**, 92-105 (2018).

54 Garrod, D. A. A transitional industry from the base of the Upper Palaeolithic in Palestine and Syria. *Journal of the Anthropological Institute of Great Britain and Ireland*, 121-130 (1951).

55 Sarel, J. The middle-upper Paleolithic transition in Israel. *BAR International Series* **1229** (2004).

56 Marks, A. E. Reflections on Levantine Upper Palaeolithic studies: past and present. *More than meets the eye: Studies on Upper Palaeolithic diversity in the Near East*, 249-264 (2003).

57 Shunkov, M. V., Fedorchenko, A. Y., Kozlikin, M. B. & Derevianko, A. P. Initial Upper Palaeolithic ornaments and formal bone tools from the East Chamber of Denisova Cave in the Russian Altai. *Quaternary International* (2020).

58 Goebel, T., Derevianko, A. P. & Petrin, V. T. Dating the middle-to-upper-paleolithic transition at Kara-Bom. *Current anthropology* **34**, 452-458 (1993).

59 Slavinskiy, V. Industrii Ranneverhinepaleoliticheskii Ourovney Ovitanya Ctoyanki Ust-Karakol 1 (Early Upper Paleolithic Industry from the occupation levels at Ust-Karakol 1). *Severnaya Evrasia v Anthropologenie. ChelovekPaleolitechnologii, Geoecologia, Ethnografii i Anthropologii*, 197-214 (2007).

60 Derevianko, A. P. *et al.* Paleolithic of the Altai, Richard Liu Foundation. *European Institute of Chinese Studies Occasional Papers* **1** (2001).

61 Goebel, T. & Aksenov, M. Accelerator radiocarbon dating of the initial Upper Palaeolithic in southeast Siberia. *Antiquity* **69**, 349-357 (1995).

62 Orlova, L., Kuzmin, Y. & Lbova, L. Radiocarbon Dates of the Paleolithic and Mesolithic Sites in Transbaikal and Mongolia. *The Paleolithic Cultures of Transbaikal and Mongolia: New Facts, Methods, and Hypotheses*, 88-92 (2005).

63 Lbova, L. Chronology and paleoecology of the Early Upper Paleolithic in the Transbaikal region (Siberia). *Eurasian Prehistory* **5** (2008).

64 Kuzmin, Y. V., Lbova, L. V., Jull, A. T. & Cruz, R. J. The Middle-to-Upper-Paleolithic transition in Transbaikal, Siberia: the Khotyk site chronology and archaeology. *Current Research in the Pleistocene* **23** (2006).

65 Tashak, V. in *Arkheologiya I Kulturnaya Antropologiya Dalnego Vostoka I Tsentralnoi Azii. Vladivostok* 25-33 (2002).

66 Antonova, Y. E. & Tashak, V. Stone Industry of Podzvonkaya South-East Complex: General Characterization. *The bulletin of Irkutsk State University.«Geoarchaeology, Ethnology, and Anthropology Series»*, 3-20 (2016).

67 Derevianko, A. *et al.* Early upper paleolithic stone tool technologies of northern Mongolia: the case of Tolbor-4 and Tolbor-15. *Archaeology, Ethnology and Anthropology of Eurasia* **41**, 21-37 (2013).

68 Zwyns, N. *Laminar technology and the onset of the Upper Paleolithic in the Altai, Siberia*. (Leiden University Press Leiden, 2012).

69 Zwyns, N. *et al.* The open-air site of Tolbor 16 (Northern Mongolia): Preliminary results and perspectives. *Quaternary International* **347**, 53-65 (2014).

70 Derevianko, A., Brantingham, P. J., Olsen, J. W. & Tseveendorj, D. in *The Early Upper Paleolithic Beyond Western Europe* 207-222 (University of California Press, 2004).

71 Li, F., Kuhn, S. L., Gao, X. & Chen, F.-y. Re-examination of the dates of large blade technology in China: A comparison of Shuidonggou Locality 1 and Locality 2. *Journal of Human Evolution* **64**, 161-168 (2013).

**Table S1.1 Modern human specimens older than ~30,000 years cal. BP from which genome-wide data has been generated and their respective radiocarbon dates.** Archaeological sites from which these specimens originate are plotted in Fig. 1. Radiocarbon dates calibrated with IntCal20[7] and OxCal 4.4.2[6].

| **Specimen** | **Radiocarbon date ID** | **Raw radiocarbon date BP** | **Date type and publication** | **Calibrated date with IntCal20[6] and OxCal 4.4.2, cal. BP 95.4%** | **Mean date, cal. BP, IntCal20 [6]and OxCal 4.4.2** |
|---|---|---|---|---|---|
| Bacho Kiro CC7-335 | ETH-86772 | 42,450±510 | Direct-UF[3,4] | 45,930-44,420 | 45,120 |
| Bacho Kiro BB7-240 | ETH-86770 | 41,850±480 | Direct-UF[3,4] | 45,550-43,940 | 44,690 |
| Ust'Ishim | OxA-25516 & 30190 | 41,400±950 | Direct-UF[15] | 45,930-42,900 | 44,360 |
| Bacho Kiro CC7-2289 | ETH-86771 | 40,600±420 | Direct-UF[3,4] | 44,400-42,990 | 43,690 |
| Bacho Kiro AA7-738 | ETH-86769 | 39,750±380 | Direct-UF[3,4] | 43,930-42,580 | 43,110 |
| Oase1 | GrA-22810 and OxA-11711 | 34,950±900 | Direct-UF[14] | 41,860-37,950 | 40,020 |
| Tianyuan | BA-03222 | 34,430±510 | ?[27] | 40,850-38,070 | 39,570 |
| Kostenki14 | OxA-X-2395-15 | 33,250±500 | HYP[28] | 39,380-36,670 | 38,050 |
| Bacho Kiro F6-597 | ETH-86773, AIX-12025 | 31,660±140 | Direct-UF[3,4] | 36,320-35,600 | 36,000 |
| Goyet Q116-1 | GrA-46175 | 30,880±170 | Direct-noUF[29] | 35,630-34,720 | 35,210 |
| Bacho Kiro BK-1653 | ETH-86768, AIX-12024 | 30,570±120 | Direct-UF[3,4] | 35,290-34,610 | 34,950 |
| Sunghir 2 | OxA-X-2395-6 | 30,100±550 | Direct-HYP[25] | 35,790-33,430 | 34,610 |
| Sunghir 3 | OxA-X-2395-7 | 30,000±550 | Direct-HYP[25] | 35,640-33,270 | 34,520 |
| Sunghir 4 | OxA-X-2462-52 | 29,820±280 | Direct-HYP[25] | 34,850-33,720 | 34,320 |
| Muierii2 | OxA-16252 | 29,110±190 | Direct-UF[30] | 34,180-33,140 | 33,660 |
| Sunghir 1 | OxA-X-2464-12 | 28,890±430 | Direct-HYP[25] | 34,280-32,020 | 33,270 |
| Cioclovina1 | OxA-15527 | 28,510±170 | Direct-UF[31] | 33,300-32,010 | 32,660 |
| Kostenki12 | GrA-5552 | 28,500±140 | layer date[32] | 33,210-32,070 | 32,650 |
| Paglicci133 | UTC-1789 and UTC-1415 | 28,100±400 and 29,300±600 | layer date[33] | 33,370-31,230 | 32,260 |
| Yana RHS | Beta-271412 and Beta-230442 | 27,940±115 | layer date[26] | 32,190-31,530 | 31,870 |
| Vestonice16 | Aix12030 | 27,220±110 | Direct-UF[34] | 31,530-31,080 | 31,250 |
| Vestonice43 | Aix12032 | 27,070±110 | Direct-UF[34] | 31,280-31,030 | 31,150 |
| Vestonice13 | Aix12027 | 27,040±100 | Direct-UF[34] | 31,240-31,030 | 31,140 |
| KremsWA3 | VERA-3941 | 26,870±220 | layer date[35] | 31,360-30,430 | 31,020 |
| Vestonice14 | Aix12028 | 26,760±100 | Direct-UF[34] | 31,150-30,860 | 31,010 |
| Vestonice15 | Aix12029 | 26,680±70 | Direct-UF[34] | 31,110-30,840 | 30,980 |
| Pavlov1 | Aix12026 | 25,490±90 | Direct-UF[34] | 30,050-29,320 | 29,800 |

**Table S1.2 Archaeological sites with layers containing Initial Upper Palaeolithic (IUP) assemblage.** These sites are plotted in Fig. 1, demonstrating distribution of IUP archaeological sites in Eurasia, and modified after Kuhn and Zwyns[18].

| Number on the map in Fig. 1 | Archaeological site with IUP | Country | Reference |
|---|---|---|---|
| 19 | Brno-Bohunice | Czech Republic | [36,37] |
| 20 | Stránská Skála III | Czech Republic | [36,38] |
| 19 | Bohunice-Kejbaly I, II | Czech Republic | [36] |
| 21 | Temnata | Bulgaria | [39] |
| 1 | Bacho Kiro Cave | Bulgaria | [3,4] |
| 22 | Kulychivka | Ukraine | [40,41] |
| 23 | Korolevo 1, 2 | Ukraine | [42,43] |
| 24 | Shlyakh | Russia | [44] |
| 25 | Üçagizli Cave | Turkey | [19,45] |
| 26 | Kanal Cave | Turkey | [46] |
| 27 | Um el'Tlel | Syria | [47] |
| 28 | Jerf Ajlah | Syria | [48] |
| 29 | Yabrud II | Syria | [49] |
| 30 | Antelias | Lebanon | [50] |
| 31 | Abou Halka | Lebanon | [50] |
| 32 | Ksar Akil rockshelter | Lebanon | [51] |
| 33 | Emireh Cave | Israel | [52,53] |
| 34 | El Wad | Israel | [54] |
| 35 | Raqefet | Israel | [55] |
| 36 | Boker Tachtit | Israel | [56] |
| 37 | Denisova Cave | Russia | [57] |
| 38 | Kara-Bom | Russia | [58] |
| 39 | Ust-Karakol 1 | Russia | [59] |
| 40 | Kara-Tenesh | Russia | [60] |
| 41 | Makarvo IV | Russia | [61] |
| 42 | Kamenka A-C | Russia | [62,63] |
| 43 | Khotyk | Russia | [63,64] |
| 44 | Podzvonkaya | Russia | [65,66] |
| 45 | Tolbor 4 | Mongolia | [67,68] |
| 46 | Tolbor 16 | Mongolia | [69] |
| 47 | Tsagan-Agui | Mongolia | [70] |
| 48 | Suindonggou 1 | China | [71] |
| 49,50 | Suindonggou 2, 9 | China | [71] |

# Supplementary Information 2

## Ancient DNA processing, quality controls and contamination estimates

### DNA extraction and library preparation

Data generation for the seven Bacho Kiro Cave specimens (specimen IDs*: F6-620, AA7-738, BB7-240, CC7-2289, CC7-335, F6-597* and *BK1653*) was based on DNA libraries prepared previously and detailed in Online Materials and Methods of *Hublin et al.*[1]. In short, five single-stranded DNA libraries[2] were prepared for each specimen on an automated liquid handling platform (Bravo NGS workstation B, Agilent Technologies)[3] by using ten µL of DNA extract as input, tagged with two unique index sequences[4,5] and amplified into plateau using AccuPrime Pfx DNA polymerase (Life Technologies)[6]. This amounted to a total of 35 libraries for Bacho Kiro Cave specimens (Tab. S2.1).

In order to generate additional data from *Oase1*, we extracted DNA from 15 mg of bone powder of the *Oase1* mandible[7,8]. Since the specimen was previously found to be highly contaminated with microbial and present-day human DNA, the bone powder was treated with 0.5% hypochlorite solution prior to DNA extraction[4]. Four single-stranded DNA libraries were prepared from the resulting extract in the same way as described above. Two additional libraries were prepared, each using 5 µL of the two DNA extracts generated previously[8] (E1406 and E1843) (Tab. S2.1).

All Bacho Kiro Cave and *Oase1* libraries were made omitting UDG treatment to maximize the recovery of endogenous DNA and to preserve deamination signals. Fifty µL of each of the amplified libraries were purified on an automated liquid handling platform using SPRI beads[3], and their concentration was determined on a NanoDrop 1000 Spectrophotometer (NanoDrop Technologies). To assess nuclear DNA preservation, the purified libraries were pooled together with libraries from other experiments and one-cycle PCR reaction with Herculase II Fusion DNA polymerase (Agilent Technologies)[7] and IS5 and IS6 primers[2,9] was used to remove heteroduplex formations. The pools of libraries were then sequenced directly on Illumina MiSeq or HiSeq 2500 platforms in a double index configuration (2x76 cycles)[5] and base calling was done using Bustard (Illumina).

**Sequencing and alignment to the nuclear genome**

For all sequencing runs we trimmed the adapters and merged overlapping forward and reverse reads into single sequences using *leeHom*[10] (version: https://bioinf.eva.mpg.de/leehom/). The Burrows-Wheeler Aligner (BWA, version: 0.5.10-evan.9-1-g44db244; https://github.com/mpieva/network-aware-bwa) with the parameters adjusted for ancient DNA ("-n 0.01 –o 2 –l 16500")[11] was used to align the data from all sequencing runs to the human reference genome (GRCh37/1000 Genomes release; ftp://ftp.1000genomes.ebi.ac.uk/vol1/ftp/technical/reference/phase2_reference_assembly_sequence/). Only reads that showed perfect matches to the expected index combinations were used for all downstream analyses. PCR duplicates were removed using *bam-rmdup* (version: 0.6.3; https://github.com/mpieva/biohazard-tools) and *SAMtools* (version: 1.3.1)[12] were used to filter for fragments that were at least 35 bp long and that had a mapping quality equal or greater than 25.

**Mitochondrial DNA capture and sequencing**

Mitochondrial DNA (mtDNA) captures of Bacho Kiro Cave libraries and their processing are detailed in *Hublin et al.*[1]. An aliquot of each newly generated *Oase1* library was also enriched for human mtDNA using a bead-based hybridization method[13]. Enriched libraries were pooled with libraries from other experiments and sequenced on an Illumina MiSeq platform in a double index configuration (2x76 cycles)[5]. Trimming of the adapters, merging of forward and reverse reads and the alignment parameters were the same as for the nuclear DNA data detailed above, with the difference of using the revised Cambridge Reference Sequence (NC_01290) for the alignment of the mtDNA data. The reads with perfect matches to the expected index combinations were retained for downstream analyses and PCR duplicates were removed using *bam-rmdup* (version: 0.6.3; https://github.com/mpieva/biohazard-tools). We used *SAMtools* (version: 1.3.1)[12] to merge libraries originating from the same extract, and filter for fragments of at least 35 bp with a mapping quality of 25 or greater.

**In solution hybridization capture of nuclear DNA**

We estimated the genomic coverage in each library that could be obtained by sequencing a library to exhaustion ("nuclear DNA content") by using the number of library molecules determined by quantitative PCR (qPCR) and the fraction of sequences of at least 35 base pairs (bp) that could be mapped to the human reference genome (Tab. S2.1)[9]. We selected the libraries with estimated genomic coverage of at least 1% after restricting the analyses to the

fragments showing cytosine (C) to thymine (T) substitutions at the first three and/or the last three positions of the alignment ends for further in solution hybridization captures to oligonucleotide probes[13].

Given the low nuclear DNA content in the Bacho Kiro Cave *F6-597* specimen (Tab. S2.1), the absence of C-to-T substitutions after restricting the analyses to sequences carrying a C-to-T substitution at the opposing end[14] ("conditional" substitutions, Tab. S2.2 and Extended Data Fig. 1) and high levels of present-day human DNA contamination as determined based on mitochondrial DNA analyses[1], we did not attempt in solution hybridization capture for this specimen.

We enriched the selected amplified libraries for ~3.7 million single nucleotide polymorphisms (SNPs) across the genome detailed in the Supplementary Data 2 of *Haak et al.*[15] (SNP Panel 1 or "390k" array), and Supplementary Data 1, 2 and 3 of *Fu et al.*[8] (SNP Panels 2, 3 and 4, or "840k", "1000k" and "Archaic admixture" arrays, respectively). For the identified male individuals (*F6-620, BB7-240* and *CC7-335;* see Supplementary Information 4), an aliquot of each library was additionally enriched for ~6.9 Mb of the Y chromosome[16]. All of the enriched libraries were sequenced on the Illumina HiSeq 2500 platforms in a double index configuration (2x76 cycles)[5] and base calling was done using Bustard (Illumina).

**Processing of nuclear DNA capture data**

In addition to the steps described in the "Sequencing and alignment to the nuclear genome" section above, BAM files of the libraries enriched for the specific subset of the nuclear genome were intersected with the BED files containing target SNP positions ("390k", "840k", "1000k", "Archaic admixture", a merged set of SNP Panels 1 and 2 or "1240k", and a merged set of SNP Panels 1, 2 and 3 or "2200k") and regions (Y chromosome) using *BEDtools*[17] (version: 2.24.0). In order to filter for endogenous ancient DNA or putatively deaminated fragments, we used elevated C-to-T substitutions relative to the reference genome at the first three and/or last three positions of the alignment ends[14]. We merged the libraries originating from the same specimen using *samtools merge*[12] to produce the final datasets for downstream analyses. Tables S2.3-S2.8 summarize the number of filter-passed fragments and SNPs covered per specimen for each library as well as for the merged data of all libraries stemming from the same specimen depending on the SNP Panel used. Extended Data Tab. 1 summarizes the number of filter-passed fragments and SNPs covered in the final merged dataset used for downstream analyses.

## Ancient DNA substitution patterns

For each specimen we analysed substitution patterns along the fragments for all libraries separately (Tab. S2.2) and for the merged data of all libraries originating from the same specimen (Extended Data Fig. 1) by counting the number of substitutions relative to the human reference genome. As deamination of cytosine (C) to uracil (U) residues results in characteristic C-to-T substitutions in ancient DNA molecules[18], elevated C-to-T substitutions close to the alignment ends provide evidence for the presence of authentic ancient DNA in specimens[19,20]. The C-to-T substitution frequencies ranged between 23.11% and 55.78% on the 5'-ends, and between 15.13% and 43.02% on the 3'-ends of the enriched libraries (Extended Data Fig. 1). These frequencies increased substantially when filtering for sequences with a C-to-T at the opposing end ("conditional" substitutions)[14] for the Bacho Kiro Cave specimens *AA7-738* and *CC7-2289* and *Oase1*, indicating that both endogenous ancient DNA as well some present-day human DNA contamination are present[14]. For the other specimens these frequencies remained stable, indicating that the majority of the data stems from one population of sequences.

## Random read sampling and consensus calling

We performed random read sampling using *bam-caller* (https://github.com/bodkan/bam-caller, version: 0.1) by picking a base with a base quality of at least 30 at each position in the "1240k" and "2200k" SNP Panels (Supplementary Information 2) that was covered by at least one fragment longer than 35 bp with a mapping quality equal or higher than 25 (L≥35bp, MQ≥25, BQ≥30). To mitigate the effect of deamination-derived substitutions on downstream analyses, we did not sample any Ts on the forward strands (in the orientation as sequenced) and any As on the reverse strands in the first three and/or last three positions from the alignment ends. Furthermore, we repeated each of the analyses using only transversion polymorphisms to make sure our inferences are robust to any aDNA damage.

Due to the haploid nature of the Y chromosome, we called genotypes across the ~6.9 Mb of the Y chromosome for the enriched libraries of male individuals by calling a consesus allele at each position by majority call requiring a minimum coverage of 3 for the specimens *F6-620* and *BB7-240* and of 2 for the specimen *CC7-335* using using *bam-caller* (https://github.com/bodkan/bam-caller, version: 0.1). Random allele calls of Bacho Kiro Cave individuals and *Oase1* for different SNP Panels and consensus calls for Y chromosome regions, both for all and putatively deaminated fragments are available in EIGENSTRAT format at http://cdna.eva.mpg.de/modern_human/BachoKiro/.

**Contamination estimates**

In addition of all analysed libraries needing to have a damage profile consistent with aDNA (Extended Data Fig. 1), we used four complementary approaches in estimating the proportion of present-day human DNA contamination in the generated data of Bacho Kiro Cave specimens and *Oase1*. The estimates of present-day human DNA contamination are summarized in the Table S2.10.

<u>Mitochondrial DNA contamination estimates.</u>

We analysed mitochondrial DNA (mtDNA) data from Bacho Kiro Cave specimens in a previous study[1] and estimated the proportion of present-day human DNA contamination to range between 0.2% to 2.9% among all fragments of specimens *F6-620, AA7-738, CC7-2289, CC7-335* and *BK1653,* and between 0% and 1.6% among putatively deaminated fragments, respectively. We estimated the proportion of present-day human DNA contamination among all fragments of the specimen *F6-597* to be 63.5% (95% confidence intervals (CI): 62.5-64.5%)[2] using *schmutzi*[21] (version: 1.5.5). Given the high contamination estimates[1] and low nuclear DNA content (<1% among putatively deaminated fragments (Tab. S2.1)), we excluded the libraries of the specimen *F6-597* from nuclear captures and downstream analyses.

We estimated proportion of present-day human DNA contamination in the newly generated *Oase1* libraries using six previously identified positions[8] where *Oase1* mtDNA genome differs from 99% of a world-wide panel of 311 present-day human mtDNAs[22]. We counted the number of recovered mtDNA fragments that cover these positions, while taking into account the strand orientation in cases where one of the diagnostic positions was a C or a G[14]. The proportion of present-day human DNA contamination among all fragments in the libraries prepared from two previously generated extracts[8] are 64.29% (95% CI: 58.36%-69.80%) and 71.96% (95% CI: 66.59%-76.77%), respectively, and 46.22% (95% CI: 40.50%-52.39%) in the libraries prepared from a new extract, indicating that the hypochlorite treatment did not substantially reduce the amount of present-day human DNA contamination. The proportion of present-day human DNA contamination among putatively deaminated fragments is 8.70% (95% CI: 2.42%-14.98%) and 9.38% (95% CI: 3.24%-15.52%) for the new libraries prepared from the two previously generated extracts[8] and 4.51% (95% CI: 0%-9.34%) for the libraries prepared from the new extract.

Contamination estimates based on the X chromosome polymorphisms in males.

We used ANGSD[23,24] (version: 0.929) to estimate contamination in male individuals where we have sufficient X coverage, i.e. at least 200 SNPs covered by at least two fragments[25]. Since males have only one copy of X chromosome, we do not expect polymorphisms in this part of their genome. For the *F6-620*, *BB7-240* and *CC7-335* contamination estimates inferred by ANGSD are 1.62% (standard error (SE): 0.12), 2.75% (SE: 0.37) and 3.41% (SE: 0.53) among all fragments, and 1.09% (SE: 0.40), 1.27% (SE: 1.03) and 2.13% (SE: 0.94) among deaminated fragments, respectively.

Contamination estimates based on the ancient DNA damage.

We used AuthentiCT[26] (version: 1.0.0), an approach that utilizes patterns of aDNA damage, to estimate the proportion of present-day human DNA contamination among nuclear sequences of the enriched libraries. Among the sequences overlapping "2200k" SNP Panel, the inferred proportion of present-day human DNA contamination ranges between 2.23% (standard error (SE): 0.54%, *F6-620*) and 42.36% (SE: 0.64%, *CC7-2289*) (Tab. S2.10).

Consistency of results from all fragments and deaminated fragments.

To test for evidence of contamination, we computed D-statistics using ADMIXTOOLS[27] (version: v5.1) as implemented in the R-package *admixr*[28] (version: 0.7.1) on SNPs from nuclear capture Panels 1, 2 and 3 ("2200k") covered by both all and putatively deaminated fragments[8,25] for specimens *F6-620*, *BB7-240*, *CC7-335* and *BK1653*. The number of overlapping informative sites between all and deaminated fragments for specimens *AA7-738* and *CC7-2289* is too low to confidently perform this test. We computed *D(BK specimen*$_1$ *all, BK specimen*$_1$ *deaminated; Test, Mbuti)* where *Test* is a present-day human population from Simons Genome Diversity Project (SGDP)[29] and three Mbuti individuals from SGDP are used as outgroup. We used a Weighted Block Jackknife[27,30] and a block size of 5 million base pairs (5 Mb) across all autosomes on the "2200k" SNP Panel to calculate these statistics.

Figures S2.1 ad S2.2 show there is no significant difference between using all fragments and restricting the analyses to deaminated fragments for *F6-620*, *BB7-240*, *CC7-335* and *BK1653* (all $|Z| < 3$). However, given the higher contamination estimates among all fragments of *BB7-240*, *CC7-335*, *CC7-2289* and *AA7-738* using AuthentiCT[26] (version: 1.0.0) (Tab. S2.10), we restricted all downstream analyses to deaminated fragments for these specimens, unless otherwise stated.

**References SI2:**


1 Hublin, J.-J. et al. Initial Upper Palaeolithic Homo sapiens from Bacho Kiro Cave, Bulgaria. *Nature* **581**, 299-302, doi:10.1038/s41586-020-2259-z (2020).

2 Gansauge, M. T. *et al.* Single-stranded DNA library preparation from highly degraded DNA using T4 DNA ligase. *Nucleic Acids Res.* **45**, e79, doi:10.1093/nar/gkx033 (2017).

3 Slon, V. *et al.* Neandertal and Denisovan DNA from Pleistocene sediments. *Science* **356**, 605-608, doi: 10.1126/science.aam9695 (2017).

4 Korlević, P. *et al.* Reducing microbial and human contamination in DNA extractions from ancient bones and teeth. *Biotechniques* **59**, 87-93, doi:10.2144/000114320 (2015).

5 Kircher, M., Sawyer, S. & Meyer, M. Double indexing overcomes inaccuracies in multiplex sequencing on the Illumina platform. *Nucleic Acids Res.* **40**, e3, doi:10.1093/nar/gkr771 (2012).

6 Dabney, J. & Meyer, M. Length and GC-biases during sequencing library amplification: a comparison of various polymerase-buffer systems with ancient and modern DNA sequencing libraries. *Biotechniques* **52**, 87-94, doi:10.2144/000113809 (2012).

7 Trinkaus, E. *et al.* An early modern human from the Peştera cu Oase, Romania. *Proc. Natl. Acad. Sci. U.S.A.* **100**, 11231-11236, doi: 10.1073/pnas.2035108100 (2003).

8 Fu, Q. *et al.* An early modern human from Romania with a recent Neanderthal ancestor. *Nature* **524**, 216-219, doi:10.1038/nature14558 (2015).

9 Glocke, I. & Meyer, M. Extending the spectrum of DNA sequences retrieved from ancient bones and teeth. *Genome Res.* **27**, 1230-1237, doi: 10.1101/gr.219675.116 (2017).

10 Renaud, G., Stenzel, U. & Kelso, J. leeHom: adaptor trimming and merging for Illumina sequencing reads. *Nucleic Acids Res.* **42**, e141, doi:10.1093/nar/gku699 (2014).

11 Meyer, M. *et al.* A high-coverage genome sequence from an archaic Denisovan individual. *Science* **338**, 222-226, doi:10.1126/science.1224344 (2012).

12 Li, H. *et al.* The Sequence Alignment/Map format and SAMtools. *Bioinformatics* **25**, 2078-2079, doi:10.1093/bioinformatics/btp352 (2009).

13 Fu, Q. *et al.* DNA analysis of an early modern human from Tianyuan Cave, China. *Proc. Natl. Acad. Sci. U.S.A.* **110**, 2223-2227, doi:10.1073/pnas.1221359110 (2013).

14 Meyer, M. *et al.* A mitochondrial genome sequence of a hominin from Sima de los Huesos. *Nature* **505**, 403-406, doi:10.1038/nature12788 (2014).

15 Haak, W. *et al.* Massive migration from the steppe was a source for Indo-European languages in Europe. *Nature* **522**, 207-211, doi:10.1038/nature14317 (2015).

16 Petr, M. *et al.* The evolutionary history of Neanderthal and Denisovan Y chromosomes. *Science* **369**, 1653-1656, doi: 10.1126/science.abb6460 (2020).

17 Quinlan, A. R. & Hall, I. M. BEDTools: a flexible suite of utilities for comparing genomic features. *Bioinformatics* **26**, 841-842, doi: 10.1093/bioinformatics/btq033 (2010).

18 Briggs, A. W. *et al.* Patterns of damage in genomic DNA sequences from a Neandertal. *Proc. Natl. Acad. Sci. U.S.A.* **104**, 14616-14621, doi:10.1073/pnas.0704665104 (2007).

19 Krause, J. *et al.* A complete mtDNA genome of an early modern human from Kostenki, Russia. *Curr. Biol.* **20**, 231-236, doi:10.1016/j.cub.2009.11.068 (2010).

20 Sawyer, S., Krause, J., Guschanski, K., Savolainen, V. & Paabo, S. Temporal patterns of nucleotide misincorporations and DNA fragmentation in ancient DNA. *PLoS One* **7**, e34131, doi:10.1371/journal.pone.0034131 (2012).

21 Renaud, G., Slon, V., Duggan, A. T. & Kelso, J. Schmutzi: estimation of contamination and endogenous mitochondrial consensus calling for ancient DNA. *Genome Biol.* **16**, 224, doi:10.1186/s13059-015-0776-0 (2015).

22 Green, R. E. *et al.* A complete Neandertal mitochondrial genome sequence determined by high-throughput sequencing. *Cell* **134**, 416-426, doi:10.1016/j.cell.2008.06.021 (2008).

23 Korneliussen, T. S., Albrechtsen, A. & Nielsen, R. ANGSD: analysis of next generation sequencing data. *BMC bioinformatics* **15**, 356 (2014).

24 Rasmussen, M. *et al.* An Aboriginal Australian genome reveals separate human dispersals into Asia. *Science* **334**, 94-98 (2011).

25 Fu, Q. *et al.* The genetic history of Ice Age Europe. *Nature* **534**, 200-205, doi:10.1038/nature17993 (2016).

26 Peyrėgne, S. & Peter, B. M. AuthentiCT: a model of ancient DNA damage to estimate the proportion of present-day DNA contamination. *Genome Biol.* **21**, 246, doi: 10.1186/s13059-020-02123-y (2020).

27 Patterson, N. *et al.* Ancient admixture in human history. *Genetics* **192**, 1065-1093, doi:10.1534/genetics.112.145037 (2012).

28 Petr, M., Vernot, B. & Kelso, J. admixr—R package for reproducible analyses using ADMIXTOOLS. *Bioinformatics* **35**, 3194-3195 (2019).

29 Mallick, S. *et al.* The Simons Genome Diversity Project: 300 genomes from 142 diverse populations. *Nature* **538**, 201-206, doi:10.1038/nature18964 (2016).

30 Busing, F. M. T. A., Meijer, E. & Van Der Leeden, R. Delete-m jackknife for unequal m. *Statistics and Computing* **9**, 3-8, doi: 10.1023/A:1008800423698 (1999).

## Table S2.1 The characteristics of Bacho Kiro Cave and *Oase1* ssDNA libraries and summary statistics of the shotgun data.

| Specimen | Specimen ID | Amount of powder used for DNA extraction (mg) | Extract ID | Volume of extract used for library preparation (µL) | Library ID | Total number of DNA molecules in the library* | Number of spike-in molecules in the library* | Number of sequenced fragments | Number of fragments ≥35 bp | Number of mapped fragments ≥35bp, MQ≥25 | Number of unique fragments ≥35bp, MQ≥25 | % mapped fragments ≥35bp, MQ≥25 | Average fragment length | Estimated coverage in the library (all fragments) | Number of deaminated fragments ≥35bp, MQ≥25 | Average deaminated fragment length | Estimated coverage in the library (deaminated fragments) |
|---|---|---|---|---|---|---|---|---|---|---|---|---|---|---|---|---|---|
| **Bacho Kiro F6-620** | SP6931 | 29.3 | E9293 | 10 | A11197 | 3.18E+10 | 1.11E+06 | 2,403,103 | 1,438,335 | 19,650 | 19,571 | **1.366** | 58.50 | **6.31** | 4,929 | 56.20 | **6.11** |
| | | | | 10 | A12357 | 2.05E+10 | 1.42E+06 | 581,401 | 342,184 | 4,384 | 4,383 | **1.281** | 59.00 | **3.72** | 1,047 | 57.20 | **3.52** |
| | | | | 10 | A12550 | 1.48E+10 | 8.29E+05 | 2,927,791 | 1,728,732 | 23,601 | 23,529 | **1.365** | 59.20 | **2.91** | 5,702 | 57.20 | **2.74** |
| | | | | 10 | A12944 | 1.74E+10 | 9.93E+05 | 2,750,462 | 1,695,480 | 19,411 | 19,336 | **1.145** | 59.30 | **3.01** | 4,715 | 57.80 | **2.88** |
| | | | | 10 | A15716 | 2.86E+10 | 1.44E+06 | 1,348,729 | 846,882 | 9,854 | 9,806 | **1.164** | 59.90 | **5.13** | 2,310 | 58.20 | **4.75** |
| **Bacho Kiro AA7-738** | SP7098 | 30.4 | E9294 | 10 | A11198 | 2.84E+09 | 1.07E+06 | 2,084,489 | 1,256,707 | 312 | 312 | **0.025** | 45.10 | **0.01** | 124 | 45.30 | **0.01** |
| | | | | 10 | A12358 | 4.58E+09 | 1.34E+06 | 535,634 | 321,994 | 100 | 100 | **0.031** | 46.70 | **0.02** | 32 | 45.80 | **0.02** |
| | | | | 10 | A12554 | 4.05E+09 | 7.37E+05 | 4,376,821 | 2,632,838 | 676 | 676 | **0.026** | 45.80 | **0.01** | 239 | 49.40 | **0.02** |
| | | | | 10 | A12945 | 5.88E+09 | 1.12E+06 | 1,446,743 | 913,218 | 170 | 170 | **0.019** | 45.90 | **0.02** | 48 | 45.10 | **0.01** |
| | | | | 10 | A15717 | 5.06E+09 | 9.95E+05 | 1,746,767 | 1,134,892 | 224 | 222 | **0.020** | 47.40 | **0.02** | 81 | 48.20 | **0.02** |
| **Bacho Kiro BB7-240** | SP7099 | 32.1 | E9295 | 10 | A11199 | 1.35E+10 | 2.21E+05 | 2,927,566 | 1,596,869 | 7,227 | 7,197 | **0.453** | 50.40 | **0.74** | 2,417 | 49.40 | **0.92** |
| | | | | 10 | A12359 | 8.39E+09 | 2.09E+05 | 542,128 | 277,697 | 1,160 | 1,160 | **0.418** | 50.80 | **0.40** | 367 | 49.70 | **0.47** |
| | | | | 10 | A12555 | 7.90E+09 | 1.82E+05 | 3,986,361 | 2,046,644 | 9,148 | 9,112 | **0.447** | 50.10 | **0.40** | 2,958 | 45.50 | **0.44** |
| | | | | 10 | A12946 | 1.32E+10 | 2.80E+05 | 2,719,208 | 1,520,238 | 6,012 | 5,995 | **0.395** | 51.50 | **0.66** | 1,989 | 50.70 | **0.82** |
| | | | | 10 | A15718 | 1.72E+10 | 3.12E+05 | 1,405,586 | 814,990 | 3,171 | 3,158 | **0.389** | 52.00 | **0.88** | 1,012 | 51.20 | **1.06** |
| **Bacho Kiro CC7-2289** | SP9100 | 34.3 | E9296 | 10 | A11200 | 5.60E+10 | 5.69E+05 | 2,549,574 | 1,524,941 | 41 | 41 | **0.003** | 47.60 | **0.03** | 10 | 45.70 | **0.02** |
| | | | | 10 | A12360 | 2.70E+10 | 9.17E+05 | 509,637 | 296,520 | 6 | 6 | **0.002** | 50.80 | **0.01** | 1 | 38.00 | **0.00** |
| | | | | 10 | A12556 | 2.12E+10 | 5.03E+05 | 3,539,956 | 2,049,743 | 76 | 75 | **0.004** | 47.10 | **0.01** | 20 | 49.30 | **0.01** |
| | | | | 10 | A12947 | 3.45E+10 | 7.84E+05 | 1,756,046 | 1,067,036 | 28 | 28 | **0.003** | 47.70 | **0.02** | 4 | 53.50 | **0.01** |
| | | | | 10 | A15719 | 3.56E+10 | 6.20E+05 | 1,289,201 | 818,443 | 19 | 19 | **0.002** | 43.10 | **0.02** | 5 | 42.20 | **0.01** |
| **Bacho Kiro CC7-335** | SP9101 | 52.4 | E9297 | 10 | A11201 | 3.07E+09 | 9.20E+05 | 2,919,402 | 1,772,388 | 11,887 | 11,832 | **0.671** | 49.70 | **0.27** | 4,300 | 49.30 | **0.37** |
| | | | | 10 | A12361 | 4.17E+09 | 1.62E+06 | 587,919 | 346,613 | 2,058 | 2,058 | **0.594** | 48.70 | **0.31** | 756 | 48.00 | **0.43** |
| | | | | 10 | A12557 | 2.81E+09 | 7.00E+05 | 3,317,600 | 2,027,299 | 11,656 | 11,623 | **0.575** | 49.60 | **0.21** | 4,232 | 66.20 | **0.39** |
| | | | | 10 | A12948 | 4.45E+09 | 1.15E+06 | 1,450,595 | 916,440 | 4,403 | 4,384 | **0.480** | 49.70 | **0.29** | 1,568 | 49.40 | **0.40** |
| | | | | 10 | A15720 | 6.90E+09 | 1.31E+06 | 2,061,774 | 1,339,133 | 6,297 | 6,269 | **0.470** | 49.60 | **0.45** | 2,282 | 49.30 | **0.63** |
| **Bacho Kiro F6-597** | SP9102 | 45.6 | E9298 | 10 | A11202 | 7.75E+10 | 7.46E+05 | 3,146,382 | 2,094,007 | 100 | 100 | **0.005** | 60.80 | **0.13** | 11 | 45.50 | **0.00** |
| | | | | 10 | A12362 | 4.76E+10 | 1.27E+06 | 540,795 | 346,748 | 16 | 16 | **0.005** | 61.80 | **0.06** | - | 54.50 | **0.00** |
| | | | | 10 | A12558 | 4.21E+10 | 6.55E+05 | 4,011,897 | 2,566,726 | 100 | 100 | **0.004** | 60.70 | **0.05** | 4 | 86.90 | **0.00** |
| | | | | 10 | A12949 | 4.89E+10 | 1.06E+06 | 1,691,740 | 1,128,437 | 52 | 52 | **0.005** | 53.70 | **0.06** | 1 | 43.00 | **0.00** |
| | | | | 10 | A15721 | 6.44E+10 | 1.04E+06 | 1,309,894 | 907,334 | 32 | 32 | **0.004** | 62.40 | **0.10** | 1 | 40.00 | **0.00** |
| **Bacho Kiro BK-1653** | SP7103 | 67.9 | E9299 | 10 | A11203 | 2.44E+10 | 8.28E+05 | 2,530,593 | 1,381,741 | 11,825 | 11,783 | **0.856** | 55.70 | **2.82** | 1,880 | 55.00 | **1.66** |
| | | | | 10 | A12363 | 2.14E+10 | 1.47E+06 | 549,052 | 300,572 | 2,028 | 2,028 | **0.675** | 55.00 | **1.97** | 318 | 46.10 | **0.95** |
| | | | | 10 | A12562 | 1.63E+10 | 9.64E+05 | 3,625,907 | 2,020,940 | 14,612 | 14,567 | **0.723** | 55.50 | **1.61** | 2,266 | 55.10 | **0.94** |
| | | | | 10 | A12950 | 1.93E+10 | 1.01E+06 | 2,167,430 | 1,216,492 | 8,285 | 8,263 | **0.681** | 55.80 | **1.83** | 1,173 | 54.80 | **0.95** |
| | | | | 10 | A15722 | 3.06E+10 | 1.13E+06 | 1,735,623 | 1,007,674 | 6,486 | 6,456 | **0.644** | 56.10 | **2.82** | 929 | 56.10 | **1.53** |
| **Oase1** | SP2538 | 24.8 | E1406[+] | 5 | A12968 | 3.36E+10 | 8.85E+05 | 1,891,916 | 998,108 | 1,462 | 1,452 | **0.146** | 49.70 | **0.54** | 337 | 48.60 | **0.10** |
| | | 10.3 | E1843[+] | 5 | A12967 | 9.47E+10 | 8.38E+05 | 2,252,369 | 1,289,806 | 668 | 666 | **0.052** | 49.30 | **0.66** | 147 | 46.80 | **0.10** |
| | | 15 | E6735 | 10 | A12384 | 2.11E+09 | 1.34E+06 | 488,744 | 236,662 | 350 | 350 | **0.148** | 48.90 | **0.03** | 90 | 50.00 | **0.01** |
| | | | | 10 | A12590 | 1.58E+09 | 8.57E+05 | 2,915,887 | 1,501,927 | 2,335 | 2,327 | **0.155** | 49.70 | **0.03** | 588 | 46.70 | **0.01** |
| | | | | 10 | A12955 | 2.10E+09 | 1.15E+06 | 2,174,525 | 1,111,248 | 1,411 | 1,406 | **0.127** | 50.00 | **0.03** | 334 | 49.00 | **0.01** |
| | | | | 10 | F9015 | 2.18E+09 | 1.41E+06 | 1,788,088 | 877,011 | 1,359 | 1,359 | **0.155** | 47.40 | **0.03** | 322 | 47.60 | **0.01** |

* As determined using quantitative PCR (qPCR), [+] DNA extracts made in the previous study (*Fu et al.*[8]); mg – milligram, µL – microlitre, bp – base pairs, MQ – mapping quality

**Table S2.2 Frequencies of C-to-T substitutions at terminal positions of the sequence alignments for the libraries generated by shotgun sequencing.** The C-to-T substitution frequencies are determined on the mapped fragments longer than 35 base pairs with mapping quality of at least 25 (MQ≥25) reported in the Table S2.1. 95% binomial confidence intervals (CI) are provided in brackets. C – cytosine, T – thymine.

| Specimen | Library ID | All fragments | | Fragments with C-to-T substitutions at the opposing end | |
|---|---|---|---|---|---|
| | | 5' C→T [95% CI] | 3' C → T [95% CI] | 5' C→T [95% CI] | 3' C → T [95% CI] |
| Bacho Kiro F6-620 | A11197 | 41.8 [40.3-43.3] | 34 [32.5-35.4] | 44.2 [38.8-49.7] | 39.2 [34.0-44.1] |
| | A12357 | 42.0 [38.8-45.2] | 29.7 [26.7-32.8] | 50.0 [36.6-63.4] | 29.8 [21.0-40.2] |
| | A12550 | 41.4 [40.0-42.7] | 32.0 [30.7-33.4] | 42.5 [37.2-47.4] | 36.5 [31.8-41.0] |
| | A12944 | 41.2 [39.7-42.6] | 33.0 [31.6-34.5] | 42.7 [36.5-48.3] | 32.3 [27.6-37.3] |
| | A15716 | 41.5 [39.4-43.6] | 30.0 [28.0-32.0] | 41.8 [33.4-50.7] | 30.2 [23.2-36.9] |
| Bacho Kiro AA7-738 | A11198 | 65.8 [53.3-74.3] | 53.8 [41.6-63.3] | 87.5 [52.9-97.8] | 58.3 [25.4-74.6] |
| | A12358 | 76.9 [53.9-86.3] | 37.5 [18.5-61.4] | 66.7 [20.8-93.9] | 66.7 [20.8-93.9] |
| | A12554 | 50.6 [43.0-58.2] | 46.5 [37.8-54.0] | 38.5 [17.7-64.5] | 35.7 [11.7-54.6] |
| | A12945 | 45.2 [29.1-57.8] | 35.7 [21.0-48.4] | 66.7 [30.0-90.3] | 57.1 [15.8-75.0] |
| | A15717 | 57.8 [43.3-71.0] | 34.7 [21.2-46.6] | 50.0 [9.5-90.5] | 14.3 [2.6-51.3] |
| Bacho Kiro BB7-240 | A11199 | 56.7 [54.3-59.0] | 42.4 [39.9-44.8] | 63.3 [54.3-70.2] | 41.5 [35.1-48.2] |
| | A12359 | 50.0 [44.2-55.8] | 41.3 [35.5-47.3] | 46.2 [28.8-64.5] | 40.0 [24.6-57.7] |
| | A12555 | 55.6 [53.5-57.7] | 38.1 [35.9-40.2] | 52.6 [45.0-59.1] | 38.3 [32.3-44.0] |
| | A12946 | 53.7 [51.2-56.2] | 38.9 [36.2-41.5] | 49.7 [41.6-57.7] | 38.2 [31.5-45.3] |
| | A15718 | 49.1 [45.5-52.4] | 38.7 [34.9-42.3] | 55.7 [42.7-65.4] | 46.4 [35.0-55.9] |
| Bacho Kiro CC7-2289 | A11200 | 43.8 [23.1-66.8] | 33.3 [3.0-56.4] | 100 [20.7-100.0] | 100 [20.7-100.0] |
| | A12360 | NA [N/A] | 0.0 [0.0-65.8] | NA [N/A] | NA [NA] |
| | A12556 | 30.4 [15.6-50.9] | 29.4 [13.3-53.1] | 0.0 [0.0-79.3] | NA [NA] |
| | A12947 | 33.3 [6.3-54.7] | 0.0 [0.0-39.0] | NA [N/A] | 0.0 [0.0-79.3] |
| | A15719 | 75.0 [30.1-95.4] | 0.0 [0.0-49.0] | NA [N/A] | 0.0 [0.0-79.3] |
| Bacho Kiro CC7-335 | A11201 | 58.7 [56.9-60.5] | 45.1 [43.1-47.1] | 56.5 [50.1-62.0] | 42.9 [37.8-48.2] |
| | A12361 | 62.7 [58.1-66.7] | 42.8 [38.1-47.6] | 64.1 [45.9-75.1] | 43.9 [30.2-55.0] |
| | A12557 | 56.3 [54.4-58.1] | 45.6 [43.6-47.7] | 51.4 [44.8-57.2] | 44.8 [39.1-50.5] |
| | A12948 | 56.6 [53.5-59.5] | 45.4 [42.0-48.6] | 56.9 [46.6-64.9] | 49.6 [41.0-58.2] |
| | A15720 | 58.8 [56.3-61.2] | 43.9 [41.0-46.6] | 58.7 [50.2-65.5] | 44.8 [38.1-51.7] |
| Bacho Kiro F6-597 | A11202 | 26.3 [8.5-43.3] | 5.3 [0.0-16.8] | NA [N/A] | NA [NA] |
| | A12362 | 0.0 [0.0-56.2] | 0.0 [0.0-65.8] | NA [N/A] | NA [NA] |
| | A12558 | 0.0 [0.0-20.4] | 9.1 [0.8-21.8] | NA [N/A] | NA [NA] |
| | A12949 | 0.0 [0.0-27.8] | 6.7 [1.2-29.8] | NA [N/A] | NA [NA] |
| | A15721 | 0.0 [0.0-35.4] | 16.7 [3.0-56.4] | NA [N/A] | NA [NA] |
| Bacho Kiro BK-1653 | A11203 | 26.8 [25.2-28.5] | 18.9 [17.3-20.5] | 30.1 [22.1-39.5] | 22.1 [15.4-28.9] |
| | A12363 | 25.8 [22.1-30.0] | 16.8 [13.4-20.9] | 44.4 [20.3-61.4] | 36.4 [19.7-57.0] |
| | A12562 | 24.8 [23.3-26.2] | 18.4 [17.0-19.9] | 29.1 [21.2-36.7] | 25.0 [18.7-32.5] |
| | A12950 | 25.0 [23.1-27.0] | 15.6 [13.8-17.4] | 29.4 [19.9-41.1] | 22.0 [14.7-31.5] |
| | A15722 | 27.1 [24.9-29.4] | 13.7 [11.9-15.8] | 48.1 [27.6-62.7] | 14.3 [8.5-22.9] |
| Oase1 | A12968* | 38.5 [33.1-43.6] | 37.6 [31.1-43.6] | 50.0 [28.0-72.0] | 38.1 [20.8-59.1] |
| | A12967* | 39.1 [31.8-46.9] | 30.4 [22.8-39.4] | 80.0 [37.6-96.4] | 40.0 [16.8-68.7] |
| | A12384 | 33.8 [24.3-44.6] | 35.5 [23.3-46.3] | 33.3 [0.0-56.2] | 33.3 [0.0-56.2] |
| | A12590 | 36.7 [32.7-40.5] | 32.7 [28.3-37.0] | 37.2 [24.4-52.1] | 42.1 [25.6-55.3] |
| | A12955 | 35.6 [30.5-41.0] | 31.7 [26.3-37.7] | 35.3 [13.3-53.1] | 26.1 [9.7-41.9] |
| | F9015 | 34.2 [29.2-39.5] | 36.9 [30.8-42.6] | 29.2 [14.9-49.2] | 41.2 [21.6-64.0] |

* New libraries prepared from two extracts used in a previous study (Fu et al.[8])

**Table S2.3 Summary statistics of the Bacho Kiro and *Oase1* DNA libraries captured with the SNP Panel 1 or "390k"[15] array.** The number of SNPs on target are determined using the unique fragments longer than 35 base pairs (L≥35 bp) with mapping quality of at least 25 (MQ≥25) that overlap the SNPs on the "390k" array[15].

| | All fragments | | | | | | | | Fragments with terminal C-to-T substitutions | | |
|---|---|---|---|---|---|---|---|---|---|---|---|
| **Specimen** | **Library ID** | **Number of sequenced fragments** | **Number of fragments ≥35 bp** | **Number of mapped fragments ≥35bp, MQ≥25** | **Number of mapped fragments on target ≥35bp, MQ≥25** | **Number of unique fragments on target ≥35bp, MQ≥25** | **Number of SNPs on target** | **% of SNPs on target** | **Number of deaminated fragments on target ≥35bp, MQ≥25** | **Number of SNPs on target** | **% of SNPs on target** |
| **Bacho Kiro F6-620** | A11197 | 11,899,581 | 8,645,070 | 3,467,069 | 1,561,833 | 1,001,291 | 316,864 | 80.47 | 259,815 | 172,587 | 43.83 |
| | A12357 | 10,869,174 | 8,052,475 | 2,598,741 | 1,317,844 | 880,398 | 300,283 | 76.25 | 213,441 | 149,237 | 37.90 |
| | A12550 | 8,917,113 | 6,472,270 | 1,909,121 | 1,050,064 | 701,836 | 274,734 | 69.77 | 166,987 | 124,241 | 31.55 |
| | A12944 | 9,586,753 | 7,103,310 | 2,090,003 | 1,024,769 | 677,230 | 272,094 | 69.10 | 159,465 | 120,418 | 30.58 |
| | A15716 | 1,671,876 | 1,334,551 | 599,155 | 463,053 | 349,380 | 191,006 | 48.50 | 80,224 | 68,100 | 17.29 |
| | merged | 42,944,497 | 31,607,676 | 10,664,089 | 5,417,563 | 3,610,135 | 372,571 | 94.61 | 879,932 | 294,287 | 74.73 |
| **Bacho Kiro AA7-738** | A11198 | 12,145,465 | 8,700,739 | 175,278 | 23,854 | 8,343 | 7,806 | 1.98 | 3,178 | 3,104 | 0.79 |
| | A12358 | 1,410,251 | 1,047,605 | 38,811 | 12,811 | 7,112 | 6,793 | 1.73 | 1,895 | 1,885 | 0.48 |
| | A12554 | 1,533,400 | 1,104,923 | 37,071 | 10,079 | 5,072 | 4,840 | 1.23 | 1,813 | 1,798 | 0.46 |
| | A12945 | 1,635,213 | 1,227,385 | 31,282 | 10,294 | 5,381 | 5,128 | 1.30 | 2,000 | 1,980 | 0.50 |
| | A15717 | 27,180,764 | 10,263,094 | 297,329 | 11,099 | 3,526 | 2,910 | 0.74 | 1,089 | 1,022 | 0.26 |
| | merged | 43,905,093 | 22,343,746 | 579,771 | 68,137 | 29,434 | 26,299 | 6.68 | 9,975 | 9,629 | 2.45 |
| **Bacho Kiro BB7-240** | A11199 | 13,252,473 | 9,002,008 | 2,123,764 | 487,691 | 235,156 | 163,518 | 41.52 | 84,770 | 73,710 | 18.72 |
| | A12359 | 12,593,847 | 8,262,403 | 1,227,745 | 374,230 | 198,565 | 143,427 | 36.42 | 69,051 | 61,084 | 15.51 |
| | A12555 | 10,688,993 | 6,831,509 | 807,933 | 299,881 | 158,830 | 120,932 | 30.71 | 54,468 | 49,494 | 12.57 |
| | A12946 | 10,720,721 | 7,377,939 | 946,511 | 329,501 | 182,060 | 132,737 | 33.71 | 60,886 | 54,551 | 13.85 |
| | A15718 | 1,423,176 | 1,057,610 | 322,999 | 174,753 | 118,568 | 93,947 | 23.86 | 38,486 | 35,757 | 9.08 |
| | merged | 48,679,210 | 32,531,469 | 5,428,952 | 1,666,056 | 893,179 | 311,587 | 79.13 | 307,661 | 191,684 | 48.68 |
| **Bacho Kiro CC7-2289** | A11200 | 11,083,652 | 7,875,798 | 26,040 | 7,442 | 2,427 | 2,375 | 0.60 | 583 | 586 | 0.15 |
| | A12360 | 1,698,092 | 1,187,404 | 8,578 | 4,113 | 2,267 | 2,258 | 0.57 | 350 | 357 | 0.09 |
| | A12556 | 1,440,718 | 1,007,689 | 5,788 | 2,839 | 1,521 | 1,504 | 0.38 | 339 | 337 | 0.09 |
| | A12947 | 1,280,691 | 939,834 | 3,974 | 2,382 | 1,268 | 1,256 | 0.32 | 256 | 258 | 0.07 |
| | A15719 | 1,496,778 | 1,115,292 | 7,269 | 3,034 | 1,614 | 1,405 | 0.36 | 300 | 284 | 0.07 |
| | merged | 16,999,931 | 12,126,017 | 51,649 | 19,810 | 9,097 | 8,659 | 2.20 | 1,828 | 1,816 | 0.46 |
| **Bacho Kiro CC7-335** | A11201 | 11,973,014 | 8,804,658 | 2,337,293 | 418,725 | 165,324 | 126,717 | 32.18 | 63,194 | 56,815 | 14.43 |
| | A12361 | 11,406,591 | 8,205,044 | 1,377,993 | 360,202 | 163,136 | 123,327 | 31.32 | 59,292 | 53,333 | 13.54 |
| | A12557 | 10,742,241 | 7,714,075 | 1,012,327 | 325,196 | 147,895 | 113,536 | 28.83 | 55,405 | 50,031 | 12.71 |
| | A12948 | 10,627,567 | 7,762,120 | 1,116,051 | 290,639 | 131,242 | 102,830 | 26.11 | 48,974 | 44,518 | 11.31 |
| | A15720 | 1,584,771 | 1,275,920 | 412,202 | 163,168 | 99,826 | 81,481 | 20.69 | 36,322 | 33,796 | 8.58 |
| | merged | 46,334,184 | 33,761,817 | 6,255,866 | 1,557,930 | 707,423 | 290,832 | 73.85 | 263,187 | 174,782 | 44.38 |
| **Bacho Kiro BK-1653** | A11203 | 11,703,301 | 8,659,849 | 2,859,809 | 1,100,323 | 599,073 | 273,924 | 69.56 | 96,817 | 83,025 | 21.08 |
| | A12363 | 11,951,100 | 8,517,115 | 1,909,417 | 898,108 | 549,819 | 254,152 | 64.54 | 84,654 | 72,863 | 18.50 |
| | A12562 | 10,428,783 | 7,311,600 | 1,643,754 | 669,793 | 398,421 | 218,118 | 55.39 | 60,080 | 54,159 | 13.75 |
| | A12950 | 10,423,957 | 7,377,054 | 1,603,265 | 704,765 | 423,243 | 224,583 | 57.03 | 62,556 | 56,132 | 14.25 |
| | A15722 | 24,068,105 | 10,618,989 | 5,828,548 | 953,264 | 539,986 | 272,135 | 69.11 | 77,161 | 69,213 | 17.58 |
| | merged | 68,575,246 | 42,484,607 | 13,844,793 | 4,326,253 | 2,510,542 | 393,788 | 93.41 | 381,268 | 215,802 | 54.8 |
| **Oase1** | A12968* | 9,450,683 | 6,293,578 | 316,504 | 225,313 | 91,716 | 76,988 | 19.55 | 19,613 | 18,981 | 4.82 |
| | A12967* | 8,702,207 | 5,908,403 | 147,181 | 106,039 | 41,967 | 39,035 | 9.91 | 7,468 | 7,456 | 1.89 |
| | A12384 | 9,523,290 | 6,124,858 | 355,622 | 259,755 | 24,872 | 23,254 | 5.91 | 5,515 | 5,458 | 1.39 |
| | A12590 | 10,954,958 | 7,076,935 | 380,208 | 272,863 | 22,833 | 21,538 | 5.47 | 5,651 | 5,584 | 1.42 |
| | A12955 | 9,542,825 | 6,218,130 | 282,570 | 199,618 | 22,392 | 21,166 | 5.37 | 5,417 | 5,390 | 1.37 |
| | F9015 | 10,928,126 | 6,664,522 | 364,795 | 256,619 | 19,621 | 18,620 | 4.73 | 4,892 | 4,845 | 1.23 |
| | merged | 59,102,089 | 38,286,426 | 1,846,880 | 1,320,207 | 223,401 | 155,508 | 39.49 | 48,556 | 44,591 | 11.32 |

***** New libraries prepared from two extracts used in a previous study (*Fu et al.*[8])

**Table S2.4 Summary statistics of the Bacho Kiro and *Oase1* DNA libraries captured with the SNP Panel 2 or "840k"[8] array.** The number of SNPs on target are determined using the unique fragments longer than 35 base pairs with mapping quality of at least 25 (MQ≥25) that overlap the SNPs on the "840k" array[8].

| | | **All fragments** | | | | | | | **Fragments with terminal C-to-T substitutions** | | |
|---|---|---|---|---|---|---|---|---|---|---|---|
| **Specimen** | **Library ID** | **Number of sequenced fragments** | **Number of fragments ≥35 bp** | **Number of mapped fragments ≥35bp, MQ≥25** | **Number of mapped fragments on target ≥35bp, MQ≥25** | **Number of unique fragments on target ≥35bp, MQ≥25** | **Number of SNPs on target** | **% of SNPs on target** | **Number of deaminated fragments on target ≥35bp, MQ≥25** | **Number of SNPs on target** | **% of SNPs on target** |
| **Bacho Kiro F6-620** | A11197 | 22,926,356 | 17,251,602 | 6,317,820 | 4,319,293 | 1,741,983 | 616,855 | 73.21 | 453,942 | 315,619 | 37.46 |
| | A12357 | 18,328,399 | 13,704,688 | 5,215,324 | 3,667,130 | 1,698,935 | 595,206 | 70.64 | 416,479 | 291,769 | 34.63 |
| | A12550 | 9,342,745 | 6,882,903 | 2,425,491 | 1,693,850 | 971,643 | 458,798 | 54.45 | 232,921 | 185,707 | 22.04 |
| | A12944 | 9,442,994 | 7,140,283 | 2,427,405 | 1,663,189 | 908,195 | 441,659 | 52.41 | 215,546 | 173,808 | 20.63 |
| | A15716 | 8,821,338 | 7,040,301 | 2,635,727 | 1,826,406 | 1,029,637 | 480,163 | 56.98 | 241,148 | 192,930 | 22.90 |
| | merged | 68,861,832 | 52,019,777 | 19,021,767 | 13,169,868 | 6,350,393 | 757,051 | 89.84 | 1,560,036 | 558,143 | 66.24 |
| **Bacho Kiro AA7-738** | A11198 | 23,445,403 | 17,152,839 | 310,849 | 186,370 | 15,639 | 14,804 | 1.76 | 5,889 | 5,835 | 0.69 |
| | A12358 | 7,757,929 | 5,754,460 | 130,802 | 83,632 | 15,563 | 14,912 | 1.77 | 4,146 | 4,134 | 0.49 |
| | A12554 | 11,338,006 | 8,280,216 | 126,265 | 74,600 | 11,717 | 11,193 | 1.33 | 4,257 | 4,235 | 0.50 |
| | A12945 | 11,004,643 | 8,279,751 | 117,146 | 69,354 | 12,179 | 11,563 | 1.37 | 4,455 | 4,413 | 0.52 |
| | A15717 | 9,125,030 | 7,040,076 | 105,180 | 65,037 | 8,940 | 8,561 | 1.02 | 3,182 | 3,162 | 0.38 |
| | merged | 62,671,011 | 46,507,342 | 790,242 | 478,993 | 64,038 | 58,037 | 6.89 | 21,929 | 21,376 | 2.54 |
| **Bacho Kiro BB7-240** | A11199 | 23,835,075 | 16,599,615 | 3,378,384 | 2,167,599 | 412,089 | 298,929 | 35.48 | 148,602 | 131,835 | 15.65 |
| | A12359 | 18,972,864 | 12,867,006 | 2,658,602 | 1,788,537 | 392,301 | 286,854 | 34.04 | 136,778 | 122,184 | 14.50 |
| | A12555 | 9,876,045 | 6,388,464 | 1,089,368 | 687,569 | 248,874 | 198,827 | 23.60 | 85,335 | 79,414 | 9.42 |
| | A12946 | 10,762,884 | 7,482,516 | 1,439,478 | 949,797 | 300,368 | 228,456 | 27.11 | 101,665 | 92,842 | 11.02 |
| | A15718 | 8,597,668 | 6,361,984 | 1,249,878 | 818,538 | 296,802 | 228,514 | 27.12 | 98,833 | 90,938 | 10.79 |
| | merged | 72,044,536 | 49,699,585 | 9,815,710 | 6,412,040 | 1,650,434 | 610,752 | 72.48 | 571,213 | 362,810 | 43.06 |
| **Bacho Kiro CC7-2289** | A11200 | 20,095,361 | 14,382,395 | 49,188 | 21,615 | 4,494 | 4,369 | 0.52 | 1,105 | 1,107 | 0.13 |
| | A12360 | 9,231,418 | 6,549,521 | 31,000 | 16,370 | 4,685 | 4,673 | 0.55 | 707 | 718 | 0.09 |
| | A12556 | 10,052,171 | 7,227,057 | 25,443 | 12,110 | 3,452 | 3,387 | 0.40 | 659 | 657 | 0.08 |
| | A12947 | 8,705,372 | 6,410,889 | 20,132 | 9,369 | 3,090 | 3,074 | 0.36 | 643 | 654 | 0.08 |
| | A15719 | 10,531,118 | 7,990,473 | 25,153 | 11,751 | 3,000 | 2,938 | 0.35 | 602 | 612 | 0.07 |
| | merged | 58,615,440 | 42,560,335 | 150,916 | 71,215 | 18,721 | 18,151 | 2.15 | 3,716 | 3,731 | 0.44 |
| **Bacho Kiro CC7-335** | A11201 | 21,338,266 | 15,910,234 | 3,627,638 | 2,258,383 | 301,906 | 236,938 | 28.12 | 114,807 | 104,619 | 12.42 |
| | A12361 | 17,989,048 | 13,218,581 | 2,762,393 | 1,794,352 | 317,663 | 242,641 | 28.80 | 116,021 | 105,046 | 12.47 |
| | A12557 | 9,943,887 | 7,176,315 | 1,143,312 | 711,224 | 217,233 | 176,557 | 20.95 | 80,635 | 74,945 | 8.89 |
| | A12948 | 10,700,557 | 7,881,140 | 1,482,421 | 951,441 | 218,273 | 177,072 | 21.01 | 81,473 | 75,681 | 8.98 |
| | A15720 | 8,822,517 | 7,045,295 | 1,374,443 | 884,459 | 235,591 | 190,005 | 22.55 | 86,348 | 80,048 | 9.50 |
| | merged | 68,794,275 | 51,231,565 | 10,390,207 | 6,599,859 | 1,290,666 | 561,737 | 66.66 | 479,284 | 326,926 | 38.80 |
| **Bacho Kiro BK-1653** | A11203 | 19,907,962 | 14,792,961 | 4,541,063 | 2,852,991 | 980,828 | 494,311 | 58.66 | 159,264 | 140,173 | 16.64 |
| | A12363 | 18,577,919 | 13,793,835 | 3,967,259 | 2,598,268 | 1,083,564 | 498,360 | 59.14 | 168,722 | 145,511 | 17.27 |
| | A12562 | 10,497,458 | 7,488,565 | 2,071,856 | 1,351,877 | 641,692 | 370,131 | 43.93 | 98,685 | 90,063 | 10.69 |
| | A12950 | 10,134,905 | 7,228,788 | 2,022,122 | 1,333,879 | 573,808 | 347,593 | 41.25 | 86,127 | 79,755 | 9.47 |
| | A15722 | 7,626,731 | 5,851,070 | 1,679,486 | 1,093,476 | 560,128 | 343,387 | 40.75 | 81,599 | 76,010 | 9.02 |
| | merged | 66,744,975 | 49,155,219 | 14,281,786 | 9,230,491 | 3,840,020 | 694,592 | 82.43 | 594,397 | 356,855 | 43.35 |
| **Oase1** | A12968* | 8,859,546 | 5,932,955 | 567,144 | 353,112 | 138,495 | 121,866 | 14.46 | 29,657 | 29,292 | 3.48 |
| | A12967* | 8,488,875 | 5,738,835 | 253,428 | 157,471 | 70,717 | 66,885 | 7.94 | 13,059 | 13,227 | 1.57 |
| | A12384 | 8,056,658 | 5,150,984 | 593,089 | 393,101 | 45,129 | 42,925 | 5.09 | 10,051 | 10,074 | 1.20 |
| | A12590 | 10,858,435 | 7,137,677 | 667,254 | 426,051 | 43,596 | 41,474 | 4.92 | 10,750 | 10,718 | 1.27 |
| | A12955 | 9,605,636 | 6,282,106 | 387,272 | 236,849 | 37,587 | 36,004 | 4.27 | 9,086 | 9,113 | 1.08 |
| | F9015 | 10,876,167 | 6,788,033 | 563,602 | 353,964 | 32,728 | 31,673 | 3.76 | 8,106 | 8,145 | 0.97 |
| | merged | 56,745,317 | 37,030,590 | 3,031,789 | 1,920,548 | 368,252 | 270,601 | 32.11 | 80,709 | 75,569 | 8.97 |

***** New libraries prepared from two extracts used in a previous study (*Fu et al.*[8])

**Table S2.5 Summary statistics of the Bacho Kiro and *Oase1* DNA libraries captured with the SNP Panel 3 or "1000k"[8] array.** The number of SNPs on target are determined using the unique fragments longer than 35 base pairs (L≥35 bp) with mapping quality of at least 25 (MQ≥25) that overlap the SNPs on the "1000k" array[8].

| | | All fragments | | | | | | | Fragments with terminal C-to-T substitutions | | |
|---|---|---|---|---|---|---|---|---|---|---|---|
| **Specimen** | **Library ID** | **Number of sequenced fragments** | **Number of fragments ≥35 bp** | **Number of mapped fragments ≥35bp, MQ≥25** | **Number of mapped fragments on target ≥35bp, MQ≥25** | **Number of unique fragments on target ≥35bp, MQ≥25** | **Number of SNPs on target** | **% of SNPs on target** | **Number of deaminated fragments on target ≥35bp, MQ≥25** | **Number of SNPs on target** | **% of SNPs on target** |
| **Bacho Kiro F6-620** | A11197 | 21,991,234 | 16,842,418 | 6,151,477 | 4,155,525 | 1,474,347 | 608,886 | 61.02 | 369,294 | 279,759 | 28.04 |
| | A12357 | 17,215,328 | 13,186,365 | 4,962,070 | 3,537,600 | 1,411,543 | 583,556 | 58.49 | 332,082 | 255,889 | 25.65 |
| | A12550 | 11,263,593 | 8,296,609 | 2,744,780 | 1,884,129 | 914,395 | 464,625 | 46.57 | 211,720 | 177,776 | 17.82 |
| | A12944 | 8,563,214 | 6,441,281 | 2,032,905 | 1,405,287 | 722,521 | 402,943 | 40.38 | 164,717 | 143,526 | 14.38 |
| | A15716 | 10,575,449 | 8,523,083 | 3,338,770 | 2,401,967 | 790,645 | 447,152 | 44.81 | 177,656 | 156,368 | 15.67 |
| | merged | 69,608,818 | 53,289,756 | 19,230,002 | 13,384,508 | 5,313,451 | 808,722 | 81.05 | 1,255,469 | 537,525 | 53.87 |
| **Bacho Kiro AA7-738** | A11198 | 23,090,198 | 17,199,659 | 253,576 | 138,541 | 12,213 | 11,956 | 1.20 | 4,476 | 4,549 | 0.46 |
| | A12358 | 7,508,510 | 5,682,372 | 119,420 | 77,957 | 11,361 | 11,251 | 1.13 | 2,861 | 2,894 | 0.29 |
| | A12554 | 9,771,730 | 7,268,861 | 131,135 | 75,322 | 7,169 | 7,141 | 0.72 | 2,508 | 2,584 | 0.26 |
| | A12945 | 9,416,186 | 7,225,669 | 106,165 | 61,279 | 7,611 | 7,573 | 0.76 | 2,767 | 2,833 | 0.28 |
| | A15717 | 11,764,615 | 4,719,341 | 67,686 | 45,702 | 3,006 | 3,069 | 0.31 | 955 | 999 | 0.10 |
| | merged | 61,551,239 | 42,095,902 | 677,982 | 398,801 | 41,360 | 39,660 | 3.97 | 13,567 | 13,677 | 1.37 |
| **Bacho Kiro BB7-240** | A11199 | 25,195,252 | 17,475,054 | 3,123,776 | 1,971,708 | 327,492 | 258,903 | 25.95 | 113,777 | 106,242 | 10.65 |
| | A12359 | 20,696,734 | 14,122,968 | 2,534,285 | 1,679,644 | 296,500 | 239,061 | 23.96 | 98,633 | 93,280 | 9.35 |
| | A12555 | 10,138,039 | 6,494,073 | 878,841 | 536,288 | 181,137 | 157,496 | 15.78 | 59,747 | 58,737 | 5.89 |
| | A12946 | 10,523,868 | 7,211,960 | 1,060,486 | 681,607 | 215,549 | 180,387 | 18.08 | 70,316 | 67,916 | 6.81 |
| | A15718 | 8,836,672 | 6,540,204 | 1,393,464 | 952,349 | 188,610 | 163,965 | 16.43 | 60,393 | 59,249 | 5.94 |
| | merged | 75,390,565 | 51,844,259 | 8,990,852 | 5,821,596 | 1,209,288 | 565,852 | 56.71 | 402,866 | 294,403 | 29.51 |
| **Bacho Kiro CC7-2289** | A11200 | 21,474,542 | 15,685,689 | 57,751 | 18,729 | 3,069 | 3,131 | 0.31 | 724 | 755 | 0.08 |
| | A12360 | 7,803,992 | 5,694,060 | 31,163 | 14,834 | 2,034 | 2,123 | 0.21 | 270 | 285 | 0.03 |
| | A12556 | 8,899,916 | 6,493,949 | 27,949 | 11,355 | 1,677 | 1,735 | 0.17 | 353 | 372 | 0.04 |
| | A12947 | 8,181,325 | 6,124,722 | 22,437 | 8,110 | 1,487 | 1,561 | 0.16 | 273 | 293 | 0.03 |
| | A15719 | 8,584,651 | 6,634,901 | 28,248 | 11,265 | 1,478 | 1,539 | 0.15 | 254 | 261 | 0.03 |
| | merged | 54,944,426 | 40,633,321 | 167,548 | 64,293 | 9,745 | 10,003 | 1.00 | 1,874 | 1,965 | 0.20 |
| **Bacho Kiro CC7-335** | A11201 | 21,990,555 | 16,458,472 | 3,600,338 | 2,220,876 | 262,444 | 217,477 | 21.80 | 96,651 | 91,365 | 9.16 |
| | A12361 | 20,500,220 | 15,249,848 | 3,227,366 | 2,105,982 | 267,013 | 217,295 | 21.78 | 94,622 | 89,027 | 8.92 |
| | A12557 | 9,823,290 | 7,102,874 | 1,015,008 | 595,845 | 178,230 | 153,326 | 15.37 | 64,825 | 62,753 | 6.29 |
| | A12948 | 11,125,768 | 8,140,989 | 1,347,104 | 845,655 | 176,385 | 151,287 | 15.16 | 64,004 | 61,709 | 6.18 |
| | A15720 | 9,024,906 | 7,269,772 | 1,717,757 | 1,167,209 | 178,891 | 155,004 | 15.53 | 63,632 | 61,835 | 6.20 |
| | merged | 72,464,739 | 54,221,955 | 10,907,573 | 6,935,567 | 1,062,963 | 536,521 | 53.77 | 383,734 | 286,514 | 28.72 |
| **Bacho Kiro BK-1653** | A11203 | 22,558,998 | 16,730,866 | 5,022,091 | 3,206,900 | 837,168 | 479,689 | 48.08 | 130,533 | 121,256 | 12.15 |
| | A12363 | 19,237,335 | 14,309,074 | 4,072,250 | 2,730,583 | 817,869 | 457,572 | 45.86 | 122,614 | 113,883 | 11.41 |
| | A12562 | 11,410,950 | 8,078,126 | 1,918,594 | 1,225,111 | 501,710 | 332,243 | 33.30 | 74,196 | 71,885 | 7.20 |
| | A12950 | 9,658,498 | 6,839,385 | 1,730,548 | 1,146,618 | 444,284 | 305,733 | 30.64 | 64,164 | 62,821 | 6.30 |
| | A15722 | 9,521,514 | 4,040,947 | 1,786,417 | 1,328,323 | 310,326 | 248,582 | 24.91 | 42,844 | 43,627 | 4.37 |
| | merged | 72,387,295 | 49,998,398 | 14,529,900 | 9,637,535 | 2,911,357 | 726,130 | 72.77 | 434,351 | 305,619 | 30.63 |
| **Oase1** | A12968* | 9,913,082 | 6,526,052 | 460,141 | 283,631 | 97,727 | 91,714 | 9.19 | 19,696 | 20,261 | 2.03 |
| | A12967* | 9,381,272 | 6,330,765 | 210,442 | 127,461 | 44,136 | 44,466 | 4.46 | 7,448 | 7,819 | 0.78 |
| | A12384 | 9,326,741 | 6,042,399 | 461,129 | 293,613 | 38,011 | 37,284 | 3.74 | 7,837 | 8,053 | 0.81 |
| | A12590 | 10,425,950 | 6,753,910 | 482,672 | 300,968 | 33,306 | 32,737 | 3.28 | 7,658 | 7,829 | 0.78 |
| | A12955 | 10,204,276 | 6,685,185 | 358,928 | 211,600 | 31,440 | 31,056 | 3.11 | 7,020 | 7,175 | 0.72 |
| | F9015 | 10,097,198 | 6,248,272 | 373,923 | 217,345 | 28,323 | 28,104 | 2.82 | 6,761 | 6,940 | 0.70 |
| | merged | 59,348,519 | 38,586,583 | 2,347,235 | 1,434,618 | 272,943 | 221,020 | 22.15 | 56,420 | 55,309 | 5.54 |

***** New libraries prepared from two extracts used in a previous study (*Fu et al.*[8])

**Table S2.6 Summary statistics of the Bacho Kiro and *Oase1* DNA libraries captured with the SNP Panel 4 or "Archaic admixture"[8] array.** The number of SNPs on target are determined using the unique fragments longer than 35 base pairs (L≥35 bp) with mapping quality of at least 25 (MQ≥25) that overlap the SNPs on the "Archaic admixture" array[8].

| | All fragments | | | | | | | | Fragments with terminal C-to-T substitutions | | |
|---|---|---|---|---|---|---|---|---|---|---|---|
| **Specimen** | **Library ID** | **Number of sequenced fragments** | **Number of fragments ≥35 bp** | **Number of mapped fragments ≥35bp, MQ≥25** | **Number of mapped fragments on target ≥35bp, MQ≥25** | **Number of unique fragments on target ≥35bp, MQ≥25** | **Number of SNPs on target** | **% of SNPs on target** | **Number of deaminated fragments on target ≥35bp, MQ≥25** | **Number of SNPs on target** | **% of SNPs on target** |
| **Bacho Kiro F6-620** | A11197 | 31,930,399 | 23,542,084 | 6,780,310 | 4,055,151 | 1,886,935 | 953,809 | 54.52 | 484,643 | 421,808 | 24.11 |
| | A12357 | 54,684,610 | 39,738,237 | 11,467,222 | 7,539,176 | 2,528,304 | 1,077,024 | 61.57 | 613,646 | 499,442 | 28.55 |
| | A12550 | 9,906,107 | 7,226,662 | 2,157,336 | 1,415,846 | 883,285 | 597,714 | 34.17 | 210,651 | 213,726 | 12.22 |
| | A12944 | 47,491,464 | 35,193,886 | 9,083,870 | 5,808,366 | 1,967,918 | 953,378 | 54.50 | 465,116 | 403,173 | 23.05 |
| | A15716 | 9,770,314 | 7,684,551 | 2,374,589 | 1,639,090 | 995,819 | 657,661 | 37.59 | 230,700 | 233,098 | 13.32 |
| | merged | 153,782,894 | 113,385,420 | 31,863,327 | 20,457,629 | 8,262,261 | 1,405,078 | 80.32 | 2,004,756 | 927,570 | 53.02 |
| **Bacho Kiro AA7-738** | A11198 | 41,109,419 | 30,072,270 | 421,964 | 163,706 | 22,805 | 21,351 | 1.22 | 7,658 | 8,277 | 0.47 |
| | A12358 | 7,465,537 | 5,674,453 | 82,595 | 41,884 | 15,442 | 15,803 | 0.90 | 3,618 | 4,161 | 0.24 |
| | A12554 | 9,618,811 | 7,113,815 | 98,615 | 46,775 | 13,118 | 12,786 | 0.73 | 4,311 | 4,807 | 0.27 |
| | A12945 | 9,670,106 | 7,379,698 | 82,321 | 39,122 | 12,981 | 12,482 | 0.71 | 4,249 | 4,769 | 0.27 |
| | A15717 | 6,610,421 | 5,163,955 | 56,111 | 24,879 | 8,616 | 8,319 | 0.48 | 2,674 | 2,969 | 0.17 |
| | merged | 74,474,294 | 55,404,191 | 741,606 | 316,366 | 72,962 | 67,495 | 3.86 | 22,510 | 24,423 | 1.40 |
| **Bacho Kiro BB7-240** | A11199 | 41,842,444 | 27,505,936 | 3,002,098 | 1,565,980 | 456,423 | 399,472 | 22.83 | 162,041 | 166,099 | 9.49 |
| | A12359 | 53,142,780 | 34,495,298 | 3,800,991 | 2,200,316 | 513,204 | 438,452 | 25.06 | 175,606 | 177,838 | 10.17 |
| | A12555 | 9,076,243 | 5,626,312 | 599,482 | 327,430 | 180,668 | 184,926 | 10.57 | 61,690 | 69,813 | 3.99 |
| | A12946 | 45,490,577 | 30,166,823 | 3,125,838 | 1,739,697 | 443,575 | 385,636 | 22.04 | 148,345 | 153,108 | 8.75 |
| | A15718 | 9,772,037 | 6,950,850 | 849,105 | 525,769 | 258,325 | 251,177 | 14.36 | 85,290 | 94,051 | 5.38 |
| | merged | 159,324,081 | 104,745,219 | 11,377,514 | 6,359,192 | 1,852,195 | 947,950 | 54.19 | 632,972 | 500,142 | 28.59 |
| **Bacho Kiro CC7-2289** | A11200 | 38,931,962 | 27,905,543 | 189,194 | 35,915 | 8,368 | 5,584 | 0.32 | 1,302 | 1,317 | 0.08 |
| | A12360 | 11,622,682 | 8,339,022 | 54,265 | 15,711 | 6,541 | 5,653 | 0.32 | 791 | 879 | 0.05 |
| | A12556 | 9,726,180 | 7,081,632 | 40,292 | 11,277 | 5,034 | 3,844 | 0.22 | 782 | 837 | 0.05 |
| | A12947 | 8,434,644 | 6,293,372 | 32,732 | 7,484 | 4,042 | 3,055 | 0.17 | 598 | 648 | 0.04 |
| | A15719 | 8,928,831 | 6,818,981 | 35,113 | 8,607 | 4,080 | 3,042 | 0.17 | 575 | 670 | 0.04 |
| | merged | 77,644,299 | 56,438,550 | 351,596 | 78,994 | 28,065 | 19,703 | 1.13 | 4,048 | 3,959 | 0.27 |
| **Bacho Kiro CC7-335** | A11201 | 40,556,360 | 28,939,042 | 4,174,229 | 2,149,600 | 404,037 | 359,352 | 20.54 | 151,372 | 154,057 | 8.81 |
| | A12361 | 53,641,009 | 38,346,940 | 4,946,301 | 2,861,939 | 488,498 | 415,698 | 23.76 | 176,473 | 176,345 | 10.08 |
| | A12557 | 8,965,066 | 6,395,492 | 716,845 | 383,083 | 183,978 | 183,343 | 10.48 | 67,784 | 74,719 | 4.27 |
| | A12948 | 44,912,691 | 32,003,257 | 3,821,315 | 2,174,713 | 352,918 | 315,426 | 18.03 | 129,552 | 132,902 | 7.60 |
| | A15720 | 8,640,345 | 6,764,261 | 911,958 | 564,798 | 221,466 | 215,586 | 12.32 | 80,996 | 87,829 | 5.02 |
| | merged | 156,715,471 | 112,448,992 | 14,570,648 | 8,134,133 | 1,650,897 | 907,904 | 51.90 | 606,177 | 488,615 | 27.93 |
| **Bacho Kiro BK-1653** | A11203 | 48,320,999 | 33,985,611 | 7,177,575 | 3,650,622 | 1,258,608 | 802,844 | 45.89 | 200,416 | 205,896 | 11.77 |
| | A12363 | 61,016,010 | 43,460,571 | 8,599,758 | 5,124,135 | 1,668,317 | 911,485 | 52.10 | 255,301 | 252,830 | 14.45 |
| | A12562 | 9,827,836 | 6,729,266 | 1,292,115 | 751,792 | 453,554 | 379,996 | 21.72 | 69,030 | 79,454 | 4.54 |
| | A12950 | 34,594,832 | 23,503,008 | 4,963,426 | 3,109,725 | 864,009 | 615,238 | 35.17 | 127,311 | 136,555 | 7.81 |
| | A15722 | 5,408,560 | 4,014,769 | 833,423 | 525,783 | 346,867 | 313,283 | 17.91 | 50,223 | 59,618 | 3.41 |
| | merged | 159,168,237 | 111,693,225 | 22,866,297 | 13,162,057 | 4,591,355 | 1,257,365 | 71.87 | 702,281 | 533,274 | 30.48 |
| **Oase1** | A12968* | 8,417,896 | 5,419,859 | 327,785 | 180,974 | 92,404 | 100,510 | 5.75 | 19,096 | 22,835 | 1.31 |
| | A12967* | 8,261,454 | 5,467,243 | 160,606 | 76,990 | 48,098 | 54,527 | 3.12 | 8,238 | 10,398 | 0.59 |
| | A12384 | 7,386,323 | 4,672,927 | 306,814 | 176,572 | 46,342 | 48,541 | 2.77 | 9,523 | 10,662 | 0.61 |
| | A12590 | 9,854,422 | 6,351,106 | 380,628 | 198,712 | 46,847 | 48,647 | 2.78 | 10,847 | 11,962 | 0.68 |
| | A12955 | 9,203,696 | 6,067,413 | 266,099 | 127,307 | 37,659 | 39,768 | 2.27 | 8,544 | 9,632 | 0.55 |
| | F9015 | 9,195,952 | 5,789,404 | 277,681 | 130,355 | 35,287 | 37,162 | 2.12 | 8,199 | 9,199 | 0.53 |
| | merged | 52,319,743 | 33,767,952 | 1,719,613 | 890,910 | 306,637 | 278,234 | 15.9 | 64,447 | 71,112 | 4.06 |

***** New libraries prepared from two extracts used in a previous study (*Fu et al.*[8])

**Table S2.7 Summary statistics of the merged data of Bacho Kiro and *Oase1* DNA libraries captured with the SNP Panels 1 and 2 comprising ~1,2 million sites (“1240k”[8,15]).** The number of SNPs on target are determined using the unique fragments longer than 35 base pairs (L≥35 bp) with mapping quality of at least 25 (MQ≥25) that overlap “1240k” SNPs of the “390k”[15] and “840k” arrays[8].

| | All fragments | | | | | | | | Fragments with terminal C-to-T substitutions | | |
|---|---|---|---|---|---|---|---|---|---|---|---|
| **Specimen** | **Library ID** | **Number of sequenced fragments** | **Number of fragments ≥35 bp** | **Number of mapped fragments ≥35bp, MQ≥25** | **Number of mapped fragments on target ≥35bp, MQ≥25** | **Number of unique fragments on target ≥35bp, MQ≥25** | **Number of SNPs on target** | **% of SNPs on target** | **Number of deaminated fragments on target ≥35bp, MQ≥25** | **Number of SNPs on target** | **% of SNPs on target** |
| **Bacho Kiro F6-620** | A11197 | 34,825,937 | 25,896,672 | 9,784,889 | 7,005,396 | 2,734,893 | 792,855 | 64.30 | 494,881 | 371,839 | 30.16 |
| | A12357 | 29,197,573 | 21,757,163 | 7,814,065 | 5,693,242 | 2,574,425 | 724,295 | 58.74 | 391,571 | 309,117 | 25.07 |
| | A12550 | 18,259,858 | 13,355,173 | 4,334,612 | 3,164,364 | 1,672,203 | 738,448 | 59.89 | 399,697 | 313,922 | 25.46 |
| | A12944 | 19,029,747 | 14,243,593 | 4,517,408 | 3,281,791 | 1,584,618 | 718,626 | 58.28 | 374,990 | 298,154 | 24.18 |
| | A15716 | 10,493,214 | 8,374,852 | 3,234,882 | 2,303,292 | 1,378,233 | 676,799 | 54.89 | 321,233 | 264,651 | 21.46 |
| | merged | 111,806,329 | 83,627,453 | 29,685,856 | 21,448,085 | 9,944,372 | 1,129,285 | 91.59 | 1,982,372 | 855,977 | 69.42 |
| **Bacho Kiro AA7-738** | A11198 | 35,590,868 | 25,853,578 | 486,127 | 308,044 | 23,880 | 18,975 | 1.54 | 7,467 | 7,483 | 0.61 |
| | A12358 | 9,168,180 | 6,802,065 | 169,613 | 112,333 | 22,611 | 21,863 | 1.77 | 6,043 | 6,070 | 0.49 |
| | A12554 | 12,871,406 | 9,385,139 | 163,336 | 100,989 | 16,774 | 16,148 | 1.31 | 6,070 | 6,088 | 0.49 |
| | A12945 | 12,639,856 | 9,507,136 | 148,428 | 91,214 | 17,504 | 16,783 | 1.36 | 6,436 | 6,416 | 0.52 |
| | A15717 | 36,305,794 | 17,303,170 | 402,509 | 305,738 | 12,444 | 11,569 | 0.94 | 4,268 | 4,218 | 0.34 |
| | merged | 106,576,104 | 68,851,088 | 1,370,013 | 918,318 | 93,213 | 84,856 | 6.88 | 30,284 | 31,219 | 2.53 |
| **Bacho Kiro BB7-240** | A11199 | 37,087,548 | 25,601,623 | 5,502,148 | 3,730,345 | 644,850 | 379,762 | 30.80 | 180,486 | 164,379 | 13.33 |
| | A12359 | 31,566,711 | 21,129,409 | 3,886,347 | 2,706,321 | 589,028 | 317,485 | 25.75 | 138,820 | 129,353 | 10.49 |
| | A12555 | 20,565,038 | 13,219,973 | 1,897,301 | 1,271,913 | 407,180 | 322,662 | 26.17 | 139,666 | 130,265 | 10.56 |
| | A12946 | 21,483,605 | 14,860,455 | 2,385,989 | 1,651,395 | 481,710 | 364,296 | 29.55 | 162,385 | 148,909 | 12.08 |
| | A15718 | 10,020,844 | 7,419,594 | 1,572,877 | 1,067,462 | 414,636 | 325,394 | 26.39 | 137,117 | 128,007 | 10.38 |
| | merged | 120,723,746 | 82,231,054 | 15,244,662 | 10,427,436 | 2,537,404 | 924,367 | 74.97 | 758,474 | 557,717 | 45.23 |
| **Bacho Kiro CC7-2289** | A11200 | 31,179,013 | 22,258,193 | 75,228 | 36,521 | 6,908 | 4,511 | 0.37 | 1,103 | 1,127 | 0.09 |
| | A12360 | 10,929,510 | 7,736,925 | 39,578 | 22,160 | 6,954 | 7,031 | 0.57 | 1,052 | 1,088 | 0.09 |
| | A12556 | 11,492,889 | 8,234,746 | 31,231 | 15,992 | 4,965 | 4,943 | 0.40 | 996 | 1,002 | 0.08 |
| | A12947 | 9,986,063 | 7,350,723 | 24,106 | 11,915 | 4,363 | 4,381 | 0.36 | 900 | 920 | 0.07 |
| | A15719 | 12,027,896 | 9,105,765 | 32,422 | 17,034 | 4,615 | 4,400 | 0.36 | 905 | 914 | 0.07 |
| | merged | 75,615,371 | 54,686,352 | 202,565 | 103,622 | 27,805 | 27,094 | 2.20 | 4,956 | 5,609 | 0.45 |
| **Bacho Kiro CC7-335** | A11201 | 33,311,280 | 24,714,892 | 5,964,931 | 3,932,017 | 465,307 | 316,935 | 25.70 | 149,782 | 138,732 | 11.25 |
| | A12361 | 29,395,639 | 21,423,625 | 4,140,386 | 2,791,855 | 479,330 | 288,770 | 23.42 | 129,872 | 121,116 | 9.82 |
| | A12557 | 20,686,128 | 14,890,390 | 2,155,639 | 1,421,108 | 364,549 | 292,309 | 23.71 | 135,931 | 126,095 | 10.23 |
| | A12948 | 21,328,124 | 15,643,260 | 2,598,472 | 1,748,502 | 348,854 | 281,960 | 22.87 | 130,234 | 121,219 | 9.83 |
| | A15720 | 10,407,288 | 8,321,215 | 1,786,645 | 1,196,498 | 334,551 | 273,373 | 22.17 | 122,406 | 114,735 | 9.31 |
| | merged | 115,128,459 | 84,993,382 | 16,646,073 | 11,089,980 | 1,992,591 | 854,541 | 69.31 | 668,225 | 504,262 | 40.90 |
| **Bacho Kiro BK-1653** | A11203 | 31,611,263 | 23,452,810 | 7,400,872 | 4,955,232 | 1,574,839 | 644,227 | 52.25 | 188,629 | 171,442 | 13.90 |
| | A12363 | 30,529,019 | 22,310,950 | 5,876,676 | 4,032,969 | 1,629,916 | 585,937 | 47.52 | 159,277 | 146,519 | 11.88 |
| | A12562 | 20,926,241 | 14,800,165 | 3,715,610 | 2,581,475 | 1,039,486 | 592,899 | 48.09 | 158,742 | 146,159 | 11.85 |
| | A12950 | 20,558,862 | 14,605,842 | 3,625,387 | 2,533,013 | 996,740 | 576,910 | 46.79 | 148,692 | 137,858 | 11.18 |
| | A15722 | 31,694,836 | 16,470,059 | 7,508,034 | 5,902,569 | 1,098,165 | 620,721 | 50.34 | 158,615 | 147,452 | 11.96 |
| | merged | 135,320,221 | 91,639,826 | 28,126,579 | 20,005,258 | 6,339,146 | 1,062,658 | 86.13 | 813,955 | 577,031 | 46.80 |
| **Oase1** | A12968* | 18,310,229 | 12,226,533 | 883,648 | 579,817 | 230,378 | 201,331 | 16.33 | 49,345 | 48,955 | 3.97 |
| | A12967* | 17,191,082 | 11,647,238 | 400,609 | 264,188 | 112,986 | 107,796 | 8.74 | 20,594 | 21,029 | 1.71 |
| | A12384 | 17,579,948 | 11,275,842 | 948,711 | 654,060 | 69,694 | 66,541 | 5.40 | 15,525 | 15,625 | 1.27 |
| | A12590 | 21,813,393 | 14,214,612 | 1,047,462 | 701,004 | 66,203 | 63,330 | 5.14 | 16,368 | 16,399 | 1.33 |
| | A12955 | 19,148,461 | 12,500,236 | 669,842 | 437,921 | 59,770 | 57,511 | 4.66 | 14,455 | 14,596 | 1.18 |
| | F9015 | 21,804,293 | 13,452,555 | 928,397 | 613,026 | 52,163 | 50,564 | 4.10 | 12,978 | 13,088 | 1.06 |
| | merged | 115,847,406 | 75,317,016 | 4,878,669 | 3,250,016 | 591,194 | 429,569 | 34.84 | 129,265 | 121,388 | 9.84 |

* New libraries prepared from two extracts used in a previous study (*Fu et al.*[8])

**Table S2.8 Summary statistics of the merged data of Bacho Kiro and *Oase1* DNA libraries captured with the SNP Panels 1, 2 and 3 comprising 2.2 million sites (“2200k”[8,15]).** Results per library detailed in the table. The number of SNPs on target are determined using the unique fragments longer than 35 base pairs (L≥35 bp) with mapping quality of at least 25 (MQ≥25) and that overlap ~2.2 million SNPs of the “390k”[15], “840k”[8] and “1000k” arrays[8].

| | All fragments | | | | | | | | Fragments with terminal C-to-T substitutions | | |
|---|---|---|---|---|---|---|---|---|---|---|---|
| **Specimen** | **Library ID** | **Number of sequenced fragments** | **Number of fragments ≥35 bp** | **Number of mapped fragments ≥35bp, MQ≥25** | **Number of mapped fragments on target ≥35bp, MQ≥25** | **Number of unique fragments on target ≥35bp, MQ≥25** | **Number of SNPs on target** | **% of SNPs on target** | **Number of deaminated fragments on target ≥35bp, MQ≥25** | **Number of SNPs on target** | **% of SNPs on target** |
| **Bacho Kiro F6-620** | A11197 | 56,817,171 | 42,739,090 | 15,936,366 | 11,244,167 | 4,115,646 | 1,504,437 | 70.15 | 1,058,735 | 764,952 | 35.67 |
| | A12357 | 46,412,901 | 34,943,528 | 12,776,135 | 9,297,117 | 3,915,997 | 1,447,043 | 67.48 | 945,338 | 698,557 | 32.57 |
| | A12550 | 29,523,451 | 21,651,782 | 7,079,392 | 5,086,346 | 2,557,164 | 1,184,154 | 55.22 | 605,245 | 494,054 | 23.04 |
| | A12944 | 27,592,961 | 20,684,874 | 6,550,313 | 4,721,438 | 2,285,148 | 1,107,439 | 51.64 | 535,144 | 444,966 | 20.75 |
| | A15716 | 21,068,663 | 16,897,935 | 6,573,652 | 4,739,589 | 2,148,695 | 1,110,491 | 51.78 | 494,820 | 424,879 | 19.81 |
| | merged | 181,415,147 | 136,917,209 | 48,915,858 | 35,088,657 | 15,022,650 | 1,867,749 | 87.09 | 3,639,282 | 1,362,667 | 64.54 |
| **Bacho Kiro AA7-738** | A11198 | 58,681,066 | 43,053,237 | 739,703 | 450,345 | 34,952 | 33,992 | 1.59 | 13,082 | 13,288 | 0.62 |
| | A12358 | 16,676,690 | 12,484,437 | 289,033 | 191,569 | 33,009 | 32,780 | 1.53 | 8,706 | 8,920 | 0.42 |
| | A12554 | 22,643,136 | 16,654,000 | 294,471 | 178,049 | 23,439 | 23,170 | 1.08 | 8,400 | 8,657 | 0.40 |
| | A12945 | 22,056,042 | 16,732,805 | 254,593 | 153,785 | 24,590 | 24,275 | 1.13 | 9,027 | 9,261 | 0.43 |
| | A15717 | 48,070,409 | 22,022,511 | 470,195 | 354,291 | 15,203 | 14,665 | 0.68 | 5,160 | 5,256 | 0.25 |
| | merged | 168,127,343 | 110,946,990 | 2,047,995 | 1,328,039 | 131,193 | 123,265 | 5.75 | 44,375 | 44,610 | 2.08 |
| **Bacho Kiro BB7-240** | A11199 | 62,282,800 | 43,076,677 | 8,625,924 | 5,748,173 | 943,806 | 710,330 | 33.12 | 336,713 | 309,131 | 14.42 |
| | A12359 | 52,263,445 | 35,252,377 | 6,420,632 | 4,419,909 | 861,912 | 661,758 | 30.86 | 296,216 | 275,364 | 12.84 |
| | A12555 | 30,703,077 | 19,714,046 | 2,776,142 | 1,822,904 | 578,138 | 478,374 | 22.31 | 196,214 | 189,300 | 8.83 |
| | A12946 | 32,007,473 | 22,072,415 | 3,446,475 | 2,351,070 | 685,063 | 542,274 | 25.29 | 228,867 | 217,287 | 10.13 |
| | A15718 | 18,857,516 | 13,959,798 | 2,966,341 | 2,035,310 | 593,947 | 488,639 | 22.79 | 194,726 | 188,221 | 8.78 |
| | merged | 196,114,311 | 134,075,313 | 24,235,514 | 16,377,366 | 3,662,866 | 1,451,175 | 67.67 | 1,252,736 | 840,421 | 39.19 |
| **Bacho Kiro CC7-2289** | A11200 | 52,653,555 | 37,943,882 | 132,979 | 55,859 | 9,826 | 9,941 | 0.46 | 2,377 | 2,482 | 0.12 |
| | A12360 | 18,733,502 | 13,430,985 | 70,741 | 37,338 | 8,930 | 9,341 | 0.44 | 1,322 | 1,405 | 0.07 |
| | A12556 | 20,392,805 | 14,728,695 | 59,180 | 27,596 | 6,592 | 6,789 | 0.32 | 1,346 | 1,404 | 0.07 |
| | A12947 | 18,167,388 | 13,475,445 | 46,543 | 20,229 | 5,832 | 6,057 | 0.28 | 1,171 | 1,236 | 0.06 |
| | A15719 | 20,612,547 | 15,740,666 | 60,670 | 28,575 | 6,058 | 6,030 | 0.28 | 1,156 | 1,197 | 0.06 |
| | merged | 130,559,797 | 95,319,673 | 370,113 | 169,597 | 37,238 | 37,590 | 1.75 | 7,372 | 7,696 | 0.36 |
| **Bacho Kiro CC7-335** | A11201 | 55,301,835 | 41,173,364 | 9,565,269 | 6,206,717 | 702,397 | 569,300 | 26.55 | 264,767 | 248,606 | 11.59 |
| | A12361 | 49,895,859 | 36,673,473 | 7,367,752 | 4,939,653 | 722,934 | 573,966 | 26.76 | 261,318 | 244,569 | 11.40 |
| | A12557 | 30,509,418 | 21,993,264 | 3,170,647 | 2,035,590 | 529,901 | 440,993 | 20.56 | 196,211 | 187,753 | 8.76 |
| | A12948 | 32,453,892 | 23,784,249 | 3,945,576 | 2,616,487 | 512,068 | 428,364 | 19.97 | 189,611 | 181,578 | 8.47 |
| | A15720 | 19,432,194 | 15,590,987 | 3,504,402 | 2,382,864 | 500,486 | 423,567 | 19.75 | 181,570 | 175,325 | 8.18 |
| | merged | 187,593,198 | 139,215,337 | 27,553,646 | 18,181,311 | 2,967,786 | 1,353,413 | 63.11 | 1,093,477 | 776,283 | 36.20 |
| **Bacho Kiro BK-1653** | A11203 | 54,170,261 | 40,183,676 | 12,422,963 | 8,226,929 | 2,352,606 | 1,223,725 | 57.06 | 377,170 | 344,695 | 16.07 |
| | A12363 | 49,766,354 | 36,620,024 | 9,948,926 | 6,813,993 | 2,402,877 | 1,191,416 | 55.56 | 369,224 | 335,185 | 15.63 |
| | A12562 | 32,337,191 | 22,878,291 | 5,634,204 | 3,836,270 | 1,523,656 | 916,582 | 42.74 | 230,660 | 220,383 | 10.28 |
| | A12950 | 30,217,360 | 21,445,227 | 5,355,935 | 3,707,402 | 1,425,078 | 876,042 | 40.85 | 210,684 | 202,809 | 9.46 |
| | A15722 | 41,216,350 | 20,511,006 | 9,294,451 | 7,279,052 | 1,393,895 | 868,204 | 40.49 | 199,595 | 193,841 | 9.04 |
| | merged | 207,707,516 | 141,638,224 | 42,656,479 | 29,863,646 | 9,098,112 | 1,728,159 | 80.59 | 1,387,333 | 874,287 | 40.77 |
| **Oase1** | A12968* | 28,223,311 | 18,752,585 | 1,343,789 | 870,557 | 326,422 | 296,458 | 13.82 | 68,880 | 70,514 | 3.29 |
| | A12967* | 26,572,354 | 17,978,003 | 611,051 | 394,828 | 157,704 | 156,272 | 7.29 | 28,229 | 29,716 | 1.39 |
| | A12384 | 26,906,689 | 17,318,241 | 1,409,840 | 954,614 | 104,016 | 102,077 | 4.76 | 22,677 | 23,361 | 1.09 |
| | A12590 | 32,239,343 | 20,968,522 | 1,530,134 | 1,010,243 | 96,272 | 94,498 | 4.41 | 23,320 | 23,929 | 1.12 |
| | A12955 | 29,352,737 | 19,185,421 | 1,028,770 | 655,370 | 88,281 | 87,220 | 4.07 | 20,853 | 21,525 | 1.00 |
| | F9015 | 31,901,491 | 19,700,827 | 1,302,320 | 836,823 | 77,891 | 77,405 | 3.61 | 19,184 | 19,785 | 0.92 |
| | merged | 175,195,925 | 113,903,599 | 7,225,904 | 4,722,435 | 850,586 | 646,646 | 30.15 | 183,143 | 177,336 | 8.27 |

* New libraries prepared from two extracts used in a previous study (*Fu et al.*[8])

**Table S2.9 Summary statistics of the Bacho Kiro libraries enriched for ~6.9 Mb of the Y chromosome[16].** The coverage on target is determined using the unique fragments longer than 35 base pairs (L≥35 bp) with mapping quality of at least 25 (MQ≥25) and that overlap the target regions.

| | All fragments | | | | | | | Fragments with terminal C-to-T substitutions | |
|---|---|---|---|---|---|---|---|---|---|
| **Specimen** | **Library ID** | **Number of sequenced fragments** | **Number of fragments ≥35 bp** | **Number of mapped fragments ≥35bp, MQ≥25** | **Number of mapped fragments on target ≥35bp, MQ≥25** | **Number of unique fragments on target ≥35bp, MQ≥25** | **Coverage on target** | **Number of deaminated fragments on target ≥35bp, MQ≥25** | **Coverage on target** |
| **Bacho Kiro F6-620** | A11197 | 6,649,928 | 5,127,264 | 2,500,217 | 2,036,336 | 308,614 | 2.75 | 78,144 | 0.68 |
| | A12357 | 5,746,174 | 4,429,045 | 2,277,437 | 1,869,731 | 350,547 | 3.23 | 83,205 | 0.74 |
| | A12550 | 6,429,418 | 5,015,419 | 2,569,376 | 2,090,273 | 361,743 | 3.34 | 84,186 | 0.75 |
| | A12944 | 5,272,554 | 3,962,253 | 1,868,796 | 1,504,334 | 316,018 | 2.86 | 73,418 | 0.64 |
| | A15716 | 7,000,727 | 5,682,409 | 2,790,390 | 2,254,962 | 328,009 | 3.02 | 75,048 | 0.67 |
| | **merged** | 31,098,801 | 24,216,390 | 12,006,216 | 9,755,636 | **1,664,931** | **15.20** | **394,001** | **3.48** |
| **Bacho Kiro BB7-240** | A11199 | 6,602,187 | 4,757,307 | 1,896,804 | 1,577,430 | 57,527 | 0.44 | 19,984 | 0.15 |
| | A12359 | 6,987,822 | 5,099,681 | 2,168,018 | 1,813,504 | 58,667 | 0.45 | 19,984 | 0.15 |
| | A12555 | 8,244,030 | 5,797,923 | 2,447,936 | 2,042,714 | 63,411 | 0.49 | 21,114 | 0.16 |
| | A12946 | 6,516,377 | 4,719,122 | 1,843,243 | 1,506,249 | 70,092 | 0.55 | 22,889 | 0.18 |
| | A15718 | 6,816,864 | 5,315,156 | 1,923,820 | 1,585,029 | 65,126 | 0.52 | 21,059 | 0.16 |
| | **merged** | 35,167,280 | 25,689,189 | 10,279,821 | 8,524,926 | **314,823** | **2.46** | **105,030** | **0.80** |
| **Bacho Kiro CC7-335** | A11201 | 5,892,475 | 4,509,883 | 1,889,636 | 1,558,502 | 35,318 | 0.26 | 13,064 | 0.10 |
| | A12361 | 6,960,643 | 5,403,081 | 2,256,934 | 1,878,258 | 42,808 | 0.32 | 15,162 | 0.11 |
| | A12557 | 10,484,235 | 7,808,450 | 3,181,164 | 2,638,950 | 46,134 | 0.34 | 16,888 | 0.12 |
| | A12948 | 7,246,254 | 5,614,926 | 2,143,855 | 1,759,374 | 39,615 | 0.30 | 14,272 | 0.11 |
| | A15720 | 7,224,312 | 5,900,108 | 2,279,432 | 1,882,344 | 39,383 | 0.30 | 13,894 | 0.10 |
| | **merged** | 37,807,919 | 29,236,448 | 11,751,021 | 9,717,428 | **203,258** | **1.52** | **73,280** | **0.54** |

## Table S2.10 Contamination estimates for Bacho Kiro Cave and *Oase 1* specimens.

| Specimen | Mitochondrial DNA contamination estimates | | Sex-based contamination estimates for male individuals (ANGSD)[24] >200 SNPs covered | | AuthentiCT[27] contamination estimates |
|---|---|---|---|---|---|
| | all fragments [95% CI] | deaminated fragments [95% CI] | all fragments [SE] | deaminated fragments [SE] | all fragments [SE] |
| **Bacho Kiro F6-620** | 0.19 [0.08-0.44] | 0.12 [0.02-0.65] | 1.62 [0.12] | 1.09 [0.40] | 2.38 [0.54] |
| **Bacho Kiro AA7-738** | 0.75 [0.21-2.70] | 1.59 [0.28-8.46] | - | - | 11.05 [0.36] |
| **Bacho Kiro BB7-240** | 2.91 [2.21-3.83] | 0.43 [0.15-1.26] | 2.75 [0.37] | 1.27 [1.03] | 4.72 [0.42] |
| **Bacho Kiro CC7-2289** | 1.00 [0-2.00] | 0 [0-0.05] | - | - | 42.36 [0.64] |
| **Bacho Kiro CC7-335** | 0.42 [0.21-0.83] | 0 [0-0.49] | 3.41 [0.53] | 2.13 [1.24] | 4.46 [0.31] |
| **Bacho Kiro BK-1653** | 1.63 [1.19-2.22] | 1.14 [0.39-3.30] | - | - | 3.53 [0.71] |
| **Oase1 new extract** | 46.22 [40.50-52.39] | 4.51 [0-9.34] | 21.74 [4.56] | - | 23.62 [0.44] |
| **Oase1 old extracts** | 68.13 [62.48-73.29] | 9.04 [2.83-15.25] | | | 28.98 [0.48] |

*SE – standard error

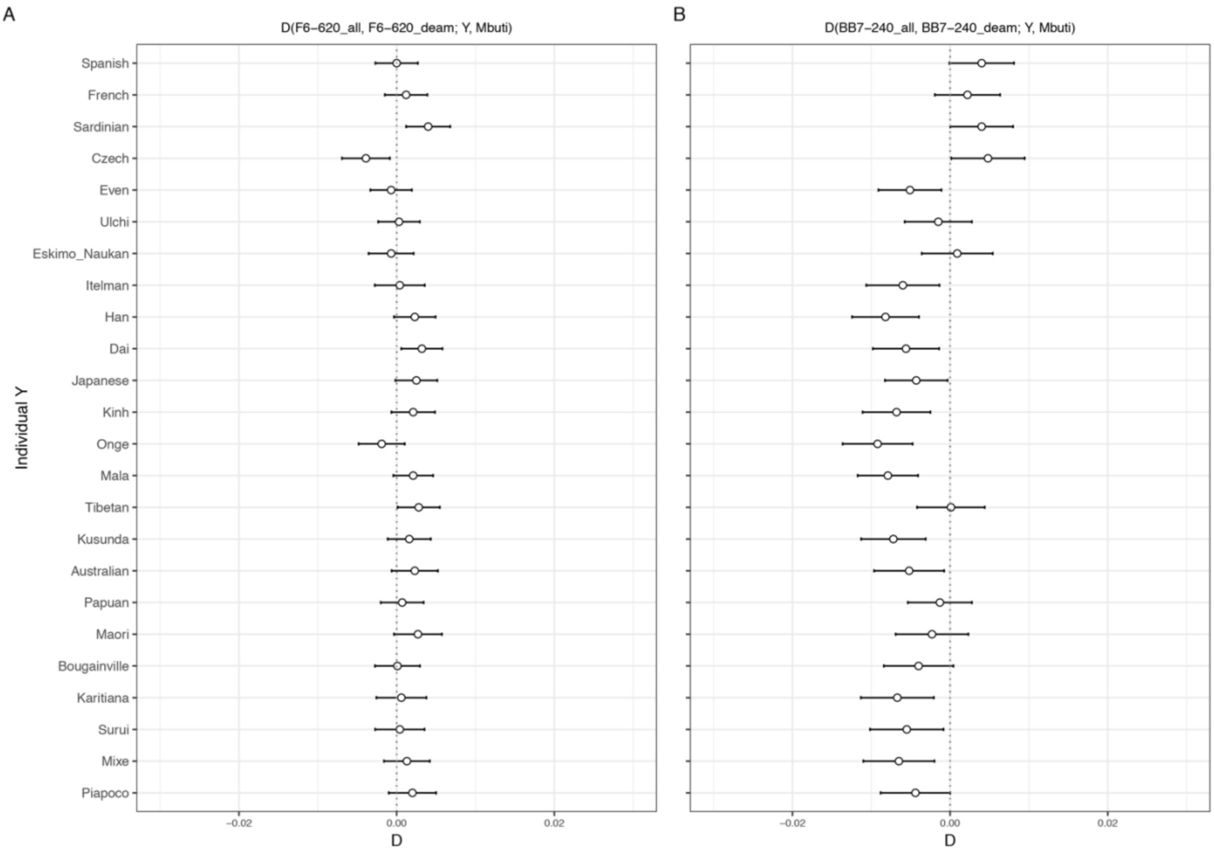


**Figure S2.1 Statistics of the form *D(Bacho Kiro$_1$ all fragments, Bacho Kiro$_1$ deaminated fragments; Test, Mbuti)* comparing derived allele sharing between all fragments and deaminated fragments only from a given specimen with a present-day human population from SGDP[29]. A)** Bacho Kiro *F6-620* and **B)** Bacho Kiro *BB7-240*. Points plotted on the *x*-axes correspond to the calculated *D*-values using ADMIXTOOLS[27] and as implemented in R-packge *admixr*[28]. Open circles indicate a non-significant Z-score, or $|Z| < 3$. Whiskers correspond to one standard error (SE) calculated using a Weighted Block Jackknife[27,30] and a block size of 5 Mb across all autosomes of "2200k" SNP Panel[8,15] covered by both all and deaminated DNA fragments of Bacho Kiro *F6-620* (panel A, n(SNPs) = 1,286,485) and Bacho Kiro *BB7-240* (panel B, n(SNPs) = 785,445). Three Mbuti individuals from SGDP[29] were used as outgroup.

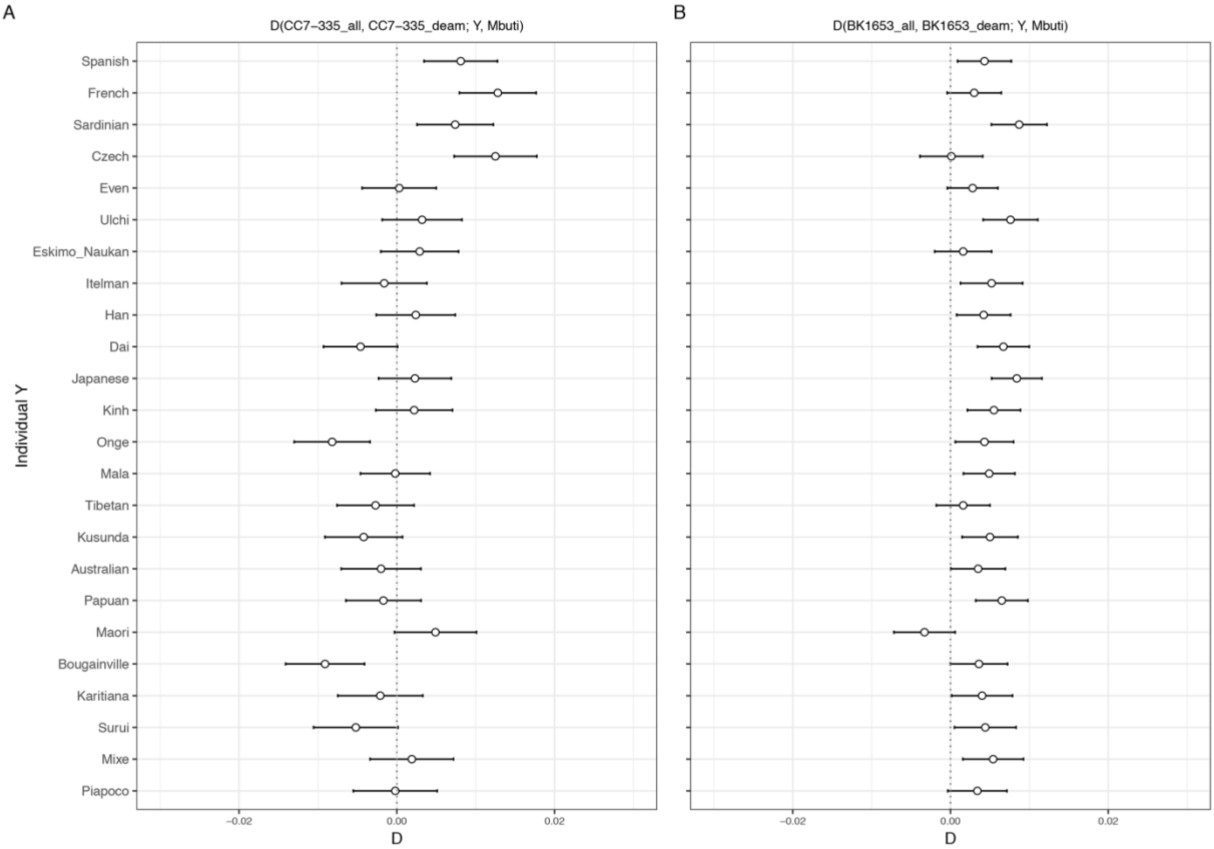


**Figure S2.2 Statistics of the form *D(Bacho Kiro$_1$ all fragments, Bacho Kiro$_1$ deaminated fragments; Test, Mbuti)* comparing derived allele sharing between all fragments and deaminated fragments only from a given specimen with a present-day human population from SGDP[29]. A)** Bacho Kiro *CC7-335* and **B)** Bacho Kiro *BK1653*. Points plotted on the x-axes correspond to the calculated *D*-values using ADMIXTOOLS[27] and as implemented in R-packge *admixr*[28]. Open circles indicate a non-significant Z-score, or $|Z| < 3$. Whiskers correspond to one standard error (SE) calculated using a Weighted Block Jackknife[27,30] and a block size of 5 Mb across all autosomes of "2200k"[8,15] SNP Panel covered by both all and deaminated DNA fragments of Bacho Kiro *CC7-335* (panel A, n(SNPs) = 720,319) and Bacho Kiro *BK1653* (panel B, n(SNPs) = 822,323). Three Mbuti individuals from SGDP[29] were used as outgroup.

# Supplementary Information 3

## Datasets for downstream analyses

We merged the data from the newly sequenced specimens detailed in the Supplementary Information 2 with datasets of previously published ancient and present-day humans, as well as the archaics. We used three different SNP panels (“1240k”, “2240k” and “Archaic admixture”) for all downstream analyses.

### Analysis panels

**“1240K” panel**

This panel consists of combined 1,233,013 SNPs from the Panel 1 detailed in *Haak et al.* (“390k”)[1] and the Panel 2 detailed in Fu *et al.* (“840k”)[2]. These data include genotypes of 2,109 ancient and 2,974 present-day individuals compiled from published studies and available in the EIGENSTRAT format[3] through the [https://reich.hms.harvard.edu/downloadable-genotypes-present-day-and-ancient-dna-data-compiled-published-papers/](https://reich.hms.harvard.edu/downloadable-genotypes-present-day-and-ancient-dna-data-compiled-published-papers/) (version 37.2, released February 22, 2019).

**“2200K” panel**

This panel consists of an extended panel of SNPs represented in the “1240k” together with the SNP Panel 3 detailed in *Fu et al.* (“1000k”)[2] , for a total of 2,144,502 SNPs across the genome[2,4]. These data include published genetic data of ancient modern humans obtained through hybridization captures[2,4,5] and a range of present-day[6-8] and ancient modern humans[9-24], as well as the archaics[7,8,25-27], for which whole-genome shotgun data of varying coverage are available. For all low-coverage individuals we randomly sampled an allele at these ~2.2 million SNPs which passed the filters described in the Supplementary Information 2 (unique fragments of at least 35 bp with a mapping quality ≥25 and a base quality ≥20). For the high-coverage individuals we used their respective published genotypes intersecting ~2.2 million SNPs, with the exception of *Ust'Ishim*[23], the *Altai* Neandertal[7], the *Denisova 3* individual[8], *Loschbour*[19] and *Stuttgart*[19] for which we used the genotypes re-called with snpAD[28], and as described in detail in *Prüfer et al.*[25].

**"Archaic admixture" panel**

"Archaic admixture" panel consists of 1,749,385 (~1.7 million) SNPs informative about the Neandertal and Denisovan ancestry in a studied individual[2]. These data include 21 ancient modern humans directly enriched for these sites[2,4,5], as well as the genotypes of present-day[6-8] and ancient modern humans[9-24], as well as the archaics[7,8,25-27], for which whole-genome shotgun data are available and that were intersected with ~1.7 million SNPs of the "Archaic admixture" panel using *BEDTools*[29] (version: 2.24.0).

**References for SI3:**


1 Haak, W. *et al.* Massive migration from the steppe was a source for Indo-European languages in Europe. *Nature* **522**, 207-211, doi:10.1038/nature14317 (2015).

2 Fu, Q. *et al.* An early modern human from Romania with a recent Neanderthal ancestor. *Nature* **524**, 216-219, doi:10.1038/nature14558 (2015).

3 Patterson, N. *et al.* Ancient admixture in human history. *Genetics* **192**, 1065-1093, doi:10.1534/genetics.112.145037 (2012).

4 Fu, Q. *et al.* The genetic history of Ice Age Europe. *Nature* **534**, 200-205, doi:10.1038/nature17993 (2016).

5 Yang, M. A. *et al.* 40,000-Year-Old Individual from Asia Provides Insight into Early Population Structure in Eurasia. *Curr. Biol.* **27**, 3202-3208. e3209, doi: 10.1016/j.cub.2017.09.030 (2017).

6 Mallick, S. *et al.* The Simons Genome Diversity Project: 300 genomes from 142 diverse populations. *Nature* **538**, 201-206, doi:10.1038/nature18964 (2016).

7 Prüfer, K. *et al.* The complete genome sequence of a Neanderthal from the Altai Mountains. *Nature* **505**, 43-49, doi:10.1038/nature12886 (2014).

8 Meyer, M. *et al.* A high-coverage genome sequence from an archaic Denisovan individual. *Science* **338**, 222-226, doi:10.1126/science.1224344 (2012).

9 Seguin-Orlando, A. *et al.* Genomic structure in Europeans dating back at least 36,200 years. *Science* **346**, 1113-1118, doi:10.1126/science.aaa0114 (2014).

10 Raghavan, M. *et al.* Upper Palaeolithic Siberian genome reveals dual ancestry of Native Americans. *Nature* **505**, 87-91, doi:10.1038/nature12736 (2014).

11 Rasmussen, M. *et al.* Ancient human genome sequence of an extinct Palaeo-Eskimo. *Nature* **463**, 757-762, doi:10.1038/nature08835 (2010).

12 Rasmussen, M. *et al.* The genome of a Late Pleistocene human from a Clovis burial site in western Montana. *Nature* **506**, 225-229, doi:10.1038/nature13025 (2014).

13 Rasmussen, M. *et al.* The ancestry and affiliations of Kennewick Man. *Nature* **523**, 455-458, doi:10.1038/nature14625 (2015).
14 Sikora, M. *et al.* Ancient genomes show social and reproductive behavior of early Upper Paleolithic foragers. *Science* **358**, 659-662, doi: 10.1126/science.aao1807 (2017).
15 Sikora, M. *et al.* The population history of northeastern Siberia since the Pleistocene. *Nature* **570**, 182-188, doi: 10.1038/s41586-019-1279-z (2019).
16 Olalde, I. *et al.* Derived immune and ancestral pigmentation alleles in a 7,000-year-old Mesolithic European. *Nature* **507**, 225-228, doi:10.1038/nature12960 (2014).
17 Gallego Llorente, M. *et al.* Ancient Ethiopian genome reveals extensive Eurasian admixture throughout the African continent. *Science* **350**, 820-822, doi:10.1126/science.aad2879 (2015).
18 Keller, A. *et al.* New insights into the Tyrolean Iceman's origin and phenotype as inferred by whole-genome sequencing. *Nat. Commun.* **3**, 698, doi:10.1038/ncomms1701 (2012).
19 Lazaridis, I. *et al.* Ancient human genomes suggest three ancestral populations for present-day Europeans. *Nature* **513**, 409-413, doi:10.1038/nature13673 (2014).
20 Gamba, C. *et al.* Genome flux and stasis in a five millennium transect of European prehistory. *Nat. Commun.* **5**, 5257, doi:10.1038/ncomms6257 (2014).
21 Moreno-Mayar, J. V. *et al.* Terminal Pleistocene Alaskan genome reveals first founding population of Native Americans. *Nature* **553**, 203-207, doi: 10.1038/nature25173 (2018).
22 Moreno-Mayar, J. V. *et al.* Early human dispersals within the Americas. *Science* **362**, eaav2621, doi:10.1126/science.aav2621 (2018).
23 Fu, Q. *et al.* Genome sequence of a 45,000-year-old modern human from western Siberia. *Nature* **514**, 445-449, doi:10.1038/nature13810 (2014).
24 Jones, E. R. *et al.* Upper Palaeolithic genomes reveal deep roots of modern Eurasians. *Nat. Commun.* **6**, 8912, doi:10.1038/ncomms9912 (2015).
25 Prüfer, K. *et al.* A high-coverage Neandertal genome from Vindija Cave in Croatia. *Science* **358**, 655-658, doi: 10.1126/science.aao1887 (2017).
26 Hajdinjak, M. *et al.* Reconstructing the genetic history of late Neanderthals. *Nature* **555**, 652-656, doi:10.1038/nature26151 (2018).
27 Mafessoni, F. *et al.* A high-coverage Neandertal genome from Chagyrskaya Cave. *Proc. Natl. Acad. Sci. U.S.A.* **117**, 15132-15136, doi:10.1073/pnas.2004944117 (2020).

28 Prüfer, K. snpAD: An ancient DNA genotype caller. *Bioinformatics* **34**, 4165-4171, doi: 10.1093/bioinformatics/bty507 (2018).

29 Quinlan, A. R. & Hall, I. M. BEDTools: a flexible suite of utilities for comparing genomic features. *Bioinformatics* **26**, 841-842, doi: 10.1093/bioinformatics/btq033 (2010).

# Supplementary Information 4

## Relatedness, sex determination and Y chromosome analyses

**Relatedness between different specimens**

Given the fragmentary nature of human specimens recovered from Bacho Kiro Cave and our previous results demonstrating that the molar (specimen *F6-620*) and one of the fragments (specimen *AA7-738*) have an identical mtDNA sequence[1], we sought to determine the degree of relationship among IUP Bacho Kiro Cave specimens.

To assess whether specimens *F6-620* and *AA7-738* might come from the same individual, we used a similar approach as described in *Mittnik et al.*[2]. To obtain a baseline of what could be expected for specimens stemming from the same individual, we first calculated a pairwise mismatch rate between different libraries stemming from the same specimen using random allele calls of the autosomal data from the "2200k" SNP panel (Supplementary Information 3). The median value of the pairwise mismatch rate between different libraries originating from the same extract is 0.125 (interquartile range: 0.118-0.135) (Tab. S4.1 and Extended Data Fig. 2B). To obtain values of what is expected for unrelated individuals, we then calculated a pairwise mismatch rate among a range of ancient modern humans from previously published studies using random allele calls of "2200k" SNP panel (Supplementary Information 3). Median value of a pairwise mismatch rate between unrelated ancient individuals is 0.241 (interquartile range: 0.228-0.255) (Extended Data Fig. 2B).

When calculating a pairwise mismatch rate between Bacho Kiro Cave specimens, we find a pairwise mismatch rate of 0.125 between the specimens *F6-620* and *AA7-738*, which is identified as an outlier by a chi-square ($\chi^2$) outlier test (p-value = 0.001). This value is indistinguishable of what is found between different libraries originating from the same specimen (chi-square ($\chi^2$) outlier test > 0.05). A median value of a pairwise mismatch rate between other specimens from Bacho Kiro Cave is 0.231, with a first quartile at 0.225 and a third quartile at 0.251, which is not significantly different from what is found between pairs of unrelated ancient individuals from previously published studies (Extended Data Fig. 2B).

Furthermore, given the identical start and end coordinates of identified Neandertal segments across the genomes of *F6-620* and *AA7-738* (Supplementary Information 8, Extended Data Fig. 8A and Fig. S8.1), we conclude that the specimen *F6-620* found in the Layer J in the Main Sector of the Bacho Kiro Cave and the specimen *AA7-738* found in the Layer I of the Niche 1 belonged to the same individual.

**Sex determination**

We determined the sex of Bacho Kiro Cave specimens by counting the number of fragments aligning to the X chromosome and the autosomes[3,4]. Based on the expected ratios for female and male individuals, we concluded that the specimens *F6-620, AA7-738, BB7-240* and *CC7-335* belonged to male, whereas *CC7-2289* and *BK1653* belonged to female individuals (Extended Data Fig. 2A).

**Y chromosome analyses**

We enriched the amplified libraries of identified male individuals from Bacho Kiro Cave for ~6.9 Mb on the Y chromosome[5] and as detailed in Supplementary Information 2. Since we determined that specimens *F6-620* and *AA7-738* stem from the same individual, we enriched the amplified libraries of the specimen *F6-620* rather than *AA7-738* as they contain two orders of magnitude more nuclear DNA (Tab. S2.1, Supplementary Information 2) and have lower contamination estimates (Tab. S2.10, Supplementary Information 2).

After applying the filters detailed in Supplementary Information 2 and restricting our analyses to fragments of at least 35 bp with a mapping quality of at least 25 that overlap ~6.9 Mb of the Y chromosome, we obtained 15.2-fold coverage at these positions for Bacho Kiro Cave specimen *F6-620*, 2.5-fold for *BB7-240* and 1.5-fold for *CC7-335*, respectively (Tab. S2.9). We called an allele at each position by majority call requiring a minimum coverage of 3 for the specimens *F6-620* and *BB7-240* and of 2 for the specimen *CC7-335* using *bam-caller* (https://github.com/bodkan/bam-caller, version: 0.1). Furthermore, we used an aDNA-specific genotype caller *snpAD*[6] (version: 0.3.4) for generating genotype calls for the Bacho Kiro *F6-620* given the high coverage of the enriched Y chromosome regions for this individual.

**Y chromosome haplogroup assignment**

We extracted genotypes at SNP positions included in the Y-haplogroup tree from the International Society of Genetic Genealogy (ISOGG, http://www.isogg.org, version: 13.38) to determine the Y chromosome haplogroup of male individuals. Haplogroup calling was performed using *yHaplo*[7] (version: 1.0.18) using default parameters with the exception of setting the parameter *ancStopThresh* to 1e6 in order to achieve a full Y-haplogroup tree search.

All three male individuals had Y chromosome haplogroups that fell basal to the supercluster of haplogroups CT and F that are typically found outside of Sub-Saharan Africa today[8]. The specimen with the highest coverage, *F6-620*, was assigned to Y haplogroup F-M89. Bacho Kiro *F6-620* carries 22 out of 25 defining mutations of C and FT described by

*Karafet et al.*[8], with no fragments covering three additional defining mutations (M213, P142, and P187). Next to these 22 mutations, *F6-620* carries three additional mutations that have been identified to define haplogroup F: M235, P316, and L132.1 (ISOGG, version 13.38). Specimens *BB7-240* and *CC7-335* were both identified as having Y haplogroup C1 with the defining SNP F3393. However, due to their lower coverage and only three and two additional mutations defining haplogroup C, respectively, it was not possible to further assess the exact location of these lineages within haplogroup C.

Both haplogroups F and C1 are rare in present-day individuals. While lineages of haplogroup C are found commonly in males from East Asia and Oceania, haplogroup F and C1 and were only found in a few males from mainland Southeast Asia and Japan[9,10]. Therefore, it had been hypothesized that these haplogroups emerged in Asia after the human migration out of Africa. However, since the coalescent time for the dispersal of all non-African haplogroups, including C and F, has been estimated to 47-52 kya (95% CI: 36-62 kya)[11] and thus overlaps with the estimated age of the Bacho Kiro Cave specimens, it is more plausible to assume that these Y chromosome haplogroups emerged closely after the migration out of Africa and spread through Eurasia before being later replaced in all parts of Eurasia but East Asia and Oceania.

**References SI4:**


1 Hublin, J.-J. et al. Initial Upper Palaeolithic Homo sapiens from Bacho Kiro Cave, Bulgaria. *Nature* **581**, 299-302, doi:10.1038/s41586-020-2259-z (2020).

2 Mittnik, A. *et al.* Kinship-based social inequality in Bronze Age Europe. *Science* **366**, 731-734, doi: 10.1126/science.aax6219 (2019).

3 Meyer, M. *et al.* Nuclear DNA sequences from the Middle Pleistocene Sima de los Huesos hominins. *Nature* **531**, 504-507, doi:10.1038/nature17405 (2016).

4 Fu, Q. *et al.* The genetic history of Ice Age Europe. *Nature* **534**, 200-205, doi:10.1038/nature17993 (2016).

5 Petr, M. *et al.* The evolutionary history of Neanderthal and Denisovan Y chromosomes. *Science* **369**, 1653-1656, doi: 10.1126/science.abb6460 (2020).

6 Prüfer, K. snpAD: An ancient DNA genotype caller. *Bioinformatics* **34**, 4165-4171, doi: 10.1093/bioinformatics/bty507 (2018).

7 Poznik, G. D. Identifying Y-chromosome haplogroups in arbitrarily large samples of sequenced or genotyped men. *bioRxiv*, 088716 (2016).

8 Karafet, T. M. *et al.* New binary polymorphisms reshape and increase resolution of the human Y chromosomal haplogroup tree. *Genome Res.* **18**, 830-838, doi: 10.1101/gr.7172008 (2008).

9 Poznik, G. D. *et al.* Punctuated bursts in human male demography inferred from 1,244 worldwide Y-chromosome sequences. *Nat. Genet.* **48**, 593-599, doi: 10.1038/ng.3559 (2016).

10 Kutanan, W. *et al.* Contrasting paternal and maternal genetic histories of Thai and Lao populations. *Mol. Biol. Evol.* **36**, 1490-1506, doi: 10.1093/molbev/msz083 (2019).

11 Karmin, M. *et al.* A recent bottleneck of Y chromosome diversity coincides with a global change in culture. *Genome Res.* **25**, 459-466, doi: 10.1101/gr.186684.114 (2015).

**Table S4.1 The pairwise mismatch rate between different libraries of Bacho Kiro Cave specimens originating from the same extract.**

| Specimen | Library1 | Library2 | nSNPs | Number of mismatches | Mismatch | Specimen | Library1 | Library2 | nSNPs | Number of mismatches | Mismatch |
|---|---|---|---|---|---|---|---|---|---|---|---|
| BK_F6_620 | A11197 | A12357 | 1,149,331 | 140,259 | 0.12 | BK_CC7_2289 | A11200 | A12360 | 82 | 9 | 0.11 |
| | A11197 | A12550 | 972,999 | 118,929 | 0.12 | | A11200 | A12556 | 56 | 12 | 0.21 |
| | A11197 | A12944 | 919,264 | 112,759 | 0.12 | | A11200 | A12947 | 46 | 9 | 0.20 |
| | A11197 | A15716 | 907,171 | 111,301 | 0.12 | | A11200 | A15719 | 57 | 15 | 0.26 |
| | A12357 | A12550 | 951,472 | 115,727 | 0.12 | | A12360 | A12556 | 44 | 10 | 0.23 |
| | A12357 | A12944 | 900,051 | 109,650 | 0.12 | | A12360 | A12947 | 65 | 17 | 0.26 |
| | A12357 | A15716 | 888,549 | 108,622 | 0.12 | | A12360 | A15719 | 59 | 13 | 0.22 |
| | A12550 | A12944 | 785,946 | 95,928 | 0.12 | | A12556 | A12947 | 40 | 5 | 0.13 |
| | A12550 | A15716 | 766,463 | 93,566 | 0.12 | | A12556 | A15719 | 38 | 6 | 0.16 |
| | A12944 | A15716 | 728,129 | 89,263 | 0.12 | | A12947 | A15719 | 33 | 5 | 0.15 |
| BK_AA7_738 | A11198 | A12358 | 798 | 118 | 0.15 | BK_CC7_335 | A11201 | A12361 | 184,218 | 24,549 | 0.13 |
| | A11198 | A12554 | 645 | 82 | 0.13 | | A11201 | A12557 | 148,295 | 19,700 | 0.13 |
| | A11198 | A12945 | 650 | 71 | 0.11 | | A11201 | A12948 | 143,923 | 19,136 | 0.13 |
| | A11198 | A15717 | 413 | 45 | 0.11 | | A11201 | A15720 | 140,618 | 19,035 | 0.14 |
| | A12358 | A12554 | 605 | 75 | 0.12 | | A12361 | A12557 | 153,709 | 20,794 | 0.14 |
| | A12358 | A12945 | 636 | 80 | 0.13 | | A12361 | A12948 | 149,491 | 20,276 | 0.14 |
| | A12358 | A15717 | 408 | 56 | 0.14 | | A12361 | A15720 | 146,432 | 20,073 | 0.14 |
| | A12554 | A12945 | 508 | 54 | 0.11 | | A12557 | A12948 | 122,988 | 16,362 | 0.13 |
| | A12554 | A15717 | 321 | 42 | 0.13 | | A12557 | A15720 | 119,343 | 16,020 | 0.13 |
| | A12945 | A15717 | 313 | 39 | 0.12 | | A12948 | A15720 | 116,575 | 15,815 | 0.14 |
| BK_BB7_240 | A11199 | A12359 | 270,665 | 36,026 | 0.13 | BK_1653 | A11203 | A12363 | 825,158 | 94,016 | 0.11 |
| | A11199 | A12555 | 205,160 | 27,372 | 0.13 | | A11203 | A12562 | 661,181 | 75,081 | 0.11 |
| | A11199 | A12946 | 232,601 | 31,271 | 0.13 | | A11203 | A12950 | 635,860 | 72,355 | 0.11 |
| | A11199 | F9162 | 207,892 | 28,194 | 0.14 | | A11203 | A15722 | 623,297 | 72,535 | 0.12 |
| | A12359 | A12555 | 194,282 | 25,974 | 0.13 | | A12363 | A12562 | 659,532 | 75,433 | 0.11 |
| | A12359 | A12946 | 220,265 | 29,906 | 0.14 | | A12363 | A12950 | 633,979 | 72,743 | 0.11 |
| | A12359 | A15718 | 198,899 | 27,313 | 0.14 | | A12363 | A15722 | 617,845 | 71,759 | 0.12 |
| | A12555 | A12946 | 171,637 | 23,117 | 0.13 | | A12562 | A12950 | 522,808 | 59,664 | 0.11 |
| | A12555 | A15718 | 153,784 | 21,068 | 0.14 | | A12562 | A15722 | 509,476 | 59,031 | 0.12 |
| | A12946 | A15718 | 174,514 | 23,938 | 0.14 | | A12950 | A15722 | 493,663 | 57,293 | 0.12 |

# Supplementary Information 5

## Population relationships

### Principal Component Analysis (PCA)

As a first assessment of the genetic affinities of the studied individuals, we carried out Principal Component Analysis (PCA) using *smartpca* from the EIGENSOFT package[1,2]. We used 2,970 present-day humans genotyped on 597,573 SNPs of the Affymetrix Human Origins array[3] ([https://reich.hms.harvard.edu/downloadable-genotypes-present-day-and-ancient-dna-data-compiled-published-papers](https://reich.hms.harvard.edu/downloadable-genotypes-present-day-and-ancient-dna-data-compiled-published-papers), version V37.2, released February 2019) to estimate the eigenvectors. We then projected 22 ancient individuals older than 30,000 years before present (BP)[4-8] with more than 30,000 of the informative SNPs covered onto the plane defined by these eigenvectors using the 'lsqproject' option in *smartpca*[1] (Extended Data Fig. 2C). We further restricted constructing principle components to a set of 1,444 present-day Eurasians and Native Americans, and repeated the projection of 22 ancient individuals onto the plane defined by these eigenvectors (Extended Data Fig. 2D).

In the first PCA plot relating present-day African and non-African populations, IUP Bacho Kiro Cave individuals fall closer to present-day non-Africans than to Africans (Extended Data Fig. 2C). In the second PCA plot focused on present-day non-African populations, IUP Bacho Kiro Cave individuals fall close to the origin, similar to the ~45,000-year-old individual from Siberia (*Ust'Ishim*)[4] and the ~40,000-year-old individual from Romania (*Oase1*)[5] (Extended Data Fig. 2D). In contrast, Aurignacian individual from Bacho Kiro Cave, *BK1653*, forms a cluster with later Upper Palaeolithic (UP) individuals from Western Eurasia which is shifted away from the centre and towards present-day Western Eurasians in both PCA plots (Extended Data Fig. 2C and D).

### Outgroup f3-statistics

We computed the statistics of the form *f3(X, Y; Mbuti)*, which measures the amount of shared genetic drift between populations X and Y since their separation from an outgroup[3]. We calculated *f3(IUP BK, Y; Mbuti)* for the three IUP Bacho Kiro Cave individuals (Fig. 2A, Tab. S5.1, Fig. S5.1) and *f3(BK1653, Y; Mbuti)* (Tab. S5.5 and Extended Data Fig. 3B) as implemented in the R package *admixr*[9] (version: 0.7.1) and using 263 present-day individuals from the Simons Genome Diversity Project (SGDP)[10]. Three Mbuti individuals from the same panel were used as outgroup. Fig. 2A and Tab. S5.1 were calculated using a pool of the three

IUP Bacho Kiro individuals (*F6-620, BB7-240 and CC7-335*, nsnps = 1,813,821), whereas panels in the Fig. S5.1 and Tab. S5.2-S5.4. show the results for each individual separately.

We find that IUP Bacho Kiro individuals share more genetic drift with present-day populations from East Asia, Central Asia and the Americas (Fig. 2A, Fig. S5.1, Tab. S5.1-S5.4), similar to *Ust'Ishim*[4] (Fig. S5.2A) and *Oase1*[5] (Fig. S5.2B). In contrast, *BK1653* shares more genetic drift with present-day West Eurasians (Extended Data Fig. 3B, Tab. S5.5), similar to other UP individuals from Europe from ~38,000 years BP onwards[6,11] (Extended Data Fig. 3C).

We performed the genetic clustering of ancient modern humans using outgroup *$f_3$-statistics*[6], restricting this analysis to ancient individuals with at least 30,000 SNPs on the "2200k" Panel. We find that IUP Bacho Kiro individuals share more drift with each other than with other ancient humans (Extended Data Fig. 3A), whereas *BK1653* shares most drift with later UP individuals in Europe, specifically ~35,000-year-old *GoyetQ116-1* from Belgium and the members of the 'Vêstonice' genetic cluster[6] (Extended Data Fig. 3A).

**Relationship of Bacho Kiro individuals to present-day populations**

In order to investigate the relationship of Bacho Kiro Cave individuals to present-day humans in more detail, we used genotype calls of 263 present-day humans from the Simons Genome Diversity Project (SGDP)[10] overlapping "2200k" SNPs, and as described in the Supplementary Information 3. We used *D-statistics*[3] (version: v5.1) as implemented in the R package *admixr*[9] (version: 0.7.1) to infer the relationships among individuals, computing standard errors using a Weighted Block Jackknife[3,12] across all autosomes with equally sized blocks of 5 million base pairs (5 Mb). Guided by the results of the PCA and outgroup *$f_3$-statistics*, we first computed *D (African, non-African; Bacho Kiro Cave individual, Chimpanzee)* using genomes of present-day Mbuti, Yoruba, San and Dinka, as well as genomes of present-day non-Africans from SGDP[10], and the genome of a chimpanzee (*panTro2*) as outgroup. We find that all Bacho Kiro Cave individuals are significantly closer to present-day non-Africans than to present-day Africans ($|Z| > 41.49$, Fig. S5.3-S5.6).

We further explored whether Bacho Kiro Cave individuals are particularly close to a certain present-day population outside of Africa by calculating *D (non-African$_1$, non-African$_2$; Bacho Kiro Cave individual, Chimpanzee)* and *D (non-African$_1$, non-African$_2$; Bacho Kiro Cave individual, Mbuti)*. We find that IUP Bacho Kiro Cave individuals share significantly more alleles with present-day East Asians, Southeast Asians and Native Americans than with present-day Europeans (Fig. S5.7 and S5.8). However, present-day Europeans derive part of

their ancestry from a population that diverged from other non-Africans before they diverged from each other ('a Basal Eurasian' population)[13-15] and similar results are obtained when substituting IUP Bacho Kiro Cave individuals in these statistics with *Oase1*[5] or *Ust'Ishim*[4] (Fig. S5.9 and S5.10), both of whom are known not to have contributed their ancestry to later populations.

Thus, we replaced present-day Europeans in these statistics with Upper Palaeolithic[6,7,11] and Mesolithic[13] individuals that lack Basal Eurasian ancestry. Whereas in those instances, and with the additional data from *Oase 1*, both *Oase 1* and *Ust'Ishim* share equally many alleles with Europeans before the introduction of agriculture as with present-day East Asians and Native Americans[5], ($|Z| < 3$, Extended Data Fig. 4B and C), IUP Bacho Kiro Cave individuals continue to share significantly more alleles with present-day East Asians and Native Americans, and some Central Asians and Siberians from SGDP than with pre-agricultural Europeans such as the ~38,000-year-old *Kostenki 14* ($3.08 \leq |Z| \leq 5.32$, Extended Data Fig. 4A). These results remain stable after restricting the analyses to transversion polymorphisms which are not susceptible to errors due to aDNA modifications ($2.22 \leq Z \leq 3.92$, Fig. S5.13).

Given the slightly higher Neandertal ancestry in present-day East Asians than in West Eurasians[16,17] and the higher Neandertal ancestry in IUP Bacho Kiro individuals (see Supplementary Information 7 and 8), we substituted Mbuti as outgroup in *D(pre-agricultural European, non-African$_2$; IUP Bacho Kiro, Outgroup)* with the high coverage genome of *Vindija 33.19* Neandertal[17] to account for a possible attraction to East Asians due to the higher Neandertal ancestry. We find, however, that the results of these statistics remain significant regardless of the outgroup ($3.05 \leq Z \leq 5.90$, Fig. S5.14).

In contrast to the IUP Bacho Kiro Cave individuals, *BK1653* that lived ~11,000 years later[18,19] shares significantly more alleles with present-day Europeans than with any other superpopulation in SGDP[10] (Fig. S5.11 and S5.12), as it was previously found for other Upper Palaeolithic individuals from Western Eurasia starting with the ~38,000-year-old *Kostenki14* from Russia[6,11].

**Relationship of Bacho Kiro Cave individuals other ancient humans**

We computed *D(ancient$_1$, ancient$_2$; IUP Bacho Kiro, Mbuti)* and *D(ancient$_1$, IUP Bacho Kiro; ancient$_2$, Mbuti)* for a range of ancient modern humans using "1240k" and/or "2200k" SNP Panels, depending on the set of sites for which the data of previously published individuals are available (see Supplementary Information 3 for dataset details). By computing *D(IUP Bacho Kiro$_1$, IUP Bacho Kiro$_2$; X, Mbuti)* we find that the *IUP Bacho Kiro F6-620, BB7-240* and

*CC7-335* form a clade to exclusion of other ancient individuals (all |Z| <3, Tab. S5.8-S5.10). Since we were able to recover only 7,696 SNPs on the "2200k" Panel for the IUP *Bacho Kiro CC7-2289* (Extended Data Tab. 1, Supplementary Information 2, Tab. S2.8), we used *D(IUP Bacho Kiro*$_1$*, X; Bacho Kiro CC7-2289, Mbuti)* to explore broad population affiliations of this individual. We find, to the limits of our resolution, that she on average shares significantly more alleles with other IUP Bacho Kiro Cave individuals than with other ancient modern humans (Tab. S5.7). Furthermore, we find *D(IUP Bacho Kiro*$_1$*, IUP Bacho Kiro*$_2$*; IUP Bacho Kiro*$_3$*, Mbuti )*~ 0 for all combinations of IUP Bacho Kiro Cave individuals (Tab. S5.6).

Interestingly, when comparing IUP Bacho Kiro Cave individuals to other early Upper Palaeolithic humans from Eurasia, we find that they share significantly more alleles with the ~40,000-year-old *Tianyuan* individual from China[20] than with the ~38,000-year-old *Kostenki14* from west Russia (3.17 ≤ |Z| ≤ 4.21, Fig. 2B and C, Tab. S5.12-S5.14). Furthermore, IUP *Bacho Kiro F6-620* shares significantly more alleles with ancient Native Americans such as the ~13,000-year-old *Anzick*[21], ~11,000-year-old *Upward Sun River* and *Spirit Cave* individuals[22,23], the ~9,000-year-old *Kennewick*[24], and even with the ~4,000-year-old *Saqqaq*[25] than with *Kostenki14* (3.70 ≤ |Z| ≤ 4.72, Fig. 2C, Tab. S5.15). From other West Eurasian Upper Palaeolithic humans, IUP Bacho Kiro Cave individuals share significantly more derived alleles with *Oase1* (3.56 ≤ |Z| ≤ 4.26, Fig. 2C, Tab. S5.12-S5.14) and the ~35,000-year-old *Goyet Q116-1* from Belgium (3.24 ≤ |Z| ≤ 4.25, Fig. 2C, Tab. S5.12-S5.14) than with *Kostenki14*. We note, however, that the higher proportion of Neandertal ancestry (see Supplementary Information 7 and 8) could be driving the excess of alleles shared by the IUP Bacho Kiro Cave individuals and the *Oase1*. Alternatively, the IUP Bacho Kiro Cave individuals and *Oase1* could belong to related but not identical populations, given that they differ in their overall relationship to later populations.

We find that *Bacho Kiro BK1653* shares significantly more alleles with later West Eurasians than with the IUP Bacho Kiro Cave individuals by *D(Test, IUP Bacho Kiro; BK1653, Mbuti)* >> 0 (3.01 ≤ |Z| ≤ 17.39) where *Test* individual is any of the later West Eurasians starting with *Kostenki14*. Specifically, *Bacho Kiro BK1653* is closest to the *GoyetQ116-1* and to the members of the 'Vêstonice' genetic cluster, i.e. individuals that lived between ~34,000 and ~26,000 years ago and were associated with the Gravettian period. Moreover, *D(GoyetQ116-1, Vestonice16; BK1653, Mbuti), D(BK1653, GoyetQ116-1; Vestonice16, Mbuti)* and *D(BK1653, Vestonice16; GoyetQ116-1, Mbuti)* are all ~0 (all |Z| < 3, Tab. S5.11 and Tab. S5.15), indicating that *BK1653* shares equally many alleles with these individuals.

**References SI5:**


1 Patterson, N., Price, A. L. & Reich, D. Population structure and eigenanalysis. *PLoS Genet* **2**, e190, doi:10.1371/journal.pgen.0020190 (2006).

2 Price, A. L. *et al.* Principal components analysis corrects for stratification in genome-wide association studies. *Nat. Genet.* **38**, 904-909, doi: 10.1038/ng1847 (2006).

3 Patterson, N. *et al.* Ancient admixture in human history. *Genetics* **192**, 1065-1093, doi:10.1534/genetics.112.145037 (2012).

4 Fu, Q. *et al.* Genome sequence of a 45,000-year-old modern human from western Siberia. *Nature* **514**, 445-449, doi:10.1038/nature13810 (2014).

5 Fu, Q. *et al.* An early modern human from Romania with a recent Neanderthal ancestor. *Nature* **524**, 216-219, doi:10.1038/nature14558 (2015).

6 Fu, Q. *et al.* The genetic history of Ice Age Europe. *Nature* **534**, 200-205, doi:10.1038/nature17993 (2016).

7 Sikora, M. *et al.* Ancient genomes show social and reproductive behavior of early Upper Paleolithic foragers. *Science* **358**, 659-662, doi: 10.1126/science.aao1807 (2017).

8 Sikora, M. *et al.* The population history of northeastern Siberia since the Pleistocene. *Nature* **570**, 182-188, doi: 10.1038/s41586-019-1279-z (2019).

9 Petr, M., Vernot, B. & Kelso, J. admixr—R package for reproducible analyses using ADMIXTOOLS. *Bioinformatics* **35**, 3194-3195 (2019).

10 Mallick, S. *et al.* The Simons Genome Diversity Project: 300 genomes from 142 diverse populations. *Nature* **538**, 201-206, doi:10.1038/nature18964 (2016).

11 Seguin-Orlando, A. *et al.* Genomic structure in Europeans dating back at least 36,200 years. *Science* **346**, 1113-1118, doi:10.1126/science.aaa0114 (2014).

12 Busing, F. M. T. A., Meijer, E. & Van Der Leeden, R. Delete-m jackknife for unequal m. *Statistics and Computing* **9**, 3-8, doi:Doi 10.1023/A:1008800423698 (1999).

13 Lazaridis, I. *et al.* Ancient human genomes suggest three ancestral populations for present-day Europeans. *Nature* **513**, 409-413, doi:10.1038/nature13673 (2014).

14 Lazaridis, I. *et al.* Genomic insights into the origin of farming in the ancient Near East. *Nature* **536**, 419-424, doi: 10.1038/nature19310 (2016).

15 Lazaridis, I. *et al.* Paleolithic DNA from the Caucasus reveals core of West Eurasian ancestry. *bioRxiv*, 423079 (2018).

16 Wall, J. D. *et al.* Higher levels of neanderthal ancestry in East Asians than in Europeans. *Genetics* **194**, 199-209, doi:10.1534/genetics.112.148213 (2013).

17 Prüfer, K. *et al.* A high-coverage Neandertal genome from Vindija Cave in Croatia. *Science* **358**, 655-658, doi: 10.1126/science.aao1887 (2017).

18 Hublin, J.-J. et al. Initial Upper Palaeolithic Homo sapiens from Bacho Kiro Cave, Bulgaria. *Nature* **581**, 299-302, doi:10.1038/s41586-020-2259-z (2020).

19 Fewlass, H. et al. A 14 C chronology for the Middle to Upper Palaeolithic transition at Bacho Kiro Cave, Bulgaria. *Nat. Ecol. Evol.* **4**, 794-801, doi: 10.1038/s41559-020-1136-3 (2020).

20 Yang, M. A. *et al.* 40,000-Year-Old Individual from Asia Provides Insight into Early Population Structure in Eurasia. *Curr. Biol.* **27**, 3202-3208. e3209, doi: 10.1016/j.cub.2017.09.030 (2017).

21 Rasmussen, M. *et al.* The genome of a Late Pleistocene human from a Clovis burial site in western Montana. *Nature* **506**, 225-229, doi:10.1038/nature13025 (2014).

22 Moreno-Mayar, J. V. *et al.* Terminal Pleistocene Alaskan genome reveals first founding population of Native Americans. *Nature* **553**, 203-207, doi: 10.1038/nature25173 (2018).

23 Moreno-Mayar, J. V. *et al.* Early human dispersals within the Americas. *Science* **362**, eaav2621, doi:10.1126/science.aav2621 (2018).

24 Rasmussen, M. *et al.* The ancestry and affiliations of Kennewick Man. *Nature* **523**, 455-458, doi:10.1038/nature14625 (2015).

25 Rasmussen, M. *et al.* Ancient human genome sequence of an extinct Palaeo-Eskimo. *Nature* **463**, 757-762, doi:10.1038/nature08835 (2010).

**Table S5.1 Shared genetic drift between IUP Bacho Kiro Cave individuals and present-day humans as measured by *f3(IUP Bacho Kiro Cave individuals, present-day humans; Mbuti)*.** Present-day modern human genomes from SGDP[10] were used. *f3* values reported were calculated using ADMIXTOOLS[3] as implemented in *admixr*[9] and were used for plotting Fig. 2A in the main text. Standard errors (SE) were computed using a Weighted Block Jackknife[3,12] across all autosomes of the "2200k" Panel using a block size of 5Mb. Number of SNPs overlapping among individuals reported in the column "nsnps".

| A | B | C | f3 | SE | Zscore | nsnps |
|---|---|---|---|---|---|---|
| IUP_BKs | She | Mbuti | 0.2250 | 0.0034 | 65.94 | 1,241,880 |
| IUP_BKs | Japanese | Mbuti | 0.2250 | 0.0034 | 67.04 | 1,268,517 |
| IUP_BKs | Ami | Mbuti | 0.2240 | 0.0034 | 65.56 | 1,238,449 |
| IUP_BKs | Han | Mbuti | 0.2240 | 0.0034 | 65.87 | 1,244,308 |
| IUP_BKs | Yi | Mbuti | 0.2239 | 0.0034 | 65.67 | 1,245,099 |
| IUP_BKs | Kinh | Mbuti | 0.2236 | 0.0034 | 65.50 | 1,244,971 |
| IUP_BKs | Lahu | Mbuti | 0.2236 | 0.0034 | 66.48 | 1,241,407 |
| IUP_BKs | Oroqen | Mbuti | 0.2234 | 0.0034 | 65.97 | 1,242,864 |
| IUP_BKs | Tujia | Mbuti | 0.2234 | 0.0034 | 65.85 | 1,243,408 |
| IUP_BKs | Karitiana | Mbuti | 0.2234 | 0.0034 | 64.92 | 1,217,789 |
| IUP_BKs | Dai | Mbuti | 0.2234 | 0.0034 | 66.20 | 1,269,344 |
| IUP_BKs | Sherpa | Mbuti | 0.2233 | 0.0034 | 66.54 | 1,240,952 |
| IUP_BKs | Tibetan | Mbuti | 0.2231 | 0.0034 | 65.28 | 1,245,035 |
| IUP_BKs | Dusun | Mbuti | 0.2231 | 0.0034 | 65.90 | 1,239,008 |
| IUP_BKs | Eskimo_Naukan | Mbuti | 0.2230 | 0.0033 | 67.17 | 1,235,556 |
| IUP_BKs | Miao | Mbuti | 0.2230 | 0.0033 | 67.07 | 1,242,930 |
| IUP_BKs | Igorot | Mbuti | 0.2229 | 0.0034 | 64.82 | 1,235,592 |
| IUP_BKs | Naxi | Mbuti | 0.2229 | 0.0034 | 65.83 | 1,271,797 |
| IUP_BKs | Korean | Mbuti | 0.2228 | 0.0033 | 66.56 | 1,244,759 |
| IUP_BKs | Yakut | Mbuti | 0.2227 | 0.0035 | 63.87 | 1,245,519 |
| IUP_BKs | Ulchi | Mbuti | 0.2226 | 0.0033 | 67.12 | 1,242,300 |
| IUP_BKs | Onge | Mbuti | 0.2226 | 0.0033 | 66.85 | 1,228,377 |
| IUP_BKs | Eskimo_Chaplin | Mbuti | 0.2224 | 0.0034 | 65.25 | 1,196,996 |
| IUP_BKs | Atayal | Mbuti | 0.2222 | 0.0035 | 62.77 | 1,195,780 |
| IUP_BKs | Eskimo_Sireniki | Mbuti | 0.2222 | 0.0033 | 67.10 | 1,239,857 |
| IUP_BKs | Burmese | Mbuti | 0.2221 | 0.0033 | 66.75 | 1,249,617 |
| IUP_BKs | Xibo | Mbuti | 0.2220 | 0.0034 | 65.80 | 1,245,927 |
| IUP_BKs | Thai | Mbuti | 0.2219 | 0.0034 | 65.58 | 1,247,431 |
| IUP_BKs | Hezhen | Mbuti | 0.2219 | 0.0034 | 66.21 | 1,243,812 |
| IUP_BKs | Tu | Mbuti | 0.2218 | 0.0034 | 65.29 | 1,248,856 |
| IUP_BKs | Even | Mbuti | 0.2218 | 0.0034 | 65.33 | 1,272,478 |
| IUP_BKs | Chipewyan | Mbuti | 0.2217 | 0.0035 | 64.16 | 1,232,134 |
| IUP_BKs | Surui | Mbuti | 0.2216 | 0.0034 | 64.99 | 1,218,304 |
| IUP_BKs | Daur | Mbuti | 0.2216 | 0.0035 | 63.76 | 1,201,096 |
| IUP_BKs | Cambodian | Mbuti | 0.2215 | 0.0033 | 66.15 | 1,246,554 |
| IUP_BKs | Khonda_Dora | Mbuti | 0.2212 | 0.0034 | 64.56 | 1,199,909 |
| IUP_BKs | Mayan | Mbuti | 0.2210 | 0.0033 | 66.06 | 1,234,796 |
| IUP_BKs | Piapoco | Mbuti | 0.2208 | 0.0033 | 65.93 | 1,229,698 |
| IUP_BKs | Itelman | Mbuti | 0.2206 | 0.0035 | 63.60 | 1,198,070 |
| IUP_BKs | Kurumba | Mbuti | 0.2206 | 0.0034 | 65.20 | 1,199,474 |
| IUP_BKs | Botocudo | Mbuti | 0.2205 | 0.0034 | 64.56 | 1,111,320 |
| IUP_BKs | Zapotec | Mbuti | 0.2204 | 0.0033 | 66.20 | 1,237,121 |
| IUP_BKs | Chane | Mbuti | 0.2203 | 0.0034 | 65.07 | 1,196,463 |
| IUP_BKs | Mixe | Mbuti | 0.2202 | 0.0034 | 65.69 | 1,230,707 |
| IUP_BKs | Kharia | Mbuti | 0.2196 | 0.0034 | 64.47 | 1,200,711 |
| IUP_BKs | Kusunda | Mbuti | 0.2195 | 0.0033 | 66.63 | 1,239,127 |
| IUP_BKs | Nahua | Mbuti | 0.2193 | 0.0034 | 64.38 | 1,198,257 |
| IUP_BKs | Quechua | Mbuti | 0.2192 | 0.0033 | 66.56 | 1,255,945 |
| IUP_BKs | Bougainville | Mbuti | 0.2186 | 0.0034 | 64.98 | 1,232,440 |
| IUP_BKs | Altaian | Mbuti | 0.2185 | 0.0034 | 64.03 | 1,202,284 |
| IUP_BKs | Kyrgyz | Mbuti | 0.2184 | 0.0033 | 66.50 | 1,256,462 |
| IUP_BKs | Hawaiian | Mbuti | 0.2184 | 0.0035 | 63.13 | 1,199,752 |
| IUP_BKs | Pima | Mbuti | 0.2183 | 0.0034 | 64.41 | 1,229,699 |
| IUP_BKs | Cree | Mbuti | 0.2180 | 0.0033 | 66.79 | 1,251,803 |
| IUP_BKs | Aleut | Mbuti | 0.2171 | 0.0033 | 66.50 | 1,252,508 |
| IUP_BKs | Papuan | Mbuti | 0.2171 | 0.0034 | 64.18 | 1,300,909 |
| IUP_BKs | Australian | Mbuti | 0.2171 | 0.0034 | 64.01 | 1,246,350 |
| IUP_BKs | Yadava | Mbuti | 0.2170 | 0.0033 | 66.41 | 1,253,859 |
| IUP_BKs | Mala | Mbuti | 0.2168 | 0.0032 | 67.45 | 1,287,674 |
| IUP_BKs | Chukchi | Mbuti | 0.2161 | 0.0033 | 64.78 | 1,209,162 |
| IUP_BKs | Tubalar | Mbuti | 0.2161 | 0.0033 | 65.38 | 1,254,292 |
| IUP_BKs | Mansi | Mbuti | 0.2159 | 0.0033 | 65.33 | 1,256,117 |
| IUP_BKs | Madiga | Mbuti | 0.2157 | 0.0033 | 65.57 | 1,255,390 |
| IUP_BKs | Relli | Mbuti | 0.2154 | 0.0033 | 65.81 | 1,254,885 |
| IUP_BKs | Irula | Mbuti | 0.2153 | 0.0033 | 65.73 | 1,252,206 |
| IUP_BKs | Mixtec | Mbuti | 0.2152 | 0.0033 | 65.60 | 1,245,981 |
| IUP_BKs | Kapu | Mbuti | 0.2149 | 0.0033 | 64.35 | 1,256,970 |
| IUP_BKs | Hazara | Mbuti | 0.2149 | 0.0033 | 64.46 | 1,257,243 |
| IUP_BKs | Uygur | Mbuti | 0.2148 | 0.0032 | 66.33 | 1,261,144 |
| IUP_BKs | Bengali | Mbuti | 0.2144 | 0.0032 | 66.70 | 1,257,345 |
| IUP_BKs | Punjabi | Mbuti | 0.2137 | 0.0032 | 66.90 | 1,312,715 |
| IUP_BKs | Tlingit | Mbuti | 0.2133 | 0.0033 | 65.45 | 1,257,431 |
| IUP_BKs | Brahmin | Mbuti | 0.2125 | 0.0032 | 66.53 | 1,258,918 |
| IUP_BKs | Saami | Mbuti | 0.2116 | 0.0033 | 64.01 | 1,256,098 |
| IUP_BKs | Sindhi | Mbuti | 0.2115 | 0.0033 | 63.35 | 1,258,843 |
| IUP_BKs | Pathan | Mbuti | 0.2111 | 0.0032 | 65.29 | 1,260,590 |
| IUP_BKs | Burusho | Mbuti | 0.2110 | 0.0032 | 65.13 | 1,260,397 |
| IUP_BKs | Kashmiri_Pandit | Mbuti | 0.2107 | 0.0033 | 63.59 | 1,206,177 |
| IUP_BKs | Finnish | Mbuti | 0.2098 | 0.0032 | 66.10 | 1,292,610 |
| IUP_BKs | Kalash | Mbuti | 0.2095 | 0.0032 | 66.18 | 1,251,539 |
| IUP_BKs | Icelandic | Mbuti | 0.2095 | 0.0033 | 64.09 | 1,261,703 |
| IUP_BKs | Maori | Mbuti | 0.2093 | 0.0033 | 63.16 | 1,210,165 |
| IUP_BKs | Czech | Mbuti | 0.2093 | 0.0033 | 62.58 | 1,208,755 |
| IUP_BKs | Tajik | Mbuti | 0.2090 | 0.0032 | 64.67 | 1,258,992 |
| IUP_BKs | Estonian | Mbuti | 0.2090 | 0.0033 | 62.46 | 1,261,200 |
| IUP_BKs | Bergamo | Mbuti | 0.2088 | 0.0034 | 61.38 | 1,209,000 |
| IUP_BKs | Russian | Mbuti | 0.2087 | 0.0033 | 63.74 | 1,261,648 |
| IUP_BKs | French | Mbuti | 0.2080 | 0.0032 | 64.80 | 1,260,502 |
| IUP_BKs | Orcadian | Mbuti | 0.2080 | 0.0032 | 64.53 | 1,261,779 |
| IUP_BKs | English | Mbuti | 0.2077 | 0.0032 | 65.63 | 1,261,866 |
| IUP_BKs | Albanian | Mbuti | 0.2077 | 0.0033 | 63.88 | 1,208,168 |
| IUP_BKs | Hungarian | Mbuti | 0.2071 | 0.0033 | 62.86 | 1,262,301 |
| IUP_BKs | Brahui | Mbuti | 0.2070 | 0.0032 | 65.51 | 1,259,647 |
| IUP_BKs | Greek | Mbuti | 0.2070 | 0.0032 | 65.51 | 1,260,821 |
| IUP_BKs | Adygei | Mbuti | 0.2067 | 0.0032 | 64.85 | 1,261,156 |
| IUP_BKs | North_Ossetian | Mbuti | 0.2067 | 0.0032 | 64.72 | 1,261,983 |
| IUP_BKs | Abkhasian | Mbuti | 0.2067 | 0.0033 | 63.21 | 1,260,283 |
| IUP_BKs | Polish | Mbuti | 0.2065 | 0.0034 | 60.33 | 1,208,763 |
| IUP_BKs | Sardinian | Mbuti | 0.2063 | 0.0032 | 64.20 | 1,260,434 |
| IUP_BKs | Armenian | Mbuti | 0.2061 | 0.0033 | 63.30 | 1,233,323 |
| IUP_BKs | Bulgarian | Mbuti | 0.2059 | 0.0031 | 65.72 | 1,262,386 |
| IUP_BKs | Basque | Mbuti | 0.2059 | 0.0033 | 63.02 | 1,260,211 |
| IUP_BKs | Balochi | Mbuti | 0.2059 | 0.0034 | 60.92 | 1,202,820 |
| IUP_BKs | Iranian | Mbuti | 0.2056 | 0.0032 | 65.12 | 1,261,197 |
| IUP_BKs | Turkish | Mbuti | 0.2056 | 0.0033 | 63.00 | 1,261,498 |
| IUP_BKs | Georgian | Mbuti | 0.2055 | 0.0032 | 64.64 | 1,261,255 |
| IUP_BKs | Chechen | Mbuti | 0.2055 | 0.0033 | 62.35 | 1,208,291 |
| IUP_BKs | Lezgin | Mbuti | 0.2053 | 0.0032 | 64.31 | 1,261,140 |
| IUP_BKs | Spanish | Mbuti | 0.2052 | 0.0032 | 64.17 | 1,261,884 |
| IUP_BKs | Crete | Mbuti | 0.2050 | 0.0033 | 61.54 | 1,208,721 |
| IUP_BKs | Tuscan | Mbuti | 0.2049 | 0.0033 | 62.63 | 1,263,034 |
| IUP_BKs | Iraqi_Jew | Mbuti | 0.2024 | 0.0032 | 63.54 | 1,260,134 |
| IUP_BKs | Makrani | Mbuti | 0.2024 | 0.0032 | 63.98 | 1,259,368 |
| IUP_BKs | Druze | Mbuti | 0.2010 | 0.0031 | 64.31 | 1,260,987 |
| IUP_BKs | Samaritan | Mbuti | 0.1996 | 0.0032 | 62.18 | 1,202,831 |
| IUP_BKs | Yemenite_Jew | Mbuti | 0.1965 | 0.0031 | 62.83 | 1,260,131 |
| IUP_BKs | Jordanian | Mbuti | 0.1964 | 0.0031 | 63.22 | 1,296,397 |
| IUP_BKs | BedouinB | Mbuti | 0.1962 | 0.0031 | 63.58 | 1,255,127 |
| IUP_BKs | Palestinian | Mbuti | 0.1945 | 0.0031 | 62.31 | 1,299,657 |
| IUP_BKs | Saharawi | Mbuti | 0.1771 | 0.0030 | 59.31 | 1,270,496 |
| IUP_BKs | Mozabite | Mbuti | 0.1735 | 0.0030 | 57.64 | 1,271,305 |
| IUP_BKs | Somali | Mbuti | 0.1362 | 0.0030 | 45.71 | 1,204,964 |
| IUP_BKs | Masai | Mbuti | 0.1130 | 0.0026 | 44.20 | 1,270,410 |
| IUP_BKs | Dinka | Mbuti | 0.0918 | 0.0024 | 37.82 | 1,267,654 |
| IUP_BKs | Mandenka | Mbuti | 0.0879 | 0.0024 | 36.94 | 1,286,047 |
| IUP_BKs | Gambian | Mbuti | 0.0877 | 0.0023 | 37.87 | 1,285,227 |
| IUP_BKs | Luo | Mbuti | 0.0862 | 0.0023 | 37.45 | 1,279,191 |
| IUP_BKs | BantuKenya | Mbuti | 0.0853 | 0.0023 | 36.33 | 1,280,312 |
| IUP_BKs | Esan | Mbuti | 0.0838 | 0.0023 | 36.18 | 1,287,115 |
| IUP_BKs | Luhya | Mbuti | 0.0838 | 0.0024 | 35.56 | 1,279,996 |
| IUP_BKs | Yoruba | Mbuti | 0.0830 | 0.0023 | 36.78 | 1,288,387 |
| IUP_BKs | Igbo | Mbuti | 0.0819 | 0.0023 | 35.22 | 1,286,896 |
| IUP_BKs | Mende | Mbuti | 0.0818 | 0.0023 | 35.61 | 1,285,776 |
| IUP_BKs | Lemande | Mbuti | 0.0806 | 0.0023 | 35.42 | 1,283,720 |
| IUP_BKs | Kongo | Mbuti | 0.0794 | 0.0024 | 33.53 | 1,213,147 |
| IUP_BKs | BantuHerero | Mbuti | 0.0781 | 0.0023 | 34.47 | 1,280,684 |
| IUP_BKs | BantuTswana | Mbuti | 0.0730 | 0.0021 | 34.23 | 1,279,719 |
| IUP_BKs | Biaka | Mbuti | 0.0517 | 0.0019 | 27.08 | 1,259,090 |
| IUP_BKs | Ju_hoan_North | Mbuti | 0.0479 | 0.0018 | 25.88 | 1,284,494 |
| IUP_BKs | Khomani_San | Mbuti | 0.0451 | 0.0019 | 23.85 | 1,243,210 |

**Table S5.2 Shared genetic drift between *Bacho Kiro F6-620* and present-day humans as measured by *f3(F6-620, present-day humans; Mbuti)*.** Present-day modern human genomes from SGDP[10] were used. *f3* values reported were calculated using ADMIXTOOLS[3] as implemented in *admixr*[9] and were used for plotting Fig. S5.1A. Standard errors (SE) were computed using a Weighted Block Jackknife[3,12] across all autosomes using a block size of 5Mb. Number of SNPs overlapping among individuals reported in the column “nsnps”.

| A | B | C | f3 | SE | Zscore | nsnps |
|---|---|---|---|---|---|---|
| F6_620 | Japanese | Mbuti | 0.2248 | 0.0036 | 62.81 | 1,228,053 |
| F6_620 | She | Mbuti | 0.2247 | 0.0037 | 60.76 | 1,197,191 |
| F6_620 | Yi | Mbuti | 0.2235 | 0.0037 | 61.06 | 1,201,115 |
| F6_620 | Karitiana | Mbuti | 0.2235 | 0.0037 | 60.50 | 1,168,378 |
| F6_620 | Han | Mbuti | 0.2234 | 0.0036 | 61.33 | 1,200,062 |
| F6_620 | Kinh | Mbuti | 0.2232 | 0.0037 | 60.88 | 1,200,593 |
| F6_620 | Tujia | Mbuti | 0.2231 | 0.0036 | 61.52 | 1,198,922 |
| F6_620 | Dai | Mbuti | 0.2230 | 0.0036 | 62.01 | 1,228,660 |
| F6_620 | Sherpa | Mbuti | 0.2230 | 0.0036 | 62.22 | 1,196,177 |
| F6_620 | Tibetan | Mbuti | 0.2230 | 0.0037 | 60.80 | 1,200,705 |
| F6_620 | Dusun | Mbuti | 0.2229 | 0.0036 | 61.42 | 1,193,697 |
| F6_620 | Eskimo_Naukan | Mbuti | 0.2228 | 0.0035 | 63.08 | 1,189,590 |
| F6_620 | Ami | Mbuti | 0.2227 | 0.0037 | 60.34 | 1,193,155 |
| F6_620 | Oroqen | Mbuti | 0.2226 | 0.0036 | 61.15 | 1,198,307 |
| F6_620 | Igorot | Mbuti | 0.2225 | 0.0037 | 60.10 | 1,189,720 |
| F6_620 | Korean | Mbuti | 0.2225 | 0.0036 | 61.95 | 1,200,345 |
| F6_620 | Miao | Mbuti | 0.2224 | 0.0036 | 62.23 | 1,198,347 |
| F6_620 | Lahu | Mbuti | 0.2223 | 0.0036 | 61.85 | 1,196,775 |
| F6_620 | Yakut | Mbuti | 0.2223 | 0.0037 | 59.45 | 1,201,060 |
| F6_620 | Naxi | Mbuti | 0.2223 | 0.0036 | 61.57 | 1,231,524 |
| F6_620 | Eskimo_Sireniki | Mbuti | 0.2223 | 0.0036 | 62.56 | 1,194,560 |
| F6_620 | Onge | Mbuti | 0.2222 | 0.0036 | 62.48 | 1,181,660 |
| F6_620 | Eskimo_Chaplin | Mbuti | 0.2220 | 0.0036 | 60.94 | 1,144,393 |
| F6_620 | Ulchi | Mbuti | 0.2219 | 0.0036 | 62.47 | 1,197,616 |
| F6_620 | Xibo | Mbuti | 0.2216 | 0.0036 | 62.24 | 1,201,887 |
| F6_620 | Atayal | Mbuti | 0.2215 | 0.0038 | 57.77 | 1,143,068 |
| F6_620 | Chipewyan | Mbuti | 0.2214 | 0.0037 | 59.67 | 1,185,370 |
| F6_620 | Burmese | Mbuti | 0.2213 | 0.0035 | 62.62 | 1,206,044 |
| F6_620 | Hezhen | Mbuti | 0.2210 | 0.0036 | 61.73 | 1,199,172 |
| F6_620 | Tu | Mbuti | 0.2210 | 0.0036 | 60.62 | 1,205,016 |
| F6_620 | Thai | Mbuti | 0.2210 | 0.0036 | 61.08 | 1,203,523 |
| F6_620 | Surui | Mbuti | 0.2210 | 0.0037 | 60.21 | 1,169,153 |
| F6_620 | Piapoco | Mbuti | 0.2207 | 0.0036 | 61.11 | 1,182,349 |
| F6_620 | Mayan | Mbuti | 0.2206 | 0.0036 | 61.28 | 1,188,183 |
| F6_620 | Kurumba | Mbuti | 0.2204 | 0.0037 | 59.85 | 1,147,705 |
| F6_620 | Daur | Mbuti | 0.2204 | 0.0037 | 59.41 | 1,149,476 |
| F6_620 | Even | Mbuti | 0.2204 | 0.0036 | 60.82 | 1,232,019 |
| F6_620 | Khonda_Dora | Mbuti | 0.2202 | 0.0037 | 59.21 | 1,148,262 |
| F6_620 | Chane | Mbuti | 0.2201 | 0.0037 | 59.86 | 1,143,287 |
| F6_620 | Mixe | Mbuti | 0.2201 | 0.0036 | 61.25 | 1,183,413 |
| F6_620 | Zapotec | Mbuti | 0.2199 | 0.0036 | 61.87 | 1,190,997 |
| F6_620 | Cambodian | Mbuti | 0.2197 | 0.0036 | 61.51 | 1,202,894 |
| F6_620 | Nahua | Mbuti | 0.2196 | 0.0037 | 59.82 | 1,145,281 |
| F6_620 | Botocudo | Mbuti | 0.2195 | 0.0037 | 59.77 | 1,059,982 |
| F6_620 | Itelman | Mbuti | 0.2190 | 0.0037 | 58.78 | 1,145,492 |
| F6_620 | Quechua | Mbuti | 0.2190 | 0.0035 | 61.89 | 1,212,610 |
| F6_620 | Kharia | Mbuti | 0.2186 | 0.0037 | 59.77 | 1,149,294 |
| F6_620 | Bougainville | Mbuti | 0.2182 | 0.0037 | 58.88 | 1,185,863 |
| F6_620 | Pima | Mbuti | 0.2180 | 0.0036 | 60.17 | 1,182,167 |
| F6_620 | Altaian | Mbuti | 0.2180 | 0.0036 | 59.91 | 1,150,627 |
| F6_620 | Kusunda | Mbuti | 0.2179 | 0.0035 | 61.77 | 1,194,069 |
| F6_620 | Kyrgyz | Mbuti | 0.2175 | 0.0035 | 62.37 | 1,213,289 |
| F6_620 | Hawaiian | Mbuti | 0.2173 | 0.0038 | 57.76 | 1,147,586 |
| F6_620 | Cree | Mbuti | 0.2170 | 0.0035 | 62.53 | 1,207,557 |
| F6_620 | Papuan | Mbuti | 0.2168 | 0.0037 | 59.37 | 1,263,180 |
| F6_620 | Australian | Mbuti | 0.2166 | 0.0037 | 59.23 | 1,202,072 |
| F6_620 | Aleut | Mbuti | 0.2165 | 0.0035 | 62.43 | 1,208,644 |
| F6_620 | Yadava | Mbuti | 0.2160 | 0.0035 | 61.76 | 1,210,425 |
| F6_620 | Mala | Mbuti | 0.2159 | 0.0034 | 62.85 | 1,248,835 |
| F6_620 | Mansi | Mbuti | 0.2156 | 0.0035 | 61.28 | 1,212,531 |
| F6_620 | Chukchi | Mbuti | 0.2149 | 0.0036 | 59.85 | 1,157,925 |
| F6_620 | Relli | Mbuti | 0.2148 | 0.0035 | 61.23 | 1,211,822 |
| F6_620 | Tubalar | Mbuti | 0.2147 | 0.0035 | 61.12 | 1,210,720 |
| F6_620 | Mixtec | Mbuti | 0.2145 | 0.0035 | 61.48 | 1,200,846 |
| F6_620 | Madiga | Mbuti | 0.2143 | 0.0035 | 61.41 | 1,212,463 |
| F6_620 | Uygur | Mbuti | 0.2142 | 0.0034 | 62.96 | 1,218,184 |
| F6_620 | Irula | Mbuti | 0.2142 | 0.0035 | 60.68 | 1,208,825 |
| F6_620 | Kapu | Mbuti | 0.2137 | 0.0036 | 59.67 | 1,213,956 |
| F6_620 | Hazara | Mbuti | 0.2136 | 0.0035 | 60.24 | 1,213,978 |
| F6_620 | Bengali | Mbuti | 0.2131 | 0.0034 | 62.41 | 1,214,446 |
| F6_620 | Punjabi | Mbuti | 0.2124 | 0.0034 | 63.00 | 1,276,307 |
| F6_620 | Tlingit | Mbuti | 0.2123 | 0.0034 | 61.88 | 1,213,542 |
| F6_620 | Brahmin | Mbuti | 0.2116 | 0.0034 | 62.37 | 1,215,848 |
| F6_620 | Saami | Mbuti | 0.2105 | 0.0035 | 59.79 | 1,211,942 |
| F6_620 | Sindhi | Mbuti | 0.2101 | 0.0035 | 59.53 | 1,215,486 |
| F6_620 | Pathan | Mbuti | 0.2100 | 0.0035 | 60.78 | 1,217,387 |
| F6_620 | Kashmiri_Pandit | Mbuti | 0.2096 | 0.0036 | 58.36 | 1,154,404 |
| F6_620 | Burusho | Mbuti | 0.2094 | 0.0034 | 60.84 | 1,217,354 |
| F6_620 | Finnish | Mbuti | 0.2090 | 0.0033 | 62.39 | 1,252,667 |
| F6_620 | Kalash | Mbuti | 0.2087 | 0.0034 | 62.15 | 1,206,735 |
| F6_620 | Maori | Mbuti | 0.2084 | 0.0036 | 57.58 | 1,158,963 |
| F6_620 | Icelandic | Mbuti | 0.2080 | 0.0035 | 60.26 | 1,217,708 |
| F6_620 | Bergamo | Mbuti | 0.2080 | 0.0036 | 57.50 | 1,156,648 |
| F6_620 | Tajik | Mbuti | 0.2078 | 0.0034 | 60.40 | 1,214,934 |
| F6_620 | Estonian | Mbuti | 0.2076 | 0.0035 | 58.77 | 1,217,041 |
| F6_620 | Czech | Mbuti | 0.2074 | 0.0036 | 58.01 | 1,156,831 |
| F6_620 | Russian | Mbuti | 0.2071 | 0.0035 | 59.55 | 1,217,844 |
| F6_620 | English | Mbuti | 0.2069 | 0.0034 | 61.27 | 1,217,798 |
| F6_620 | French | Mbuti | 0.2068 | 0.0034 | 61.17 | 1,216,429 |
| F6_620 | Albanian | Mbuti | 0.2066 | 0.0035 | 59.19 | 1,155,708 |
| F6_620 | Orcadian | Mbuti | 0.2063 | 0.0034 | 60.55 | 1,217,886 |
| F6_620 | Brahui | Mbuti | 0.2061 | 0.0033 | 62.06 | 1,215,558 |
| F6_620 | Abkhasian | Mbuti | 0.2060 | 0.0035 | 59.31 | 1,215,995 |
| F6_620 | Hungarian | Mbuti | 0.2060 | 0.0035 | 59.17 | 1,218,236 |
| F6_620 | North_Ossetian | Mbuti | 0.2054 | 0.0034 | 60.41 | 1,217,940 |
| F6_620 | Greek | Mbuti | 0.2054 | 0.0033 | 61.71 | 1,216,650 |
| F6_620 | Adygei | Mbuti | 0.2053 | 0.0034 | 60.15 | 1,217,032 |
| F6_620 | Iranian | Mbuti | 0.2052 | 0.0033 | 61.46 | 1,217,134 |
| F6_620 | Armenian | Mbuti | 0.2051 | 0.0035 | 58.82 | 1,185,190 |
| F6_620 | Polish | Mbuti | 0.2050 | 0.0037 | 55.48 | 1,156,609 |
| F6_620 | Georgian | Mbuti | 0.2049 | 0.0034 | 60.83 | 1,216,976 |
| F6_620 | Sardinian | Mbuti | 0.2048 | 0.0034 | 59.42 | 1,215,977 |
| F6_620 | Balochi | Mbuti | 0.2046 | 0.0036 | 57.22 | 1,149,984 |
| F6_620 | Bulgarian | Mbuti | 0.2044 | 0.0033 | 62.42 | 1,218,484 |
| F6_620 | Lezgin | Mbuti | 0.2043 | 0.0034 | 60.45 | 1,216,833 |
| F6_620 | Crete | Mbuti | 0.2042 | 0.0035 | 57.78 | 1,156,472 |
| F6_620 | Turkish | Mbuti | 0.2042 | 0.0034 | 59.39 | 1,217,458 |
| F6_620 | Chechen | Mbuti | 0.2041 | 0.0035 | 58.25 | 1,156,214 |
| F6_620 | Basque | Mbuti | 0.2040 | 0.0035 | 58.52 | 1,215,909 |
| F6_620 | Spanish | Mbuti | 0.2037 | 0.0034 | 60.01 | 1,217,634 |
| F6_620 | Tuscan | Mbuti | 0.2032 | 0.0035 | 58.17 | 1,218,914 |
| F6_620 | Iraqi_Jew | Mbuti | 0.2015 | 0.0034 | 58.86 | 1,215,640 |
| F6_620 | Makrani | Mbuti | 0.2012 | 0.0033 | 60.20 | 1,214,842 |
| F6_620 | Druze | Mbuti | 0.2002 | 0.0033 | 60.12 | 1,216,365 |
| F6_620 | Samaritan | Mbuti | 0.1978 | 0.0035 | 57.32 | 1,149,203 |
| F6_620 | Yemenite_Jew | Mbuti | 0.1956 | 0.0033 | 59.10 | 1,215,229 |
| F6_620 | Jordanian | Mbuti | 0.1955 | 0.0033 | 59.80 | 1,255,736 |
| F6_620 | BedouinB | Mbuti | 0.1945 | 0.0033 | 59.45 | 1,209,568 |
| F6_620 | Palestinian | Mbuti | 0.1931 | 0.0033 | 58.64 | 1,259,275 |
| F6_620 | Saharawi | Mbuti | 0.1761 | 0.0032 | 55.63 | 1,224,879 |
| F6_620 | Mozabite | Mbuti | 0.1718 | 0.0032 | 54.29 | 1,225,170 |
| F6_620 | Somali | Mbuti | 0.1357 | 0.0031 | 43.32 | 1,147,736 |
| F6_620 | Masai | Mbuti | 0.1128 | 0.0026 | 42.69 | 1,219,956 |
| F6_620 | Dinka | Mbuti | 0.0917 | 0.0025 | 36.39 | 1,214,506 |
| F6_620 | Mandenka | Mbuti | 0.0878 | 0.0025 | 35.51 | 1,232,569 |
| F6_620 | Gambian | Mbuti | 0.0876 | 0.0024 | 36.70 | 1,231,938 |
| F6_620 | Luo | Mbuti | 0.0858 | 0.0024 | 36.50 | 1,226,077 |
| F6_620 | BantuKenya | Mbuti | 0.0850 | 0.0024 | 35.02 | 1,227,124 |
| F6_620 | Esan | Mbuti | 0.0839 | 0.0024 | 35.13 | 1,233,222 |
| F6_620 | Luhya | Mbuti | 0.0831 | 0.0024 | 34.36 | 1,226,634 |
| F6_620 | Yoruba | Mbuti | 0.0825 | 0.0023 | 35.38 | 1,234,861 |
| F6_620 | Mende | Mbuti | 0.0814 | 0.0024 | 34.17 | 1,232,278 |
| F6_620 | Igbo | Mbuti | 0.0814 | 0.0024 | 33.87 | 1,233,141 |
| F6_620 | Lemande | Mbuti | 0.0800 | 0.0023 | 34.17 | 1,230,077 |
| F6_620 | Kongo | Mbuti | 0.0793 | 0.0025 | 31.84 | 1,152,001 |
| F6_620 | BantuHerero | Mbuti | 0.0775 | 0.0023 | 33.05 | 1,226,363 |
| F6_620 | BantuTswana | Mbuti | 0.0730 | 0.0022 | 32.97 | 1,225,213 |
| F6_620 | Biaka | Mbuti | 0.0512 | 0.0020 | 25.97 | 1,202,579 |
| F6_620 | Ju_hoan_North | Mbuti | 0.0480 | 0.0019 | 24.88 | 1,229,370 |
| F6_620 | Khomani_San | Mbuti | 0.0449 | 0.0020 | 22.79 | 1,184,667 |

**Table S5.3 Shared genetic drift between *Bacho Kiro BB7-240* and present-day humans as measured by *f3(BB7-240, present-day humans; Mbuti)*.** Present-day modern human genomes from SGDP[10] were used. *f3* values reported were calculated using ADMIXTOOLS[3] as implemented in *admixr*[9] and were used for plotting Fig. S5.1B. Standard errors (SE) were computed using a Weighted Block Jackknife[3,12] across all autosomes using a block size of 5Mb. Number of SNPs overlapping among individuals reported in the column "nsnps".

| A | B | C | f3 | SE | Zscore | nsnps |
|---|---|---|---|---|---|---|
| BB7_240 | She | Mbuti | 0.2286 | 0.0035 | 64.70 | 532,598 |
| BB7_240 | Ami | Mbuti | 0.2280 | 0.0037 | 62.47 | 530,941 |
| BB7_240 | Lahu | Mbuti | 0.2272 | 0.0036 | 62.33 | 532,013 |
| BB7_240 | Kinh | Mbuti | 0.2265 | 0.0036 | 62.69 | 533,949 |
| BB7_240 | Yi | Mbuti | 0.2265 | 0.0036 | 62.95 | 534,375 |
| BB7_240 | Japanese | Mbuti | 0.2262 | 0.0035 | 64.07 | 546,821 |
| BB7_240 | Miao | Mbuti | 0.2262 | 0.0036 | 63.43 | 533,135 |
| BB7_240 | Cambodian | Mbuti | 0.2260 | 0.0035 | 64.29 | 534,583 |
| BB7_240 | Igorot | Mbuti | 0.2259 | 0.0037 | 61.53 | 529,140 |
| BB7_240 | Han | Mbuti | 0.2258 | 0.0036 | 62.94 | 533,885 |
| BB7_240 | Tujia | Mbuti | 0.2257 | 0.0037 | 61.65 | 533,432 |
| BB7_240 | Naxi | Mbuti | 0.2255 | 0.0036 | 62.84 | 548,346 |
| BB7_240 | Dai | Mbuti | 0.2255 | 0.0036 | 62.12 | 546,838 |
| BB7_240 | Atayal | Mbuti | 0.2254 | 0.0038 | 59.55 | 507,848 |
| BB7_240 | Even | Mbuti | 0.2252 | 0.0036 | 63.44 | 548,278 |
| BB7_240 | Sherpa | Mbuti | 0.2252 | 0.0036 | 62.53 | 531,777 |
| BB7_240 | Xibo | Mbuti | 0.2251 | 0.0037 | 61.68 | 534,906 |
| BB7_240 | Oroqen | Mbuti | 0.2251 | 0.0035 | 63.50 | 532,877 |
| BB7_240 | Yakut | Mbuti | 0.2248 | 0.0037 | 61.13 | 534,349 |
| BB7_240 | Ulchi | Mbuti | 0.2246 | 0.0036 | 62.76 | 532,736 |
| BB7_240 | Thai | Mbuti | 0.2244 | 0.0036 | 62.33 | 535,488 |
| BB7_240 | Korean | Mbuti | 0.2243 | 0.0035 | 63.42 | 534,120 |
| BB7_240 | Hezhen | Mbuti | 0.2242 | 0.0036 | 62.60 | 533,450 |
| BB7_240 | Karitiana | Mbuti | 0.2239 | 0.0037 | 60.60 | 519,356 |
| BB7_240 | Kusunda | Mbuti | 0.2239 | 0.0035 | 64.14 | 530,584 |
| BB7_240 | Dusun | Mbuti | 0.2238 | 0.0036 | 62.06 | 531,211 |
| BB7_240 | Eskimo_Chaplin | Mbuti | 0.2238 | 0.0037 | 60.50 | 508,117 |
| BB7_240 | Surui | Mbuti | 0.2238 | 0.0037 | 60.74 | 519,448 |
| BB7_240 | Tibetan | Mbuti | 0.2238 | 0.0036 | 61.72 | 534,575 |
| BB7_240 | Tu | Mbuti | 0.2236 | 0.0035 | 63.27 | 535,878 |
| BB7_240 | Daur | Mbuti | 0.2236 | 0.0037 | 60.30 | 510,506 |
| BB7_240 | Burmese | Mbuti | 0.2236 | 0.0035 | 64.01 | 536,295 |
| BB7_240 | Mayan | Mbuti | 0.2236 | 0.0035 | 63.40 | 528,227 |
| BB7_240 | Chipewyan | Mbuti | 0.2236 | 0.0036 | 62.13 | 526,818 |
| BB7_240 | Khonda_Dora | Mbuti | 0.2236 | 0.0037 | 60.71 | 510,255 |
| BB7_240 | Eskimo_Sireniki | Mbuti | 0.2235 | 0.0036 | 62.76 | 531,205 |
| BB7_240 | Onge | Mbuti | 0.2233 | 0.0037 | 60.74 | 525,377 |
| BB7_240 | Kharia | Mbuti | 0.2232 | 0.0037 | 60.38 | 510,489 |
| BB7_240 | Piapoco | Mbuti | 0.2230 | 0.0036 | 62.35 | 525,552 |
| BB7_240 | Botocudo | Mbuti | 0.2229 | 0.0037 | 60.19 | 481,609 |
| BB7_240 | Zapotec | Mbuti | 0.2228 | 0.0035 | 62.88 | 529,419 |
| BB7_240 | Eskimo_Naukan | Mbuti | 0.2228 | 0.0036 | 62.62 | 528,993 |
| BB7_240 | Itelman | Mbuti | 0.2225 | 0.0037 | 59.88 | 508,865 |
| BB7_240 | Hawaiian | Mbuti | 0.2218 | 0.0036 | 61.44 | 509,840 |
| BB7_240 | Chane | Mbuti | 0.2216 | 0.0038 | 59.10 | 507,933 |
| BB7_240 | Mixe | Mbuti | 0.2214 | 0.0036 | 61.76 | 525,871 |
| BB7_240 | Quechua | Mbuti | 0.2214 | 0.0035 | 62.43 | 539,236 |
| BB7_240 | Altaian | Mbuti | 0.2213 | 0.0037 | 59.32 | 511,563 |
| BB7_240 | Kurumba | Mbuti | 0.2212 | 0.0036 | 61.24 | 509,836 |
| BB7_240 | Kyrgyz | Mbuti | 0.2210 | 0.0035 | 63.18 | 539,507 |
| BB7_240 | Australian | Mbuti | 0.2209 | 0.0037 | 59.53 | 534,341 |
| BB7_240 | Nahua | Mbuti | 0.2208 | 0.0036 | 61.25 | 508,891 |
| BB7_240 | Pima | Mbuti | 0.2208 | 0.0036 | 61.29 | 525,609 |
| BB7_240 | Cree | Mbuti | 0.2206 | 0.0035 | 62.68 | 537,258 |
| BB7_240 | Tubalar | Mbuti | 0.2205 | 0.0035 | 62.52 | 538,596 |
| BB7_240 | Mala | Mbuti | 0.2194 | 0.0034 | 64.69 | 555,568 |
| BB7_240 | Kapu | Mbuti | 0.2192 | 0.0035 | 62.36 | 539,529 |
| BB7_240 | Papuan | Mbuti | 0.2190 | 0.0037 | 59.85 | 562,780 |
| BB7_240 | Mansi | Mbuti | 0.2189 | 0.0035 | 61.96 | 539,466 |
| BB7_240 | Madiga | Mbuti | 0.2188 | 0.0035 | 62.68 | 539,210 |
| BB7_240 | Aleut | Mbuti | 0.2188 | 0.0035 | 62.03 | 537,617 |
| BB7_240 | Hazara | Mbuti | 0.2186 | 0.0035 | 62.77 | 539,936 |
| BB7_240 | Bougainville | Mbuti | 0.2186 | 0.0036 | 61.20 | 527,796 |
| BB7_240 | Irula | Mbuti | 0.2185 | 0.0034 | 63.45 | 537,545 |
| BB7_240 | Yadava | Mbuti | 0.2185 | 0.0034 | 63.64 | 538,491 |
| BB7_240 | Chukchi | Mbuti | 0.2184 | 0.0035 | 61.68 | 514,377 |
| BB7_240 | Mixtec | Mbuti | 0.2175 | 0.0035 | 62.58 | 534,136 |
| BB7_240 | Relli | Mbuti | 0.2172 | 0.0034 | 63.44 | 538,987 |
| BB7_240 | Bengali | Mbuti | 0.2171 | 0.0034 | 63.32 | 540,042 |
| BB7_240 | Uygur | Mbuti | 0.2167 | 0.0035 | 61.07 | 542,257 |

| A | B | C | f3 | SE | Zscore | nsnps |
|---|---|---|---|---|---|---|
| BB7_240 | Tlingit | Mbuti | 0.2167 | 0.0035 | 62.57 | 540,128 |
| BB7_240 | Punjabi | Mbuti | 0.2164 | 0.0034 | 63.89 | 568,585 |
| BB7_240 | Pathan | Mbuti | 0.2151 | 0.0034 | 62.67 | 541,520 |
| BB7_240 | Brahmin | Mbuti | 0.2150 | 0.0034 | 63.13 | 540,905 |
| BB7_240 | Burusho | Mbuti | 0.2147 | 0.0034 | 62.59 | 541,528 |
| BB7_240 | Sindhi | Mbuti | 0.2143 | 0.0035 | 61.50 | 540,375 |
| BB7_240 | Saami | Mbuti | 0.2141 | 0.0035 | 60.60 | 539,338 |
| BB7_240 | Czech | Mbuti | 0.2141 | 0.0036 | 59.81 | 513,798 |
| BB7_240 | Icelandic | Mbuti | 0.2132 | 0.0035 | 60.75 | 541,774 |
| BB7_240 | Kashmiri_Pandit | Mbuti | 0.2127 | 0.0035 | 60.30 | 512,650 |
| BB7_240 | Russian | Mbuti | 0.2124 | 0.0034 | 62.23 | 541,749 |
| BB7_240 | Tajik | Mbuti | 0.2122 | 0.0035 | 61.28 | 540,555 |
| BB7_240 | Kalash | Mbuti | 0.2121 | 0.0034 | 61.88 | 536,812 |
| BB7_240 | Finnish | Mbuti | 0.2112 | 0.0034 | 62.80 | 558,414 |
| BB7_240 | Orcadian | Mbuti | 0.2112 | 0.0035 | 60.95 | 542,056 |
| BB7_240 | Maori | Mbuti | 0.2111 | 0.0034 | 62.83 | 515,289 |
| BB7_240 | French | Mbuti | 0.2110 | 0.0034 | 62.27 | 541,241 |
| BB7_240 | Bergamo | Mbuti | 0.2109 | 0.0036 | 59.23 | 513,789 |
| BB7_240 | Estonian | Mbuti | 0.2108 | 0.0035 | 59.94 | 541,818 |
| BB7_240 | Greek | Mbuti | 0.2107 | 0.0034 | 62.23 | 541,395 |
| BB7_240 | Albanian | Mbuti | 0.2100 | 0.0035 | 60.47 | 513,904 |
| BB7_240 | North_Ossetian | Mbuti | 0.2095 | 0.0033 | 62.79 | 541,984 |
| BB7_240 | English | Mbuti | 0.2095 | 0.0034 | 62.46 | 541,896 |
| BB7_240 | Polish | Mbuti | 0.2094 | 0.0035 | 59.38 | 514,290 |
| BB7_240 | Basque | Mbuti | 0.2093 | 0.0034 | 61.10 | 541,124 |
| BB7_240 | Adygei | Mbuti | 0.2091 | 0.0033 | 63.40 | 541,777 |
| BB7_240 | Brahui | Mbuti | 0.2091 | 0.0034 | 62.08 | 540,608 |
| BB7_240 | Hungarian | Mbuti | 0.2090 | 0.0034 | 61.16 | 542,362 |
| BB7_240 | Sardinian | Mbuti | 0.2086 | 0.0033 | 62.55 | 540,702 |
| BB7_240 | Balochi | Mbuti | 0.2086 | 0.0036 | 57.35 | 510,826 |
| BB7_240 | Tuscan | Mbuti | 0.2085 | 0.0034 | 60.75 | 542,235 |
| BB7_240 | Bulgarian | Mbuti | 0.2082 | 0.0034 | 60.36 | 542,292 |
| BB7_240 | Turkish | Mbuti | 0.2079 | 0.0034 | 60.97 | 541,696 |
| BB7_240 | Spanish | Mbuti | 0.2079 | 0.0034 | 61.08 | 541,883 |
| BB7_240 | Abkhasian | Mbuti | 0.2075 | 0.0034 | 60.32 | 541,142 |
| BB7_240 | Lezgin | Mbuti | 0.2074 | 0.0034 | 61.72 | 541,771 |
| BB7_240 | Chechen | Mbuti | 0.2073 | 0.0035 | 59.06 | 513,663 |
| BB7_240 | Crete | Mbuti | 0.2066 | 0.0036 | 58.05 | 513,898 |
| BB7_240 | Georgian | Mbuti | 0.2064 | 0.0034 | 61.50 | 541,603 |
| BB7_240 | Armenian | Mbuti | 0.2063 | 0.0034 | 60.61 | 526,973 |
| BB7_240 | Iranian | Mbuti | 0.2047 | 0.0034 | 60.78 | 541,863 |
| BB7_240 | Makrani | Mbuti | 0.2046 | 0.0034 | 61.05 | 540,505 |
| BB7_240 | Iraqi_Jew | Mbuti | 0.2031 | 0.0033 | 62.44 | 541,085 |
| BB7_240 | Druze | Mbuti | 0.2013 | 0.0033 | 60.95 | 541,580 |
| BB7_240 | Samaritan | Mbuti | 0.2012 | 0.0035 | 57.90 | 510,297 |
| BB7_240 | BedouinB | Mbuti | 0.1999 | 0.0033 | 60.60 | 537,914 |
| BB7_240 | Yemenite_Jew | Mbuti | 0.1980 | 0.0034 | 58.47 | 540,581 |
| BB7_240 | Palestinian | Mbuti | 0.1979 | 0.0033 | 59.95 | 560,334 |
| BB7_240 | Jordanian | Mbuti | 0.1979 | 0.0033 | 59.69 | 558,898 |
| BB7_240 | Saharawi | Mbuti | 0.1793 | 0.0032 | 56.54 | 544,487 |
| BB7_240 | Mozabite | Mbuti | 0.1766 | 0.0032 | 55.40 | 544,187 |
| BB7_240 | Somali | Mbuti | 0.1358 | 0.0031 | 43.87 | 509,538 |
| BB7_240 | Masai | Mbuti | 0.1127 | 0.0026 | 42.72 | 541,153 |
| BB7_240 | Dinka | Mbuti | 0.0917 | 0.0025 | 36.50 | 537,784 |
| BB7_240 | Mandenka | Mbuti | 0.0878 | 0.0025 | 35.18 | 545,374 |
| BB7_240 | Gambian | Mbuti | 0.0871 | 0.0024 | 36.08 | 544,833 |
| BB7_240 | Luo | Mbuti | 0.0867 | 0.0025 | 34.61 | 542,610 |
| BB7_240 | BantuKenya | Mbuti | 0.0854 | 0.0025 | 34.85 | 542,758 |
| BB7_240 | Luhya | Mbuti | 0.0842 | 0.0024 | 34.88 | 542,410 |
| BB7_240 | Yoruba | Mbuti | 0.0839 | 0.0024 | 35.30 | 546,023 |
| BB7_240 | Esan | Mbuti | 0.0832 | 0.0024 | 34.42 | 545,266 |
| BB7_240 | Igbo | Mbuti | 0.0821 | 0.0025 | 33.42 | 544,689 |
| BB7_240 | Mende | Mbuti | 0.0818 | 0.0024 | 33.54 | 544,878 |
| BB7_240 | Lemande | Mbuti | 0.0809 | 0.0024 | 33.07 | 543,905 |
| BB7_240 | Kongo | Mbuti | 0.0804 | 0.0025 | 32.18 | 509,798 |
| BB7_240 | BantuHerero | Mbuti | 0.0781 | 0.0024 | 33.03 | 542,214 |
| BB7_240 | BantuTswana | Mbuti | 0.0725 | 0.0022 | 32.28 | 542,733 |
| BB7_240 | Biaka | Mbuti | 0.0522 | 0.0020 | 26.08 | 532,391 |
| BB7_240 | Ju_hoan_North | Mbuti | 0.0470 | 0.0019 | 24.62 | 546,805 |
| BB7_240 | Khomani_San | Mbuti | 0.0443 | 0.0019 | 22.77 | 526,124 |

**Table S5.4 Shared genetic drift between *Bacho Kiro CC7-335* and present-day humans as measured by *f3(CC7-335, present-day humans; Mbuti)*.** Present-day modern human genomes from SGDP[10] were used. *f3* values reported were calculated using ADMIXTOOLS[3] as implemented in *admixr*[9] and were used for plotting Fig. S5.1C. Standard errors (SE) were computed using a Weighted Block Jackknife[3,12] across all autosomes using a block size of 5Mb. Number of SNPs overlapping among individuals reported in the column "nsnps".

| A | B | C | f3 | SE | Zscore | nsnps |
|---|---|---|---|---|---|---|
| CC7_335 | Lahu | Mbuti | 0.2250 | 0.0037 | 60.37 | 486,320 |
| CC7_335 | Oroqen | Mbuti | 0.2248 | 0.0038 | 59.38 | 487,060 |
| CC7_335 | Ulchi | Mbuti | 0.2242 | 0.0037 | 61.25 | 486,763 |
| CC7_335 | Han | Mbuti | 0.2241 | 0.0038 | 59.09 | 487,752 |
| CC7_335 | Daur | Mbuti | 0.2241 | 0.0039 | 57.08 | 466,483 |
| CC7_335 | Yi | Mbuti | 0.2241 | 0.0038 | 58.86 | 487,903 |
| CC7_335 | Eskimo_Naukan | Mbuti | 0.2239 | 0.0037 | 60.82 | 483,473 |
| CC7_335 | Japanese | Mbuti | 0.2239 | 0.0037 | 60.07 | 499,886 |
| CC7_335 | Itelman | Mbuti | 0.2239 | 0.0039 | 57.51 | 464,834 |
| CC7_335 | Surui | Mbuti | 0.2238 | 0.0038 | 58.80 | 474,876 |
| CC7_335 | Even | Mbuti | 0.2238 | 0.0038 | 59.09 | 501,167 |
| CC7_335 | Ami | Mbuti | 0.2237 | 0.0037 | 60.26 | 485,326 |
| CC7_335 | Dusun | Mbuti | 0.2234 | 0.0038 | 59.55 | 485,207 |
| CC7_335 | Khonda_Dora | Mbuti | 0.2234 | 0.0038 | 58.78 | 466,143 |
| CC7_335 | Eskimo_Chaplin | Mbuti | 0.2233 | 0.0038 | 58.02 | 464,589 |
| CC7_335 | Hezhen | Mbuti | 0.2233 | 0.0038 | 58.93 | 487,762 |
| CC7_335 | Dai | Mbuti | 0.2233 | 0.0037 | 60.28 | 499,888 |
| CC7_335 | Korean | Mbuti | 0.2232 | 0.0037 | 59.94 | 488,017 |
| CC7_335 | Burmese | Mbuti | 0.2232 | 0.0037 | 60.17 | 490,199 |
| CC7_335 | Karitiana | Mbuti | 0.2231 | 0.0039 | 57.47 | 474,521 |
| CC7_335 | Igorot | Mbuti | 0.2231 | 0.0038 | 58.39 | 483,610 |
| CC7_335 | Kinh | Mbuti | 0.2230 | 0.0038 | 59.38 | 488,238 |
| CC7_335 | Sherpa | Mbuti | 0.2229 | 0.0037 | 60.56 | 486,096 |
| CC7_335 | Cambodian | Mbuti | 0.2229 | 0.0037 | 59.48 | 488,908 |
| CC7_335 | Onge | Mbuti | 0.2229 | 0.0036 | 61.47 | 480,136 |
| CC7_335 | Chipewyan | Mbuti | 0.2228 | 0.0038 | 58.93 | 481,723 |
| CC7_335 | Naxi | Mbuti | 0.2228 | 0.0037 | 59.92 | 501,277 |
| CC7_335 | Thai | Mbuti | 0.2228 | 0.0037 | 60.36 | 489,285 |
| CC7_335 | Tujia | Mbuti | 0.2227 | 0.0038 | 59.31 | 487,384 |
| CC7_335 | Miao | Mbuti | 0.2226 | 0.0037 | 60.19 | 487,206 |
| CC7_335 | Yakut | Mbuti | 0.2225 | 0.0038 | 58.87 | 488,441 |
| CC7_335 | Tibetan | Mbuti | 0.2223 | 0.0037 | 60.17 | 488,222 |
| CC7_335 | Tu | Mbuti | 0.2221 | 0.0038 | 58.85 | 489,983 |
| CC7_335 | She | Mbuti | 0.2221 | 0.0037 | 59.30 | 487,033 |
| CC7_335 | Kusunda | Mbuti | 0.2220 | 0.0036 | 61.33 | 485,064 |
| CC7_335 | Botocudo | Mbuti | 0.2217 | 0.0039 | 57.47 | 440,853 |
| CC7_335 | Mayan | Mbuti | 0.2215 | 0.0037 | 59.90 | 483,026 |
| CC7_335 | Kurumba | Mbuti | 0.2214 | 0.0037 | 59.91 | 465,616 |
| CC7_335 | Atayal | Mbuti | 0.2213 | 0.0038 | 57.88 | 464,424 |
| CC7_335 | Eskimo_Sireniki | Mbuti | 0.2212 | 0.0036 | 61.99 | 485,676 |
| CC7_335 | Zapotec | Mbuti | 0.2210 | 0.0037 | 60.29 | 483,828 |
| CC7_335 | Xibo | Mbuti | 0.2210 | 0.0038 | 58.77 | 488,834 |
| CC7_335 | Chane | Mbuti | 0.2208 | 0.0037 | 60.19 | 464,254 |
| CC7_335 | Cree | Mbuti | 0.2207 | 0.0036 | 62.05 | 490,741 |
| CC7_335 | Kyrgyz | Mbuti | 0.2207 | 0.0037 | 59.69 | 493,250 |
| CC7_335 | Mixe | Mbuti | 0.2204 | 0.0038 | 58.49 | 480,981 |
| CC7_335 | Yadava | Mbuti | 0.2201 | 0.0036 | 60.73 | 491,886 |
| CC7_335 | Hawaiian | Mbuti | 0.2201 | 0.0040 | 54.83 | 465,927 |
| CC7_335 | Piapoco | Mbuti | 0.2198 | 0.0036 | 60.62 | 480,561 |
| CC7_335 | Quechua | Mbuti | 0.2195 | 0.0036 | 60.66 | 492,928 |
| CC7_335 | Kharia | Mbuti | 0.2194 | 0.0037 | 59.73 | 466,848 |
| CC7_335 | Madiga | Mbuti | 0.2194 | 0.0037 | 60.09 | 492,548 |
| CC7_335 | Aleut | Mbuti | 0.2193 | 0.0037 | 60.04 | 491,282 |
| CC7_335 | Pima | Mbuti | 0.2191 | 0.0038 | 58.23 | 480,344 |
| CC7_335 | Bougainville | Mbuti | 0.2191 | 0.0036 | 60.08 | 481,995 |
| CC7_335 | Nahua | Mbuti | 0.2187 | 0.0038 | 57.52 | 465,105 |
| CC7_335 | Chukchi | Mbuti | 0.2187 | 0.0036 | 60.12 | 470,182 |
| CC7_335 | Tubalar | Mbuti | 0.2180 | 0.0036 | 60.01 | 492,383 |
| CC7_335 | Irula | Mbuti | 0.2179 | 0.0035 | 61.81 | 491,403 |
| CC7_335 | Altaian | Mbuti | 0.2178 | 0.0038 | 57.02 | 467,186 |
| CC7_335 | Mala | Mbuti | 0.2177 | 0.0035 | 62.04 | 507,743 |
| CC7_335 | Papuan | Mbuti | 0.2176 | 0.0037 | 59.10 | 514,723 |
| CC7_335 | Hazara | Mbuti | 0.2171 | 0.0037 | 59.10 | 493,262 |
| CC7_335 | Punjabi | Mbuti | 0.2171 | 0.0035 | 61.22 | 519,396 |
| CC7_335 | Kapu | Mbuti | 0.2170 | 0.0036 | 60.06 | 493,356 |
| CC7_335 | Mansi | Mbuti | 0.2167 | 0.0035 | 61.38 | 492,953 |
| CC7_335 | Relli | Mbuti | 0.2165 | 0.0035 | 61.74 | 492,801 |
| CC7_335 | Mixtec | Mbuti | 0.2164 | 0.0037 | 58.81 | 487,871 |
| CC7_335 | Bengali | Mbuti | 0.2163 | 0.0035 | 61.12 | 493,518 |
| CC7_335 | Uygur | Mbuti | 0.2156 | 0.0035 | 61.47 | 495,209 |

| A | B | C | f3 | SE | Zscore | nsnps |
|---|---|---|---|---|---|---|
| CC7_335 | Tlingit | Mbuti | 0.2154 | 0.0036 | 59.30 | 493,493 |
| CC7_335 | Australian | Mbuti | 0.2152 | 0.0037 | 57.45 | 488,597 |
| CC7_335 | Sindhi | Mbuti | 0.2145 | 0.0036 | 59.95 | 493,942 |
| CC7_335 | Burusho | Mbuti | 0.2141 | 0.0036 | 60.27 | 494,720 |
| CC7_335 | Saami | Mbuti | 0.2139 | 0.0036 | 58.88 | 492,664 |
| CC7_335 | Brahmin | Mbuti | 0.2135 | 0.0035 | 60.66 | 494,553 |
| CC7_335 | Estonian | Mbuti | 0.2131 | 0.0036 | 59.32 | 494,602 |
| CC7_335 | Kashmiri_Pandit | Mbuti | 0.2129 | 0.0037 | 58.25 | 468,262 |
| CC7_335 | Russian | Mbuti | 0.2124 | 0.0036 | 58.26 | 495,113 |
| CC7_335 | Czech | Mbuti | 0.2124 | 0.0037 | 57.21 | 469,531 |
| CC7_335 | Pathan | Mbuti | 0.2124 | 0.0035 | 60.25 | 494,836 |
| CC7_335 | Finnish | Mbuti | 0.2123 | 0.0035 | 60.59 | 509,846 |
| CC7_335 | Icelandic | Mbuti | 0.2122 | 0.0035 | 59.80 | 494,918 |
| CC7_335 | Maori | Mbuti | 0.2117 | 0.0036 | 58.12 | 470,475 |
| CC7_335 | Polish | Mbuti | 0.2112 | 0.0037 | 56.84 | 469,527 |
| CC7_335 | Orcadian | Mbuti | 0.2110 | 0.0035 | 60.69 | 494,892 |
| CC7_335 | Tajik | Mbuti | 0.2109 | 0.0035 | 60.04 | 494,050 |
| CC7_335 | Basque | Mbuti | 0.2107 | 0.0035 | 59.80 | 493,880 |
| CC7_335 | Kalash | Mbuti | 0.2104 | 0.0035 | 60.01 | 490,352 |
| CC7_335 | French | Mbuti | 0.2103 | 0.0036 | 57.89 | 494,422 |
| CC7_335 | Albanian | Mbuti | 0.2102 | 0.0036 | 58.66 | 469,393 |
| CC7_335 | Balochi | Mbuti | 0.2102 | 0.0038 | 54.93 | 466,571 |
| CC7_335 | English | Mbuti | 0.2100 | 0.0035 | 60.15 | 494,908 |
| CC7_335 | Bulgarian | Mbuti | 0.2100 | 0.0036 | 59.15 | 495,201 |
| CC7_335 | Bergamo | Mbuti | 0.2099 | 0.0038 | 54.73 | 469,393 |
| CC7_335 | Hungarian | Mbuti | 0.2098 | 0.0037 | 57.31 | 495,273 |
| CC7_335 | Chechen | Mbuti | 0.2097 | 0.0037 | 57.25 | 469,420 |
| CC7_335 | Adygei | Mbuti | 0.2096 | 0.0035 | 59.34 | 494,760 |
| CC7_335 | Greek | Mbuti | 0.2094 | 0.0036 | 58.78 | 494,448 |
| CC7_335 | Armenian | Mbuti | 0.2094 | 0.0036 | 58.53 | 481,404 |
| CC7_335 | Turkish | Mbuti | 0.2094 | 0.0036 | 58.23 | 494,654 |
| CC7_335 | Sardinian | Mbuti | 0.2093 | 0.0035 | 60.16 | 494,116 |
| CC7_335 | North_Ossetian | Mbuti | 0.2091 | 0.0035 | 59.35 | 495,272 |
| CC7_335 | Abkhasian | Mbuti | 0.2091 | 0.0036 | 58.31 | 494,457 |
| CC7_335 | Tuscan | Mbuti | 0.2087 | 0.0035 | 58.91 | 495,405 |
| CC7_335 | Brahui | Mbuti | 0.2087 | 0.0035 | 59.17 | 494,007 |
| CC7_335 | Spanish | Mbuti | 0.2086 | 0.0035 | 59.09 | 494,911 |
| CC7_335 | Lezgin | Mbuti | 0.2082 | 0.0035 | 59.41 | 494,705 |
| CC7_335 | Iranian | Mbuti | 0.2077 | 0.0034 | 60.58 | 494,963 |
| CC7_335 | Georgian | Mbuti | 0.2071 | 0.0035 | 58.97 | 494,680 |
| CC7_335 | Crete | Mbuti | 0.2067 | 0.0037 | 55.52 | 469,483 |
| CC7_335 | Iraqi_Jew | Mbuti | 0.2057 | 0.0034 | 60.23 | 493,625 |
| CC7_335 | Makrani | Mbuti | 0.2054 | 0.0036 | 56.84 | 493,589 |
| CC7_335 | Samaritan | Mbuti | 0.2044 | 0.0036 | 57.12 | 466,236 |
| CC7_335 | Druze | Mbuti | 0.2031 | 0.0034 | 59.06 | 494,414 |
| CC7_335 | BedouinB | Mbuti | 0.1997 | 0.0034 | 58.65 | 491,331 |
| CC7_335 | Yemenite_Jew | Mbuti | 0.1994 | 0.0035 | 57.73 | 494,009 |
| CC7_335 | Jordanian | Mbuti | 0.1977 | 0.0034 | 57.95 | 510,776 |
| CC7_335 | Palestinian | Mbuti | 0.1970 | 0.0034 | 57.63 | 511,746 |
| CC7_335 | Saharawi | Mbuti | 0.1795 | 0.0032 | 56.60 | 497,555 |
| CC7_335 | Mozabite | Mbuti | 0.1772 | 0.0033 | 54.01 | 497,130 |
| CC7_335 | Somali | Mbuti | 0.1381 | 0.0032 | 42.53 | 465,274 |
| CC7_335 | Masai | Mbuti | 0.1139 | 0.0028 | 41.01 | 494,670 |
| CC7_335 | Dinka | Mbuti | 0.0920 | 0.0026 | 35.62 | 491,883 |
| CC7_335 | Gambian | Mbuti | 0.0880 | 0.0025 | 35.23 | 498,645 |
| CC7_335 | Mandenka | Mbuti | 0.0873 | 0.0025 | 34.68 | 499,105 |
| CC7_335 | Luo | Mbuti | 0.0865 | 0.0025 | 34.21 | 496,230 |
| CC7_335 | BantuKenya | Mbuti | 0.0861 | 0.0026 | 33.57 | 496,815 |
| CC7_335 | Luhya | Mbuti | 0.0859 | 0.0026 | 33.03 | 496,498 |
| CC7_335 | Esan | Mbuti | 0.0842 | 0.0025 | 33.07 | 499,118 |
| CC7_335 | Yoruba | Mbuti | 0.0836 | 0.0025 | 34.06 | 499,368 |
| CC7_335 | Mende | Mbuti | 0.0833 | 0.0025 | 33.98 | 498,637 |
| CC7_335 | Igbo | Mbuti | 0.0828 | 0.0025 | 33.31 | 498,676 |
| CC7_335 | Lemande | Mbuti | 0.0818 | 0.0025 | 33.17 | 497,854 |
| CC7_335 | BantuHerero | Mbuti | 0.0790 | 0.0025 | 32.15 | 496,294 |
| CC7_335 | Kongo | Mbuti | 0.0781 | 0.0026 | 29.96 | 466,479 |
| CC7_335 | BantuTswana | Mbuti | 0.0728 | 0.0024 | 30.98 | 496,443 |
| CC7_335 | Biaka | Mbuti | 0.0521 | 0.0021 | 24.88 | 487,451 |
| CC7_335 | Ju_hoan_North | Mbuti | 0.0478 | 0.0020 | 23.36 | 500,326 |
| CC7_335 | Khomani_San | Mbuti | 0.0455 | 0.0021 | 21.71 | 481,422 |

**Table S5.5 Shared genetic drift between *Bacho Kiro BK1653* and present-day humans as measured by *f3(BK1653, present-day humans; Mbuti)*.** Present-day modern human genomes from SGDP[10] were used. *f3* values reported were calculated using ADMIXTOOLS[3] as implemented in *admixr*[9] and were used for plotting Extended Data Fig. 3B. Standard errors (SE) were computed using a Weighted Block Jackknife[3,12] across all autosomes using a block size of 5Mb. Number of SNPs overlapping among individuals reported in the column "nsnps".

| A | B | C | f3 | stderr | Zscore | nsnps |
|---|---|---|---|---|---|---|
| BK1653 | Icelandic | Mbuti | 0.2412 | 0.0035 | 68.12 | 567,146 |
| BK1653 | Czech | Mbuti | 0.2403 | 0.0036 | 66.79 | 538,697 |
| BK1653 | Estonian | Mbuti | 0.2393 | 0.0036 | 65.99 | 566,739 |
| BK1653 | Finnish | Mbuti | 0.2389 | 0.0034 | 70.63 | 583,875 |
| BK1653 | English | Mbuti | 0.2386 | 0.0036 | 67.21 | 567,076 |
| BK1653 | Orcadian | Mbuti | 0.2381 | 0.0035 | 67.77 | 567,147 |
| BK1653 | French | Mbuti | 0.2381 | 0.0035 | 67.21 | 566,370 |
| BK1653 | Basque | Mbuti | 0.2378 | 0.0035 | 67.82 | 566,063 |
| BK1653 | Sardinian | Mbuti | 0.2378 | 0.0035 | 68.44 | 565,999 |
| BK1653 | Saami | Mbuti | 0.2375 | 0.0036 | 65.92 | 565,404 |
| BK1653 | Hungarian | Mbuti | 0.2373 | 0.0036 | 66.05 | 567,453 |
| BK1653 | Bergamo | Mbuti | 0.2372 | 0.0036 | 65.37 | 538,614 |
| BK1653 | Spanish | Mbuti | 0.2372 | 0.0036 | 66.37 | 567,155 |
| BK1653 | Russian | Mbuti | 0.2350 | 0.0035 | 68.10 | 567,223 |
| BK1653 | Bulgarian | Mbuti | 0.2349 | 0.0035 | 67.96 | 568,141 |
| BK1653 | Greek | Mbuti | 0.2349 | 0.0034 | 68.13 | 566,767 |
| BK1653 | Crete | Mbuti | 0.2345 | 0.0038 | 62.05 | 538,818 |
| BK1653 | Polish | Mbuti | 0.2344 | 0.0037 | 64.15 | 539,127 |
| BK1653 | Tuscan | Mbuti | 0.2332 | 0.0036 | 65.01 | 567,863 |
| BK1653 | Albanian | Mbuti | 0.2329 | 0.0036 | 64.67 | 538,698 |
| BK1653 | Adygei | Mbuti | 0.2322 | 0.0035 | 66.21 | 567,075 |
| BK1653 | Chukchi | Mbuti | 0.2319 | 0.0036 | 64.70 | 540,554 |
| BK1653 | Abkhasian | Mbuti | 0.2306 | 0.0035 | 65.28 | 567,007 |
| BK1653 | Georgian | Mbuti | 0.2303 | 0.0034 | 67.08 | 566,996 |
| BK1653 | North_Ossetian | Mbuti | 0.2301 | 0.0035 | 65.31 | 568,126 |
| BK1653 | Tajik | Mbuti | 0.2299 | 0.0035 | 65.45 | 566,732 |
| BK1653 | Mansi | Mbuti | 0.2299 | 0.0035 | 65.76 | 566,474 |
| BK1653 | Cree | Mbuti | 0.2297 | 0.0035 | 65.18 | 564,306 |
| BK1653 | Chechen | Mbuti | 0.2296 | 0.0036 | 63.23 | 539,041 |
| BK1653 | Lezgin | Mbuti | 0.2294 | 0.0035 | 66.37 | 567,006 |
| BK1653 | Tlingit | Mbuti | 0.2289 | 0.0035 | 65.97 | 566,773 |
| BK1653 | Pathan | Mbuti | 0.2281 | 0.0035 | 65.70 | 568,261 |
| BK1653 | Aleut | Mbuti | 0.2277 | 0.0035 | 65.13 | 564,802 |
| BK1653 | Turkish | Mbuti | 0.2276 | 0.0034 | 66.27 | 567,643 |
| BK1653 | Armenian | Mbuti | 0.2274 | 0.0037 | 61.95 | 552,929 |
| BK1653 | Chipewyan | Mbuti | 0.2271 | 0.0036 | 63.00 | 554,621 |
| BK1653 | Chane | Mbuti | 0.2261 | 0.0038 | 59.84 | 534,539 |
| BK1653 | Iranian | Mbuti | 0.2261 | 0.0035 | 64.82 | 567,592 |
| BK1653 | Druze | Mbuti | 0.2261 | 0.0033 | 67.56 | 567,073 |
| BK1653 | Mayan | Mbuti | 0.2261 | 0.0035 | 63.76 | 555,908 |
| BK1653 | Kashmiri_Pandit | Mbuti | 0.2261 | 0.0036 | 63.28 | 538,491 |
| BK1653 | Surui | Mbuti | 0.2260 | 0.0037 | 61.07 | 546,837 |
| BK1653 | Iraqi_Jew | Mbuti | 0.2259 | 0.0035 | 64.52 | 566,698 |
| BK1653 | Burusho | Mbuti | 0.2255 | 0.0035 | 64.29 | 568,480 |
| BK1653 | Tubalar | Mbuti | 0.2253 | 0.0035 | 64.61 | 566,190 |
| BK1653 | Quechua | Mbuti | 0.2252 | 0.0036 | 62.57 | 567,153 |
| BK1653 | Pima | Mbuti | 0.2250 | 0.0035 | 63.52 | 552,950 |
| BK1653 | Kalash | Mbuti | 0.2250 | 0.0035 | 65.17 | 563,325 |
| BK1653 | Piapoco | Mbuti | 0.2249 | 0.0037 | 61.35 | 553,043 |
| BK1653 | Zapotec | Mbuti | 0.2249 | 0.0036 | 63.05 | 557,066 |
| BK1653 | Nahua | Mbuti | 0.2247 | 0.0037 | 60.68 | 535,895 |
| BK1653 | Karitiana | Mbuti | 0.2247 | 0.0037 | 61.41 | 546,931 |
| BK1653 | Kyrgyz | Mbuti | 0.2246 | 0.0034 | 66.06 | 567,611 |
| BK1653 | Eskimo_Naukan | Mbuti | 0.2246 | 0.0035 | 64.56 | 556,829 |
| BK1653 | Samaritan | Mbuti | 0.2245 | 0.0038 | 59.79 | 535,337 |
| BK1653 | Maori | Mbuti | 0.2243 | 0.0037 | 60.96 | 541,257 |
| BK1653 | Eskimo_Chaplin | Mbuti | 0.2242 | 0.0038 | 59.11 | 535,586 |
| BK1653 | Hazara | Mbuti | 0.2242 | 0.0035 | 64.62 | 567,606 |
| BK1653 | Brahmin | Mbuti | 0.2241 | 0.0034 | 66.08 | 567,961 |
| BK1653 | Sindhi | Mbuti | 0.2240 | 0.0035 | 64.19 | 567,568 |
| BK1653 | Itelman | Mbuti | 0.2240 | 0.0036 | 62.20 | 535,848 |
| BK1653 | Mixtec | Mbuti | 0.2239 | 0.0035 | 63.89 | 561,248 |
| BK1653 | Altaian | Mbuti | 0.2237 | 0.0036 | 62.72 | 538,383 |
| BK1653 | Punjabi | Mbuti | 0.2237 | 0.0033 | 66.79 | 596,704 |
| BK1653 | Balochi | Mbuti | 0.2234 | 0.0037 | 60.66 | 536,702 |
| BK1653 | Mixe | Mbuti | 0.2234 | 0.0036 | 61.52 | 553,752 |
| BK1653 | Uygur | Mbuti | 0.2229 | 0.0035 | 64.40 | 569,437 |
| BK1653 | Brahui | Mbuti | 0.2229 | 0.0034 | 65.15 | 567,046 |
| BK1653 | Eskimo_Sireniki | Mbuti | 0.2227 | 0.0035 | 64.24 | 559,266 |
| BK1653 | Yadava | Mbuti | 0.2225 | 0.0034 | 65.31 | 565,596 |

| A | B | C | f3 | stderr | Zscore | nsnps |
|---|---|---|---|---|---|---|
| BK1653 | Yemenite_Jew | Mbuti | 0.2222 | 0.0035 | 63.75 | 566,408 |
| BK1653 | Kapu | Mbuti | 0.2218 | 0.0034 | 65.09 | 567,249 |
| BK1653 | BedouinB | Mbuti | 0.2216 | 0.0034 | 65.25 | 563,344 |
| BK1653 | Madiga | Mbuti | 0.2214 | 0.0034 | 64.24 | 566,852 |
| BK1653 | Bengali | Mbuti | 0.2212 | 0.0034 | 64.26 | 567,824 |
| BK1653 | Mala | Mbuti | 0.2212 | 0.0033 | 66.52 | 583,946 |
| BK1653 | Makrani | Mbuti | 0.2210 | 0.0035 | 63.56 | 566,729 |
| BK1653 | Yakut | Mbuti | 0.2205 | 0.0035 | 62.91 | 562,835 |
| BK1653 | Even | Mbuti | 0.2204 | 0.0035 | 63.00 | 577,274 |
| BK1653 | Relli | Mbuti | 0.2203 | 0.0034 | 65.03 | 567,044 |
| BK1653 | Kurumba | Mbuti | 0.2203 | 0.0036 | 61.59 | 537,067 |
| BK1653 | Palestinian | Mbuti | 0.2201 | 0.0034 | 64.00 | 586,450 |
| BK1653 | Khonda_Dora | Mbuti | 0.2193 | 0.0036 | 61.75 | 537,853 |
| BK1653 | She | Mbuti | 0.2191 | 0.0035 | 62.32 | 561,439 |
| BK1653 | Irula | Mbuti | 0.2191 | 0.0034 | 64.95 | 565,501 |
| BK1653 | Jordanian | Mbuti | 0.2190 | 0.0034 | 64.63 | 585,458 |
| BK1653 | Oroqen | Mbuti | 0.2190 | 0.0034 | 63.81 | 561,439 |
| BK1653 | Ulchi | Mbuti | 0.2187 | 0.0035 | 62.86 | 560,932 |
| BK1653 | Miao | Mbuti | 0.2186 | 0.0035 | 62.98 | 561,446 |
| BK1653 | Kinh | Mbuti | 0.2182 | 0.0036 | 61.20 | 562,644 |
| BK1653 | Kharia | Mbuti | 0.2180 | 0.0035 | 61.74 | 538,015 |
| BK1653 | Han | Mbuti | 0.2180 | 0.0035 | 63.00 | 562,450 |
| BK1653 | Tibetan | Mbuti | 0.2177 | 0.0035 | 62.20 | 563,061 |
| BK1653 | Dai | Mbuti | 0.2176 | 0.0035 | 62.46 | 575,587 |
| BK1653 | Burmese | Mbuti | 0.2175 | 0.0034 | 63.71 | 565,166 |
| BK1653 | Thai | Mbuti | 0.2175 | 0.0035 | 62.45 | 563,920 |
| BK1653 | Naxi | Mbuti | 0.2173 | 0.0035 | 62.25 | 577,546 |
| BK1653 | Ami | Mbuti | 0.2173 | 0.0036 | 60.05 | 559,436 |
| BK1653 | Korean | Mbuti | 0.2171 | 0.0035 | 62.51 | 562,443 |
| BK1653 | Onge | Mbuti | 0.2171 | 0.0036 | 61.10 | 553,779 |
| BK1653 | Hezhen | Mbuti | 0.2169 | 0.0034 | 63.15 | 561,938 |
| BK1653 | Daur | Mbuti | 0.2169 | 0.0036 | 59.64 | 538,188 |
| BK1653 | Tujia | Mbuti | 0.2168 | 0.0035 | 61.37 | 561,915 |
| BK1653 | Kusunda | Mbuti | 0.2167 | 0.0035 | 62.73 | 559,039 |
| BK1653 | Xibo | Mbuti | 0.2167 | 0.0035 | 61.54 | 563,581 |
| BK1653 | Yi | Mbuti | 0.2164 | 0.0035 | 62.54 | 563,195 |
| BK1653 | Lahu | Mbuti | 0.2164 | 0.0035 | 61.94 | 560,869 |
| BK1653 | Tu | Mbuti | 0.2162 | 0.0034 | 62.75 | 564,421 |
| BK1653 | Botocudo | Mbuti | 0.2162 | 0.0036 | 59.51 | 507,705 |
| BK1653 | Sherpa | Mbuti | 0.2159 | 0.0035 | 62.54 | 560,765 |
| BK1653 | Atayal | Mbuti | 0.2158 | 0.0037 | 58.47 | 535,879 |
| BK1653 | Australian | Mbuti | 0.2157 | 0.0036 | 59.81 | 563,162 |
| BK1653 | Igorot | Mbuti | 0.2156 | 0.0036 | 60.07 | 557,799 |
| BK1653 | Japanese | Mbuti | 0.2156 | 0.0034 | 62.73 | 576,154 |
| BK1653 | Cambodian | Mbuti | 0.2156 | 0.0035 | 61.50 | 564,057 |
| BK1653 | Dusun | Mbuti | 0.2155 | 0.0035 | 60.82 | 559,626 |
| BK1653 | Hawaiian | Mbuti | 0.2145 | 0.0037 | 58.54 | 537,517 |
| BK1653 | Bougainville | Mbuti | 0.2137 | 0.0035 | 60.42 | 555,929 |
| BK1653 | Papuan | Mbuti | 0.2129 | 0.0035 | 60.26 | 592,384 |
| BK1653 | Saharawi | Mbuti | 0.1985 | 0.0034 | 59.19 | 570,351 |
| BK1653 | Mozabite | Mbuti | 0.1963 | 0.0034 | 58.02 | 570,096 |
| BK1653 | Somali | Mbuti | 0.1474 | 0.0033 | 45.19 | 535,618 |
| BK1653 | Masai | Mbuti | 0.1192 | 0.0028 | 42.61 | 569,367 |
| BK1653 | Dinka | Mbuti | 0.0924 | 0.0026 | 35.02 | 566,732 |
| BK1653 | Mandenka | Mbuti | 0.0894 | 0.0026 | 34.93 | 574,270 |
| BK1653 | Gambian | Mbuti | 0.0879 | 0.0024 | 36.30 | 573,546 |
| BK1653 | Luo | Mbuti | 0.0864 | 0.0026 | 33.67 | 571,520 |
| BK1653 | Luhya | Mbuti | 0.0853 | 0.0024 | 34.86 | 571,591 |
| BK1653 | BantuKenya | Mbuti | 0.0852 | 0.0025 | 34.73 | 571,422 |
| BK1653 | Esan | Mbuti | 0.0831 | 0.0025 | 33.28 | 574,167 |
| BK1653 | Yoruba | Mbuti | 0.0830 | 0.0024 | 34.06 | 574,776 |
| BK1653 | Mende | Mbuti | 0.0827 | 0.0025 | 33.54 | 573,964 |
| BK1653 | Igbo | Mbuti | 0.0824 | 0.0024 | 33.88 | 574,063 |
| BK1653 | Lemande | Mbuti | 0.0815 | 0.0025 | 32.49 | 572,791 |
| BK1653 | Kongo | Mbuti | 0.0812 | 0.0026 | 31.51 | 537,560 |
| BK1653 | BantuHerero | Mbuti | 0.0794 | 0.0024 | 33.15 | 571,057 |
| BK1653 | BantuTswana | Mbuti | 0.0723 | 0.0023 | 31.26 | 571,965 |
| BK1653 | Biaka | Mbuti | 0.0520 | 0.0019 | 26.74 | 561,347 |
| BK1653 | Ju_hoan_North | Mbuti | 0.0465 | 0.0020 | 23.53 | 576,762 |
| BK1653 | Khomani_San | Mbuti | 0.0462 | 0.0021 | 22.27 | 554,629 |

**Table S5.6** ***D(IUP Bacho Kiro1, IUP Bacho Kiro2; IUP Bacho Kiro3, Mbuti)*****.** Derived allele sharing among IUP Bacho Kiro Cave specimens. These statistics were calculated using ADMIXTOOLS[3] as implemented in *admixr*[9]. Standard errors (SE) were computed using a Weighted Block Jackknife[3,12] across all autosomes of the "2200k" Panel and using a block size of 5Mb. Three Mbuti individuals from SGDP[10] were used as outgroup. Number of SNPs overlapping among individuals reported in the column "#SNPs".

| WW | X | Y | Z | D | SE | Z-score | ABBA | BABA | #SNPs |
|---|---|---|---|---|---|---|---|---|---|
| BachoKiro_F6_620 | BachoKiro_CC7_2289 | BachoKiro_BB7_240 | Mbuti | -0.0844 | 0.0429 | -1.966 | 196 | 232 | 4,280 |
| BachoKiro_F6_620 | BachoKiro_CC7_335 | BachoKiro_BB7_240 | Mbuti | -0.026 | 0.0103 | -2.523 | 17,437 | 18,370 | 383,549 |
| BachoKiro_CC7_2289 | BachoKiro_CC7_335 | BachoKiro_BB7_240 | Mbuti | 0.0949 | 0.0564 | 1.681 | 139 | 115 | 2,592 |
| BachoKiro_F6_620 | BachoKiro_BB7_240 | BachoKiro_CC7_335 | Mbuti | -0.0062 | 0.0107 | -0.578 | 18,144 | 18,370 | 383,549 |
| BachoKiro_F6_620 | BachoKiro_CC7_2289 | BachoKiro_CC7_335 | Mbuti | -0.0511 | 0.0454 | -1.126 | 173 | 191 | 3,824 |
| BachoKiro_BB7_240 | BachoKiro_CC7_2289 | BachoKiro_CC7_335 | Mbuti | -0.0278 | 0.0597 | -0.466 | 115 | 122 | 2,592 |
| BachoKiro_BB7_240 | BachoKiro_CC7_2289 | BachoKiro_F6_620 | Mbuti | -0.0227 | 0.0426 | -0.534 | 196 | 205 | 4,280 |
| BachoKiro_BB7_240 | BachoKiro_CC7_335 | BachoKiro_F6_620 | Mbuti | -0.0199 | 0.0102 | -1.953 | 17,437 | 18,144 | 383,549 |
| BachoKiro_CC7_2289 | BachoKiro_CC7_335 | BachoKiro_F6_620 | Mbuti | -0.0067 | 0.0480 | -0.139 | 170 | 173 | 3,824 |

**Table S5.7 *D(IUP Bacho Kiro$_1$, X; IUP Bacho Kiro CC7-2289, Mbuti)*.** Derived allele sharing between Bacho Kiro *CC7-2289* and the remaining three IUP Bacho Kiro Cave individuals. These statistics were calculated using ADMIXTOOLS[3] as implemented in *admixr*[9]. Standard errors (SE) were computed using a Weighted Block Jackknife[3,12] across all autosomes of the "2200k" Panel and using a block size of 5Mb. Number of SNPs overlapping among individuals reported in the column "#SNPs". Significant statistics (*Z-score*) or Z ≥ 3 highlighted in yellow.

| W | X | Y | Z | D | SE | Z-score | ABBA | BABA | #SNPs |
|---|---|---|---|---|---|---|---|---|---|
| BachoKiro_F6_620 | UstIshim | BachoKiro_CC7_2289 | Mbuti | 0.1209 | 0.030523 | 3.96 | 380 | 298 | 6,630 |
| BachoKiro_F6_620 | Tianyuan | BachoKiro_CC7_2289 | Mbuti | 0.1367 | 0.034261 | 3.99 | 366 | 278 | 6,440 |
| BachoKiro_F6_620 | Kostenki14 | BachoKiro_CC7_2289 | Mbuti | 0.156 | 0.035395 | 4.41 | 383 | 280 | 6,495 |
| BachoKiro_F6_620 | SunghirIII | BachoKiro_CC7_2289 | Mbuti | 0.1535 | 0.030878 | 4.97 | 396 | 291 | 6,629 |
| BachoKiro_F6_620 | GoyetQ116-1 | BachoKiro_CC7_2289 | Mbuti | 0.0711 | 0.039682 | 1.79 | 270 | 234 | 4,733 |
| BachoKiro_F6_620 | Vestonice16 | BachoKiro_CC7_2289 | Mbuti | 0.0747 | 0.038493 | 1.94 | 291 | 251 | 5,210 |
| BachoKiro_F6_620 | Yana_old | BachoKiro_CC7_2289 | Mbuti | 0.1185 | 0.030971 | 3.83 | 412 | 325 | 6,898 |
| BachoKiro_F6_620 | Malta1 | BachoKiro_CC7_2289 | Mbuti | 0.117 | 0.036479 | 3.21 | 292 | 231 | 4,993 |
| BachoKiro_F6_620 | ElMiron | BachoKiro_CC7_2289 | Mbuti | 0.0878 | 0.041912 | 2.10 | 259 | 217 | 4,723 |
| BachoKiro_F6_620 | Villabruna | BachoKiro_CC7_2289 | Mbuti | 0.1224 | 0.035597 | 3.44 | 357 | 279 | 6,015 |
| BachoKiro_F6_620 | Bichon | BachoKiro_CC7_2289 | Mbuti | 0.1322 | 0.031973 | 4.13 | 411 | 315 | 6,887 |
| BachoKiro_F6_620 | Satsurblia | BachoKiro_CC7_2289 | Mbuti | 0.1156 | 0.038892 | 2.97 | 279 | 221 | 4,643 |
| BachoKiro_F6_620 | Kotias | BachoKiro_CC7_2289 | Mbuti | 0.1575 | 0.031806 | 4.95 | 410 | 299 | 6,895 |
| BachoKiro_F6_620 | Karelia | BachoKiro_CC7_2289 | Mbuti | 0.128 | 0.033567 | 3.81 | 340 | 263 | 6,030 |
| BachoKiro_F6_620 | Loschbour | BachoKiro_CC7_2289 | Mbuti | 0.1465 | 0.029846 | 4.91 | 388 | 289 | 6,630 |
| BachoKiro_F6_620 | LaBrana1 | BachoKiro_CC7_2289 | Mbuti | 0.1142 | 0.033225 | 3.44 | 391 | 311 | 6,678 |
| BachoKiro_F6_620 | LBK | BachoKiro_CC7_2289 | Mbuti | 0.1174 | 0.030731 | 3.82 | 388 | 307 | 6,630 |
| BachoKiro_F6_620 | Saqqaq | BachoKiro_CC7_2289 | Mbuti | 0.136 | 0.033334 | 4.08 | 390 | 297 | 6,747 |
| BachoKiro_BB7_240 | UstIshim | BachoKiro_CC7_2289 | Mbuti | 0.1417 | 0.036482 | 3.88 | 247 | 186 | 4,150 |
| BachoKiro_BB7_240 | Tianyuan | BachoKiro_CC7_2289 | Mbuti | 0.1415 | 0.042673 | 3.32 | 239 | 180 | 4,150 |
| BachoKiro_BB7_240 | Kostenki14 | BachoKiro_CC7_2289 | Mbuti | 0.1882 | 0.040817 | 4.61 | 259 | 177 | 4,030 |
| BachoKiro_BB7_240 | SunghirIII | BachoKiro_CC7_2289 | Mbuti | 0.1947 | 0.037684 | 5.17 | 248 | 167 | 4,149 |
| BachoKiro_BB7_240 | GoyetQ116-1 | BachoKiro_CC7_2289 | Mbuti | 0.1395 | 0.045057 | 3.10 | 204 | 154 | 3,216 |
| BachoKiro_BB7_240 | Vestonice16 | BachoKiro_CC7_2289 | Mbuti | 0.1246 | 0.04492 | 2.77 | 202 | 158 | 3,525 |
| BachoKiro_BB7_240 | Yana_old | BachoKiro_CC7_2289 | Mbuti | 0.1551 | 0.040902 | 3.79 | 259 | 189 | 4,307 |
| BachoKiro_BB7_240 | Malta1 | BachoKiro_CC7_2289 | Mbuti | 0.1421 | 0.044923 | 3.16 | 185 | 139 | 3,131 |
| BachoKiro_BB7_240 | ElMiron | BachoKiro_CC7_2289 | Mbuti | 0.1232 | 0.046449 | 2.65 | 187 | 146 | 3,263 |
| BachoKiro_BB7_240 | Villabruna | BachoKiro_CC7_2289 | Mbuti | 0.1503 | 0.041844 | 3.59 | 246 | 182 | 3,986 |
| BachoKiro_BB7_240 | Bichon | BachoKiro_CC7_2289 | Mbuti | 0.1454 | 0.041171 | 3.53 | 255 | 190 | 4,301 |
| BachoKiro_BB7_240 | Satsurblia | BachoKiro_CC7_2289 | Mbuti | 0.1731 | 0.045897 | 3.77 | 185 | 130 | 2,919 |
| BachoKiro_BB7_240 | Kotias | BachoKiro_CC7_2289 | Mbuti | 0.1605 | 0.040556 | 3.96 | 266 | 192 | 4,306 |
| BachoKiro_BB7_240 | Karelia | BachoKiro_CC7_2289 | Mbuti | 0.1283 | 0.042825 | 3.00 | 236 | 182 | 3,808 |
| BachoKiro_BB7_240 | Loschbour | BachoKiro_CC7_2289 | Mbuti | 0.1829 | 0.037259 | 4.91 | 257 | 178 | 4,151 |
| BachoKiro_BB7_240 | LaBrana1 | BachoKiro_CC7_2289 | Mbuti | 0.1511 | 0.040459 | 3.74 | 257 | 190 | 4,194 |
| BachoKiro_BB7_240 | LBK | BachoKiro_CC7_2289 | Mbuti | 0.1455 | 0.036741 | 3.96 | 259 | 193 | 4,150 |
| BachoKiro_BB7_240 | Saqqaq | BachoKiro_CC7_2289 | Mbuti | 0.167 | 0.040003 | 4.17 | 252 | 180 | 4,246 |
| BachoKiro_CC7_335 | UstIshim | BachoKiro_CC7_2289 | Mbuti | 0.1158 | 0.040286 | 2.87 | 200 | 158 | 3,711 |
| BachoKiro_CC7_335 | Tianyuan | BachoKiro_CC7_2289 | Mbuti | 0.1594 | 0.044517 | 3.58 | 209 | 151 | 3,719 |
| BachoKiro_CC7_335 | Kostenki14 | BachoKiro_CC7_2289 | Mbuti | 0.1778 | 0.048147 | 3.69 | 207 | 145 | 3,616 |
| BachoKiro_CC7_335 | SunghirIII | BachoKiro_CC7_2289 | Mbuti | 0.1618 | 0.039198 | 4.13 | 215 | 155 | 3,710 |
| BachoKiro_CC7_335 | GoyetQ116-1 | BachoKiro_CC7_2289 | Mbuti | 0.108 | 0.048589 | 2.22 | 172 | 138 | 2,832 |
| BachoKiro_CC7_335 | Vestonice16 | BachoKiro_CC7_2289 | Mbuti | 0.0802 | 0.045406 | 1.77 | 186 | 158 | 3,135 |
| BachoKiro_CC7_335 | Yana_old | BachoKiro_CC7_2289 | Mbuti | 0.1377 | 0.042943 | 3.21 | 215 | 163 | 3,855 |
| BachoKiro_CC7_335 | Malta1 | BachoKiro_CC7_2289 | Mbuti | 0.1068 | 0.051949 | 2.06 | 145 | 117 | 2,805 |
| BachoKiro_CC7_335 | ElMiron | BachoKiro_CC7_2289 | Mbuti | 0.137 | 0.050974 | 2.69 | 178 | 135 | 2,902 |
| BachoKiro_CC7_335 | Villabruna | BachoKiro_CC7_2289 | Mbuti | 0.155 | 0.045352 | 3.42 | 211 | 154 | 3,527 |
| BachoKiro_CC7_335 | Bichon | BachoKiro_CC7_2289 | Mbuti | 0.1405 | 0.040644 | 3.46 | 220 | 166 | 3,852 |
| BachoKiro_CC7_335 | Kotias | BachoKiro_CC7_2289 | Mbuti | 0.1738 | 0.040859 | 4.25 | 223 | 157 | 3,851 |
| BachoKiro_CC7_335 | Karelia | BachoKiro_CC7_2289 | Mbuti | 0.1354 | 0.043918 | 3.08 | 186 | 142 | 3,372 |
| BachoKiro_CC7_335 | Loschbour | BachoKiro_CC7_2289 | Mbuti | 0.1399 | 0.040158 | 3.48 | 203 | 153 | 3,710 |
| BachoKiro_CC7_335 | LaBrana1 | BachoKiro_CC7_2289 | Mbuti | 0.1099 | 0.043837 | 2.51 | 206 | 165 | 3,760 |
| BachoKiro_CC7_335 | LBK | BachoKiro_CC7_2289 | Mbuti | 0.1086 | 0.04105 | 2.65 | 210 | 169 | 3,711 |
| BachoKiro_CC7_335 | Saqqaq | BachoKiro_CC7_2289 | Mbuti | 0.1384 | 0.041355 | 3.35 | 208 | 158 | 3,805 |

**Table S5.8 Z-scores of the statistics *D(Bacho Kiro F6-620, X; Y Mbuti)*.** Standard errors (SE) were computed using a Weighted Block Jackknife[3,12] across all autosomes of the “2200k” Panel and using a block size of 5Mb. Blue: Z-score ≤ -3; Yellow: Z-score ≥ 3.

| X/Y | BachoKiro_BB7_240 | BachoKiro_CC7_335 | UstIshim | Oase1 | Tianyuan | Kostenki14 | GoyetQ116-1 | BachoKiro_BK_1653 | SunghirIII | Yana_old | Vestonice16 | Malta1 | ElMiron | Villabruna | Bichon | Clovis | Satsurblia | Kolyma_River | Kotias | Karelia | Loschbour | LaBrana1 | Hungarian.KO1 | Motala12 | LBK | Saqqaq |
|---|---|---|---|---|---|---|---|---|---|---|---|---|---|---|---|---|---|---|---|---|---|---|---|---|---|---|
| BachoKiro_BB7_240 | NA | -0.58 | -0.73 | -0.84 | -1.66 | -1.99 | -2.67 | -1.85 | -2.04 | -0.21 | -2.11 | -0.40 | -2.16 | -2.57 | -2.89 | -1.25 | -1.53 | -0.46 | -1.69 | -0.72 | -2.17 | -1.91 | -1.79 | -2.14 | -0.76 | -0.41 |
| BachoKiro_CC7_335 | -2.52 | NA | -1.01 | -0.28 | -0.93 | -2.30 | -2.36 | 0.15 | -1.34 | -0.06 | -2.52 | -0.74 | -0.84 | -2.87 | -2.36 | -0.79 | -1.79 | -1.52 | -1.11 | -0.71 | -2.41 | -2.10 | -1.48 | -2.01 | -1.82 | -0.26 |
| UstIshim | 8.67 | 9.63 | NA | 5.94 | 0.63 | -4.02 | -3.14 | -1.23 | -3.10 | -1.10 | -3.74 | -2.36 | -2.41 | -3.21 | -3.91 | -0.95 | -3.37 | -1.72 | -2.00 | -0.80 | -3.84 | -3.34 | -1.48 | -3.61 | -2.17 | 0.20 |
| Oase1 | 4.64 | 6.66 | 2.73 | NA | 2.50 | 0.94 | 1.09 | 2.00 | 1.55 | 2.71 | 1.20 | 2.23 | 0.55 | 0.15 | 0.54 | 2.92 | 0.14 | 1.38 | 2.06 | 1.67 | 0.85 | 0.69 | 0.89 | 1.24 | 1.19 | 2.27 |
| Tianyuan | 6.79 | 8.30 | -1.59 | 3.47 | NA | -3.27 | -4.20 | -1.68 | -3.53 | -5.69 | -3.05 | -3.56 | -2.78 | -3.81 | -4.47 | -8.05 | -2.62 | -8.74 | -2.95 | -4.19 | -4.48 | -4.21 | -3.41 | -4.01 | -1.54 | -8.87 |
| Kostenki14 | 8.98 | 11.46 | -1.84 | 5.58 | 1.01 | NA | -11.57 | -12.26 | -17.98 | -10.17 | -15.93 | -9.26 | -12.87 | -14.31 | -14.56 | -2.94 | -9.35 | -2.52 | -9.13 | -10.37 | -15.42 | -14.95 | -12.56 | -13.88 | -10.10 | -0.83 |
| GoyetQ116-1_old | 5.77 | 7.66 | -3.04 | 3.33 | -2.20 | -14.94 | NA | -15.82 | -13.80 | -10.15 | -15.46 | -10.66 | -22.26 | -15.01 | -16.22 | -4.26 | -8.41 | -4.66 | -9.17 | -11.33 | -18.84 | -20.03 | -14.21 | -15.43 | -12.57 | -2.89 |
| BachoKiro_BK_1653 | 8.50 | 11.46 | -0.18 | 5.52 | 0.98 | -13.16 | -13.34 | NA | -12.38 | -7.61 | -17.39 | -7.00 | -14.48 | -15.82 | -15.72 | -1.84 | -7.28 | -2.06 | -7.82 | -10.02 | -15.60 | -16.72 | -13.01 | -13.83 | -12.34 | -0.55 |
| SunghirIII | 9.14 | 11.47 | -1.80 | 5.71 | -0.29 | -19.14 | -11.76 | -12.37 | NA | -7.59 | -21.64 | -9.47 | -13.79 | -15.43 | -16.54 | -2.59 | -9.05 | -2.72 | -9.74 | -11.55 | -16.70 | -16.67 | -14.72 | -14.17 | -11.44 | -1.60 |
| Yana_old | 9.48 | 11.55 | -1.49 | 5.66 | -3.74 | -12.54 | -9.67 | -9.21 | -9.22 | NA | -11.94 | -11.18 | -9.53 | -10.52 | -12.06 | -7.10 | -7.86 | -6.04 | -8.24 | -11.14 | -12.16 | -11.87 | -11.06 | -10.41 | -9.18 | -5.66 |
| Vestonice16 | 7.73 | 9.56 | -2.08 | 4.64 | 0.33 | -17.21 | -12.22 | -17.39 | -21.84 | -10.69 | NA | -11.04 | -16.90 | -19.03 | -18.32 | -3.94 | -9.09 | -3.21 | -9.08 | -12.44 | -20.30 | -18.25 | -18.60 | -16.40 | -12.94 | -1.78 |
| Malta1 | 9.25 | 10.19 | -1.91 | 4.77 | -1.51 | -11.61 | -9.59 | -8.32 | -10.18 | -10.34 | -11.68 | NA | -9.98 | -12.10 | -14.21 | -13.35 | -11.45 | -11.56 | -11.26 | -21.53 | -14.56 | -13.11 | -13.88 | -19.81 | -9.84 | -8.75 |
| ElMiron | 7.80 | 9.32 | -1.64 | 3.51 | -0.07 | -15.49 | -19.76 | -14.87 | -13.87 | -8.63 | -17.41 | -9.16 | NA | -26.67 | -28.54 | -5.34 | -8.98 | -3.94 | -11.08 | -14.38 | -30.94 | -34.40 | -23.10 | -22.09 | -14.99 | -2.40 |
| Villabruna | 8.39 | 10.27 | -1.48 | 4.15 | -0.12 | -14.52 | -12.24 | -15.03 | -14.62 | -8.34 | -18.22 | -10.37 | -24.63 | NA | -41.40 | -4.54 | -12.63 | -4.39 | -14.00 | -19.97 | -44.93 | -37.85 | -38.04 | -32.43 | -19.41 | -2.73 |
| Bichon | 8.66 | 11.09 | -1.66 | 5.85 | -0.05 | -14.56 | -12.80 | -13.67 | -14.83 | -8.72 | -16.98 | -12.02 | -26.40 | -39.83 | NA | -4.79 | -13.44 | -5.29 | -13.51 | -20.57 | -45.57 | -38.74 | -36.29 | -32.40 | -18.59 | -4.06 |
| Clovis | 8.41 | 10.38 | -1.83 | 5.17 | -6.15 | -6.03 | -4.20 | -3.68 | -4.80 | -7.42 | -5.82 | -13.73 | -6.77 | -7.01 | -8.41 | NA | -8.67 | -22.65 | -7.58 | -15.26 | -10.01 | -9.52 | -8.89 | -12.10 | -6.57 | -21.38 |
| Satsurblia | 12.59 | 12.99 | 1.51 | 7.82 | 4.27 | -5.96 | -2.54 | -3.16 | -4.96 | -1.98 | -4.75 | -6.91 | -4.90 | -9.01 | -11.55 | -2.20 | NA | -2.12 | -27.74 | -10.59 | -11.13 | -9.08 | -10.59 | -11.71 | -15.05 | -0.09 |
| Kolyma_River | 8.91 | 9.96 | -2.61 | 4.19 | -7.13 | -5.46 | -4.96 | -3.99 | -5.00 | -6.84 | -5.23 | -12.44 | -5.48 | -7.35 | -9.04 | -23.97 | -8.23 | NA | -7.67 | -14.57 | -8.52 | -7.96 | -8.82 | -11.27 | -6.45 | -25.92 |
| Kotias | 11.61 | 14.34 | 1.59 | 8.24 | 3.25 | -7.06 | -3.93 | -4.66 | -6.41 | -3.38 | -6.00 | -7.20 | -7.19 | -10.66 | -12.07 | -2.38 | -28.28 | -2.57 | NA | -11.96 | -12.56 | -11.23 | -11.48 | -13.63 | -15.85 | -2.05 |
| Karelia | 9.56 | 11.64 | -0.06 | 5.67 | -0.96 | -12.29 | -9.50 | -10.18 | -11.74 | -9.43 | -12.72 | -20.23 | -14.47 | -21.12 | -23.90 | -13.93 | -15.77 | -13.04 | -15.25 | NA | -24.22 | -22.41 | -25.03 | -29.50 | -16.22 | -9.70 |
| Loschbour | 9.40 | 11.06 | -1.50 | 6.23 | -0.06 | -14.38 | -14.74 | -14.08 | -14.81 | -8.91 | -17.50 | -11.81 | -27.59 | -41.83 | -44.77 | -6.03 | -13.24 | -4.81 | -13.72 | -21.12 | NA | -39.30 | -37.19 | -33.99 | -19.70 | -3.90 |
| LaBrana1 | 9.65 | 11.46 | -1.17 | 5.92 | 0.22 | -13.79 | -16.06 | -14.75 | -14.65 | -8.77 | -15.92 | -10.76 | -30.41 | -35.90 | -39.47 | -5.90 | -11.58 | -4.60 | -13.38 | -19.28 | -41.59 | NA | -32.81 | -29.36 | -17.90 | -4.19 |
| Hungarian.KO1 | 9.54 | 11.18 | 0.01 | 5.02 | 0.88 | -13.19 | -11.35 | -12.29 | -13.81 | -8.62 | -16.21 | -12.37 | -20.83 | -37.69 | -38.10 | -6.02 | -13.88 | -5.83 | -14.46 | -22.87 | -39.56 | -36.23 | NA | -32.48 | -21.44 | -4.69 |
| Motala12 | 10.64 | 12.21 | -1.10 | 6.01 | 0.54 | -12.52 | -11.80 | -12.05 | -11.88 | -7.30 | -14.55 | -16.84 | -19.15 | -31.00 | -32.02 | -8.54 | -14.52 | -7.32 | -16.18 | -27.37 | -34.22 | -29.47 | -30.83 | NA | -19.78 | -5.13 |
| LBK | 12.58 | 13.58 | 1.32 | 8.16 | 4.40 | -8.05 | -6.49 | -8.89 | -8.34 | -3.97 | -9.42 | -5.88 | -10.58 | -15.81 | -16.79 | -1.23 | -15.68 | -1.14 | -16.46 | -12.06 | -17.91 | -15.58 | -17.59 | -17.53 | NA | -1.04 |
| Saqqaq | 8.26 | 10.27 | -1.55 | 4.56 | -7.68 | -4.62 | -3.84 | -3.37 | -4.52 | -7.08 | -4.49 | -10.84 | -4.67 | -6.16 | -8.18 | -23.90 | -7.22 | -27.06 | -8.12 | -12.61 | -8.44 | -8.50 | -8.51 | -9.44 | -7.28 | NA |

**Table S5.9 Z-scores of the statistics *D(Bacho Kiro BB7-240, X; Y Mbuti)***. Standard errors (SE) were computed using a Weighted Block Jackknife[3,12] across all autosomes of the "2200k" Panel and using a block size of 5Mb. Blue: Z-score ≤ -3; Yellow: Z-score ≥ 3.

| X/Y | BachoKiro_F6_620 | BachoKiro_CC7_335 | UstIshim | Oase1 | Tianyuan | Kostenki14 | GoyetQ116-1 | BachoKiro_BK1653 | SunghirIII | Yana_old | Vestonice16 | Malta1 | ElMiron | Villabruna | Bichon | Clovis | Satsurblia | Kolyma_River | Kotias | Karelia | Loschbour | LaBrana1 | Hungarian.KO1 | Motala12 | LBK | Saqqaq |
|---|---|---|---|---|---|---|---|---|---|---|---|---|---|---|---|---|---|---|---|---|---|---|---|---|---|---|
| BachoKiro_F6_620 | NA | 0.58 | 0.73 | 0.84 | 1.66 | 1.99 | 2.67 | 1.85 | 2.04 | 0.21 | 2.11 | 0.40 | 2.16 | 2.57 | 2.89 | 1.25 | 1.53 | 0.46 | 1.69 | 0.72 | 2.17 | 1.91 | 1.79 | 2.14 | 0.76 | 0.41 |
| BachoKiro_CC7_335 | -1.95 | NA | 0.09 | 0.97 | 0.73 | -0.01 | 0.94 | 2.01 | 0.91 | 0.53 | 0.09 | -0.15 | 0.07 | -0.53 | 0.45 | 0.49 | -0.24 | -0.40 | 0.72 | 0.59 | 0.68 | -0.26 | 0.52 | 0.40 | -0.33 | 0.94 |
| UstIshim | 9.42 | 10.87 | NA | 6.62 | 1.23 | -1.86 | 0.22 | 0.72 | -1.41 | -1.07 | -1.57 | -1.59 | -0.42 | -0.66 | -1.07 | -0.33 | -2.00 | -1.87 | -0.55 | -0.11 | -1.82 | -1.47 | -0.20 | -1.66 | -1.82 | 0.06 |
| Oase1 | 5.28 | 6.62 | 2.70 | NA | 2.53 | 1.86 | 3.25 | 1.86 | 2.23 | 2.95 | 3.36 | 2.12 | 2.63 | 1.34 | 2.41 | 2.77 | 1.87 | 1.13 | 2.38 | 1.16 | 2.90 | 2.31 | 1.64 | 1.50 | 1.57 | 2.20 |
| Tianyuan | 8.56 | 9.95 | -1.23 | 3.88 | NA | -1.04 | -0.50 | 0.97 | -1.43 | -4.86 | -0.08 | -2.88 | -0.59 | -1.02 | -1.26 | -6.10 | -0.55 | -7.98 | -0.84 | -2.89 | -2.11 | -1.39 | -1.39 | -1.60 | -0.86 | -7.70 |
| Kostenki14 | 11.45 | 11.97 | -1.29 | 6.15 | 2.28 | NA | -8.32 | -9.78 | -15.47 | -9.24 | -12.17 | -8.36 | -9.82 | -10.67 | -11.03 | -2.12 | -6.93 | -2.74 | -7.83 | -9.79 | -12.45 | -11.55 | -11.25 | -10.75 | -9.49 | -0.67 |
| GoyetQ116-1_old | 9.13 | 9.00 | -2.66 | 4.07 | -1.17 | -12.41 | NA | -13.19 | -11.68 | -9.02 | -11.08 | -9.15 | -19.80 | -11.83 | -13.01 | -2.88 | -6.06 | -3.90 | -7.04 | -9.72 | -16.28 | -16.39 | -11.60 | -12.65 | -10.94 | -2.46 |
| BachoKiro_BK1653 | 10.69 | 12.45 | 0.60 | 6.31 | 3.07 | -11.26 | -9.89 | NA | -11.36 | -7.10 | -13.61 | -6.45 | -11.93 | -12.41 | -11.58 | -0.61 | -5.04 | -1.09 | -6.56 | -9.19 | -13.26 | -12.79 | -10.12 | -10.88 | -10.93 | 0.34 |
| SunghirIII | 11.59 | 13.38 | -1.44 | 6.22 | 1.26 | -16.11 | -8.15 | -10.04 | NA | -7.76 | -17.90 | -8.63 | -11.85 | -12.15 | -13.94 | -2.07 | -7.68 | -3.17 | -9.19 | -10.72 | -15.05 | -13.99 | -13.34 | -13.02 | -11.91 | -1.47 |
| Yana_old | 10.23 | 12.44 | -0.73 | 6.91 | -2.12 | -9.54 | -5.96 | -6.63 | -7.42 | NA | -8.27 | -10.75 | -6.69 | -7.00 | -8.11 | -5.45 | -5.92 | -5.98 | -6.78 | -9.87 | -10.00 | -9.56 | -9.15 | -8.56 | -8.64 | -5.05 |
| Vestonice16 | 10.79 | 10.70 | -2.08 | 6.16 | 1.64 | -13.75 | -9.04 | -14.52 | -18.20 | -9.81 | NA | -8.99 | -14.51 | -15.38 | -14.99 | -2.42 | -7.16 | -2.99 | -7.72 | -12.17 | -17.54 | -14.67 | -15.03 | -14.16 | -11.72 | -1.56 |
| Malta1 | 10.01 | 10.92 | -1.65 | 5.35 | -0.71 | -9.29 | -6.37 | -6.40 | -8.72 | -10.46 | -8.59 | NA | -7.44 | -9.19 | -11.20 | -11.87 | -10.17 | -10.33 | -10.35 | -20.15 | -12.46 | -11.52 | -12.16 | -16.55 | -9.80 | -8.71 |
| ElMiron | 10.16 | 10.28 | -1.30 | 4.91 | 1.00 | -11.75 | -16.22 | -12.32 | -12.59 | -8.26 | -13.39 | -7.51 | NA | -22.51 | -24.94 | -4.00 | -6.59 | -3.40 | -8.69 | -12.95 | -29.63 | -29.23 | -20.19 | -20.08 | -13.62 | -1.98 |
| Villabruna | 11.14 | 11.70 | -0.93 | 4.77 | 1.10 | -11.65 | -8.59 | -12.20 | -12.78 | -7.62 | -14.14 | -9.37 | -21.80 | NA | -35.78 | -3.37 | -10.80 | -3.99 | -12.27 | -18.47 | -42.27 | -32.07 | -33.40 | -29.12 | -18.12 | -2.27 |
| Bichon | 12.24 | 12.18 | -1.38 | 6.51 | 0.86 | -11.36 | -9.73 | -11.19 | -13.62 | -8.72 | -13.61 | -11.80 | -23.91 | -36.56 | NA | -4.39 | -11.99 | -5.64 | -12.54 | -19.34 | -41.86 | -35.65 | -32.01 | -30.86 | -18.26 | -3.82 |
| Clovis | 9.51 | 11.50 | -1.21 | 5.76 | -3.80 | -3.67 | -0.49 | -1.04 | -2.94 | -6.71 | -2.78 | -12.69 | -4.11 | -3.95 | -4.87 | NA | -6.63 | -21.88 | -5.82 | -14.33 | -7.44 | -6.29 | -6.71 | -9.88 | -5.84 | -20.01 |
| Satsurblia | 13.75 | 13.66 | 1.52 | 7.93 | 5.31 | -3.62 | 0.76 | -1.21 | -3.23 | -2.23 | -2.25 | -5.74 | -2.39 | -5.89 | -7.55 | -1.32 | NA | -2.02 | -24.16 | -8.84 | -8.20 | -6.88 | -8.26 | -8.75 | -13.33 | 0.68 |
| Kolyma_River | 9.70 | 11.18 | -1.97 | 4.98 | -5.34 | -3.48 | -1.34 | -1.51 | -3.34 | -6.29 | -2.65 | -10.93 | -2.96 | -4.49 | -5.59 | -21.98 | -6.63 | NA | -6.52 | -13.13 | -6.27 | -5.55 | -6.99 | -8.73 | -6.02 | -24.59 |
| Kotias | 13.18 | 15.11 | 2.28 | 8.10 | 4.60 | -5.15 | -0.89 | -3.00 | -5.02 | -3.45 | -3.42 | -6.86 | -4.96 | -7.85 | -8.74 | -1.53 | -25.62 | -2.94 | NA | -10.59 | -10.26 | -9.12 | -10.17 | -11.93 | -14.69 | -1.75 |
| Karelia | 10.24 | 11.86 | 0.36 | 6.06 | 0.37 | -9.24 | -5.94 | -7.84 | -9.88 | -8.78 | -9.78 | -17.91 | -11.59 | -16.80 | -18.26 | -12.39 | -13.54 | -11.97 | -13.69 | NA | -21.59 | -18.62 | -21.29 | -26.48 | -14.95 | -9.31 |
| Loschbour | 11.56 | 12.82 | -1.15 | 7.15 | 1.28 | -11.34 | -11.36 | -11.42 | -13.61 | -8.69 | -13.97 | -10.92 | -25.18 | -38.85 | -39.63 | -5.41 | -11.53 | -5.03 | -12.81 | -20.43 | NA | -36.56 | -34.40 | -33.30 | -19.25 | -3.81 |
| LaBrana1 | 11.96 | 12.54 | -0.69 | 6.61 | 1.86 | -10.48 | -11.77 | -11.08 | -13.08 | -8.50 | -12.06 | -9.90 | -25.83 | -31.08 | -34.94 | -4.49 | -10.20 | -4.41 | -12.08 | -17.63 | -38.33 | NA | -29.65 | -26.68 | -17.19 | -3.43 |
| Hungarian.KO1 | 11.52 | 11.86 | 0.34 | 5.47 | 1.63 | -11.06 | -7.56 | -9.41 | -11.83 | -8.30 | -12.34 | -11.02 | -18.44 | -32.09 | -32.04 | -5.21 | -11.91 | -5.79 | -13.77 | -21.21 | -36.71 | -30.64 | NA | -30.18 | -20.75 | -4.28 |
| Motala12 | 12.43 | 13.29 | -0.18 | 6.35 | 2.15 | -9.17 | -7.72 | -8.87 | -10.02 | -7.08 | -11.11 | -14.40 | -16.80 | -27.45 | -26.44 | -6.83 | -12.25 | -6.58 | -14.03 | -24.65 | -32.07 | -25.08 | -28.45 | NA | -18.09 | -4.40 |
| LBK | 13.14 | 14.70 | 1.68 | 8.49 | 5.33 | -5.56 | -3.00 | -6.35 | -6.82 | -4.19 | -6.31 | -5.13 | -8.06 | -12.51 | -12.65 | -0.68 | -13.57 | -1.54 | -14.39 | -10.59 | -15.19 | -13.54 | -15.66 | -15.34 | NA | -1.27 |
| Saqqaq | 8.85 | 11.16 | -1.19 | 4.98 | -5.71 | -2.49 | -0.44 | -1.13 | -2.77 | -6.44 | -1.78 | -8.81 | -2.59 | -3.12 | -4.73 | -20.03 | -5.12 | -24.82 | -6.50 | -11.49 | -5.69 | -5.34 | -5.94 | -7.40 | -7.08 | NA |

**Table S5.10 Z-scores of the statistics *D(Bacho Kiro CC7-335, X; Y Mbuti)*.** Standard errors (SE) were computed using a Weighted Block Jackknife[3,12] across all autosomes of the "2200k" Panel and using a block size of 5Mb. Blue: Z-score ≤ -3; Yellow: Z-score ≥ 3.

| X/Y | BachoKiro_F6_620 | BachoKiro_BB7_240 | UstIshim | Oase1 | Tianyuan | Kostenki14 | GoyetQ116-1 | BachoKiro_BK1653 | SunghirIII | Yana_old2 | Vestonice16 | Malta1 | ElMiron | Villabruna | Bichon | Clovis | Satsurblia | Kolyma_River | Kotias | Karelia | Loschbour | LaBrana1 | Hungarian.KO1 | Motala12 | LBK | Saqqaq |
|---|---|---|---|---|---|---|---|---|---|---|---|---|---|---|---|---|---|---|---|---|---|---|---|---|---|---|
| BachoKiro_F6_620 | NA | 2.52 | 1.01 | 0.28 | 0.93 | 2.30 | 2.36 | 0.15 | 1.34 | 0.06 | 2.52 | 0.74 | 0.84 | 2.87 | 2.36 | 0.79 | 1.79 | 1.52 | 1.11 | 0.71 | 2.41 | 2.10 | 1.48 | 2.01 | 1.82 | 0.26 |
| BachoKiro_BB7_240 | 1.95 | NA | -0.09 | -0.97 | -0.73 | 0.01 | -0.94 | -2.01 | -0.91 | -0.53 | -0.09 | 0.15 | -0.07 | 0.53 | -0.45 | -0.49 | 0.24 | 0.40 | -0.72 | -0.59 | -0.68 | 0.26 | -0.52 | -0.40 | 0.33 | -0.94 |
| UstIshim | 11.04 | 11.00 | NA | 5.93 | 1.35 | -2.20 | -0.70 | -1.60 | -2.34 | -1.10 | -1.52 | -0.67 | -1.26 | -0.40 | -2.13 | -0.69 | -1.25 | -0.87 | -0.89 | -0.46 | -2.06 | -1.25 | -0.78 | -1.91 | -0.89 | 0.12 |
| Oase1 | 6.88 | 5.55 | 3.34 | NA | 2.43 | 1.38 | 2.06 | 1.00 | 1.74 | 2.16 | 1.71 | 2.02 | 1.37 | 1.53 | 1.49 | 2.78 | 1.76 | 2.08 | 2.44 | 0.83 | 2.54 | 3.11 | 1.27 | 1.77 | 2.14 | 2.33 |
| Tianyuan | 9.32 | 9.40 | -0.50 | 3.52 | NA | -1.49 | -1.83 | -1.30 | -2.64 | -5.44 | -0.65 | -1.78 | -0.98 | -0.53 | -1.94 | -7.18 | -0.59 | -7.52 | -1.41 | -3.23 | -2.10 | -1.82 | -2.24 | -2.05 | -0.14 | -8.08 |
| Kostenki14 | 13.68 | 12.01 | -1.00 | 4.86 | 1.79 | NA | -8.97 | -11.46 | -16.30 | -9.84 | -12.43 | -7.68 | -9.96 | -10.85 | -11.95 | -1.98 | -6.39 | -1.52 | -7.41 | -9.96 | -12.05 | -11.24 | -11.18 | -11.00 | -8.60 | -1.18 |
| GoyetQ116-1 | 10.51 | 7.96 | -2.59 | 2.43 | -2.07 | -12.24 | NA | -15.02 | -12.29 | -10.02 | -12.29 | -8.70 | -20.44 | -12.01 | -14.15 | -3.38 | -6.33 | -4.07 | -8.63 | -11.30 | -17.05 | -16.81 | -12.49 | -13.57 | -11.38 | -2.75 |
| BachoKiro_BK1653 | 11.31 | 10.34 | 0.74 | 5.57 | 2.65 | -10.45 | -10.12 | NA | -10.94 | -7.30 | -13.76 | -5.34 | -11.43 | -11.98 | -11.70 | -0.83 | -4.14 | -0.36 | -6.24 | -8.73 | -12.38 | -12.21 | -10.47 | -10.68 | -9.74 | -0.45 |
| SunghirIII | 13.39 | 12.46 | -0.82 | 5.07 | 0.61 | -15.78 | -8.56 | -12.18 | NA | -7.43 | -18.09 | -7.48 | -11.00 | -11.84 | -13.99 | -1.79 | -6.18 | -1.97 | -7.95 | -10.54 | -13.46 | -13.09 | -12.54 | -11.28 | -9.89 | -1.28 |
| Yana_old2 | 11.81 | 11.58 | -0.20 | 5.19 | -2.40 | -10.07 | -6.31 | -8.30 | -8.00 | NA | -8.79 | -9.65 | -7.27 | -6.64 | -8.77 | -6.23 | -4.98 | -5.21 | -6.41 | -9.76 | -9.25 | -8.45 | -9.00 | -8.53 | -7.10 | -5.75 |
| Vestonice16 | 12.42 | 10.46 | -1.80 | 4.01 | 0.97 | -14.48 | -9.55 | -16.46 | -20.06 | -10.74 | NA | -9.22 | -14.67 | -16.24 | -15.37 | -2.80 | -6.51 | -2.40 | -8.65 | -11.91 | -17.12 | -14.28 | -15.15 | -15.28 | -11.36 | -2.14 |
| Malta1 | 11.46 | 10.86 | -0.97 | 4.21 | -0.48 | -9.30 | -6.84 | -8.13 | -9.46 | -10.72 | -9.23 | NA | -7.62 | -8.89 | -11.10 | -12.67 | -9.10 | -10.41 | -10.72 | -20.10 | -11.78 | -10.32 | -11.76 | -16.01 | -8.78 | -9.09 |
| ElMiron | 10.60 | 10.47 | -1.56 | 3.16 | 0.32 | -12.58 | -17.40 | -14.52 | -13.38 | -9.30 | -15.13 | -7.99 | NA | -22.49 | -26.66 | -5.12 | -6.73 | -4.04 | -10.33 | -14.14 | -27.51 | -29.21 | -19.58 | -20.91 | -13.32 | -2.95 |
| Villabruna | 13.20 | 11.92 | -0.29 | 4.20 | 1.50 | -12.14 | -9.03 | -13.65 | -13.26 | -7.98 | -15.08 | -8.62 | -20.36 | NA | -37.03 | -3.32 | -9.68 | -3.59 | -11.78 | -19.07 | -39.86 | -32.15 | -34.90 | -28.54 | -17.54 | -2.12 |
| Bichon | 13.76 | 11.74 | -0.89 | 5.12 | 0.79 | -12.74 | -10.40 | -13.48 | -14.45 | -9.03 | -14.06 | -10.67 | -23.35 | -36.62 | NA | -4.54 | -10.88 | -4.60 | -12.40 | -19.20 | -40.98 | -35.69 | -32.07 | -28.77 | -17.05 | -4.21 |
| Clovis | 11.54 | 10.63 | -0.60 | 5.26 | -4.59 | -3.52 | -1.38 | -3.19 | -3.37 | -7.09 | -2.85 | -11.92 | -4.48 | -3.86 | -5.34 | NA | -6.19 | -21.79 | -6.14 | -15.12 | -6.68 | -6.42 | -7.05 | -10.31 | -4.95 | -20.41 |
| Satsurblia | 15.43 | 14.99 | 2.45 | 6.85 | 4.19 | -4.04 | -0.64 | -3.01 | -4.10 | -1.98 | -2.38 | -4.90 | -2.69 | -6.26 | -8.25 | -2.06 | NA | -1.47 | -24.76 | -9.39 | -8.66 | -7.47 | -9.00 | -8.89 | -12.81 | -0.37 |
| Kolyma_River | 12.09 | 11.65 | -1.33 | 4.13 | -6.06 | -3.36 | -2.32 | -3.86 | -4.05 | -6.81 | -2.65 | -10.90 | -3.93 | -4.27 | -6.11 | -23.51 | -6.36 | NA | -6.87 | -13.74 | -5.94 | -5.30 | -7.27 | -8.48 | -4.78 | -24.48 |
| Kotias | 15.03 | 14.67 | 3.19 | 7.91 | 4.61 | -4.32 | -1.83 | -4.32 | -5.09 | -2.87 | -3.62 | -5.98 | -4.80 | -7.21 | -8.84 | -1.53 | -22.46 | -1.59 | NA | -10.40 | -9.29 | -8.53 | -9.62 | -10.55 | -13.98 | -1.91 |
| Karelia | 13.00 | 11.48 | 1.19 | 5.26 | 0.14 | -9.50 | -6.98 | -9.63 | -10.38 | -9.45 | -9.79 | -18.31 | -11.60 | -17.20 | -19.05 | -12.86 | -11.76 | -11.51 | -13.46 | NA | -19.73 | -18.66 | -21.54 | -26.14 | -13.99 | -9.51 |
| Loschbour | 14.00 | 12.63 | -0.57 | 5.71 | 1.11 | -12.16 | -12.26 | -14.50 | -14.05 | -9.15 | -14.73 | -10.38 | -24.95 | -39.05 | -42.52 | -5.86 | -11.14 | -4.21 | -12.89 | -20.74 | NA | -37.32 | -35.09 | -31.18 | -18.45 | -3.94 |
| LaBrana1 | 13.66 | 12.45 | -0.17 | 6.27 | 1.32 | -11.39 | -13.22 | -13.64 | -13.90 | -8.95 | -12.63 | -9.19 | -27.08 | -33.15 | -36.21 | -5.51 | -10.13 | -3.84 | -12.11 | -19.23 | -38.03 | NA | -30.22 | -27.96 | -17.17 | -4.26 |
| Hungarian.KO1 | 12.87 | 11.63 | 0.67 | 4.43 | 1.42 | -10.73 | -8.22 | -11.63 | -12.39 | -8.05 | -12.48 | -9.68 | -17.65 | -31.76 | -31.32 | -4.97 | -10.74 | -4.70 | -12.85 | -21.12 | -33.89 | -28.81 | NA | -27.21 | -18.28 | -4.03 |
| Motala12 | 14.56 | 13.14 | 0.45 | 5.35 | 2.08 | -10.24 | -8.58 | -11.26 | -10.68 | -7.65 | -11.72 | -13.24 | -16.45 | -26.65 | -27.73 | -7.53 | -10.81 | -5.86 | -13.88 | -25.70 | -30.31 | -26.02 | -27.39 | NA | -17.29 | -4.29 |
| LBK | 15.66 | 15.65 | 2.74 | 7.69 | 5.46 | -5.73 | -4.07 | -8.41 | -7.17 | -3.82 | -6.48 | -4.06 | -8.11 | -11.99 | -12.74 | -0.79 | -12.39 | -0.07 | -14.93 | -11.52 | -14.02 | -12.59 | -14.95 | -14.10 | NA | -0.89 |
| Saqqaq | 11.08 | 10.45 | -0.18 | 4.72 | -6.10 | -2.71 | -1.04 | -3.55 | -3.11 | -7.17 | -1.68 | -9.12 | -2.88 | -2.66 | -5.36 | -20.90 | -4.61 | -24.84 | -6.90 | -11.56 | -5.29 | -5.61 | -6.02 | -6.87 | -5.37 | NA |

**Table S5.11 Z-scores of the statistics *D(Bacho Kiro BK1653, X; Y Mbuti)*.** Standard errors (SE) were computed using a Weighted Block Jackknife[3,12] across all autosomes of the "2200k" Panel and using a block size of 5Mb. Blue: Z-score ≤ -3; Yellow: Z-score ≥ 3.

| X/Y | BachoKiro_F6_620 | BachoKiro_BB7_240 | BachoKiro_CC7_335 | UstIshim | Oase1 | Tianyuan | Kostenki14 | GoyetQ116-1 | SunghirIII | Yana_old | Vestonice16 | Malta1 | ElMiron | Villabruna | Bichon | Clovis | Satsurblia | Kolyma_River | Kotias | Karelia | Loschbour | LaBrana1 | Hungarian.KO1 | Motala12 | LBK | Saqqaq |
|---|---|---|---|---|---|---|---|---|---|---|---|---|---|---|---|---|---|---|---|---|---|---|---|---|---|---|
| BachoKiro_F6_620 | NA | -8.50 | -11.46 | 0.18 | -5.52 | -0.98 | 13.16 | 13.34 | 12.38 | 7.61 | 17.39 | 7.00 | 14.48 | 15.82 | 15.72 | 1.84 | 7.28 | 2.06 | 7.82 | 10.02 | 15.60 | 16.72 | 13.01 | 13.83 | 12.34 | 0.55 |
| BachoKiro_BB7_240 | -10.69 | NA | -12.45 | -0.60 | -6.31 | -3.07 | 11.26 | 9.89 | 11.36 | 7.10 | 13.61 | 6.45 | 11.93 | 12.41 | 11.58 | 0.61 | 5.04 | 1.09 | 6.56 | 9.19 | 13.26 | 12.79 | 10.12 | 10.88 | 10.93 | -0.34 |
| BachoKiro_CC7_335 | -11.31 | -10.34 | NA | -0.74 | -5.57 | -2.65 | 10.45 | 10.12 | 10.94 | 7.30 | 13.76 | 5.34 | 11.43 | 11.98 | 11.70 | 0.83 | 4.14 | 0.36 | 6.24 | 8.73 | 12.38 | 12.21 | 10.47 | 10.68 | 9.74 | 0.45 |
| UstIshim | -0.91 | 0.11 | -2.24 | NA | 0.07 | -1.05 | 9.13 | 9.93 | 9.58 | 6.37 | 13.33 | 5.26 | 12.42 | 12.77 | 11.52 | 0.51 | 3.80 | -0.22 | 5.73 | 9.84 | 12.46 | 13.21 | 12.00 | 10.53 | 9.32 | 0.51 |
| Oase1 | -3.33 | -4.24 | -4.18 | 2.28 | NA | 0.43 | 11.87 | 11.80 | 11.53 | 8.32 | 15.02 | 7.46 | 12.62 | 11.89 | 13.70 | 2.30 | 5.08 | 2.26 | 7.80 | 9.42 | 14.52 | 12.98 | 10.09 | 11.06 | 10.24 | 2.29 |
| Tianyuan | -2.63 | -1.94 | -4.13 | -1.33 | -2.03 | NA | 10.70 | 9.74 | 9.04 | 2.58 | 14.92 | 4.11 | 11.78 | 12.36 | 10.88 | -6.10 | 4.61 | -7.26 | 5.12 | 6.54 | 11.69 | 12.26 | 10.37 | 9.97 | 10.10 | -8.29 |
| Kostenki14 | 0.56 | 0.80 | -1.37 | -2.20 | -0.25 | -0.10 | NA | 2.27 | -5.26 | -2.02 | 1.98 | -1.88 | 2.41 | 2.58 | 1.59 | -1.22 | -2.09 | -1.10 | -1.37 | 0.25 | 0.96 | 2.06 | 0.54 | 1.45 | 2.08 | -0.64 |
| GoyetQ116-1 | -1.99 | -3.41 | -4.20 | -4.10 | -2.38 | -4.18 | -1.08 | NA | -1.18 | -2.36 | 2.73 | -2.84 | -8.14 | 0.66 | -1.19 | -2.05 | -1.81 | -3.29 | -1.95 | -0.88 | -2.87 | -3.68 | -1.11 | -1.24 | 0.62 | -3.39 |
| SunghirIII | -0.15 | 0.40 | -1.13 | -2.28 | -0.69 | -2.19 | -5.24 | 2.01 | NA | 0.38 | -3.03 | -1.81 | 1.28 | 1.35 | -0.84 | -1.09 | -2.33 | -1.64 | -2.19 | -0.92 | -1.18 | -0.21 | -1.52 | 0.21 | 0.38 | -1.71 |
| Yana_old | -1.81 | 0.52 | -1.48 | -1.51 | -0.29 | -5.30 | 2.11 | 4.79 | 4.32 | NA | 6.89 | -4.28 | 6.23 | 6.15 | 5.27 | -5.51 | -0.25 | -5.09 | -0.04 | -0.04 | 4.32 | 5.19 | 3.22 | 3.44 | 4.23 | -5.94 |
| Vestonice16 | 0.30 | -0.61 | -2.30 | -2.37 | -0.52 | -1.23 | -3.66 | 1.48 | -8.08 | -2.59 | NA | -3.15 | -1.99 | -2.79 | -2.63 | -1.53 | -2.07 | -1.65 | -1.99 | -2.03 | -3.90 | -1.38 | -3.98 | -2.24 | -0.15 | -1.43 |
| Malta1 | -1.10 | 0.03 | -2.89 | -2.77 | -1.47 | -3.49 | 2.34 | 4.06 | 2.71 | -3.85 | 6.19 | NA | 4.39 | 3.68 | 1.70 | -12.94 | -3.96 | -10.48 | -4.45 | -11.12 | 1.41 | 2.68 | -0.83 | -5.23 | 1.67 | -9.30 |
| ElMiron | -1.22 | -0.69 | -2.76 | -1.58 | -1.41 | -2.15 | -0.73 | -7.10 | -1.68 | -1.00 | 0.03 | -1.47 | NA | -11.41 | -14.37 | -3.87 | -2.32 | -2.71 | -4.26 | -4.91 | -16.04 | -16.89 | -10.69 | -8.67 | -3.03 | -3.14 |
| Villabruna | 0.16 | -0.43 | -2.43 | -1.64 | -2.53 | -1.62 | 0.01 | 2.43 | -0.94 | -0.67 | 0.49 | -2.64 | -9.86 | NA | -26.05 | -3.11 | -6.08 | -3.24 | -6.57 | -9.70 | -28.64 | -20.91 | -24.32 | -19.05 | -6.95 | -2.92 |
| Bichon | 1.07 | 0.20 | -1.79 | -2.12 | -0.65 | -1.92 | 0.16 | 1.02 | -2.02 | -0.34 | 1.18 | -4.11 | -12.08 | -24.52 | NA | -3.80 | -7.50 | -4.57 | -6.75 | -10.22 | -29.10 | -21.73 | -23.06 | -18.35 | -6.88 | -4.19 |
| Clovis | -1.83 | -0.45 | -2.66 | -2.13 | -1.81 | -7.90 | 8.71 | 11.23 | 9.17 | 1.20 | 13.06 | -6.36 | 8.86 | 9.61 | 8.39 | NA | -1.06 | -21.49 | 0.63 | -4.69 | 7.71 | 8.39 | 4.96 | 2.02 | 6.02 | -21.17 |
| Satsurblia | 3.90 | 3.71 | 1.22 | 1.42 | 1.64 | 2.97 | 7.52 | 11.17 | 7.91 | 6.36 | 12.55 | 1.47 | 10.26 | 6.36 | 5.18 | -1.08 | NA | -0.42 | -19.17 | -0.11 | 4.68 | 7.04 | 2.37 | 2.83 | -1.92 | 0.16 |
| Kolyma_River | -2.02 | -0.42 | -3.41 | -3.11 | -2.67 | -9.62 | 8.65 | 10.13 | 8.35 | 1.45 | 13.85 | -4.50 | 9.82 | 9.80 | 7.72 | -21.97 | -1.18 | NA | -0.37 | -3.86 | 8.10 | 9.15 | 5.01 | 3.16 | 6.20 | -26.61 |
| Kotias | 2.63 | 3.17 | 1.52 | 1.84 | 1.83 | 1.73 | 7.73 | 9.72 | 6.89 | 5.14 | 12.63 | -0.02 | 8.03 | 6.04 | 4.46 | -0.90 | -21.17 | -1.49 | NA | -0.98 | 3.98 | 4.78 | 1.75 | 0.50 | -3.75 | -1.63 |
| Karelia | 0.02 | 0.85 | -1.05 | -0.14 | -0.20 | -2.69 | 2.92 | 4.71 | 2.00 | -1.18 | 6.01 | -12.70 | 1.42 | -4.46 | -5.88 | -12.19 | -8.28 | -12.01 | -7.75 | NA | -6.97 | -5.15 | -9.66 | -14.46 | -3.42 | -10.20 |
| Loschbour | 0.69 | 0.84 | -1.48 | -2.10 | -0.18 | -1.93 | -0.52 | -0.92 | -2.47 | -1.27 | 0.20 | -4.46 | -14.15 | -26.37 | -29.39 | -5.21 | -7.47 | -4.03 | -7.58 | -12.21 | NA | -25.94 | -25.40 | -21.39 | -8.01 | -4.42 |
| LaBrana1 | 0.89 | 0.79 | -1.34 | -1.51 | 0.17 | -1.19 | 0.26 | -1.35 | -1.65 | -0.79 | 2.43 | -3.84 | -13.94 | -18.70 | -22.82 | -4.25 | -5.25 | -3.89 | -6.57 | -10.11 | -24.34 | NA | -19.30 | -15.47 | -5.44 | -4.43 |
| Hungarian.KO1 | 0.86 | 0.82 | -1.07 | -0.22 | -0.68 | -1.32 | 0.77 | 2.89 | -0.85 | -0.14 | 1.86 | -4.58 | -7.00 | -21.07 | -21.15 | -4.66 | -6.94 | -5.17 | -7.48 | -12.67 | -23.77 | -18.44 | NA | -19.19 | -8.98 | -4.62 |
| Motala12 | 1.35 | 1.37 | -0.63 | -1.37 | -0.50 | -1.22 | 2.49 | 3.40 | 1.30 | 0.12 | 4.07 | -8.62 | -4.34 | -15.10 | -15.61 | -7.40 | -7.41 | -6.59 | -8.28 | -16.95 | -17.94 | -12.82 | -17.86 | NA | -7.60 | -6.02 |
| LBK | 2.47 | 3.85 | 1.19 | 1.23 | 1.81 | 2.42 | 6.54 | 8.93 | 5.26 | 4.78 | 10.27 | 1.95 | 5.26 | 1.13 | 0.16 | -0.06 | -8.59 | 0.24 | -8.55 | -1.16 | -0.61 | 1.86 | -3.86 | -2.79 | NA | -1.11 |
| Saqqaq | -2.89 | -1.56 | -3.25 | -1.85 | -1.75 | -9.78 | 9.27 | 10.59 | 8.83 | 0.90 | 14.56 | -2.43 | 10.78 | 10.98 | 8.62 | -20.57 | 0.19 | -25.59 | 0.01 | -1.60 | 8.33 | 8.81 | 5.53 | 4.39 | 5.02 | NA |

**Table S5.12 Z-scores of the statistics *D(X,Y; Bacho Kiro F6-620, Mbuti)*.** Standard errors (SE) were computed using a Weighted Block Jackknife[3,12] across all autosomes of the "2200k" Panel and using a block size of 5Mb. Blue: Z-score $\leq$ -3; Yellow: Z-score $\geq$ 3.

| X/Y | BachoKiro_BB7_240 | BachoKiro_CC7_335 | UstIshim | Oase1 | Tianyuan | Kostenki14 | GoyetQ116-1 | BachoKiro_BK_1653 | SunghirIII | Yana_old | Vestonice16 | Malta1 | ElMiron | Villabruna | Bichon | Clovis | Satsurblia | Kolyma_River | Kotias | Karelia | Loschbour | LaBrana1 | Hungarian.KO1 | Motala12 | LBK | Saqqaq |
|---|---|---|---|---|---|---|---|---|---|---|---|---|---|---|---|---|---|---|---|---|---|---|---|---|---|---|
| BachoKiro_BB7_240 | NA | -1.95 | 9.42 | 5.28 | 8.56 | 11.45 | 9.13 | 10.69 | 11.59 | 9.71 | 10.79 | 10.01 | 10.16 | 11.14 | 12.24 | 9.51 | 13.75 | 9.70 | 13.18 | 10.24 | 11.56 | 11.96 | 11.52 | 12.43 | 13.14 | 8.85 |
| BachoKiro_CC7_335 | 1.95 | NA | 11.04 | 6.88 | 9.32 | 13.68 | 10.51 | 11.31 | 13.39 | 12.14 | 12.42 | 11.46 | 10.60 | 13.20 | 13.76 | 11.54 | 15.43 | 12.09 | 15.03 | 13.00 | 14.00 | 13.66 | 12.87 | 14.56 | 15.66 | 11.08 |
| UstIshim | -9.42 | -11.04 | NA | -2.92 | -2.26 | 1.94 | -0.16 | 0.91 | 1.09 | -0.50 | 1.57 | 0.19 | 0.55 | 1.61 | 2.06 | -1.09 | 4.73 | -1.11 | 3.47 | 0.68 | 1.99 | 1.83 | 1.41 | 2.20 | 3.37 | -1.81 |
| Oase1 | -5.28 | -6.88 | 2.92 | NA | 0.94 | 4.26 | 2.20 | 3.33 | 3.88 | 2.38 | 3.37 | 2.39 | 3.01 | 3.74 | 4.97 | 2.31 | 7.11 | 2.78 | 5.67 | 3.60 | 5.30 | 4.92 | 3.87 | 4.47 | 6.47 | 2.28 |
| Tianyuan | -8.56 | -9.32 | 2.26 | -0.94 | NA | 4.21 | 1.85 | 2.63 | 3.33 | 1.58 | 3.29 | 2.06 | 2.74 | 3.68 | 4.45 | 1.34 | 6.99 | 1.54 | 6.13 | 3.32 | 4.21 | 4.32 | 4.17 | 4.34 | 5.93 | 0.61 |
| Kostenki14 | -11.45 | -13.68 | -1.94 | -4.26 | -4.21 | NA | -3.24 | -0.56 | -0.87 | -2.72 | -0.83 | -2.07 | -2.00 | -0.84 | 0.19 | -3.26 | 2.93 | -3.07 | 2.21 | -1.44 | 0.26 | -0.07 | -0.32 | 0.24 | 1.91 | -3.96 |
| GoyetQ116-1 | -9.13 | -10.51 | 0.16 | -2.20 | -1.85 | 3.24 | NA | 1.99 | 2.08 | 0.15 | 2.63 | 0.80 | 1.59 | 2.40 | 3.03 | -0.07 | 5.74 | -0.43 | 5.20 | 1.41 | 3.06 | 2.94 | 2.36 | 2.77 | 5.04 | -1.03 |
| BachoKiro_BK_1653 | -10.69 | -11.31 | -0.91 | -3.33 | -2.63 | 0.56 | -1.99 | NA | -0.15 | -1.71 | 0.30 | -1.10 | -1.22 | 0.16 | 1.07 | -1.83 | 3.90 | -2.02 | 2.63 | 0.02 | 0.69 | 0.89 | 0.86 | 1.35 | 2.47 | -2.89 |
| SunghirIII | -11.59 | -13.39 | -1.09 | -3.88 | -3.33 | 0.87 | -2.08 | 0.15 | NA | -1.78 | 0.32 | -1.06 | -0.41 | 0.46 | 1.16 | -2.36 | 4.14 | -2.43 | 2.76 | -0.59 | 1.06 | 0.89 | 0.65 | 1.38 | 2.61 | -3.12 |
| Yana_old | -9.71 | -12.14 | 0.50 | -2.38 | -1.58 | 2.72 | -0.15 | 1.71 | 1.78 | NA | 2.00 | 0.62 | 1.33 | 2.45 | 2.96 | -0.59 | 5.44 | -0.63 | 4.39 | 1.35 | 2.84 | 2.66 | 2.60 | 3.06 | 4.23 | -1.38 |
| Vestonice16 | -10.79 | -12.42 | -1.57 | -3.37 | -3.29 | 0.83 | -2.63 | -0.30 | -0.32 | -2.00 | NA | -1.07 | -0.95 | -0.14 | 0.73 | -2.19 | 4.04 | -2.32 | 2.66 | -0.74 | 0.60 | 0.78 | 0.98 | 0.84 | 2.60 | -2.97 |
| Malta1 | -10.01 | -11.46 | -0.19 | -2.39 | -2.06 | 2.07 | -0.80 | 1.10 | 1.06 | -0.62 | 1.07 | NA | 1.07 | 1.40 | 2.05 | -1.37 | 4.50 | -1.27 | 3.98 | 0.56 | 1.99 | 2.07 | 1.67 | 2.22 | 3.75 | -2.52 |
| ElMiron | -10.16 | -10.60 | -0.55 | -3.01 | -2.74 | 2.00 | -1.59 | 1.22 | 0.41 | -1.33 | 0.95 | -1.07 | NA | 1.05 | 2.07 | -1.55 | 4.15 | -1.44 | 3.94 | 0.36 | 1.51 | 2.03 | 2.02 | 1.94 | 4.13 | -2.29 |
| Villabruna | -11.14 | -13.20 | -1.61 | -3.74 | -3.68 | 0.84 | -2.40 | -0.16 | -0.46 | -2.45 | 0.14 | -1.40 | -1.05 | NA | 0.82 | -2.61 | 3.61 | -2.94 | 2.91 | -1.14 | 0.49 | 0.85 | 0.28 | 1.10 | 2.66 | -3.34 |
| Bichon | -12.24 | -13.76 | -2.06 | -4.97 | -4.45 | -0.19 | -3.03 | -1.07 | -1.16 | -2.96 | -0.73 | -2.05 | -2.07 | -0.82 | NA | -3.68 | 2.84 | -3.77 | 1.89 | -1.87 | -0.17 | -0.48 | -0.58 | 0.15 | 1.56 | -4.29 |
| Clovis | -9.51 | -11.54 | 1.09 | -2.31 | -1.34 | 3.26 | 0.07 | 1.83 | 2.36 | 0.59 | 2.19 | 1.37 | 1.55 | 2.61 | 3.68 | NA | 6.39 | -0.05 | 5.45 | 2.19 | 3.68 | 3.53 | 3.16 | 3.78 | 5.41 | -1.12 |
| Satsurblia | -13.75 | -15.43 | -4.73 | -7.11 | -6.99 | -2.93 | -5.74 | -3.90 | -4.14 | -5.44 | -4.04 | -4.50 | -4.15 | -3.61 | -2.84 | -6.39 | NA | -6.56 | -1.53 | -5.20 | -3.21 | -3.11 | -3.39 | -3.09 | -1.76 | -7.32 |
| Kolyma_River | -9.70 | -12.09 | 1.11 | -2.78 | -1.54 | 3.07 | 0.43 | 2.02 | 2.43 | 0.63 | 2.32 | 1.27 | 1.44 | 2.94 | 3.77 | 0.05 | 6.56 | NA | 5.21 | 2.08 | 3.61 | 3.47 | 3.26 | 3.86 | 5.29 | -1.18 |
| Kotias | -13.18 | -15.03 | -3.47 | -5.67 | -6.13 | -2.21 | -5.20 | -2.63 | -2.76 | -4.39 | -2.66 | -3.98 | -3.94 | -2.91 | -1.89 | -5.45 | 1.53 | -5.21 | NA | -4.00 | -1.85 | -2.32 | -2.67 | -1.96 | -0.35 | -6.12 |
| Karelia | -10.24 | -13.00 | -0.68 | -3.60 | -3.32 | 1.44 | -1.41 | -0.02 | 0.59 | -1.35 | 0.74 | -0.56 | -0.36 | 1.14 | 1.87 | -2.19 | 5.20 | -2.08 | 4.00 | NA | 1.72 | 1.70 | 1.51 | 1.93 | 3.46 | -2.99 |
| Loschbour | -11.56 | -14.00 | -1.99 | -5.30 | -4.21 | -0.26 | -3.06 | -0.69 | -1.06 | -2.84 | -0.60 | -1.99 | -1.51 | -0.49 | 0.17 | -3.68 | 3.21 | -3.61 | 1.85 | -1.72 | NA | -0.17 | -0.61 | 0.31 | 1.70 | -4.46 |
| LaBrana1 | -11.96 | -13.66 | -1.83 | -4.92 | -4.32 | 0.07 | -2.94 | -0.89 | -0.89 | -2.66 | -0.78 | -2.07 | -2.03 | -0.85 | 0.48 | -3.53 | 3.11 | -3.47 | 2.32 | -1.70 | 0.17 | NA | -0.34 | 0.47 | 1.92 | -4.27 |
| Hungarian.KO1 | -11.52 | -12.87 | -1.41 | -3.87 | -4.17 | 0.32 | -2.36 | -0.86 | -0.65 | -2.60 | -0.98 | -1.67 | -2.02 | -0.28 | 0.58 | -3.16 | 3.39 | -3.26 | 2.67 | -1.51 | 0.61 | 0.34 | NA | 0.58 | 2.19 | -4.01 |
| Motala12 | -12.43 | -14.56 | -2.20 | -4.47 | -4.34 | -0.24 | -2.77 | -1.35 | -1.38 | -3.06 | -0.84 | -2.22 | -1.94 | -1.10 | -0.15 | -3.78 | 3.09 | -3.86 | 1.96 | -1.93 | -0.31 | -0.47 | -0.58 | NA | 1.86 | -4.47 |
| LBK | -13.14 | -15.66 | -3.37 | -6.47 | -5.93 | -1.91 | -5.04 | -2.47 | -2.61 | -4.23 | -2.60 | -3.75 | -4.13 | -2.66 | -1.56 | -5.41 | 1.76 | -5.29 | 0.35 | -3.46 | -1.70 | -1.92 | -2.19 | -1.86 | NA | -6.14 |
| Saqqaq | -8.85 | -11.08 | 1.81 | -2.28 | -0.61 | 3.96 | 1.03 | 2.89 | 3.12 | 1.38 | 2.97 | 2.52 | 2.29 | 3.34 | 4.29 | 1.12 | 7.32 | 1.18 | 6.12 | 2.99 | 4.46 | 4.27 | 4.01 | 4.47 | 6.14 | NA |

**Table S5.13 Z-scores of the statistics *D(X,Y; Bacho Kiro BB7-240, Mbuti)*.** Standard errors (SE) were computed using a Weighted Block Jackknife[3,12] across all autosomes of the "2200k" Panel and using a block size of 5Mb. Blue: Z-score ≤ -3; Yellow: Z-score ≥ 3.

| X/Y | BachoKiro_F6_620 | BachoKiro_CC7_335 | UstIshim | Oase1 | Tianyuan | Kostenki14 | GoyetQ116-1 | BachoKiro_BK_1653 | SunghirIII | Yana_old | Vestonice16 | Malta1 | ElMiron | Villabruna | Bichon | Clovis | Satsurblia | Kolyma_River | Kotias | Karelia | Loschbour | LaBrana1 | Hungarian.KO1 | Motala12 | LBK | Saqqaq |
|---|---|---|---|---|---|---|---|---|---|---|---|---|---|---|---|---|---|---|---|---|---|---|---|---|---|
| BachoKiro_F6_620 | NA | -2.52 | 8.67 | 4.64 | 6.79 | 8.98 | 5.77 | 8.50 | 9.14 | 9.48 | 7.73 | 9.25 | 7.80 | 8.39 | 8.66 | 8.41 | 12.59 | 8.91 | 11.61 | 9.56 | 9.40 | 9.65 | 9.54 | 10.64 | 12.58 | 8.26 |
| BachoKiro_CC7_335 | 2.52 | NA | 11.00 | 5.55 | 9.40 | 12.01 | 7.96 | 10.34 | 12.46 | 11.58 | 10.46 | 10.86 | 10.47 | 11.92 | 11.74 | 10.63 | 14.99 | 11.65 | 14.67 | 11.48 | 12.63 | 12.45 | 11.63 | 13.14 | 15.65 | 10.45 |
| UstIshim | -8.67 | -11.00 | NA | -3.91 | -2.38 | 0.59 | -2.97 | -0.11 | -0.02 | 0.37 | -0.51 | -0.15 | -0.94 | -0.28 | -0.34 | -0.86 | 3.53 | -0.22 | 2.76 | 0.45 | 0.55 | 0.74 | 0.53 | 1.37 | 3.36 | -1.21 |
| Oase1 | -4.64 | -5.55 | 3.91 | NA | 1.52 | 3.95 | 1.01 | 4.24 | 3.88 | 3.84 | 2.65 | 3.12 | 2.47 | 3.25 | 4.11 | 2.84 | 5.73 | 3.66 | 5.71 | 4.70 | 4.32 | 4.33 | 3.75 | 4.95 | 6.69 | 2.84 |
| Tianyuan | -6.79 | -9.40 | 2.38 | -1.52 | NA | 3.35 | -0.68 | 1.94 | 2.84 | 2.85 | 1.66 | 2.09 | 1.54 | 2.05 | 2.16 | 1.87 | 6.03 | 2.48 | 5.34 | 3.12 | 3.20 | 3.30 | 2.99 | 3.62 | 6.16 | 1.55 |
| Kostenki14 | -8.98 | -12.01 | -0.59 | -3.95 | -3.35 | NA | -4.25 | -0.80 | -0.81 | -0.26 | -1.80 | -0.90 | -1.85 | -1.60 | -1.01 | -1.66 | 3.23 | -0.91 | 2.40 | -0.19 | 0.02 | 0.23 | -0.33 | 0.50 | 3.07 | -1.94 |
| GoyetQ116-1 | -5.77 | -7.96 | 2.97 | -1.01 | 0.68 | 4.25 | NA | 3.41 | 3.46 | 3.61 | 2.46 | 2.71 | 2.91 | 2.98 | 2.98 | 2.48 | 7.15 | 2.65 | 5.97 | 3.73 | 4.04 | 4.39 | 4.03 | 4.33 | 7.62 | 1.98 |
| BachoKiro_BK_1653 | -8.50 | -10.34 | 0.11 | -4.24 | -1.94 | 0.80 | -3.41 | NA | 0.40 | 0.52 | -0.61 | 0.03 | -0.69 | -0.43 | 0.20 | -0.45 | 3.71 | -0.42 | 3.17 | 0.85 | 0.84 | 0.79 | 0.82 | 1.37 | 3.85 | -1.56 |
| SunghirIII | -9.14 | -12.46 | 0.02 | -3.88 | -2.84 | 0.81 | -3.46 | -0.40 | NA | 0.41 | -0.53 | -0.51 | -0.84 | -0.44 | -0.38 | -1.00 | 4.38 | -0.22 | 3.10 | 0.47 | 0.73 | 0.87 | 1.11 | 1.84 | 4.15 | -1.50 |
| Yana_old | -9.48 | -11.58 | -0.37 | -3.84 | -2.85 | 0.26 | -3.61 | -0.52 | -0.41 | NA | -1.20 | -0.64 | -1.85 | -0.95 | -0.78 | -1.33 | 3.66 | -0.65 | 2.70 | 0.10 | 0.22 | 0.52 | 0.24 | 0.96 | 3.52 | -1.75 |
| Vestonice16 | -7.73 | -10.46 | 0.51 | -2.65 | -1.66 | 1.80 | -2.46 | 0.61 | 0.53 | 1.20 | NA | 0.09 | 0.16 | 0.23 | 0.85 | -0.46 | 4.58 | 0.33 | 3.69 | 1.55 | 1.46 | 1.82 | 1.91 | 2.12 | 4.60 | -0.36 |
| Malta1 | -9.25 | -10.86 | 0.15 | -3.12 | -2.09 | 0.90 | -2.71 | -0.03 | 0.51 | 0.64 | -0.09 | NA | -0.37 | -0.01 | -0.39 | -0.84 | 4.11 | -0.17 | 3.25 | 0.76 | 0.86 | 1.41 | 0.97 | 1.46 | 4.26 | -0.69 |
| ElMiron | -7.80 | -10.47 | 0.94 | -2.47 | -1.54 | 1.85 | -2.91 | 0.69 | 0.84 | 1.85 | -0.16 | 0.37 | NA | 0.02 | 0.67 | 0.07 | 4.17 | 0.57 | 3.67 | 1.57 | 1.78 | 1.86 | 1.37 | 2.20 | 5.07 | -0.65 |
| Villabruna | -8.39 | -11.92 | 0.28 | -3.25 | -2.05 | 1.60 | -2.98 | 0.43 | 0.44 | 0.95 | -0.23 | 0.01 | -0.02 | NA | 0.21 | -0.63 | 4.64 | -0.35 | 3.61 | 0.91 | 1.14 | 1.73 | 1.51 | 1.87 | 4.79 | -1.01 |
| Bichon | -8.66 | -11.74 | 0.34 | -4.11 | -2.16 | 1.01 | -2.98 | -0.20 | 0.38 | 0.78 | -0.85 | 0.39 | -0.67 | -0.21 | NA | -0.56 | 4.50 | 0.13 | 3.70 | 1.00 | 1.23 | 1.64 | 1.11 | 2.19 | 4.71 | -0.94 |
| Clovis | -8.41 | -10.63 | 0.86 | -2.84 | -1.87 | 1.66 | -2.48 | 0.45 | 1.00 | 1.33 | 0.46 | 0.84 | -0.07 | 0.63 | 0.56 | NA | 5.59 | 0.84 | 4.21 | 1.78 | 1.69 | 1.97 | 1.82 | 2.59 | 5.17 | -0.35 |
| Satsurblia | -12.59 | -14.99 | -3.53 | -5.73 | -6.03 | -3.23 | -7.15 | -3.71 | -4.38 | -3.66 | -4.58 | -4.11 | -4.17 | -4.64 | -4.50 | -5.59 | NA | -4.73 | -1.24 | -3.87 | -3.90 | -3.27 | -3.62 | -3.60 | -0.46 | -6.03 |
| Kolyma_River | -8.91 | -11.65 | 0.22 | -3.66 | -2.48 | 0.91 | -2.65 | 0.42 | 0.22 | 0.65 | -0.33 | 0.17 | -0.57 | 0.35 | -0.13 | -0.84 | 4.73 | NA | 3.41 | 0.86 | 0.90 | 1.11 | 1.04 | 1.79 | 4.27 | -1.36 |
| Kotias | -11.61 | -14.67 | -2.76 | -5.71 | -5.34 | -2.40 | -5.97 | -3.17 | -3.10 | -2.70 | -3.69 | -3.25 | -3.67 | -3.61 | -3.70 | -4.21 | 1.24 | -3.41 | NA | -2.99 | -2.64 | -2.66 | -2.98 | -1.89 | 0.71 | -4.59 |
| Karelia | -9.56 | -11.48 | -0.45 | -4.70 | -3.12 | 0.19 | -3.73 | -0.85 | -0.47 | -0.10 | -1.55 | -0.76 | -1.57 | -0.91 | -1.00 | -1.78 | 3.87 | -0.86 | 2.99 | NA | 0.19 | 0.67 | 0.08 | 0.91 | 3.42 | -1.98 |
| Loschbour | -9.40 | -12.63 | -0.55 | -4.32 | -3.20 | -0.02 | -4.04 | -0.84 | -0.73 | -0.22 | -1.46 | -0.86 | -1.78 | -1.14 | -1.23 | -1.69 | 3.90 | -0.90 | 2.64 | -0.19 | NA | 0.39 | -0.22 | 0.98 | 3.76 | -2.13 |
| LaBrana1 | -9.65 | -12.45 | -0.74 | -4.33 | -3.30 | -0.23 | -4.39 | -0.79 | -0.87 | -0.52 | -1.82 | -1.41 | -1.86 | -1.73 | -1.64 | -1.97 | 3.27 | -1.11 | 2.66 | -0.67 | -0.39 | NA | -0.29 | 0.56 | 3.35 | -2.29 |
| Hungarian.KO1 | -9.54 | -11.63 | -0.53 | -3.75 | -2.99 | 0.33 | -4.03 | -0.82 | -1.11 | -0.24 | -1.91 | -0.97 | -1.37 | -1.51 | -1.11 | -1.82 | 3.62 | -1.04 | 2.98 | -0.08 | 0.22 | 0.29 | NA | 1.22 | 4.01 | -1.95 |
| Motala12 | -10.64 | -13.14 | -1.37 | -4.95 | -3.62 | -0.50 | -4.33 | -1.37 | -1.84 | -0.96 | -2.12 | -1.46 | -2.20 | -1.87 | -2.19 | -2.59 | 3.60 | -1.79 | 1.89 | -0.91 | -0.98 | -0.56 | -1.22 | NA | 2.79 | -3.23 |
| LBK | -12.58 | -15.65 | -3.36 | -6.69 | -6.16 | -3.07 | -7.62 | -3.85 | -4.15 | -3.52 | -4.60 | -4.26 | -5.07 | -4.79 | -4.71 | -5.17 | 0.46 | -4.27 | -0.71 | -3.42 | -3.76 | -3.35 | -4.01 | -2.79 | NA | -5.62 |
| Saqqaq | -8.26 | -10.45 | 1.21 | -2.84 | -1.55 | 1.94 | -1.98 | 1.56 | 1.50 | 1.75 | 0.36 | 0.69 | 0.65 | 1.01 | 0.94 | 0.35 | 6.03 | 1.36 | 4.59 | 1.98 | 2.13 | 2.29 | 1.95 | 3.23 | 5.62 | NA |

**Table S5.14 Z-scores of the statistics *D(X,Y; Bacho Kiro CC7-335, Mbuti)*.** Standard errors (SE) were computed using a Weighted Block Jackknife[3,12] across all autosomes of the "2200k" Panel and using a block size of 5Mb. Blue: Z-score ≤ -3; Yellow: Z-score ≥ 3.

| X/Y | BachoKiro_F6_620 | BachoKiro_BB7_240 | UstIshim | Oase1 | Tianyuan | Kostenki14 | GoyetQ116-1 | BachoKiro_BK_1653 | SunghirIII | Yana_old | Vestonice16 | Malta1 | ElMiron | Villabruna | Bichon | Clovis | Satsurblia | Kolyma_River | Kotias | Karelia | Loschbour | LaBrana1 | Hungarian.KO1 | Motala12 | LBK | Saqqaq |
|---|---|---|---|---|---|---|---|---|---|---|---|---|---|---|---|---|---|---|---|---|---|---|---|---|---|---|
| BachoKiro_F6_620 | NA | -0.58 | 9.63 | 6.66 | 8.30 | 11.46 | 7.66 | 11.46 | 11.47 | 11.55 | 9.56 | 10.19 | 9.32 | 10.27 | 11.09 | 10.38 | 12.99 | 9.96 | 14.34 | 11.64 | 11.06 | 11.46 | 11.18 | 12.21 | 13.58 | 10.27 |
| BachoKiro_BB7_240 | 0.58 | NA | 10.87 | 6.62 | 9.95 | 11.97 | 9.00 | 12.45 | 13.38 | 12.44 | 10.70 | 10.92 | 10.28 | 11.70 | 12.18 | 11.50 | 13.66 | 11.18 | 15.11 | 11.86 | 12.82 | 12.54 | 11.86 | 13.29 | 14.70 | 11.16 |
| UstIshim | -9.63 | -10.87 | NA | -2.36 | -1.71 | 1.05 | -1.94 | 2.24 | 1.35 | 0.80 | -0.23 | -0.31 | -0.36 | 0.08 | 1.03 | 0.01 | 3.50 | -0.55 | 3.99 | 1.55 | 1.27 | 0.95 | 1.31 | 2.08 | 3.42 | -0.28 |
| Oase1 | -6.66 | -6.62 | 2.36 | NA | 1.11 | 3.56 | 0.43 | 4.18 | 3.22 | 3.06 | 2.04 | 2.33 | 1.72 | 2.52 | 3.31 | 2.50 | 4.77 | 2.04 | 5.39 | 4.03 | 2.97 | 2.94 | 3.03 | 3.29 | 5.31 | 2.36 |
| Tianyuan | -8.30 | -9.95 | 1.71 | -1.11 | NA | 3.17 | -0.17 | 4.13 | 3.14 | 2.98 | 1.57 | 1.28 | 1.40 | 2.10 | 2.79 | 1.90 | 4.81 | 1.43 | 6.14 | 3.35 | 3.24 | 3.24 | 3.70 | 4.13 | 5.80 | 1.86 |
| Kostenki14 | -11.46 | -11.97 | -1.05 | -3.56 | -3.17 | NA | -3.32 | 1.37 | 0.32 | -0.38 | -1.74 | -1.60 | -1.81 | -1.27 | -0.43 | -1.39 | 2.45 | -1.79 | 3.10 | 0.05 | 0.09 | -0.22 | 0.23 | 0.68 | 2.58 | -1.65 |
| GoyetQ116-1 | -7.66 | -9.00 | 1.94 | -0.43 | 0.17 | 3.32 | NA | 4.20 | 3.31 | 3.28 | 1.97 | 1.33 | 2.48 | 2.24 | 3.37 | 1.89 | 5.50 | 1.55 | 6.77 | 3.26 | 3.69 | 3.51 | 3.58 | 4.11 | 6.24 | 1.58 |
| BachoKiro_BK_1653 | -11.46 | -12.45 | -2.24 | -4.18 | -4.13 | -1.37 | -4.20 | NA | -1.13 | -1.48 | -2.30 | -2.89 | -2.76 | -2.43 | -1.79 | -2.66 | 1.22 | -3.41 | 1.52 | -1.05 | -1.48 | -1.34 | -1.07 | -0.63 | 1.19 | -3.25 |
| SunghirIII | -11.47 | -13.38 | -1.35 | -3.22 | -3.14 | -0.32 | -3.31 | 1.13 | NA | -0.60 | -1.45 | -1.75 | -1.53 | -1.32 | -0.48 | -1.45 | 1.96 | -2.06 | 2.98 | 0.21 | -0.19 | -0.61 | 0.64 | 0.81 | 2.37 | -1.92 |
| Yana_old | -11.55 | -12.44 | -0.80 | -3.06 | -2.98 | 0.38 | -3.28 | 1.48 | 0.60 | NA | -1.28 | -1.23 | -1.33 | -1.07 | 0.18 | -0.91 | 3.09 | -1.55 | 3.54 | 0.79 | 0.44 | 0.15 | 0.89 | 1.20 | 2.98 | -1.33 |
| Vestonice16 | -9.56 | -10.70 | 0.23 | -2.04 | -1.57 | 1.74 | -1.97 | 2.30 | 1.45 | 1.28 | NA | -0.47 | -0.49 | 0.37 | 1.42 | -0.18 | 3.90 | -0.33 | 4.64 | 1.43 | 1.36 | 1.30 | 2.40 | 2.45 | 4.07 | 0.36 |
| Malta1 | -10.19 | -10.92 | 0.31 | -2.33 | -1.28 | 1.60 | -1.33 | 2.89 | 1.75 | 1.23 | 0.47 | NA | 0.26 | 0.43 | 1.30 | 0.54 | 4.32 | -0.23 | 4.75 | 1.82 | 1.64 | 1.61 | 2.40 | 2.66 | 4.34 | -0.12 |
| ElMiron | -9.32 | -10.28 | 0.36 | -1.72 | -1.40 | 1.81 | -2.48 | 2.76 | 1.53 | 1.33 | 0.49 | -0.26 | NA | 0.78 | 1.41 | 0.29 | 3.90 | -0.06 | 5.30 | 1.63 | 1.63 | 1.22 | 1.42 | 3.02 | 4.28 | -0.13 |
| Villabruna | -10.27 | -11.70 | -0.08 | -2.52 | -2.10 | 1.27 | -2.24 | 2.43 | 1.32 | 1.07 | -0.37 | -0.43 | -0.78 | NA | 1.01 | -0.62 | 3.68 | -0.81 | 4.46 | 1.07 | 1.15 | 1.15 | 1.58 | 2.44 | 4.18 | -0.59 |
| Bichon | -11.09 | -12.18 | -1.03 | -3.31 | -2.79 | 0.43 | -3.37 | 1.79 | 0.48 | -0.18 | -1.42 | -1.30 | -1.41 | -1.01 | NA | -1.16 | 2.78 | -1.82 | 3.84 | 0.55 | 0.33 | 0.18 | 0.57 | 1.17 | 2.95 | -1.49 |
| Clovis | -10.38 | -11.50 | -0.01 | -2.50 | -1.90 | 1.39 | -1.89 | 2.66 | 1.45 | 0.91 | 0.18 | -0.54 | -0.29 | 0.62 | 1.16 | NA | 4.04 | -0.80 | 4.61 | 1.66 | 1.42 | 1.27 | 2.18 | 2.32 | 4.06 | -0.16 |
| Satsurblia | -12.99 | -13.66 | -3.50 | -4.77 | -4.81 | -2.45 | -5.50 | -1.22 | -1.96 | -3.09 | -3.90 | -4.32 | -3.90 | -3.68 | -2.78 | -4.04 | NA | -5.11 | 0.64 | -2.32 | -2.52 | -2.48 | -1.71 | -1.77 | -0.27 | -4.36 |
| Kolyma_River | -9.96 | -11.18 | 0.55 | -2.04 | -1.43 | 1.79 | -1.55 | 3.41 | 2.06 | 1.55 | 0.33 | 0.23 | 0.06 | 0.81 | 1.82 | 0.80 | 5.11 | NA | 5.25 | 2.14 | 2.09 | 1.86 | 2.56 | 2.79 | 4.65 | 0.31 |
| Kotias | -14.34 | -15.11 | -3.99 | -5.39 | -6.14 | -3.10 | -6.77 | -1.52 | -2.98 | -3.54 | -4.64 | -4.75 | -5.30 | -4.46 | -3.84 | -4.61 | -0.64 | -5.25 | NA | -3.37 | -3.54 | -3.70 | -3.12 | -2.76 | -0.85 | -5.09 |
| Karelia | -11.64 | -11.86 | -1.55 | -4.03 | -3.35 | -0.05 | -3.26 | 1.05 | -0.21 | -0.79 | -1.43 | -1.82 | -1.63 | -1.07 | -0.55 | -1.66 | 2.32 | -2.14 | 3.37 | NA | -0.32 | -0.11 | 0.31 | 1.04 | 2.35 | -2.49 |
| Loschbour | -11.06 | -12.82 | -1.27 | -2.97 | -3.24 | -0.09 | -3.69 | 1.48 | 0.19 | -0.44 | -1.36 | -1.64 | -1.63 | -1.15 | -0.33 | -1.42 | 2.52 | -2.09 | 3.54 | 0.32 | NA | -0.38 | 0.54 | 1.10 | 2.72 | -1.79 |
| LaBrana1 | -11.46 | -12.54 | -0.95 | -2.94 | -3.24 | 0.22 | -3.51 | 1.34 | 0.61 | -0.15 | -1.30 | -1.61 | -1.22 | -1.15 | -0.18 | -1.27 | 2.48 | -1.86 | 3.70 | 0.11 | 0.38 | NA | 0.83 | 1.03 | 3.02 | -1.62 |
| Hungarian.KO1 | -11.18 | -11.86 | -1.31 | -3.03 | -3.70 | -0.23 | -3.58 | 1.07 | -0.64 | -0.89 | -2.40 | -2.40 | -1.42 | -1.58 | -0.57 | -2.18 | 1.71 | -2.56 | 3.12 | -0.31 | -0.54 | -0.83 | NA | 0.10 | 1.91 | -2.09 |
| Motala12 | -12.21 | -13.29 | -2.08 | -3.29 | -4.13 | -0.68 | -4.11 | 0.63 | -0.81 | -1.20 | -2.45 | -2.66 | -3.02 | -2.44 | -1.17 | -2.32 | 1.77 | -2.79 | 2.76 | -1.04 | -1.10 | -1.03 | -0.10 | NA | 1.98 | -2.76 |
| LBK | -13.58 | -14.70 | -3.42 | -5.31 | -5.80 | -2.58 | -6.24 | -1.19 | -2.37 | -2.98 | -4.07 | -4.34 | -4.28 | -4.18 | -2.95 | -4.06 | 0.27 | -4.65 | 0.85 | -2.35 | -2.72 | -3.02 | -1.91 | -1.98 | NA | -4.56 |
| Saqqaq | -10.27 | -11.16 | 0.28 | -2.36 | -1.86 | 1.65 | -1.58 | 3.25 | 1.92 | 1.33 | -0.36 | 0.12 | 0.13 | 0.59 | 1.49 | 0.16 | 4.36 | -0.31 | 5.09 | 2.49 | 1.79 | 1.62 | 2.09 | 2.76 | 4.56 | NA |

**Table S5.15 Z-scores of the statistics *D(X,Y; Bacho Kiro BK1653, Mbuti)*.** Standard errors (SE) were computed using a Weighted Block Jackknife[3,12] across all autosomes of the "2200k" Panel and using a block size of 5Mb. Blue: Z-score ≤ -3; Yellow: Z-score ≥ 3.

| X/Y | BachoKiro_F6_620 | BachoKiro_BB7_240 | BachoKiro_CC7_335 | UstIshim | Oase1 | Tianyuan | Kostenki14 | GoyetQ116-1 | SunghirIII | Yana_old | Vestonice16 | Malta1 | ElMiron | Villabruna | Bichon | Clovis | Satsurblia | Kolyma_River | Kotias | Karelia | Loschbour | LaBrana1 | Hungarian.KO1 | Motala12 | LBK | Saqqaq |
|---|---|---|---|---|---|---|---|---|---|---|---|---|---|---|---|---|---|---|---|---|---|---|---|---|---|---|
| BachoKiro_F6_620 | NA | -2.36 | 0.15 | -1.23 | 2.00 | -1.68 | -12.26 | -15.82 | -12.37 | -9.21 | -17.39 | -8.32 | -14.87 | -15.03 | -13.67 | -3.68 | -3.16 | -3.99 | -4.66 | -10.18 | -14.08 | -14.75 | -12.29 | -12.05 | -8.89 | -3.37 |
| BachoKiro_BB7_240 | 2.36 | NA | 2.01 | 0.72 | 1.86 | 0.97 | -9.78 | -13.19 | -10.04 | -6.63 | -14.52 | -6.40 | -12.32 | -12.20 | -11.19 | -1.04 | -1.21 | -1.51 | -3.00 | -7.84 | -11.42 | -11.08 | -9.41 | -8.87 | -6.35 | -1.13 |
| BachoKiro_CC7_335 | -0.15 | -2.01 | NA | -1.60 | 1.00 | -1.30 | -11.46 | -15.02 | -12.18 | -8.30 | -16.46 | -8.13 | -14.52 | -13.65 | -13.48 | -3.19 | -3.01 | -3.86 | -4.32 | -9.63 | -14.50 | -13.64 | -11.63 | -11.26 | -8.41 | -3.55 |
| UstIshim | 1.23 | -0.72 | 1.60 | NA | 2.38 | -0.30 | -12.03 | -14.52 | -12.01 | -8.44 | -17.32 | -7.65 | -14.17 | -14.84 | -14.06 | -2.62 | -2.29 | -2.93 | -3.66 | -9.86 | -14.46 | -14.66 | -12.01 | -11.75 | -8.21 | -2.32 |
| Oase1 | -2.00 | -1.86 | -1.00 | -2.38 | NA | -2.40 | -11.87 | -14.89 | -12.26 | -9.24 | -15.86 | -8.87 | -14.31 | -14.42 | -13.94 | -4.14 | -3.55 | -5.00 | -5.72 | -9.84 | -14.63 | -13.28 | -11.07 | -10.95 | -8.97 | -3.90 |
| Tianyuan | 1.68 | -0.97 | 1.30 | 0.30 | 2.40 | NA | -10.79 | -15.38 | -12.06 | -8.61 | -16.34 | -8.04 | -14.66 | -14.41 | -13.55 | -1.96 | -1.62 | -2.49 | -2.87 | -8.97 | -13.24 | -13.56 | -11.80 | -11.71 | -7.41 | -1.83 |
| Kostenki14 | 12.26 | 9.78 | 11.46 | 12.03 | 11.87 | 10.79 | NA | -3.47 | -0.10 | 4.06 | -5.57 | 4.23 | -3.14 | -2.65 | -1.41 | 10.15 | 9.58 | 9.92 | 8.90 | 2.83 | -1.49 | -1.74 | 0.23 | 0.99 | 4.66 | 9.88 |
| GoyetQ116-1 | 15.82 | 13.19 | 15.02 | 14.52 | 14.89 | 15.38 | 3.47 | NA | 3.22 | 7.69 | -1.21 | 7.17 | 0.98 | 1.84 | 2.24 | 13.86 | 13.03 | 14.17 | 12.16 | 5.43 | 2.03 | 2.38 | 4.17 | 4.41 | 8.62 | 14.48 |
| SunghirIII | 12.37 | 10.04 | 12.18 | 12.01 | 12.26 | 12.06 | 0.10 | -3.22 | NA | 4.07 | -5.27 | 4.40 | -2.89 | -2.26 | -1.21 | 10.46 | 9.96 | 9.79 | 8.79 | 2.83 | -1.39 | -1.50 | 0.59 | 1.10 | 4.82 | 10.72 |
| Yana_old | 9.21 | 6.63 | 8.30 | 8.44 | 9.24 | 8.61 | -4.06 | -7.69 | -4.07 | NA | -9.66 | 0.45 | -7.25 | -7.21 | -5.46 | 6.90 | 6.75 | 6.66 | 5.16 | -1.20 | -5.73 | -5.96 | -3.26 | -3.47 | 0.93 | 7.00 |
| Vestonice16 | 17.39 | 14.52 | 16.46 | 17.32 | 15.86 | 16.34 | 5.57 | 1.21 | 5.27 | 9.66 | NA | 9.41 | 2.03 | 3.24 | 3.86 | 15.60 | 15.27 | 16.07 | 14.91 | 8.21 | 3.94 | 3.91 | 5.83 | 6.63 | 10.60 | 16.19 |
| Malta1 | 8.32 | 6.40 | 8.13 | 7.65 | 8.87 | 8.04 | -4.23 | -7.17 | -4.40 | -0.45 | -9.41 | NA | -5.94 | -6.28 | -5.85 | 6.63 | 5.29 | 5.92 | 4.15 | -1.59 | -6.05 | -6.43 | -3.93 | -3.66 | 0.32 | 6.69 |
| ElMiron | 14.87 | 12.32 | 14.52 | 14.17 | 14.31 | 14.66 | 3.14 | -0.98 | 2.89 | 7.25 | -2.03 | 5.94 | NA | 1.55 | 2.22 | 14.08 | 12.91 | 13.07 | 11.96 | 6.14 | 1.92 | 2.21 | 3.87 | 4.10 | 8.45 | 13.95 |
| Villabruna | 15.03 | 12.20 | 13.65 | 14.84 | 14.42 | 14.41 | 2.65 | -1.84 | 2.26 | 7.21 | -3.24 | 6.28 | -1.55 | NA | 1.04 | 13.31 | 13.01 | 13.07 | 12.41 | 5.36 | 0.81 | 0.91 | 3.41 | 3.55 | 8.27 | 14.23 |
| Bichon | 13.67 | 11.19 | 13.48 | 14.06 | 13.94 | 13.55 | 1.41 | -2.24 | 1.21 | 5.46 | -3.86 | 5.85 | -2.22 | -1.04 | NA | 12.70 | 12.66 | 12.27 | 11.27 | 4.48 | -0.20 | -0.25 | 2.10 | 2.80 | 6.90 | 13.14 |
| Clovis | 3.68 | 1.04 | 3.19 | 2.62 | 4.14 | 1.96 | -10.15 | -13.86 | -10.46 | -6.90 | -15.60 | -6.63 | -14.08 | -13.31 | -12.70 | NA | -0.07 | -0.40 | -1.53 | -8.13 | -13.03 | -12.93 | -10.30 | -10.21 | -6.29 | 0.30 |
| Satsurblia | 3.16 | 1.21 | 3.01 | 2.29 | 3.55 | 1.62 | -9.58 | -13.03 | -9.96 | -6.75 | -15.27 | -5.29 | -12.91 | -13.01 | -12.66 | 0.07 | NA | -0.78 | -2.05 | -8.08 | -12.37 | -12.81 | -9.44 | -9.89 | -6.79 | 0.03 |
| Kolyma_River | 3.99 | 1.51 | 3.86 | 2.93 | 5.00 | 2.49 | -9.92 | -14.17 | -9.79 | -6.66 | -16.07 | -5.92 | -13.07 | -13.07 | -12.27 | 0.40 | 0.78 | NA | -1.10 | -7.67 | -12.47 | -13.05 | -9.86 | -9.93 | -5.97 | 0.50 |
| Kotias | 4.66 | 3.00 | 4.32 | 3.66 | 5.72 | 2.87 | -8.90 | -12.16 | -8.79 | -5.16 | -14.91 | -4.15 | -11.96 | -12.41 | -11.27 | 1.53 | 2.05 | 1.10 | NA | -6.85 | -11.36 | -11.73 | -9.02 | -8.78 | -4.76 | 1.54 |
| Karelia | 10.18 | 7.84 | 9.63 | 9.86 | 9.84 | 8.97 | -2.83 | -5.43 | -2.83 | 1.20 | -8.21 | 1.59 | -6.14 | -5.36 | -4.48 | 8.13 | 8.08 | 7.67 | 6.85 | NA | -4.89 | -5.15 | -3.02 | -2.13 | 2.17 | 8.29 |
| Loschbour | 14.08 | 11.42 | 14.50 | 14.46 | 14.63 | 13.24 | 1.49 | -2.03 | 1.39 | 5.73 | -3.94 | 6.05 | -1.92 | -0.81 | 0.20 | 13.03 | 12.37 | 12.47 | 11.36 | 4.89 | NA | -0.20 | 2.32 | 3.07 | 7.49 | 12.87 |
| LaBrana1 | 14.75 | 11.08 | 13.64 | 14.66 | 13.28 | 13.56 | 1.74 | -2.38 | 1.50 | 5.96 | -3.91 | 6.43 | -2.21 | -0.91 | 0.25 | 12.93 | 12.81 | 13.05 | 11.73 | 5.15 | 0.20 | NA | 2.59 | 3.06 | 7.54 | 13.26 |
| Hungarian.KO1 | 12.29 | 9.41 | 11.63 | 12.01 | 11.07 | 11.80 | -0.23 | -4.17 | -0.59 | 3.26 | -5.83 | 3.93 | -3.87 | -3.41 | -2.10 | 10.30 | 9.44 | 9.86 | 9.02 | 3.02 | -2.32 | -2.59 | NA | 0.75 | 4.82 | 10.58 |
| Motala12 | 12.05 | 8.87 | 11.26 | 11.75 | 10.95 | 11.71 | -0.99 | -4.41 | -1.10 | 3.47 | -6.63 | 3.66 | -4.10 | -3.55 | -2.80 | 10.21 | 9.89 | 9.93 | 8.78 | 2.13 | -3.07 | -3.06 | -0.75 | NA | 4.66 | 10.35 |
| LBK | 8.89 | 6.35 | 8.41 | 8.21 | 8.97 | 7.41 | -4.66 | -8.62 | -4.82 | -0.93 | -10.60 | -0.32 | -8.45 | -8.27 | -6.90 | 6.29 | 6.79 | 5.97 | 4.76 | -2.17 | -7.49 | -7.54 | -4.82 | -4.66 | NA | 5.95 |
| Saqqaq | 3.37 | 1.13 | 3.55 | 2.32 | 3.90 | 1.83 | -9.88 | -14.48 | -10.72 | -7.00 | -16.19 | -6.69 | -13.95 | -14.23 | -13.14 | -0.30 | -0.03 | -0.50 | -1.54 | -8.29 | -12.87 | -13.26 | -10.58 | -10.35 | -5.95 | NA |

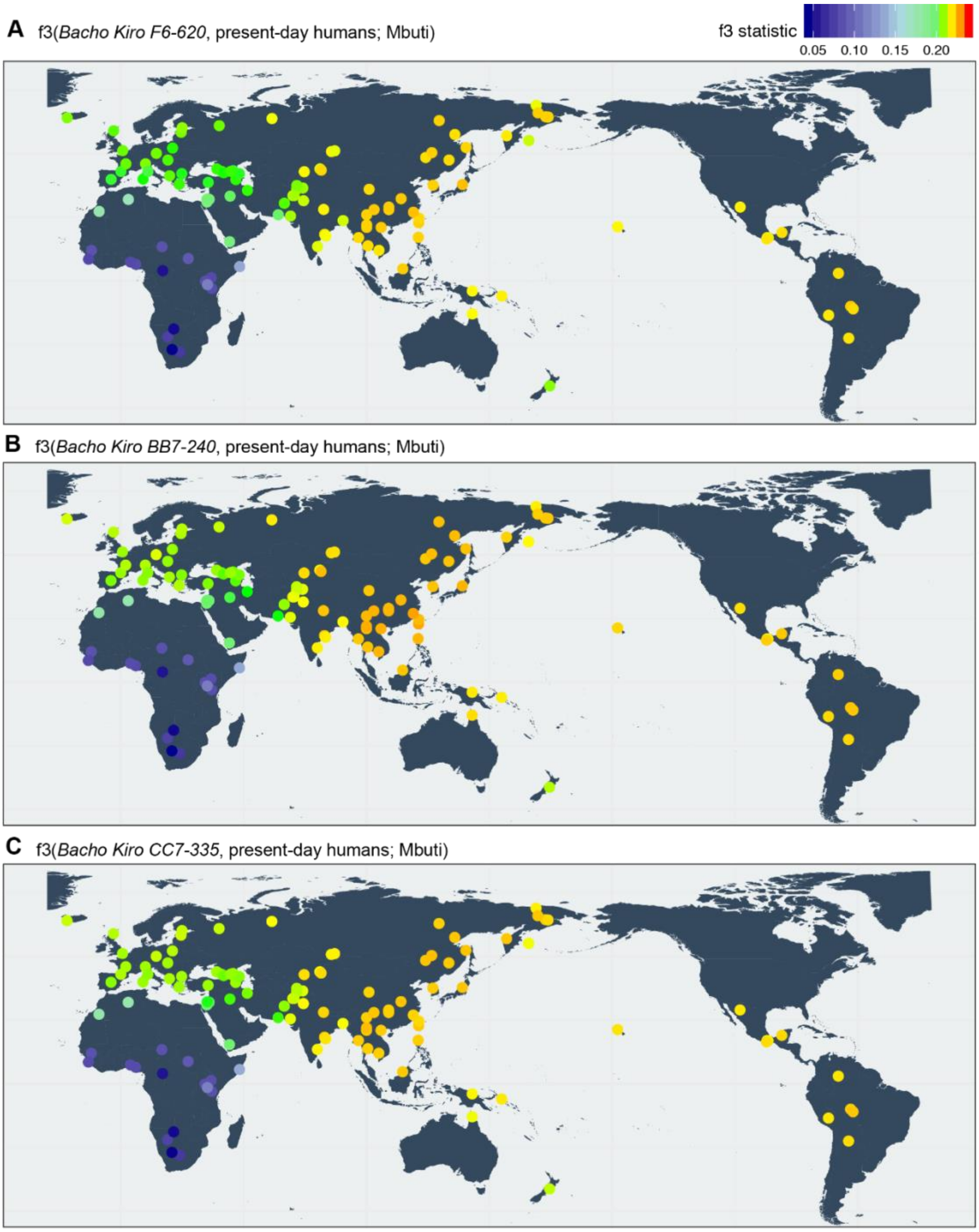


**Figure S5.1 Heatmap of the shared genetic drift between the IUP Bacho Kiro Cave individuals and present-day human populations calculated as *f3(IUP Bacho Kiro individual, present-day humans; Mbuti)*. A)** *Bacho Kiro F6-620*, **B)** *Bacho Kiro BB7-240*, **C)** *Bacho Kiro CC7-335*. Present-day modern human genomes from SGDP[10] were used for calculating these statistics. Plotted *f3* values were calculated using ADMIXTOOLS[3] as implemented in *admixr*[9], and which are reported in detail in Tables S5.2-S5.4 along with the number of SNPs used for these calculations. A higher *f3*-value is indicated with warmer colour and corresponds to the higher shared genetic drift between an IUP Bacho Kiro Cave individual

and a given present-day human population. Standard errors (SE) were computed using a Weighted Block Jackknife[3,12] across all autosomes of the “2200k” Panel and using a block size of 5Mb. Three Mbuti individuals from SGDP[10] were used as outgroup. Coordinates for present-day humans were obtained from *Mallick et al.*[10]. The heatmap scale is kept consistent with the Fig. 2A, Extended Data Fig. 3B and C, and Fig. S5.2.

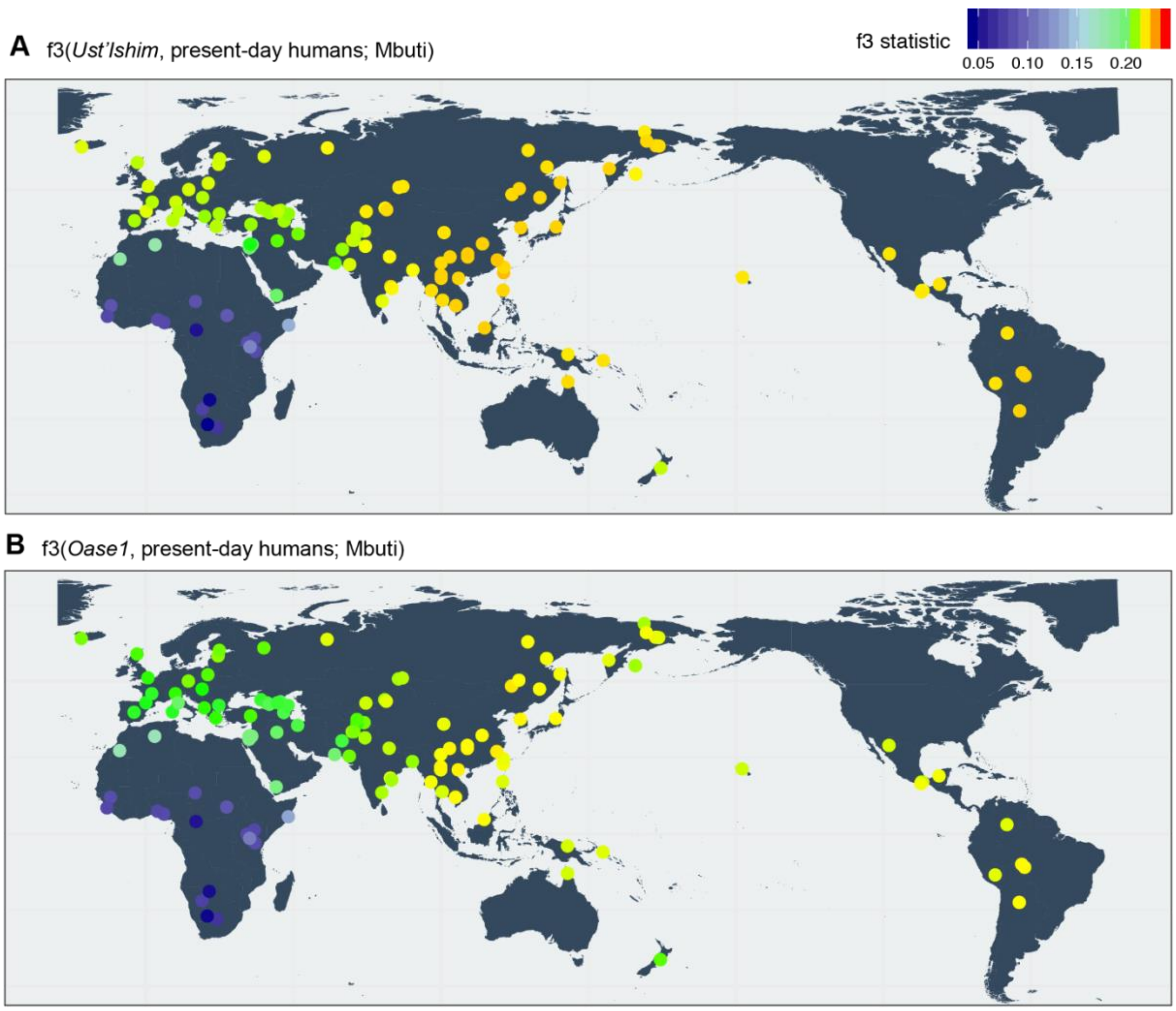


**Figure S5.2 Heatmap of the shared genetic drift between the *Ust'Ishim* or *Oase1* and present-day human populations calculated as *f3(Ust'Ishim/Oase1, present-day humans; Mbuti)*.** Present-day modern human genomes from SGDP[10] were used for calculating these statistics. Plotted *f3* values were calculated using ADMIXTOOLS[3] as implemented in *admixr*[9]. A higher *f3*-value is indicated with warmer colour and corresponds to higher shared genetic drift between **A)** *Ust'Ishim* or **B)** *Oase1* and a given present-day human population. Standard errors (SE) were computed using a Weighted Block Jackknife[3,12] across all autosomes of the "2200k" Panel (nsnps (*Ust'Ishim*) = 1,951,462; nsnps (*Oase 1*) = 402,526), and using a block size of 5Mb. Three Mbuti individuals from SGDP[10] were used as outgroup. Coordinates for present-day humans were obtained from *Mallick et al.*[10]. The heatmap scale is kept consistent with the Fig. 2A, Extended Data Fig. 3B and C, and Fig. S5.1.

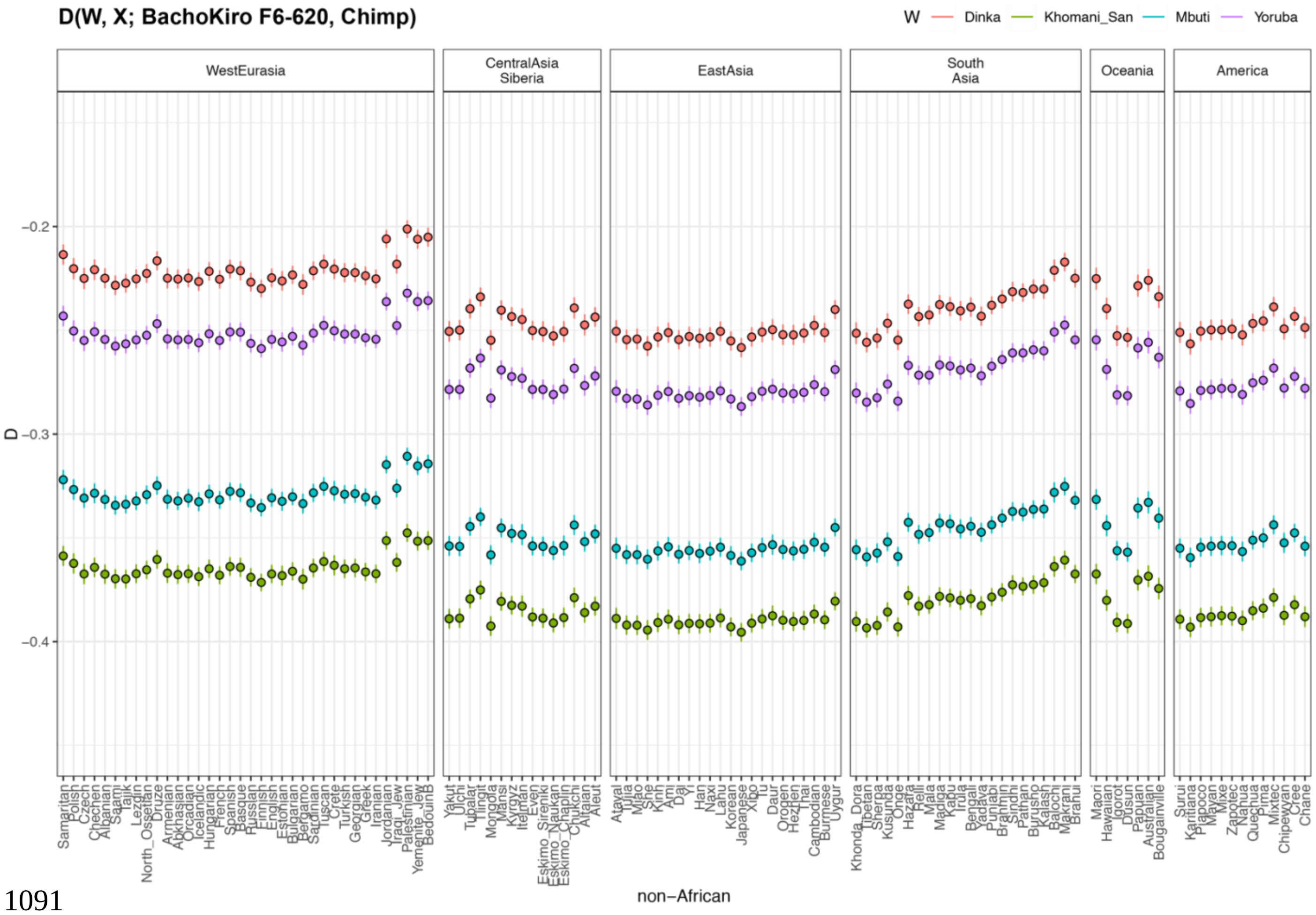


**Figure S5.3 D-statistics of the form *D(W, X; Bacho Kiro F6-620, Chimpanzee)* where *W* is a present-day African population and *X* is a present-day non-African population from SGDP[10].** *Bacho Kiro F6-620* is significantly closer to present-day non-Africans than present-day Africans. *D* values plotted on the *y*-axis were calculated using ADMIXTOOLS[3] as implemented in *admixr*[9]. Filled-in circles indicate a significant Z-score or $|Z| \geq 3$, and open circles indicate a non-significant Z-score or $|Z| < 3$. Whiskers on each side of the plotted *D* values correspond to one standard error calculated using a Weighted Block Jackknife[3,12] across all autosomes on the "2200k" Panel (nsnps (*Bacho Kiro F6-620*) = 1,779,883) and a block size of 5 Mb.

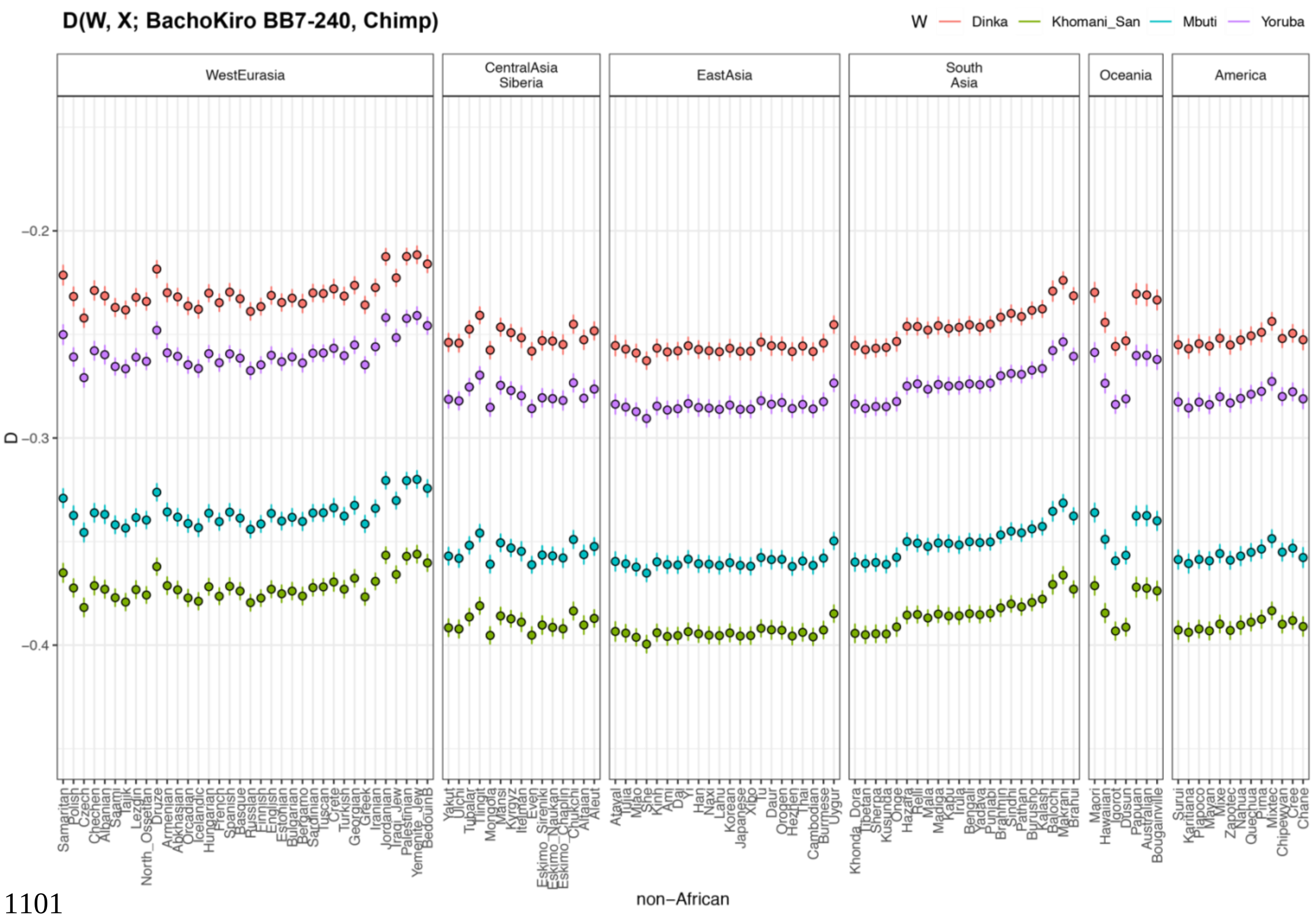


**Figure S5.4 D-statistics of the form *D(W, X; Bacho Kiro BB7-240, Chimpanzee)* where *W* is a present-day African population and *X* is a present-day non-African population from SGDP[10].** *Bacho Kiro BB7-240* is significantly closer to present-day non-Africans than present-day Africans. *D* values plotted on the *y*-axis were calculated using ADMIXTOOLS[3] as implemented in *admixr*[9]. Filled-in circles indicate a significant Z-score or $|Z| \geq 3$, and open circles indicate a non-significant Z-score or $|Z| < 3$. Whiskers on each side of the plotted *D* values correspond to one standard error calculated using a Weighted Block Jackknife[3,12] across all autosomes on the “2200k” Panel (nsnps (*Bacho Kiro BB7-240*) = 787,706) and a block size of 5 Mb.

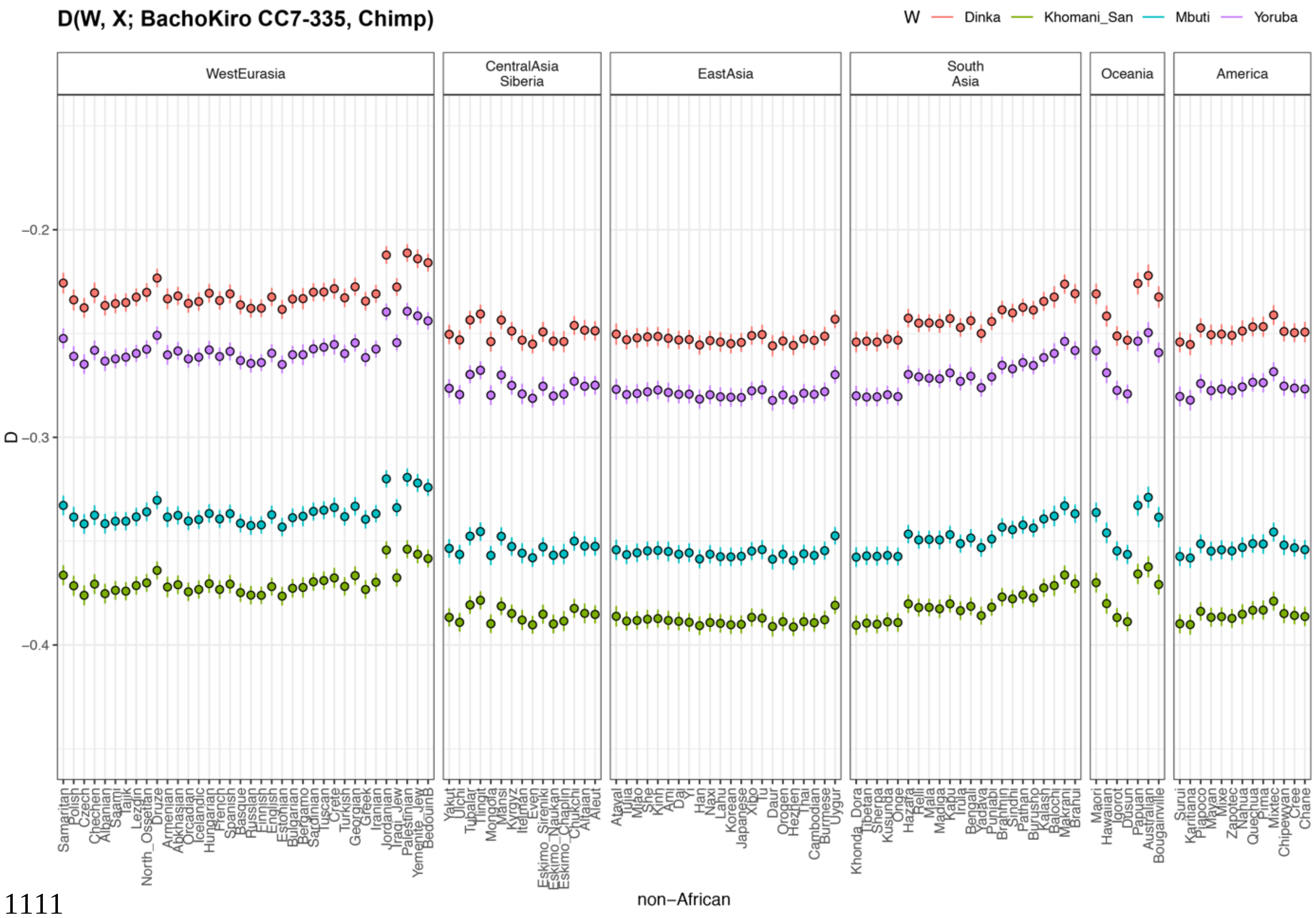


**Figure S5.5 D-statistics of the form *D(W, X; Bacho Kiro CC7-335, Chimpanzee)* where *W* is a present-day African population and *X* is a present-day non-African population from SGDP[10].** *Bacho Kiro CC7-335* is significantly closer to present-day non-Africans than present-day Africans. *D* values denoted as circles and plotted on the *y*-axis were calculated using ADMIXTOOLS[3] as implemented in *admixr*[9]. Filled-in circles indicate a significant Z-score or $|Z| \geq 3$, and open circles indicate a non-significant Z-score or $|Z| < 3$. Whiskers on each side of the plotted *D* values correspond to one standard error calculated using a Weighted Block Jackknife[3,12] across all autosomes on the “2200k” Panel (nsnps (*Bacho Kiro CC7-335*) = 723,129) and a block size of 5 Mb.

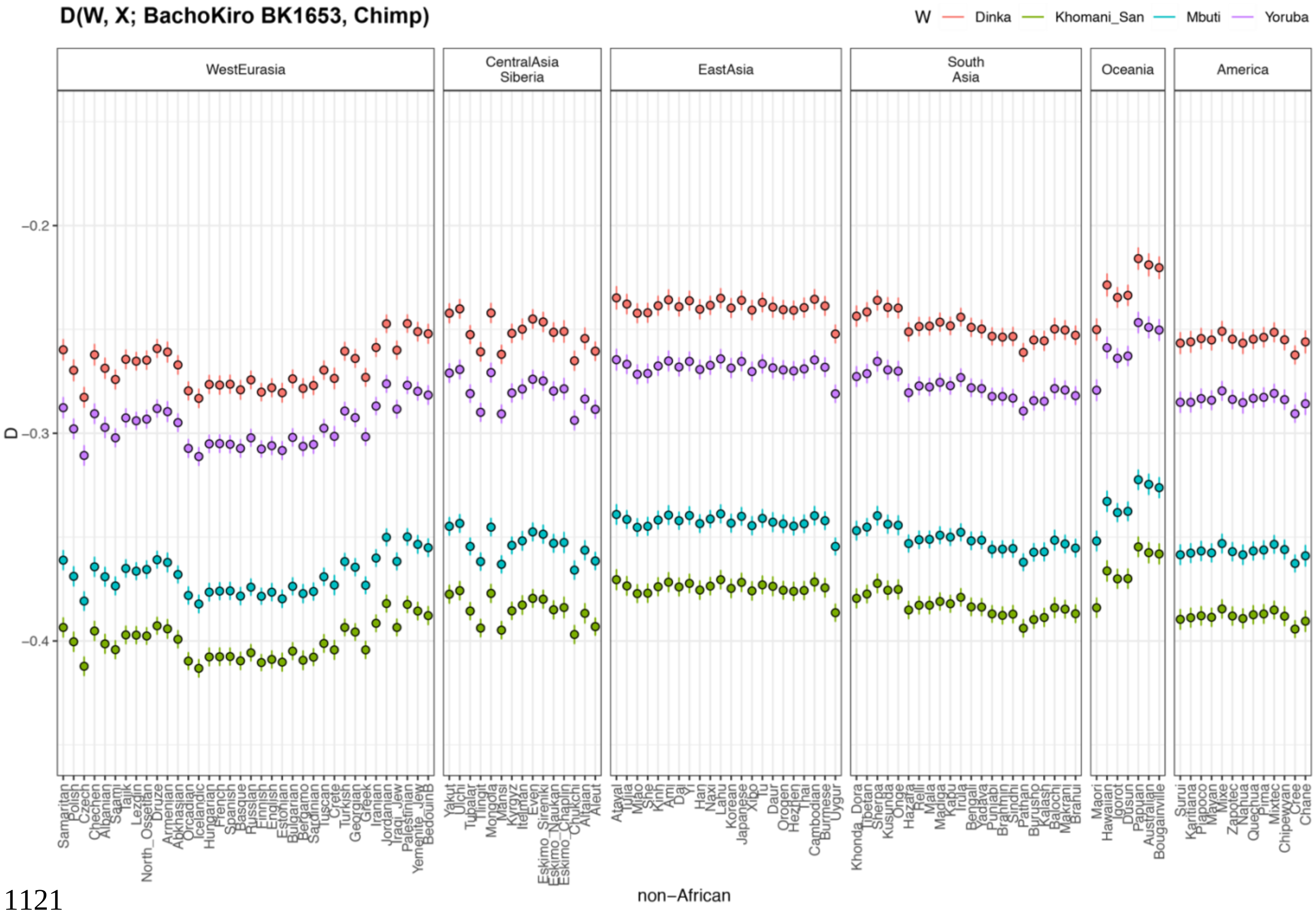


**Figure S5.6 D-statistics of the form *D(W, X; Bacho Kiro BK1653, Chimpanzee)* where *W* is a present-day African population and *X* is a present-day non-African population from SGDP[10].** *Bacho Kiro BK1653* is significantly closer to present-day non-Africans than present-day Africans. *D* values denoted as circles and plotted on the *y*-axis were calculated using ADMIXTOOLS[3] as implemented in *admixr*[9]. Filled-in circles indicate a significant Z-score or $|Z| \geq 3$, and open circles indicate a non-significant Z-score or $|Z| < 3$. Whiskers on each side of the plotted *D* values correspond to one standard error calculated using a Weighted Block Jackknife[3,12] across all autosomes on the "2200k" Panel (nsnps (*Bacho Kiro BK1653*) = 825,379) and a block size of 5 Mb.

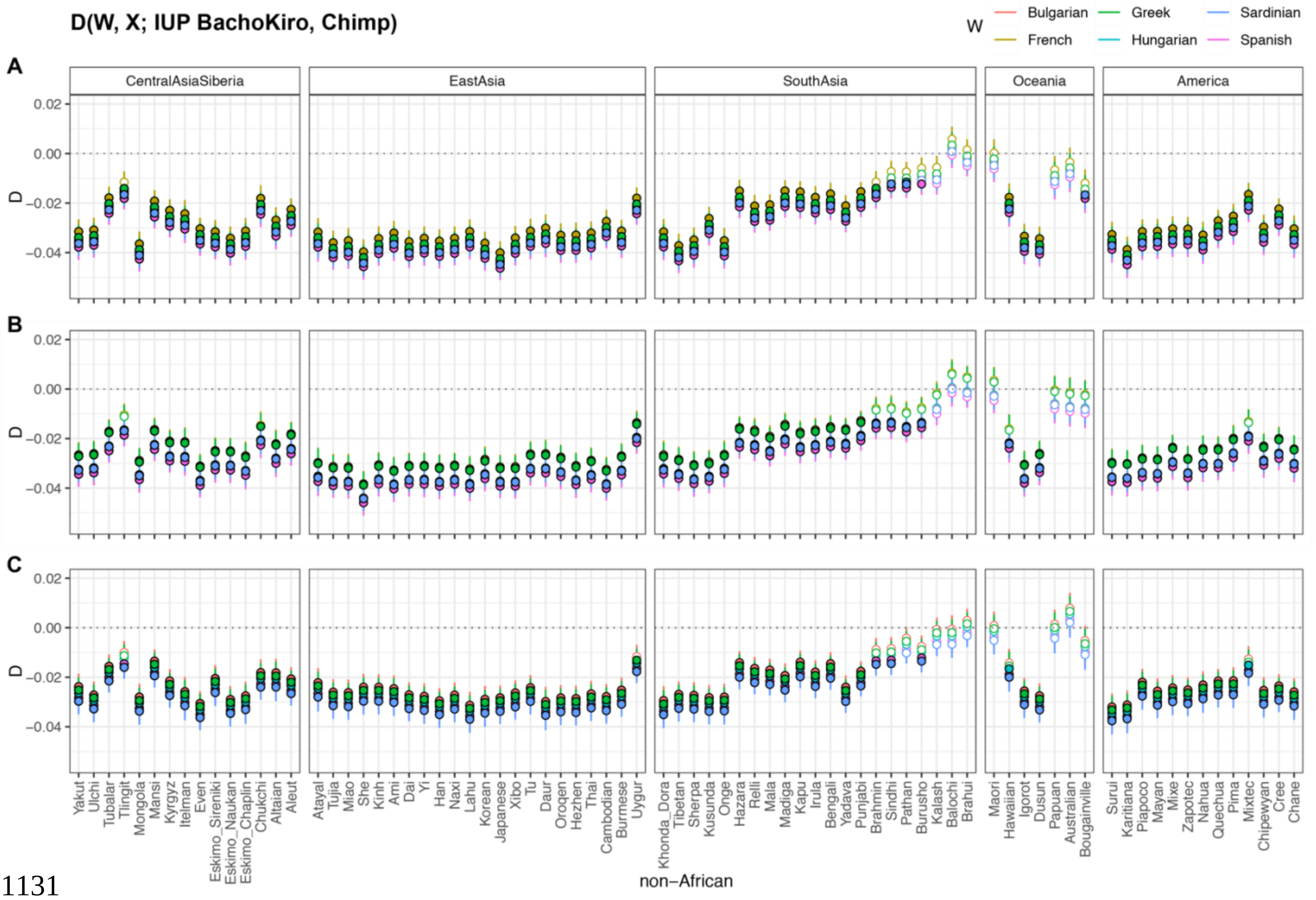


**Figure S5.7 D-statistics of the form *D(W, X; IUP Bacho Kiro individual, Chimpanzee)* where *W* is one of the six present-day West Eurasian populations and *X* is a present-day non-African population from Central Asia and Siberia, East Asia, South Asia, Oceania or Americas. A)** *Bacho Kiro F6-620* (nsnps = 1,779,883), **B)** *Bacho Kiro BB7-240* (nsnps = 787,706) and **C)** *Bacho Kiro CC7-335* (nsnps = 723,129). *D* values denoted as circles and plotted on the *y*-axes were calculated using ADMIXTOOLS[3] as implemented in *admixr*[9]. Present-day human genomes from SGDP[10] were used in these statistics, and a genome of *panTro2* as an outgroup. Filled-in circles indicate a significant Z-score or |Z| ≥ 3, and open circles indicate a non-significant Z-score or |Z| < 3. Whiskers on each side of the plotted *D* values correspond to one standard error calculated using a Weighted Block Jackknife[3,12] across all autosomes on the "2200k" Panel and a block size of 5 Mb.

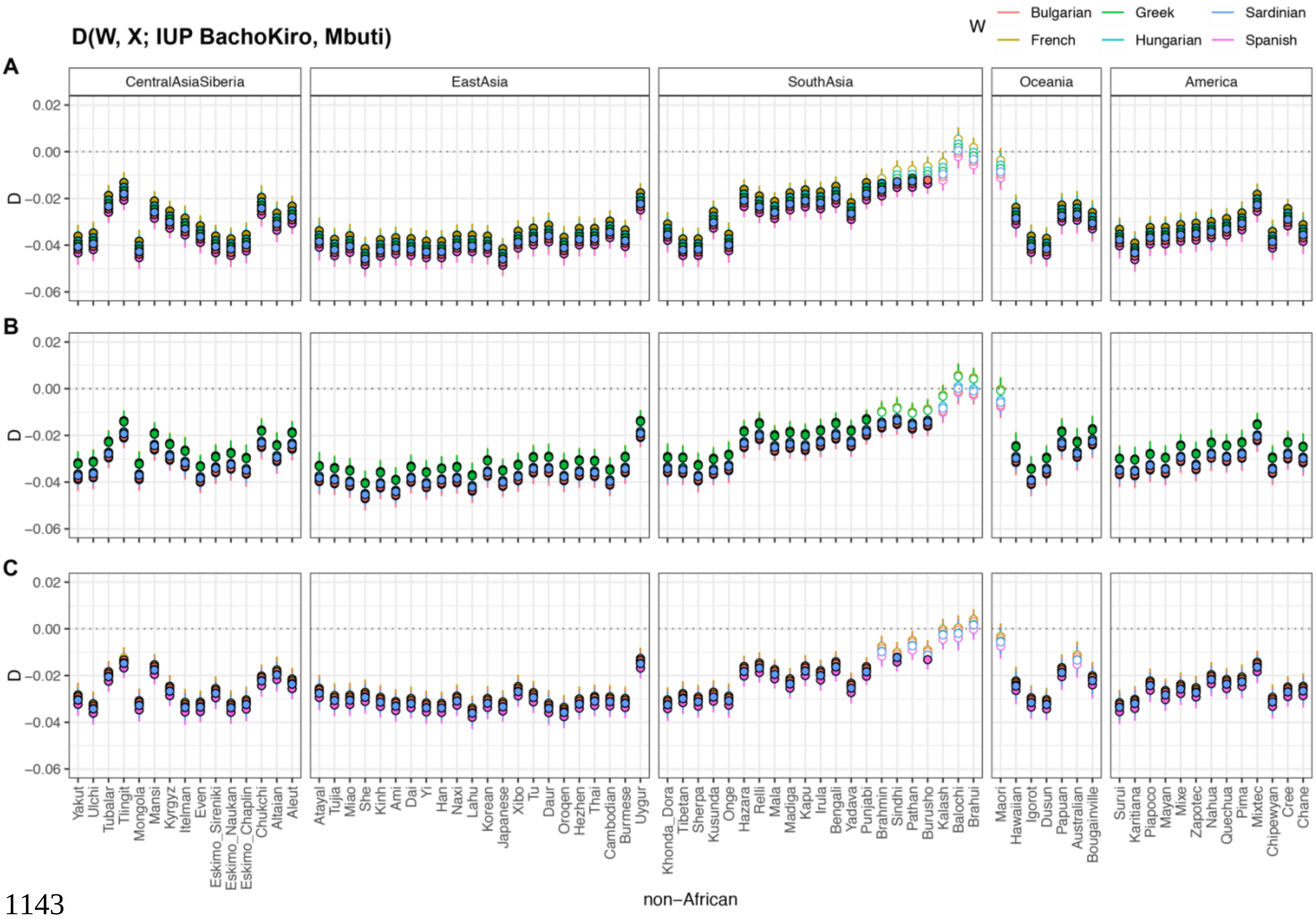


**Figure S5.8 D-statistics of the form *D(W, X; Bacho Kiro BK1653, Mbuti)* where *W* is one of the six present-day West Eurasian populations and *X* is a present-day non-African population from Central Asia and Siberia, East Asia, South Asia, Oceania or Americas. A)** *Bacho Kiro F6-620* (nsnps = 1,779,883), **B)** *Bacho Kiro BB7-240* (nsnps = 787,706) and **C)** *Bacho Kiro CC7-335* (nsnps = 723,129). *D* values denoted as circles and plotted on the *y*-axes were calculated using ADMIXTOOLS[3] as implemented in *admixr*[9]. Present-day human genomes from SGDP[10] were used in these statistics, and three Mbuti individuals from the same panel were used as outgroup. Filled-in circles indicate a significant Z-score or |Z| ≥ 3, and open circles indicate a non-significant Z-score or |Z| < 3. Whiskers on each side of the plotted *D* values correspond to one standard error calculated using a Weighted Block Jackknife[3,12] across all autosomes on the “2200k” Panel and a block size of 5 Mb.

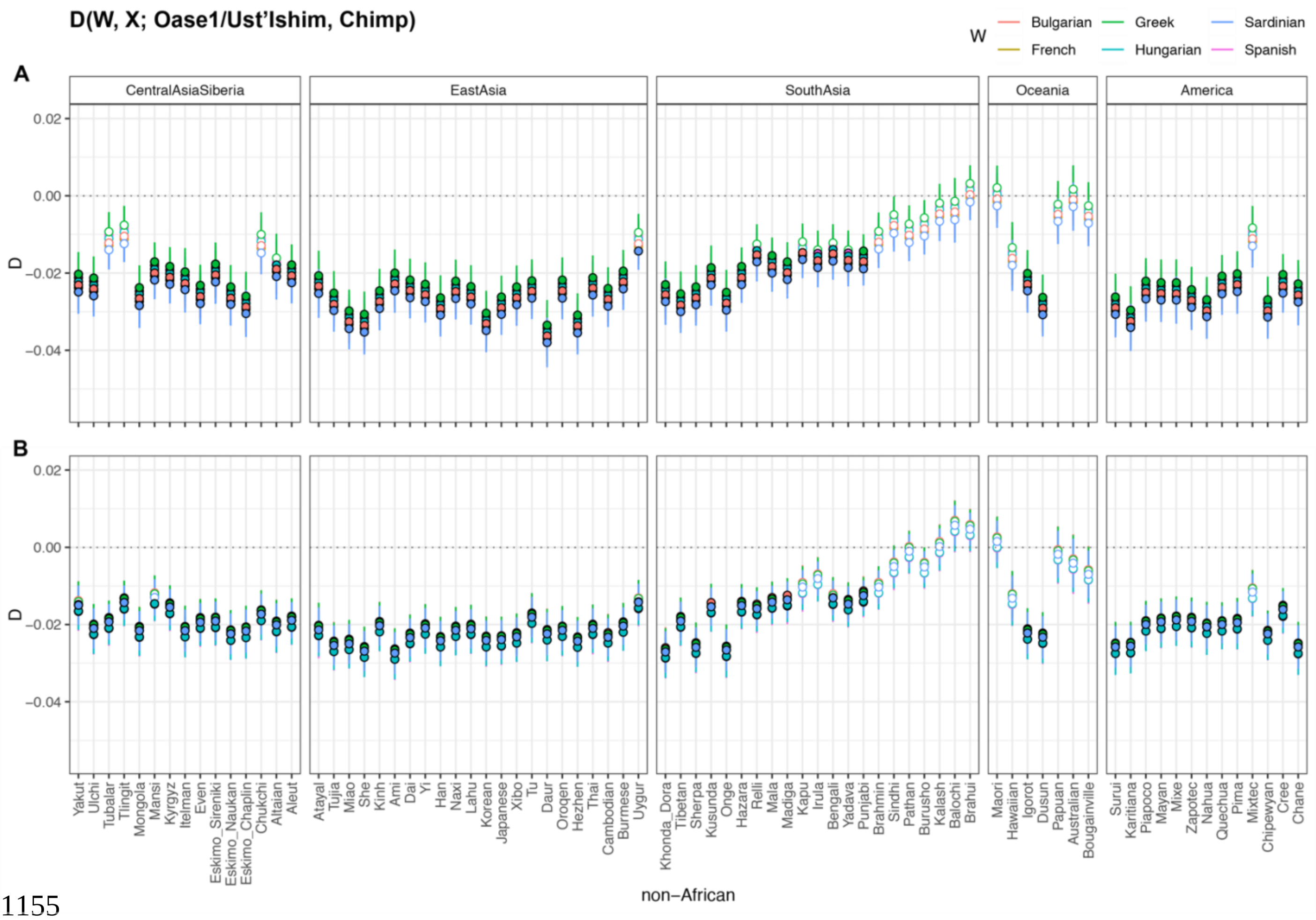


**Figure S5.9 D-statistics of the form *D(W, X; Ust'Ishim/Oase1, Chimpanzee)* where *W* is one of the six present-day West Eurasian populations and *X* is a present-day non-African population from Central Asia and Siberia, East Asia, South Asia, Oceania or Americas. A)** *Oase1* (nsnps = 402,526) and **B)** *Ust'Ishim* (nsnps = 1,951,462). *D* values denoted as circles and plotted on the *y*-axes were calculated using ADMIXTOOLS[3] as implemented in *admixr*[9]. Present-day human genomes from SGDP[10] were used in these statistics, and a genome of *panTro2* as an outgroup. Filled-in circles indicate a significant Z-score or |Z| ≥ 3, and open circles indicate a non-significant Z-score or |Z| < 3. Whiskers on each side of the plotted *D* values correspond to one standard error calculated using a Weighted Block Jackknife[3,12] across all autosomes on the "2200k" Panel and a block size of 5 Mb.

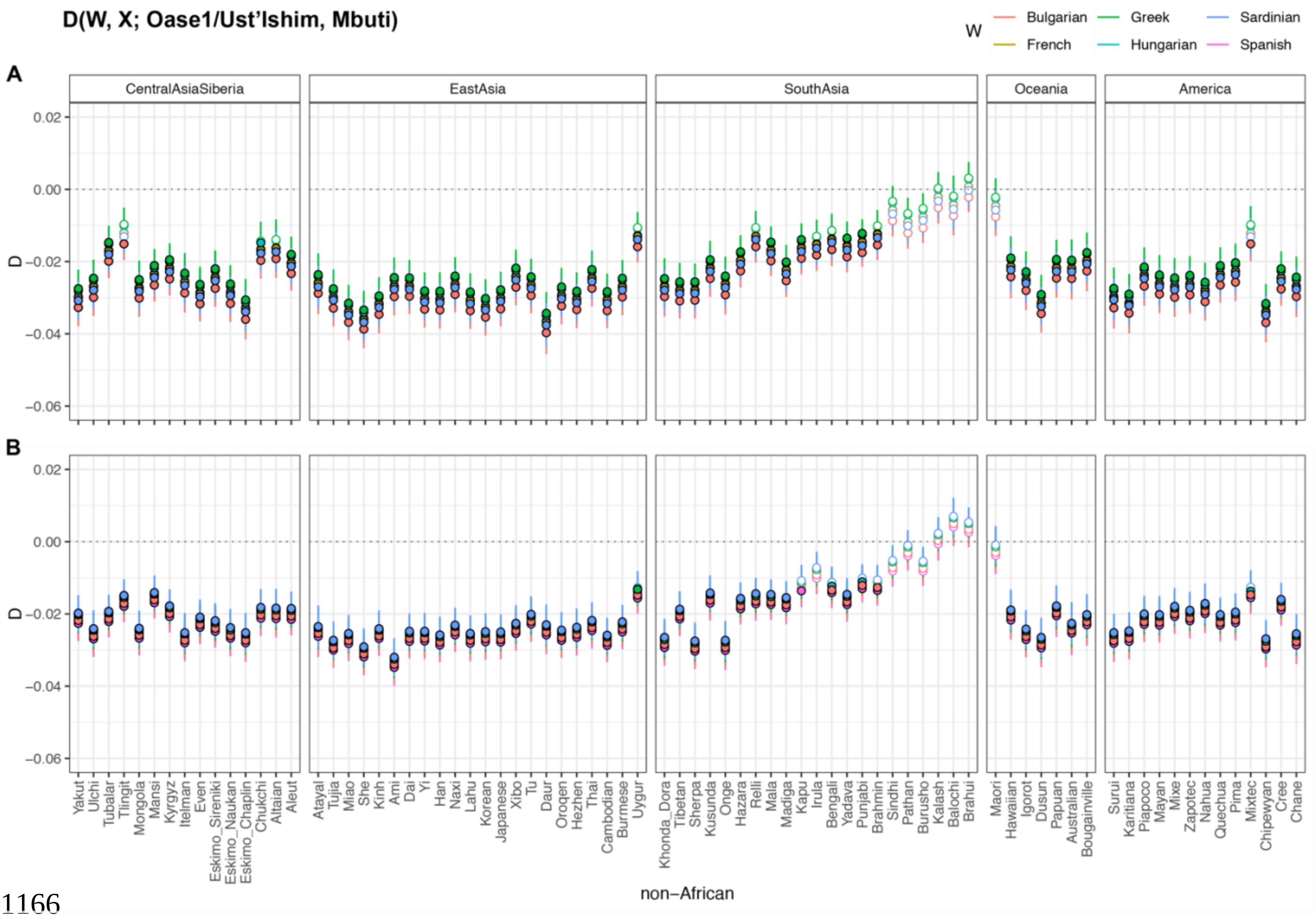


**Figure S5.10 D-statistics of the form *D(W, X; Ust'Ishim/Oase1, Mbuti)* where *W* is one of the six present-day West Eurasian populations and *X* is a present-day non-African population from Central Asia and Siberia, East Asia, South Asia, Oceania or Americas.** **A)** *Oase1* (nsnps = 402,526) and **B)** *Ust'Ishim* (nsnps = 1,951,462). *D* values denoted as circles and plotted on the *y*-axes were calculated using ADMIXTOOLS[3] as implemented in *admixr*[9]. Present-day human genomes from SGDP[10] were used in these statistics, and three Mbuti individuals from the same panel were used as outgroup. Filled-in circles indicate a significant Z-score or |Z| ≥ 3, and open circles indicate a non-significant Z-score or |Z| < 3. Whiskers on each side of the plotted *D* values correspond to one standard error calculated using a Weighted Block Jackknife[3,12] across all autosomes on the "2200k" Panel and a block size of 5 Mb.

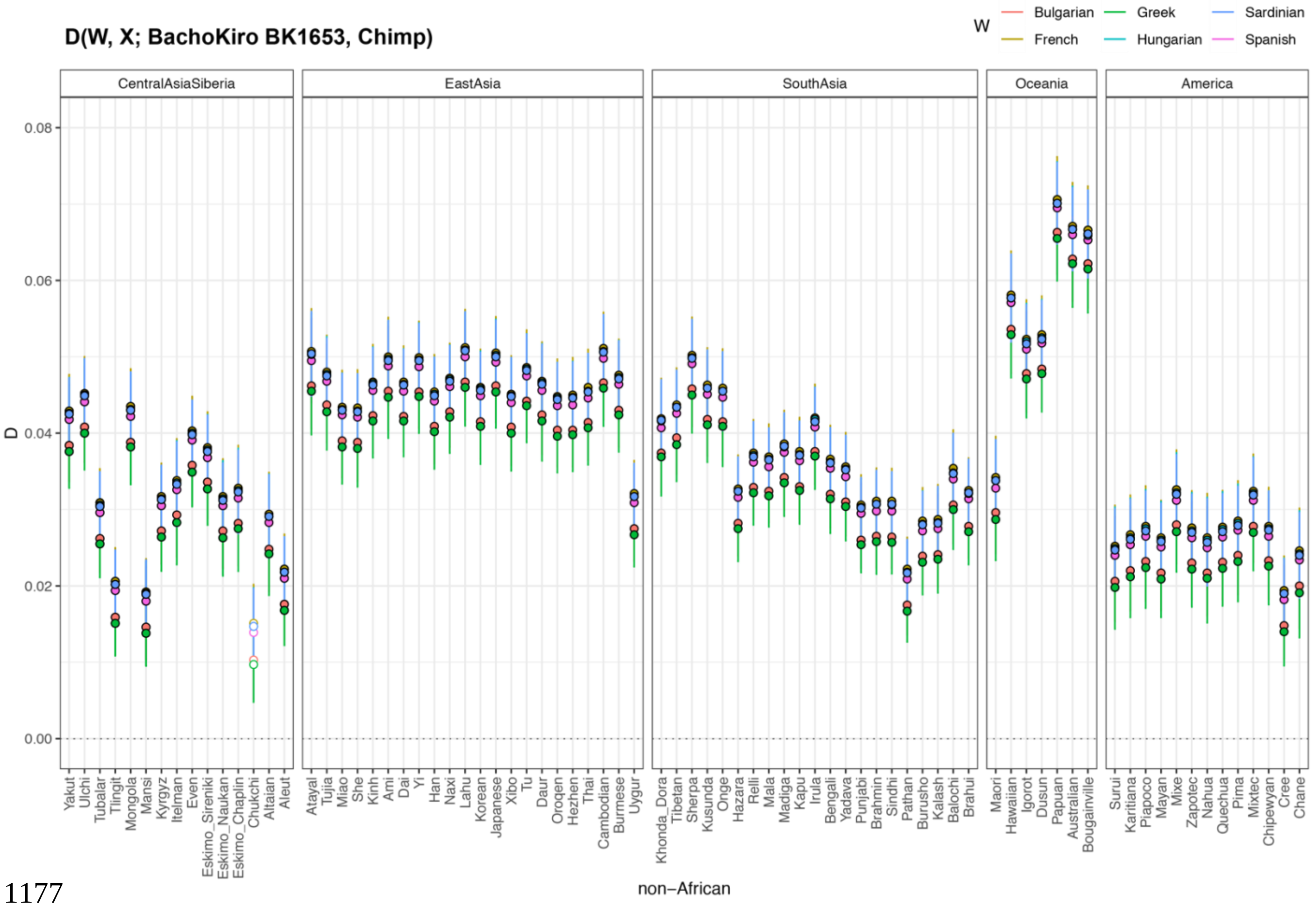


**Figure S5.11 D-statistics of the form *D(W, X; Bacho Kiro BK1653, Chimpanzee)* where *W* is one of the six present-day West Eurasian populations and *X* is a present-day non-African population from Central Asia and Siberia, East Asia, South Asia, Oceania or Americas.** *D* values denoted as circles and plotted on the *y*-axis were calculated using ADMIXTOOLS[3] as implemented in *admixr*[9]. Present-day human genomes from SGDP[10] were used in these statistics, and a genome of *panTro2* as an outgroup. *Bacho Kiro BK1653* (nsnps = 825,379) is significantly closer to present-day West Eurasians than to any other superpopulation from SGDP. Filled-in circles indicate a significant Z-score or |Z| ≥ 3, and open circles indicate a non-significant Z-score or |Z| < 3. Whiskers on each side of the plotted *D* values correspond to one standard error calculated using a Weighted Block Jackknife[3,12] across all autosomes on the “2200k” Panel and a block size of 5 Mb.

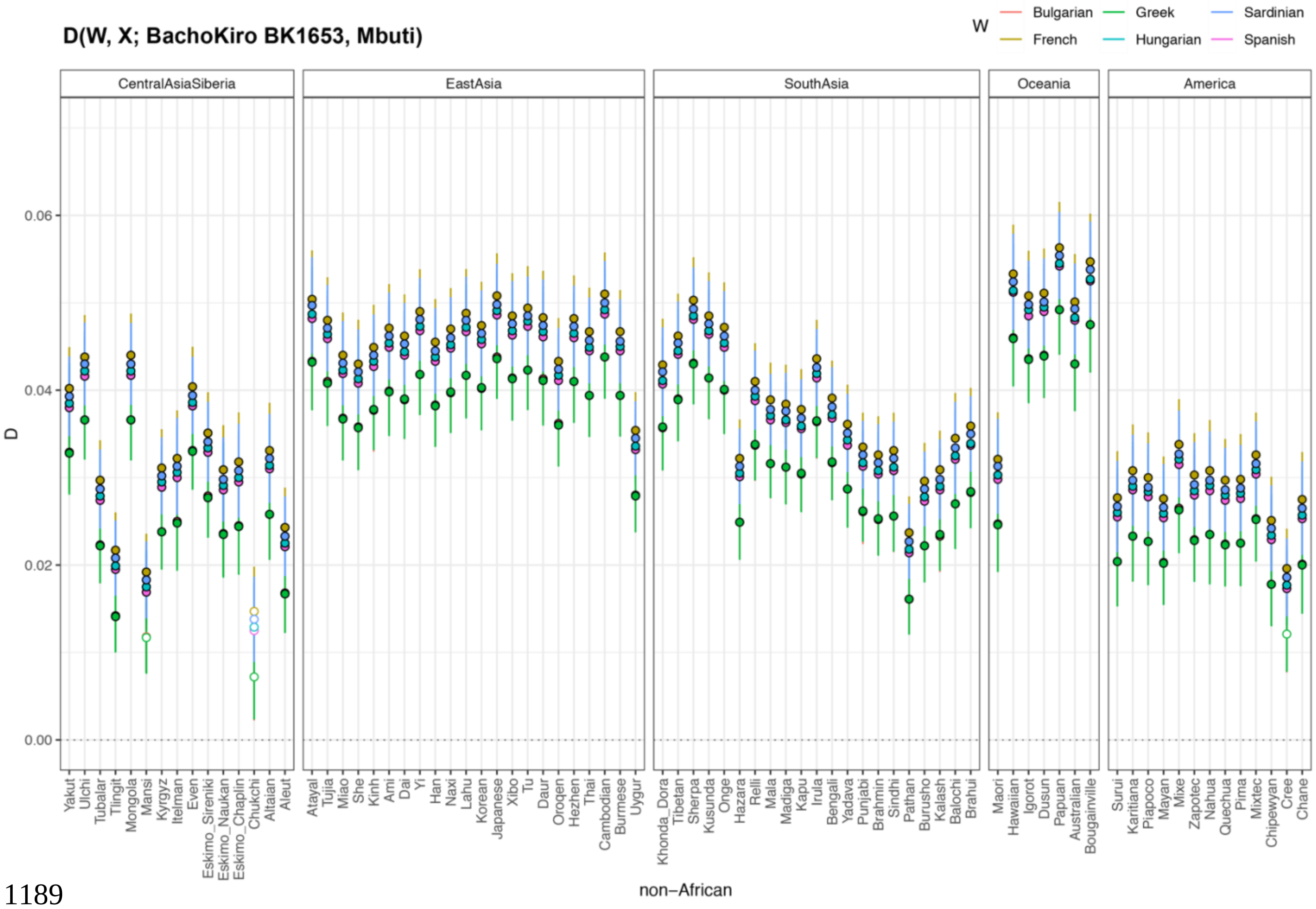


**Figure S5.12 D-statistics of the form *D(W, X; Bacho Kiro BK1653, Mbuti)* where *W* is one of the six present-day West Eurasian populations and *X* is a present-day non-African population from Central Asia and Siberia, East Asia, South Asia, Oceania or Americas.** *D* values denoted as circles and plotted on the *y*-axis were calculated using ADMIXTOOLS[3] as implemented in *admixr*[9]. Present-day human genomes from SGDP[10] were used in these statistics, and three Mbuti individuals from the same panel were used as outgroup. *Bacho Kiro BK1653* (nsnps = 825,379) is significantly closer to present-day West Eurasians than to any other superpopulation from SGDP. Filled-in circles indicate a significant Z-score or |Z| ≥ 3, and open circles indicate a non-significant Z-score or |Z| < 3. Whiskers on each side of the plotted *D* values correspond to one standard error calculated using a Weighted Block Jackknife[3,12] across all autosomes on the “2200k” Panel and a block size of 5 Mb.

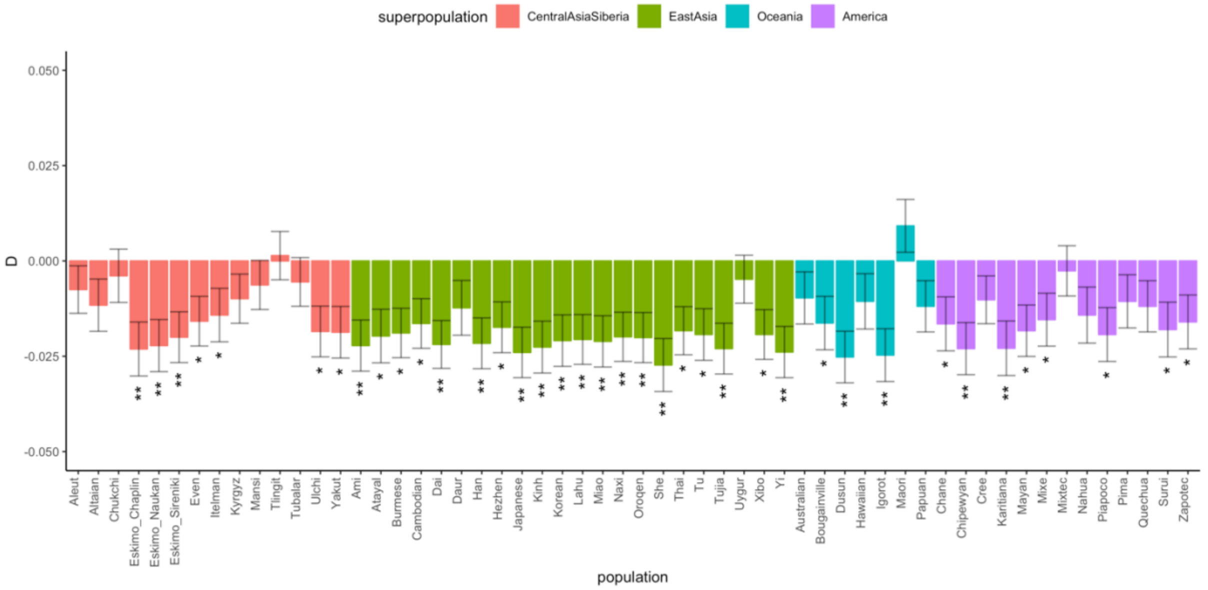


**Figure S5.13** ***D(Kostenki14, X; IUP BachoKiro, Mbuti)*** **restricted to transversion polymorphisms where** ***X*** **is a present-day non-African population from Central Asia and Siberia, East Asia, South Asia, Oceania or Americas.** *D* values denoted as bars plotted on the *y*-axis were calculated using ADMIXTOOLS[3] as implemented in *admixr*[9]. Positive *D*-value indicates higher proportion of alleles shared between IUP Bacho Kiro Cave individuals and *Kostenki 14*, whereas a negative *D*-value indicates higher proportion of alleles shared between IUP Bacho Kiro Cave individuals and a given present-day human population indicated on *x*-axis. Present-day human genomes from SGDP[10] were used in these statistics, and three Mbuti individuals from the same panel were used as outgroup. Two stars (**) indicate a significant Z-score or $|Z| \geq 3$, and one star (*) indicates $|Z| \geq 2$. Standard errors (SE) plotted around the centre of *D*-values were calculated using a Weighted Block Jackknife[3,12] across all autosomes on the "2200k" Panel (nsnps (IUP Bacho Kiro individuals) = 945,785) and a block size of 5 Mb.

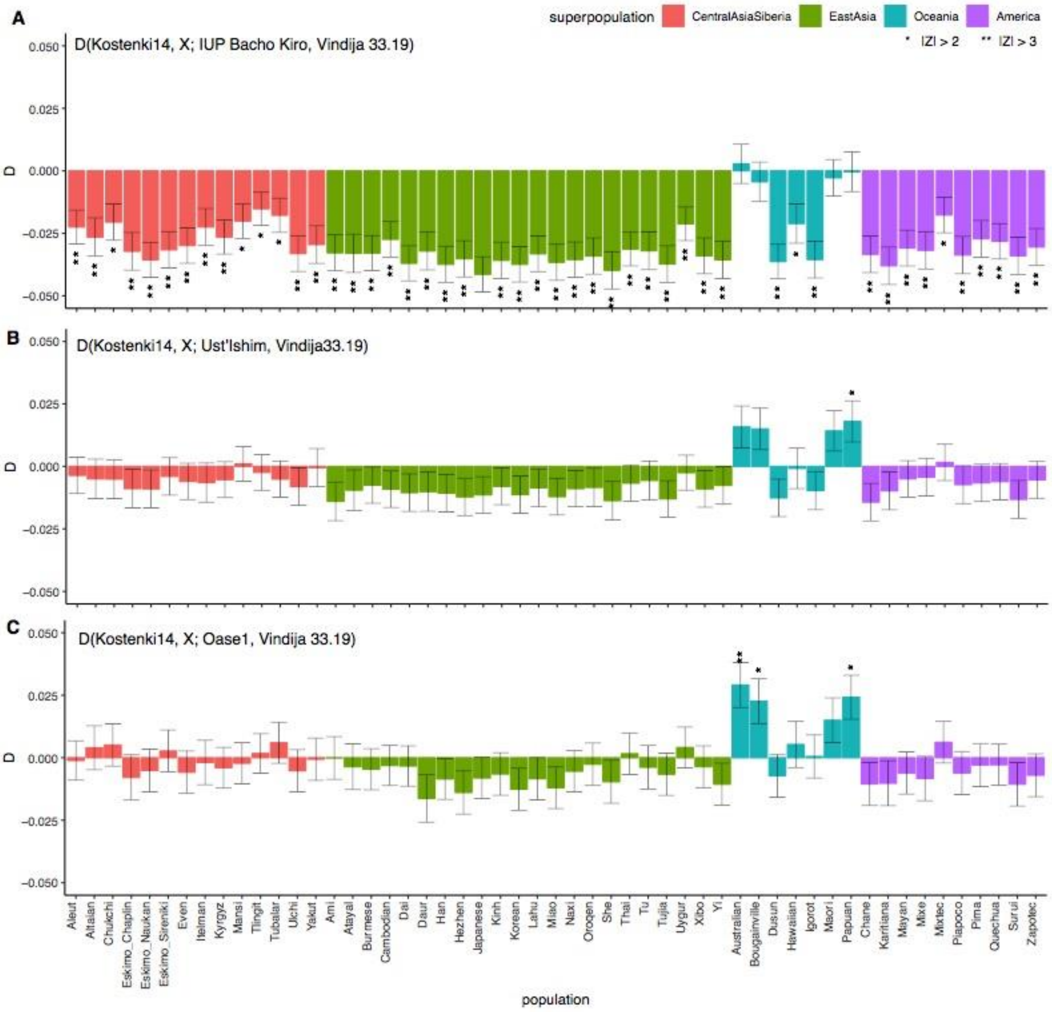


**Figure S5.14** ***D(Kostenki14, X; IUP BachoKiro/Ust'Ishim/Oase1, Vindija 33.19)*** **where *X* is a present-day non-African population from Central Asia and Siberia, East Asia, South Asia, Oceania or Americas. A)** IUP Bacho Kiro Cave individuals, **B)** *Ust'Ishim* and **C)** *Oase 1*. *D* values denoted as bars plotted on the *y*-axis were calculated using ADMIXTOOLS[3] as implemented in *admixr*[9]. Positive *D*-value indicates higher proportion of alleles shared between IUP Bacho Kiro Cave individuals (panel A), *Ust'Ishim* (panel B) or *Oase 1* (panel C) and *Kostenki 14*, whereas a negative *D*-value indicates higher proportion of alleles shared between former individuals and a given present-day human population indicated on *x*-axes. Present-day human genomes from SGDP[10] were used in these statistics, and *Vindija 33.19* Neandertal[17] was used as outgroup. Two stars (**) indicate a significant Z-score or $|Z| \geq 3$, and one star (*) indicates $|Z| \geq 2$. Standard errors (SE) plotted around *D*-values were calculated using a Weighted Block Jackknife[3,12] across all autosomes on the "2200k" Panel (nsnps (IUP Bacho Kiro individuals) = 1,813,821; nsnps (*Ust'Ishim*) = 1,951,462; nsnps (*Oase 1*) = 402,526) and a block size of 5 Mb.

# Supplementary Information 6

## Admixture Graph Modelling

We used program *qpGraph* (Admixture Graph) from ADMIXTOOLS[1] (version: v5.1) to test models of the relationship among Initial Upper Palaeolithic Bacho KiroCave individuals, ~34,000-year-old *Bacho Kiro BK1653* and other ancient modern humans from Eurasia older than 30,000 years BP. Admixture Graph examines whether a proposed model or a graph fits the data by determining whether the estimated values of all possible $f_2$-, $f_3$-, and $f_4$-statistics among all pairs, triples, and quadruples of individuals or populations match the observed values, and evaluates the significance of the difference between them using a Weighted Block Jackknife[1,2]. We run *qpGraph* on the "2200k" SNP Panel, using a Weighted Block Jackknife across all autosomes with a block size of 5 Mb and the following parameters:

outpop: NULL, blgsize: 0.005, lsqmode: YES, diag: .0001, hires: YES, lambdascale: 1, useallsnps: NO, bigiter: 6, forcezmode: YES

We begin with a simple model (Fig. S6.1) that relates three high coverage Mbuti individuals from SGDP[3], the ~45,000-year-old *Ust'Ishim* from Siberia[4], the ~40,000-year-old *Tianyuan*[5] from China, the ~38,000-year-old *Kostenki14*[6,7] and the ~34,000-year-old *SunghirIII*[8], both from Russia. We use the capture data of *Kostenki14*[7] for most of our *qpGraph* analyses due to the higher coverage of SNPs in the "2200k" Panel. In each of the Admixture Graphs shown in this supplementary note labels on solid branches correspond to the estimated drift in $f_2$-units of squared frequency difference and labels on the dotted edges give admixture proportions.

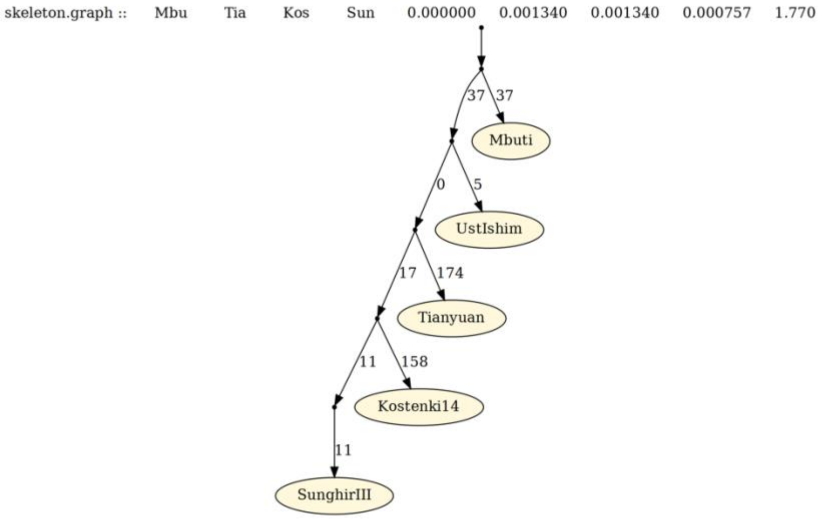


**Figure S6.1 Base (skeleton) graph.** This model uses 886,336 SNPs covered in all individuals.

We proceed by attempting to fit the ~45,000-year-old Initial Upper Palaeolithic (IUP) Bacho Kiro individual with highest coverage (*Bacho Kiro F6-620*) to this base graph. We add IUP *Bacho Kiro F6-620* to all possible nodes of the graph in Fig. S6.1, either as a simple branch without mixture, or as a mixture between branches. Altogether, we identify two models that fit the data (maximum |Z-score| < 3) and show these in Fig. S6.2. In both graphs the 40,000-year-old *Tianyuan* is modelled as a mixture with a lineage related to *Bacho Kiro F6-620*.

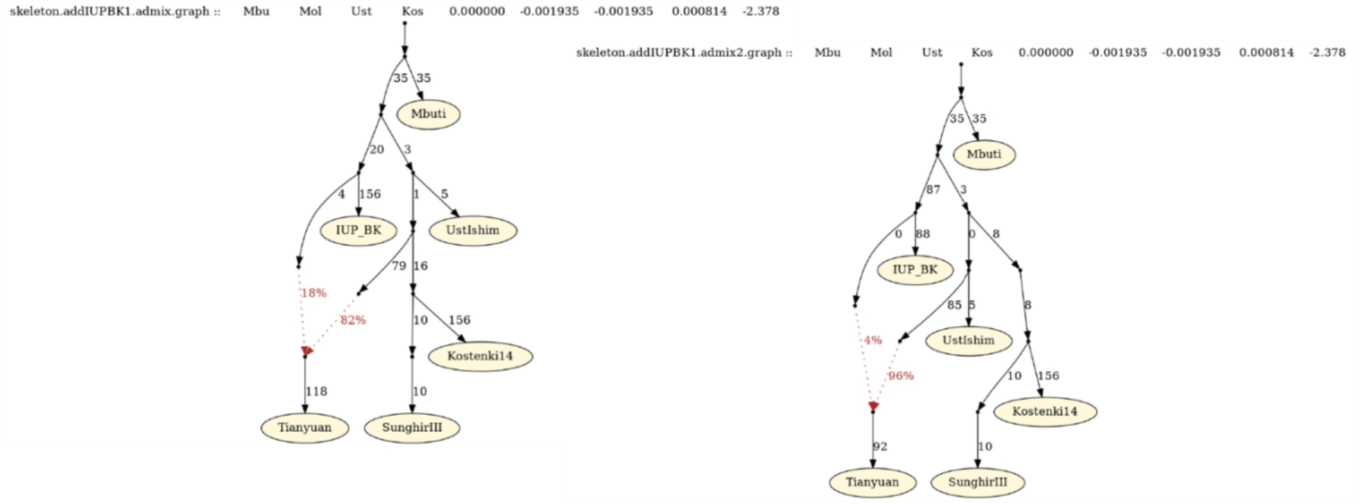


**Figure S6.2 Adding *Bacho Kiro F6-620* to the base graph.** These models use 879,787 SNPs in all individuals.

Given the higher Neandertal ancestry in *Bacho Kiro F6-620* than in other ancient modern humans included in these models (see Supplementary Information 7 and 8), we account for this and for the archaic ancestry in other ancient modern humans by adding the high coverage genomes of *Vindija 33.19* Neandertal[9] and *Denisova 3*[10] to the graphs in Fig. S6.2.

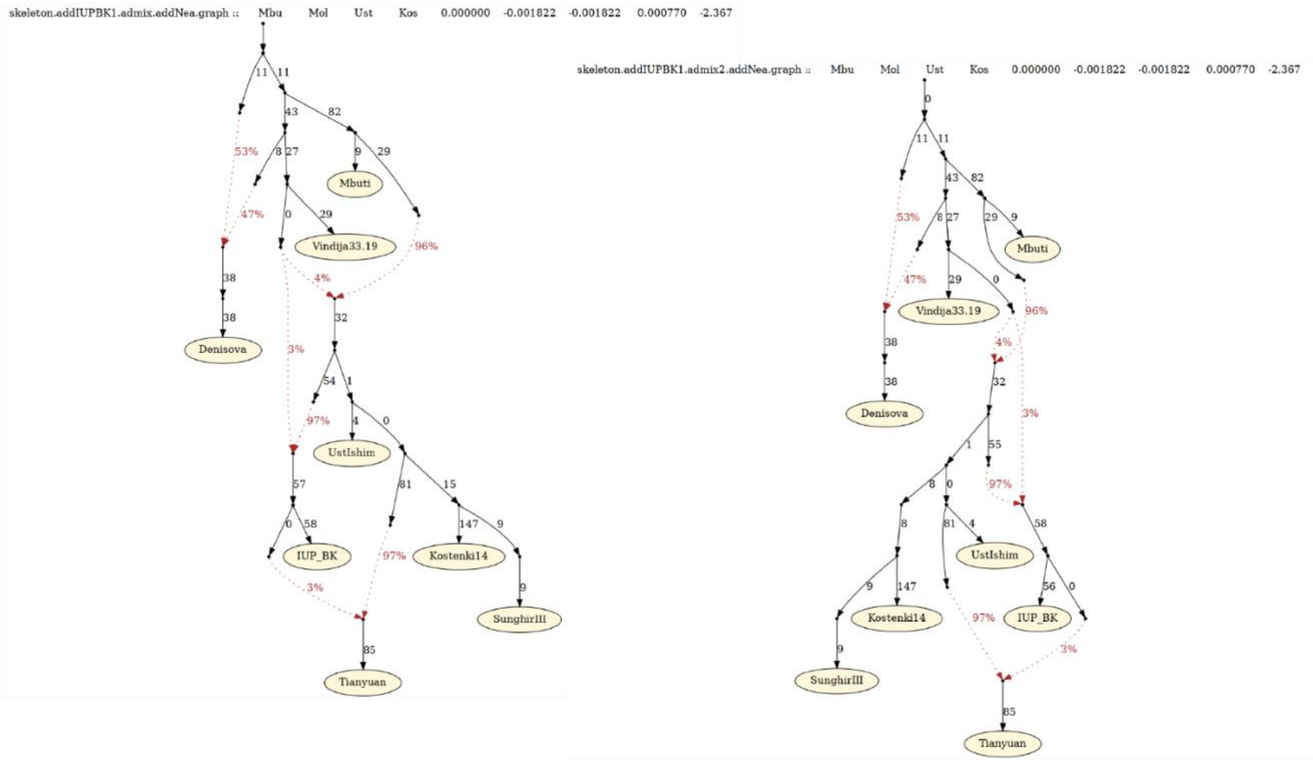


**Figure S6.3 Adding *Vindija 33.19* Neandertal and *Denisova 3*.** These models use 832,495 SNPs in all individuals.

We further proceed to fit the ~35,000-year-old *GoyetQ116-1* from Belgium[7], the younger ~34,000-year-old *Bacho Kiro BK1653* and the ~30,000-year-old *Vestonice16* from Czech Republic[7] to the graphs in Fig. S6.3, starting with the oldest individual and moving forward in time.

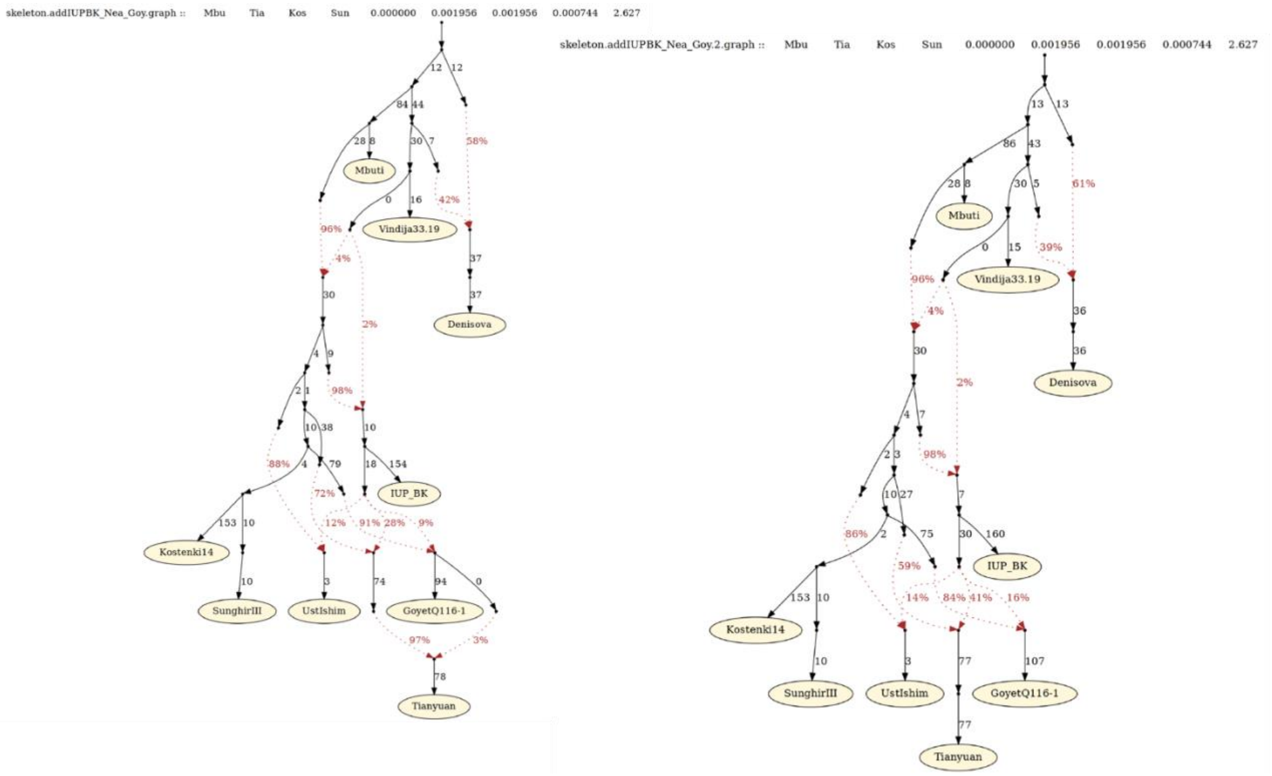


**Figure S6.4 Adding the ~35,000-year-old *Goyet Q116-1* from Belgium.** These models use 520,102 SNPs in all individuals.

Interestingly, after adding the ~35,000-year-old *Goyet Q116-1* to all the possible nodes shown in the Fig. S6.3 either as a simple branch without mixture or as a mixture between branches, the ~45,000-year-old *Ust'Ishim*, the ~40,000-year-old *Tianyuan* and *Goyet Q116-1* all fit the models best as being admixed with a lineage related to the *Bacho Kiro F6-620*, with *Tianyuan* having the largest contribution from this lineage (between 28% and 41%, depending on the model). Furthermore, having part of the ancestry of *GoyetQ116-1* deriving from a lineage related to IUP Bacho Kiro eliminates the need for an additional admixture edge between *GoyetQ116-1* and *Tianyuan* (Fig. S6.4). We note, however, that both models with and without this additional admixture edge fit the data with no outliers (maximum |Z-score| < 3) (Fig. S6.4). We also tested whether the connections observed between *Ust'Ishim* or *GoyetQ116-1* and *Bacho Kiro F6-620* could be explained by the underlying models requiring an additional admixture edge from Neandertals to these individuals. However, these models are rejected (|Z-score| > 3) in favour of the models presented in Fig. S6.4.

We next add the ~34,000-year-old *Bacho Kiro 1653* to the models in Fig. S6.4 either as a simple branch without mixture or as a mixture between branches. We infer for this individual to be best modelled as admixed between a lineage related to *GoyetQ116-1* and a lineage related to *Kostenki14/SunghirIII*, with one outlier remaining (*f4(Mbuti, Tianyuan; Kostenki14, Sunghir)*, Z-score = 3.01). In the models highlighted in the Fig. S6.5, an additional admixture

edge stemming from a deep Eurasian branch that splits before the separation of *IUP Bacho Kiro, Ust'Ishim* and other Eurasians is required to explain the data. These models fit the data best to the limits of our resolution, albeit with a single outlier (Z-score = 3.01). To investigate this outlier further, we compute the statistics *D(Mbuti, Tianyuan; Kostenki14, SunghirIII)* and obtain a Z-score of 1.56 (all SNPs). Given the number of statistics we are computing to test the fit of these models to the data, we do not view a single outlier at |Z|>3 as a strong rejection of the models highlighted in Fig. S6.5.

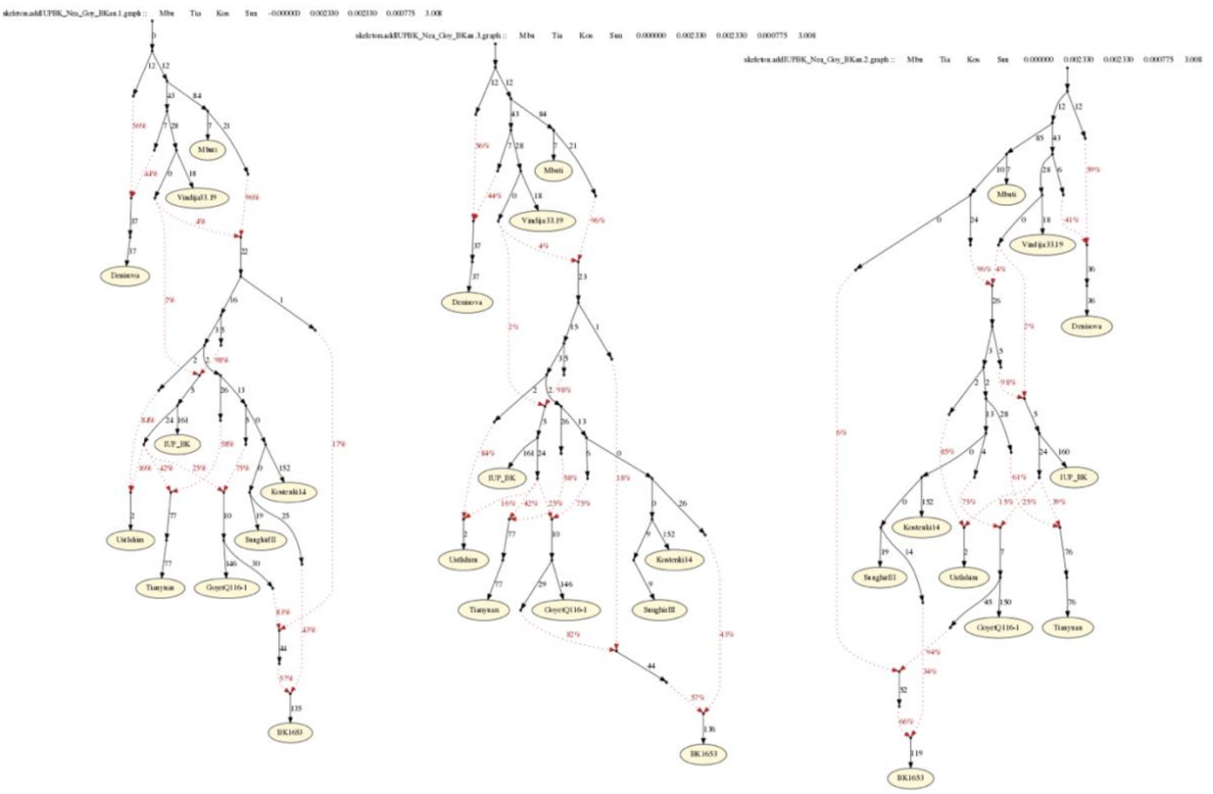

**Figure S6.5 Adding 34,000-year-old *Bacho Kiro BK1653*.** These models use 326,550 SNPs in all individuals.

We added the ~30,000-year-old *Vestonice16* to all possible branches of the models outlined in the Fig. S6.5, removing in each case the deep branching lineage contributing to *Bacho Kiro BK1653*. Two models fit the data best (Fig. S6.6 and Fig. S6.7), albeit each with one outlier and still requiring a deep branching lineage, with either *Vestonice16* being admixed between lineages related to *Bacho Kiro BK1653* and a lineage related to *Kostenki14/SunghirIII* or *Bacho Kiro BK1653* being admixed between the lineages related to *GoyetQ116-1* and *Vestonice16*. In each case we are left with a single outlier to the data, *f4(Tianyuan, SunghirIII; GoyetQ116-1, BachoKiro BK1653)* (Z-score = 3.22) and *f4(Mbuti, Vestonice16; Kostenki14, Vestonice16)* (Z-score = 3.67).

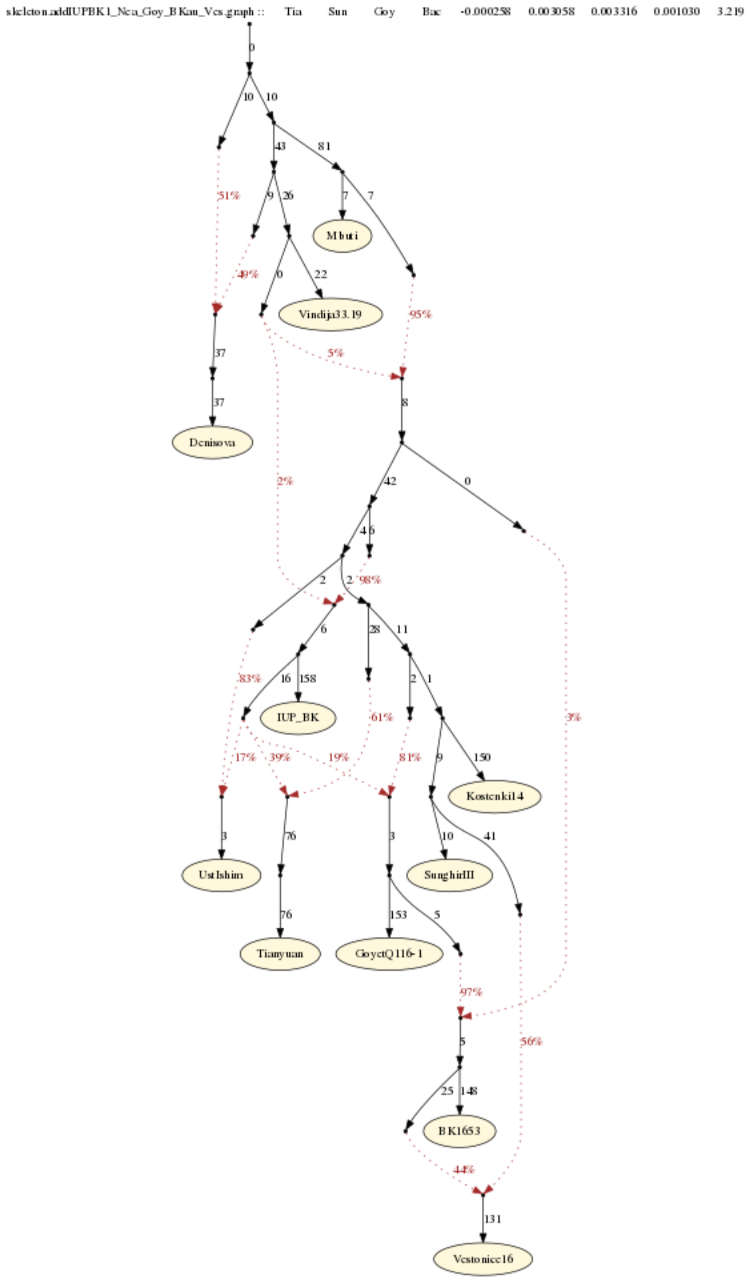


**Figure S6.6 Adding *Vestonice16*, with *Vestonice16* being admixed.** This model uses 281,732 SNPs in all individuals.

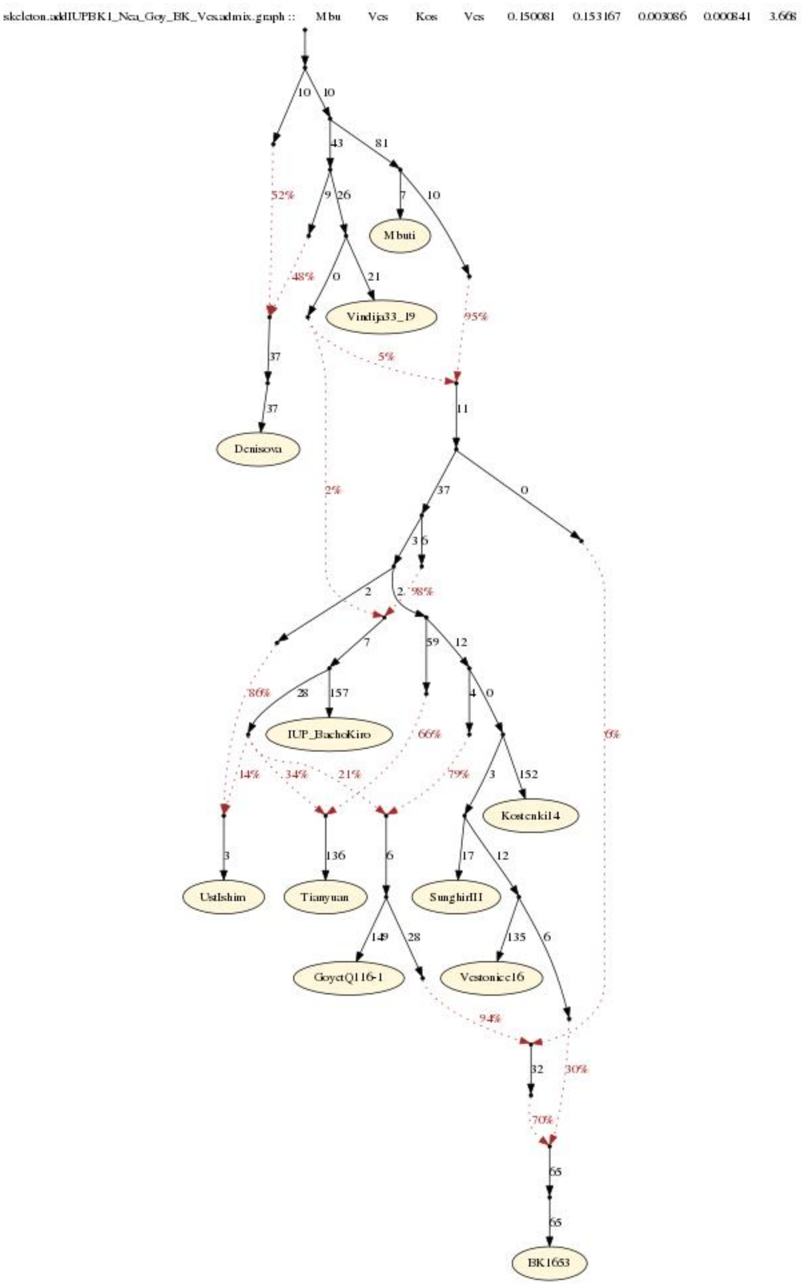


**Figure S6.7 Adding *Vestonice16*, with *BachoKiro BK1653* being admixed.** This model uses 281,732 SNPs in all individuals.

We additionally explored the relationship of *Bacho Kiro BK1653* to other Upper Palaeolithic West Eurasian ancient modern humans by reducing the graph to a smaller number of individuals (Fig S6.8). We confirm the placement of *Bacho Kiro BK1653* as being closest to a lineage related to *GoyetQ116-1*, further contributing her ancestry to *Vestonice16*, or alternatively being an admixed individual herself between the lineages related to *GoyetQ116-1* and *Kostenki14/SunghirIII* or *Vestonice16* and *GoyetQ116-1*. All of these models fit with the data with no outliers, albeit with invoking a very small contribution of a lineage that splits off from *IUP Bacho Kiro, Ust'Ishim* and other Eurasians before they split from each other.

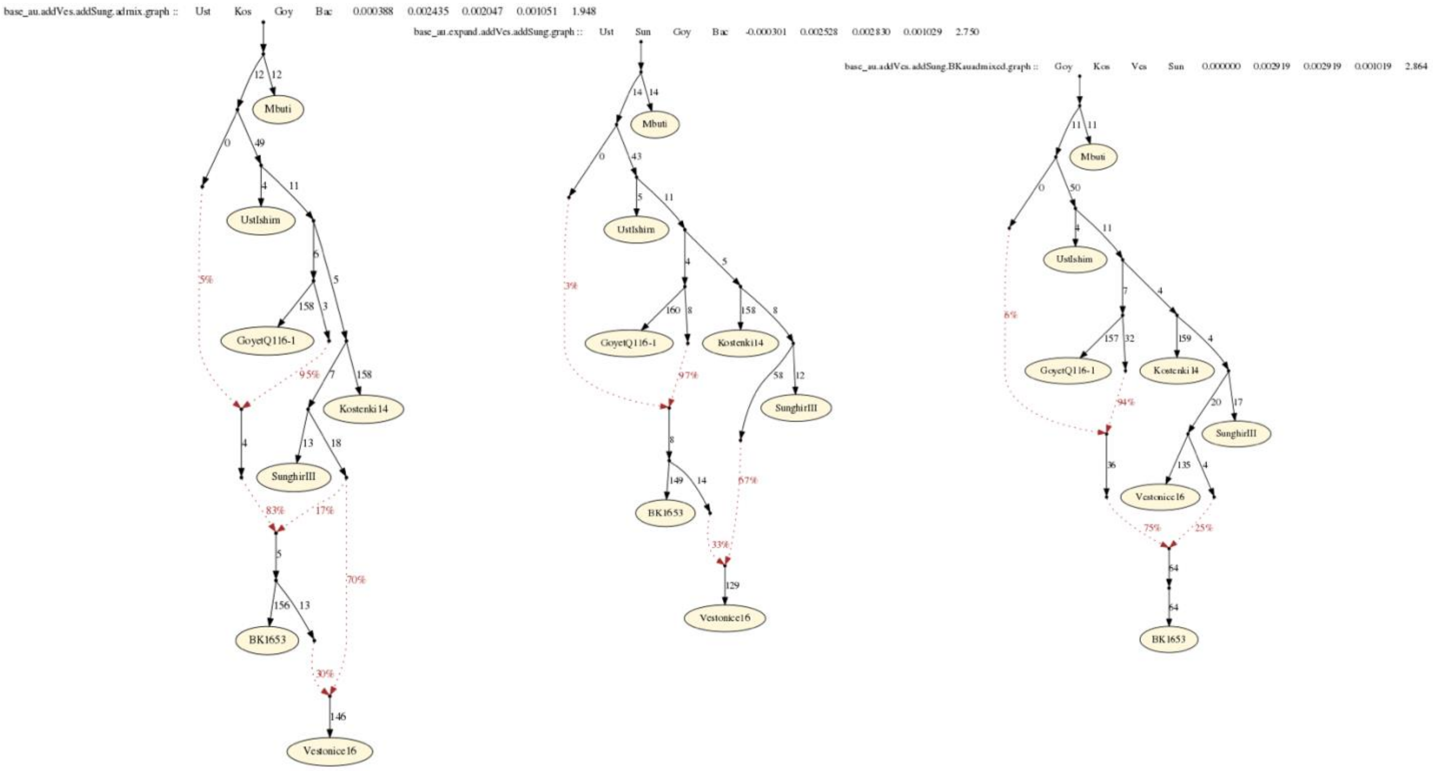


**Figure S6.8 Admixture Graphs relating smaller number of ancient West Eurasians older than 30,000 years BP.** These models use 327,698 SNPs in all individuals.

**References SI6:**


1 Patterson, N. *et al.* Ancient admixture in human history. *Genetics* **192**, 1065-1093, doi:10.1534/genetics.112.145037 (2012).

2 Busing, F. M. T. A., Meijer, E. & Van Der Leeden, R. Delete-m jackknife for unequal m. *Statistics and Computing* **9**, 3-8, doi:Doi 10.1023/A:1008800423698 (1999).

3 Mallick, S. *et al.* The Simons Genome Diversity Project: 300 genomes from 142 diverse populations. *Nature* **538**, 201-206, doi:10.1038/nature18964 (2016).

4 Fu, Q. *et al.* Genome sequence of a 45,000-year-old modern human from western Siberia. *Nature* **514**, 445-449, doi:10.1038/nature13810 (2014).

5 Yang, M. A. *et al.* 40,000-Year-Old Individual from Asia Provides Insight into Early Population Structure in Eurasia. *Curr. Biol.* **27**, 3202-3208.e9, doi: 10.1016/j.cub.2017.09.030 (2017).

6 Seguin-Orlando, A. *et al.* Genomic structure in Europeans dating back at least 36,200 years. *Science* **346**, 1113-1118, doi:10.1126/science.aaa0114 (2014).

7 Fu, Q. *et al.* The genetic history of Ice Age Europe. *Nature* **534**, 200-205, doi:10.1038/nature17993 (2016).

8 Sikora, M. *et al.* Ancient genomes show social and reproductive behavior of early Upper Paleolithic foragers. *Science* **358**, 659-662, doi: 10.1126/science.aao1807 (2017).

9 Prüfer, K. *et al.* A high-coverage Neandertal genome from Vindija Cave in Croatia. *Science* **358**, 655-658, doi: 10.1126/science.aao1887 (2017).

10 Meyer, M. *et al.* A high-coverage genome sequence from an archaic Denisovan individual. *Science* **338**, 222-226, doi:10.1126/science.1224344 (2012).

# Supplementary Information 7

## Neandertal ancestry in Bacho Kiro individuals

**Estimates of Neandertal ancestry proportions**

We calculated the proportion of Neandertal ancestry in Bacho Kiro individuals and other ancient and present-day modern humans by computing a direct $f_4$*-ratio* that takes advantage of the two high-coverage Neandertal genomes[1], the ~130,000-year-old *Denisova 5* (*'Altai')* Neandertal from Siberia[2] and the ~45,000-year-old *Vindija 33.19* Neandertal from Croatia[3]. We also re-calculated the proportion of Neandertal ancestry in *Oase1* after merging previously published data[4] with new data from additional libraries prepared for this study (see Supplementary Information 2). We used ADMIXTOOLS[5] as implemented in the R package *admixr*[6], with a Weighted Block Jackknife[5,7] and a block size of 5 million base pairs (5Mb) to calculate alpha ($\alpha$) on the "2200k" SNP Panel as:

$$\alpha = \frac{f4(Altai\ Neandertal, Chimpanzee; X, Mbuti)}{f4(Altai\ Neandertal, Chimpanzee; Vindija\ 33.19\ Neandertal, Mbuti)}$$

We find that Bacho Kiro individuals *F6-620*, *BB7-240* and *CC7-335* have 3.82% (95% confidence interval (CI): 3.28-4.37%), 3.01% (95% CI: 2.43-3.58%) and 3.43% (95% CI: 2.84-4.02%) Neandertal ancestry, respectively, which is more than the average of 1.95% (95% CI: 1.52-2.38%) found in other Upper Palaeolithic modern humans older than 30,000 years BP, with the exception of *Oase1*[4] (6.39% (95% CI: 5.65-7.13%)) (Extended Data Figure 6A). In contrast, Bacho Kiro *BK1653* has 1.91% (95% CI: 1.44-2.37%) Neandertal ancestry, similar to other ancient and present-day modern humans (Extended Data Figure 6A).

To test formally if IUP Bacho Kiro individuals share significantly more derived alleles with archaics than other ancient and present-day humans, we computed *D(IUP Bacho Kiro, Test; Neandertal, Outgroup)*. We used the genomes of Dinka, Yoruba and Mbuti individuals from the Simons Genome Diversity Panel (SGDP)[8] as outgroups. Since these statistics are expected to be skewed significantly positive for the Upper Palaeolithic individuals such as the ~45,000-year-old *Ust'Ishim* (Fig. S7.4) and the ~38,000-year-old *Kostenki14* (Fig. S7.6) due to gene-flow with Africans[1] or when a *Test* individual post-dates the introduction of "Basal Eurasian" ancestry[9,10], we compute the same statistics using the genome of a chimpanzee (*panTro2*) as an outgroup and restricting analyses to transversion polymorphisms to mitigate the effect of ancient DNA damage (Fig. S7.8-S7.14).

We find that IUP Bacho Kiro Cave individuals share significantly more derived alleles with the three high coverage Neandertals[2,3,11] than most other Upper Palaeolithic ancient humans (Fig. S7.1-S7.3 and Fig. S7.8-S7.10) with an exception of *Oase1*[4] (Z-score < -3.01). They also share significantly more alleles with Neandertals than present-day non-Africans (Fig. S7.8-S7.10), similar to *Oase1* (Fig. S7.12) and unlike *Ust'Ishim* (Fig. S7.11), *Kostenki14* (Fig. S7.13) or Bacho Kiro *BK1653* (Fig. S7.14). When present-day Papuans, Australians and Bougainville individuals are used as a *Test* population, the statistics *D(IUP Bacho Kiro, Test; Neandertal, Outgroup)* are not significantly different from 0 (Fig. S7.8-S7.10), likely due to the additional gene flow from Denisovans, a sister group of Neandertals, into these populations[12-15].

**Introgressing Neandertal(s)**

We tested if IUP Bacho Kiro Cave individuals share more derived alleles with one of the already sequenced Neandertal genomes by calculating *D(*$Archaic_1$*,* $Archaic_2$*; IUP Bacho Kiro, Outgroup)*. We first investigated whether there is a significant difference in derived allele sharing with the genomes of the ~50,000-year-old *Denisova 3* individual and any of the high coverage Neandertals (~130,000-year-old *Altai*[2] Neandertal, ~80,000-year-old *Chagyrskaya 8*[11] and ~45,000-year-old *Vindija 33.19*[3]) or if there are differences in allele sharing when comparing between the high coverage Neandertals. For the high-coverage genomes of the archaics we used their *snpAD*[3,16] genotype calls that overlap positions on the "2200k" SNP Panel. We used the genomes of *panTro2*, and Dinka, Yoruba and Mbuti individuals from the Simons Genome Diversity Panel (SGDP)[8] as outgroups.

We find that Bacho Kiro individuals share significantly more derived alleles with Neandertals than with the Denisovan individual (Z-score < -26.27, Fig. S7.15 and S7.16). Furthermore, the three IUP Bacho Kiro individuals and *BK1653* share significantly more derived alleles with *Chagyrskaya 8* and *Vindija 33.19* than they do with the *Altai* Neandertal (-5.08 < Z-score < -2.77, Fig. S7.15, and -5.03 < Z-score < -3.01, Fig. S7.16), similar to what has been previously observed for other ancient and present-day humans[3,11,17].

**Investigating overlapping sites with late Neandertals and *Mezmaiskaya1***

Given that IUP Bacho Kiro Cave individuals overlap in time with late Neandertals, we co-analysed the low-coverage late Neandertal genomes of *Les Cottés Z4-1514, Goyet Q56-1, Mezmaiskaya 2* and *Spy 94a*[17] with the genome of a >70,000-year-old *Mezmaiskaya 1* Neandertal[3] and the high-coverage archaic genomes. For the late Neandertals and *Mezmaiskaya 1*, we randomly sampled an allele from fragments longer than 35 base pairs that had a mapping quality of at least 25 and that overlapped positions on the "2200k" SNP Panel. The analyses involving low-coverage Neandertals were further restricted to the fragments that showed C-to-T substitutions with respect to the human reference genome in the first and/or last three positions to diminish the impact of present-day human DNA contamination[18] that was previously inferred for these samples[3,17]. To make these analyses comparable with those of high-coverage archaics and minimize the differences in the statistics that involve genomes of substantially different qualities, we followed the same filtering as above for the high coverage *Altai* and *Vindija 33.19* Neandertals and randomly sampled an allele from fragments longer than 35 base pairs that had a mapping quality of at least 25 and that overlapped positions on the "2200k" SNP Panel, thereby effectively lowering the qualities of the high coverage genomes to similar levels as the low-coverage Neandertals.

However, the number of informative sites in low-coverage Neandertals that overlap "2200k" SNP Panel is low (Tab. S7.1). This is best demonstrated calculating *D(Altai Neandertal, Vindija33.19; Modern Human Test, Outgroup)* where down-sampling high-coverage Neandertal genomes to the coverage of late Neandertals and *Mezmaiskaya1* causes otherwise significantly positive statistics (Fig. S7.15 and Fig. S7.16) to become not significantly different from 0 (Fig. S7.17). Thus, we do not have enough power to detect any potential differences among late Neandertal populations that contributed additional Neandertal ancestry to IUP Bacho Kiro individuals when compared to other ancient modern humans, even if such differences may exist.

**References SI7:**

1 Petr, M., Pääbo, S., Kelso, J. & Vernot, B. Limits of long-term selection against Neandertal introgression. *Proc. Natl. Acad. Sci. U.S.A.* **116**, 1639-1644 (2019).

2 Prüfer, K. *et al.* The complete genome sequence of a Neanderthal from the Altai Mountains. *Nature* **505**, 43-49, doi:10.1038/nature12886 (2014).

3 Prüfer, K. *et al.* A high-coverage Neandertal genome from Vindija Cave in Croatia. *Science* **358**, 655-658, doi: 10.1126/science.aao1887 (2017).

4 Fu, Q. *et al.* An early modern human from Romania with a recent Neanderthal ancestor. *Nature* **524**, 216-219, doi:10.1038/nature14558 (2015).

5 Patterson, N. *et al.* Ancient admixture in human history. *Genetics* **192**, 1065-1093, doi:10.1534/genetics.112.145037 (2012).

6 Petr, M., Vernot, B. & Kelso, J. admixr—R package for reproducible analyses using ADMIXTOOLS. *Bioinformatics* **35**, 3194-3195 (2019).

7 Busing, F. M. T. A., Meijer, E. & Van Der Leeden, R. Delete-m jackknife for unequal m. *Statistics and Computing* **9**, 3-8, doi:Doi 10.1023/A:1008800423698 (1999).

8 Mallick, S. *et al.* The Simons Genome Diversity Project: 300 genomes from 142 diverse populations. *Nature* **538**, 201-206, doi:10.1038/nature18964 (2016).

9 Lazaridis, I. *et al.* Ancient human genomes suggest three ancestral populations for present-day Europeans. *Nature* **513**, 409-413, doi:10.1038/nature13673 (2014).

10 Lazaridis, I. *et al.* Genomic insights into the origin of farming in the ancient Near East. *Nature* **536**, 419-424 (2016).

11 Mafessoni, F. *et al.* A high-coverage Neandertal genome from Chagyrskaya Cave. *Proc. Natl. Acad. Sci. U.S.A.* **117**, 15132-15136, doi:10.1073/pnas.2004944117 (2020).

12 Reich, D. *et al.* Genetic history of an archaic hominin group from Denisova Cave in Siberia. *Nature* **468**, 1053-1060, doi:10.1038/nature09710 (2010).

13 Meyer, M. *et al.* A high-coverage genome sequence from an archaic Denisovan individual. *Science* **338**, 222-226, doi:10.1126/science.1224344 (2012).

14 Vernot, B. *et al.* Excavating Neandertal and Denisovan DNA from the genomes of Melanesian individuals. *Science* **352**, 235-239, doi:10.1126/science.aad9416 (2016).

15 Sankararaman, S., Mallick, S., Patterson, N. & Reich, D. The Combined Landscape of Denisovan and Neanderthal Ancestry in Present-Day Humans. *Curr. Biol.* **26**, 1241-1247, doi:10.1016/j.cub.2016.03.037 (2016).

16 Prüfer, K. snpAD: An ancient DNA genotype caller. *Bioinformatics* **34**, 4165-4171 (2018).

17 Hajdinjak, M. *et al.* Reconstructing the genetic history of late Neanderthals. *Nature* **555**, 652-656, doi:10.1038/nature26151 (2018).

18 Meyer, M. *et al.* A mitochondrial genome sequence of a hominin from Sima de los Huesos. *Nature* **505**, 403-406, doi:10.1038/nature12788 (2014).

19 Fu, Q. *et al.* The genetic history of Ice Age Europe. *Nature* **534**, 200-205, doi:10.1038/nature17993 (2016).

**Table S7.1 Number of informative sites in late Neandertals and *Mezmaiskaya1* overlapping "2200k" SNP Panel after filtering for fragments ≥35 bp and MQ≥25 that showed C-to-T substitutions in the first three and/or the last three positions relative to the reference genome.**

| Individual | # of SNPs | # of transversion SNPs |
|---|---|---|
| *Les Cottes Z4-1514* | 1,074,561 | 587,467 |
| *Spy 94a* | 330,889 | 182,085 |
| *Goyet Q56-1* | 446,309 | 238,416 |
| *Mezmaiskaya 2* | 760,142 | 402,957 |
| *Mezmaiskaya 1* | 546,819 | 306,969 |

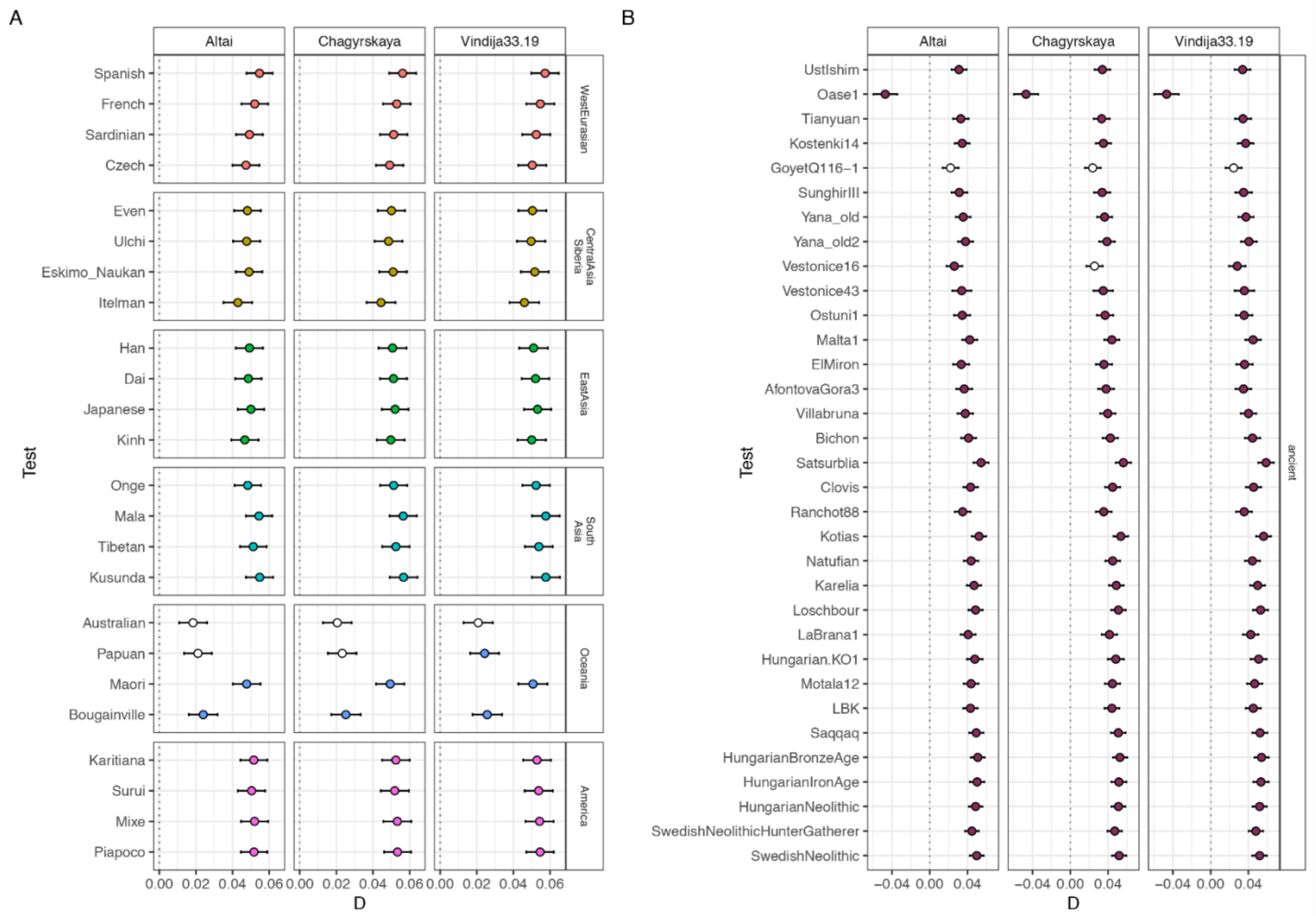


**Figure S7.1 D-statistics comparing derived allele sharing with an archaic genome between IUP Bacho Kiro *F6-620* and a range of present-day and ancient modern humans.** Values for *D(Bacho Kiro F6-620, Test; Archaic, Mbuti)* denoted as circles are plotted on the *x*-axes where *Archaic* is a high-coverage Neandertal genome[2,3,11] and *Test* is either a present-day population from SGDP[8] (panel A) or an ancient individual (panel B). Three Mbuti individuals from SGDP[8] were used as outgroup. *D* statistics were calculated using ADMIXTOOLS[5] as implemented in *admixr*[6]. Filled-in circles indicate a significant Z-score or $|Z| \geq 3$, and open circles indicate a non-significant Z-score or $|Z| < 3$. Whiskers on each side of the plotted *D* values correspond to one standard error calculated with a Weighted Block Jackknife[5,7] and a block size of 5 Mb across all autosomes on the “2200k” Panel (nsnps (*Bacho Kiro F6-620*) = 1,779,883).

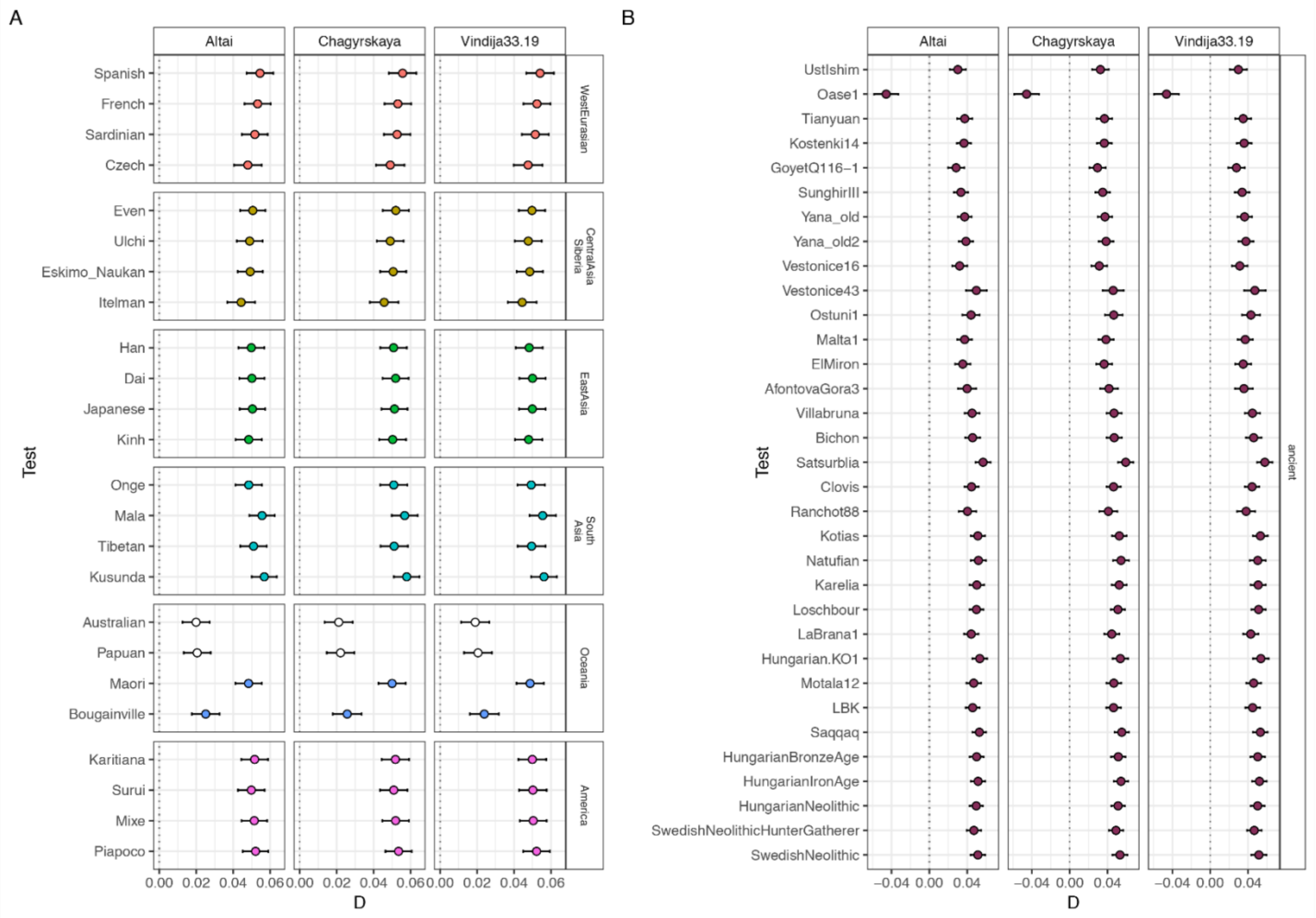


**Figure S7.2 D-statistics comparing derived allele sharing with an archaic genome between IUP Bacho Kiro *BB7-240* and a range of present-day and ancient modern humans.** Values for *D(Bacho Kiro BB7-240, Test; Archaic, Mbuti)* denoted as circles are plotted on the *x*-axes where *Archaic* is a high-coverage Neandertal genome[2,3,11] and *Test* is either a present-day population from SGDP[8] (panel A) or an ancient individual (panel B). Three Mbuti individuals from SGDP[8] were used as outgroup. *D* statistics were calculated using ADMIXTOOLS[5] as implemented in *admixr*[6]. Filled-in circles indicate a significant Z-score or $|Z| \geq 3$, and open circles indicate a non-significant Z-score or $|Z| < 3$. Whiskers on each side of the plotted *D* values correspond to standard errors calculated with a Weighted Block Jackknife[5,7] and a block size of 5 Mb across all autosomes on the “2200k” Panel (nsnps (*Bacho Kiro BB7-240*) = 787,706).

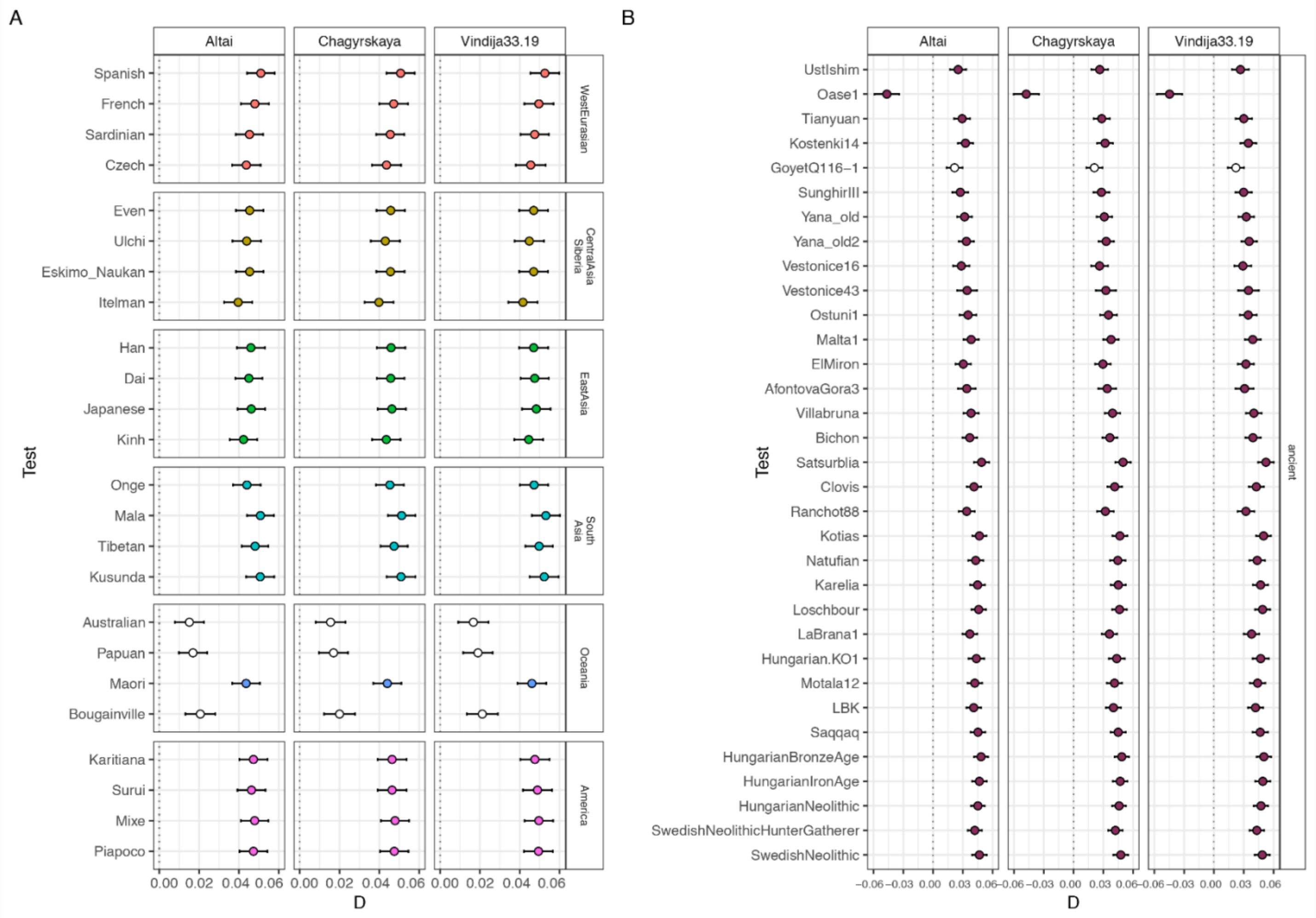


**Figure S7.3 D-statistics comparing derived allele sharing with an archaic genome between IUP Bacho Kiro CC7-335 and a range of present-day and ancient modern humans.** Values for *D(Bacho Kiro CC7-335, Test; Archaic, Mbuti)* denoted as circles are plotted on the *x*-axes where *Archaic* is a high-coverage Neandertal[2,3,11] genome and *Test* is either a present-day population from SGDP[8] (panel A) or an ancient individual (panel B). Three Mbuti individuals from SGDP[8] were used as outgroup. *D* statistics were calculated using ADMIXTOOLS[5] as implemented in *admixr*[6]. Filled-in circles indicate a significant Z-score or |Z| ≥ 3, and open circles indicate a non-significant Z-score or |Z| < 3. Whiskers on each side of the plotted *D* values correspond to one standard error calculated using a Weighted Block Jackknife[5,7] and a block size of 5 Mb across all autosomes on the “2200k” Panel (nsnps (*Bacho Kiro CC7-335*) = 723,129).

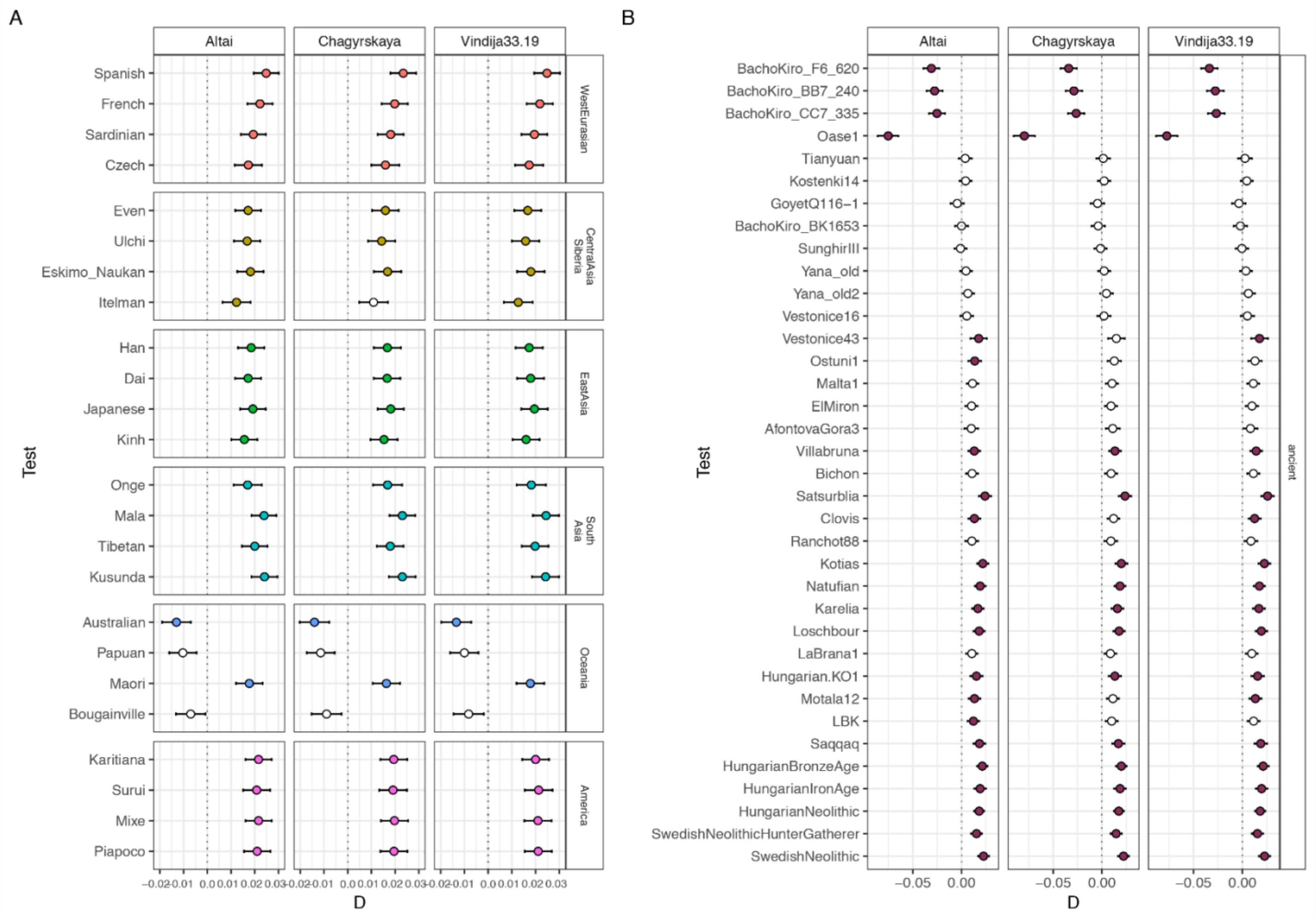


**Figure S7.4 D-statistics comparing derived allele sharing with an archaic genome between ~45,000-year-old *Ust'Ishim* from Siberia and a range of present-day and ancient modern humans.** Values for *D(Ust'Ishim, Test; Archaic, Mbuti)* denoted as circles are plotted on the *x*-axes where *Archaic* is a high-coverage Neandertal[2,3,11] genome and *Test* is either a present-day population from SGDP[8] (panel A) or an ancient individual (panel B). Three Mbuti individuals from SGDP[8] were used as outgroup. *D* statistics were calculated using ADMIXTOOLS[5] as implemented in *admixr*[6]. Filled-in circles indicate a significant Z-score or |Z| ≥ 3, and open circles indicate a non-significant Z-score or |Z| < 3. Whiskers on each side of the plotted *D* values correspond to one standard error calculated using a Weighted Block Jackknife[5,7] and a block size of 5 Mb across all autosomes on the "2200k" Panel (nsnps (*Ust'Ishim*) = 1,951,462).

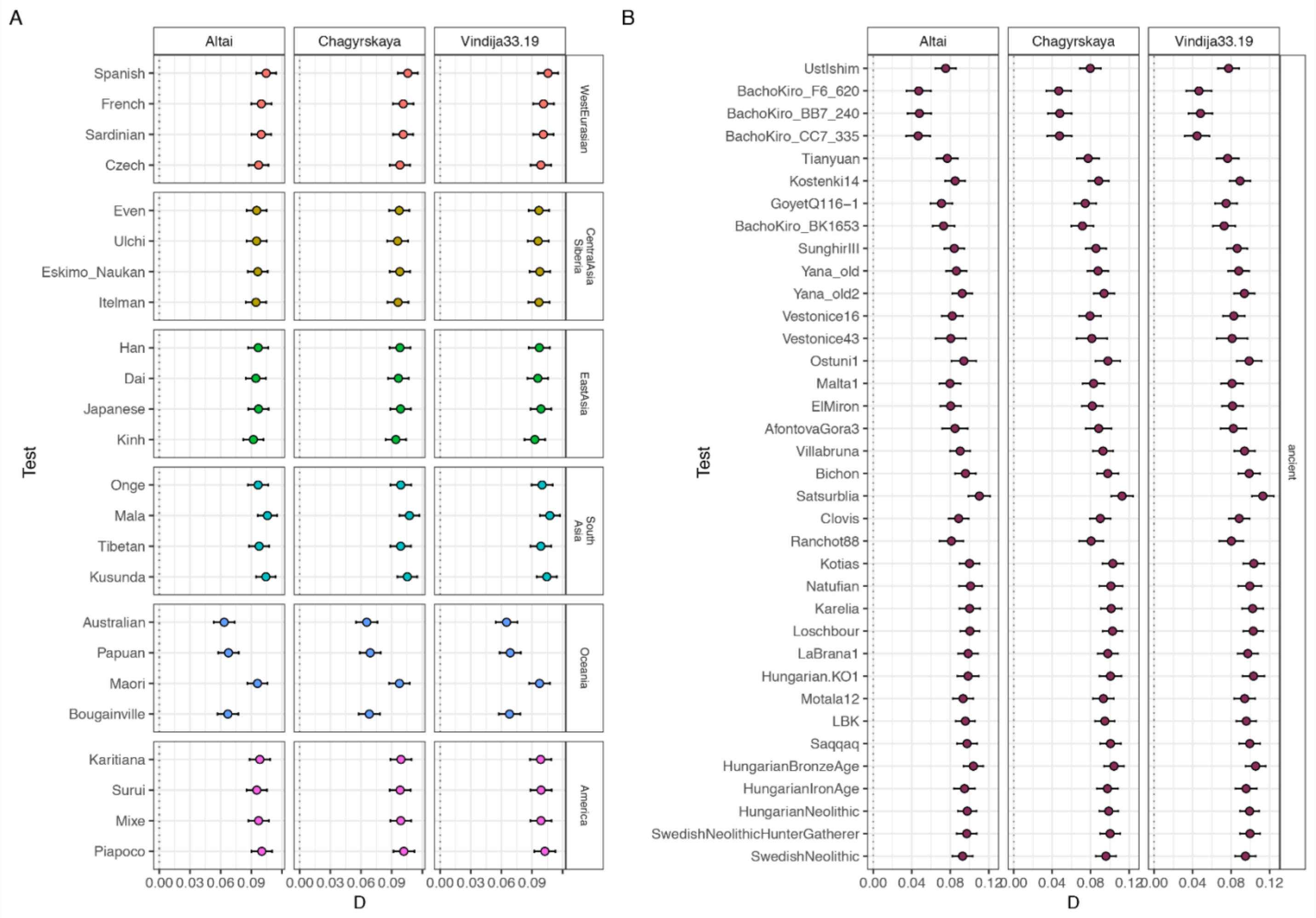


**Figure S7.5 D-statistics comparing derived allele sharing with an archaic genome between *Oase1* and a range of present-day and ancient modern humans.** Values for *D(Oase1, Test; Archaic, Mbuti)* denoted as circles are plotted on the *x*-axes where *Archaic* is a high-coverage Neandertal[2,3,11] genome and *Test* is either a present-day population from SGDP[8] (panel A) or an ancient individual (panel B). Three Mbuti individuals from SGDP[8] were used as outgroup. *D* statistics were calculated using ADMIXTOOLS[5] as implemented in *admixr*[6]. We used a merged dataset of *Oase1* from this and the previous study. Filled-in circles indicate a significant Z-score or |Z| ≥ 3, and open circles indicate a non-significant Z-score or |Z| < 3. Whiskers on each side of the plotted *D* values correspond to one standard error calculated using a Weighted Block Jackknife[5,7] and a block size of 5 Mb across all autosomes on the “2200k” Panel (nsnps (*Oase 1*) = 402,526).

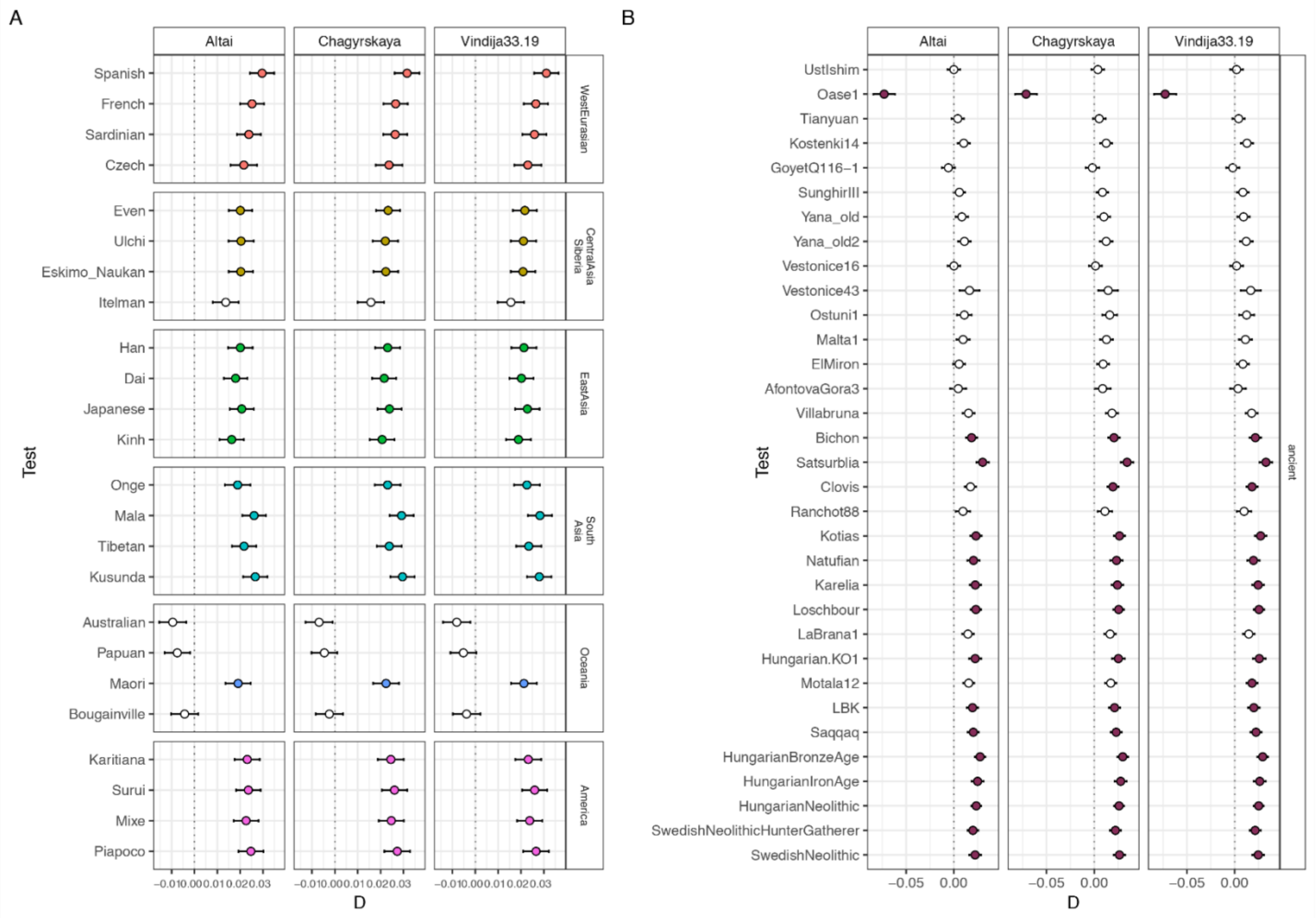


**Figure S7.6 D-statistics comparing derived allele sharing with an archaic genome between Bacho Kiro BK1653 and a range of present-day and ancient modern humans.** Values for *D(Bacho Kiro BK1653, Test; Archaic, Mbuti)* denoted as circles are plotted on the *x*-axes where *Archaic* is a high-coverage Neandertal[2,3,11] genome and *Test* is either a present-day population from SGDP[8] (panel A) or an ancient individual (panel B).Three Mbuti individuals from SGDP[8] were used as outgroup. *D* statistics were calculated using ADMIXTOOLS[5] as implemented in *admixr*[6]. Filled-in circles indicate a significant Z-score or |Z| ≥ 3, and open circles indicate a non-significant Z-score or |Z| < 3. Whiskers on each side of the plotted *D* values correspond to one standard error calculated using a Weighted Block Jackknife[5,7] and a block size of 5 Mb across all autosomes on the “2200k” Panel (nsnps (*Bacho Kiro BK1653*) = 825,379).

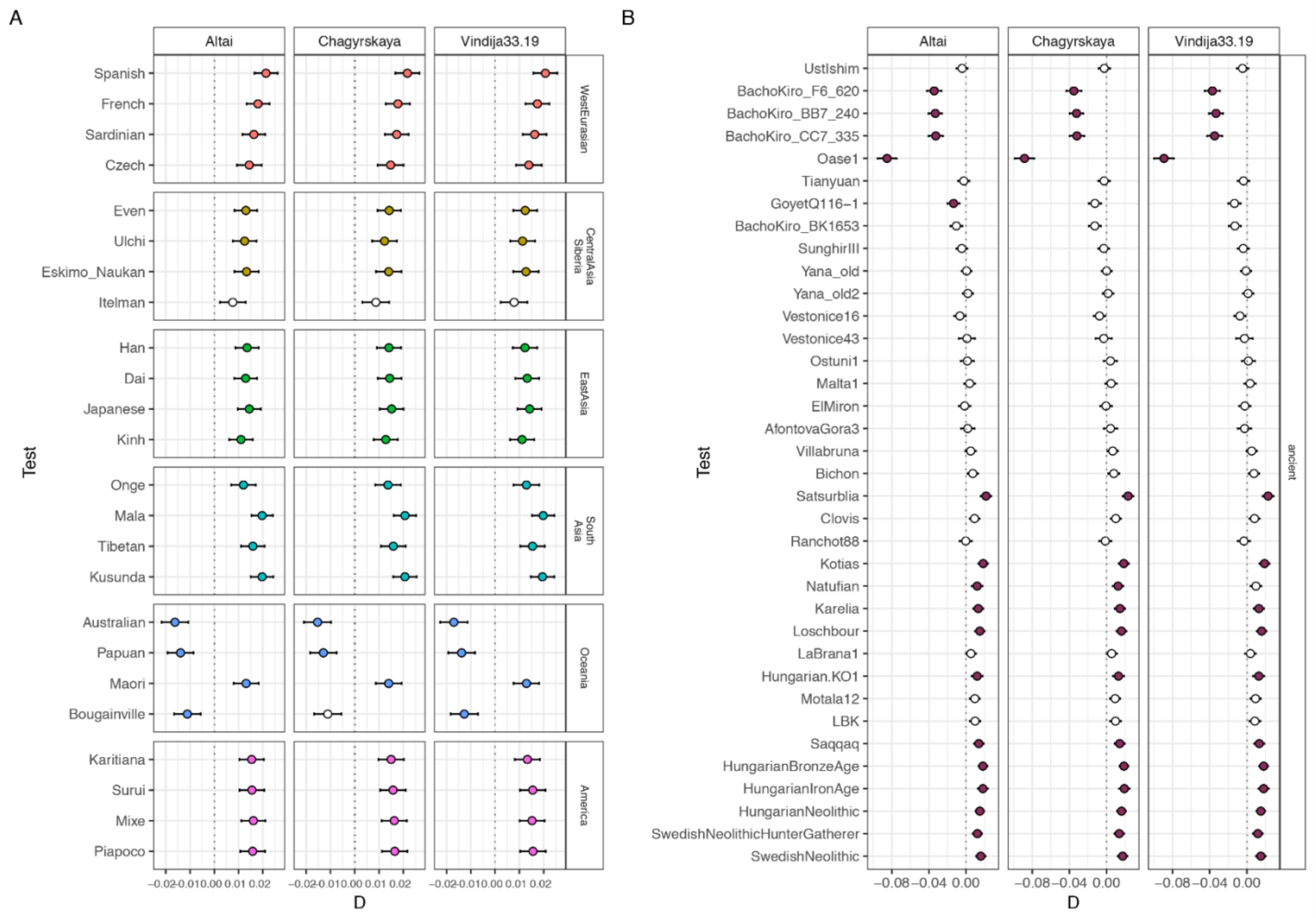


**Figure S7.7 D-statistics comparing derived allele sharing with an archaic genome between ~38,000-year-old *Kostenki14*[19] from Russia and a range of present-day and ancient modern humans.** Values for *D(Kostenki14, Test; Archaic, Mbuti)* denoted as circles are plotted on the *x*-axes where *Archaic* is a high-coverage Neandertal[2,3,11] genome and *Test* is either a present-day population from SGDP[8] (panel A) or an ancient individual (panel B). Three Mbuti individuals from SGDP[8] were used as outgroup. *D* statistics were calculated using ADMIXTOOLS[5] as implemented in *admixr*[6]. Filled-in circles indicate a significant Z-score or $|Z| \geq 3$, and open circles indicate a non-significant Z-score or $|Z| < 3$. Whiskers on each side of the plotted *D* values correspond to one standard error calculated using a Weighted Block Jackknife[5,7] and a block size of 5 Mb across all autosomes on the “2200k” Panel (nsnps (*Kostenki 14*) = 1,676,430).

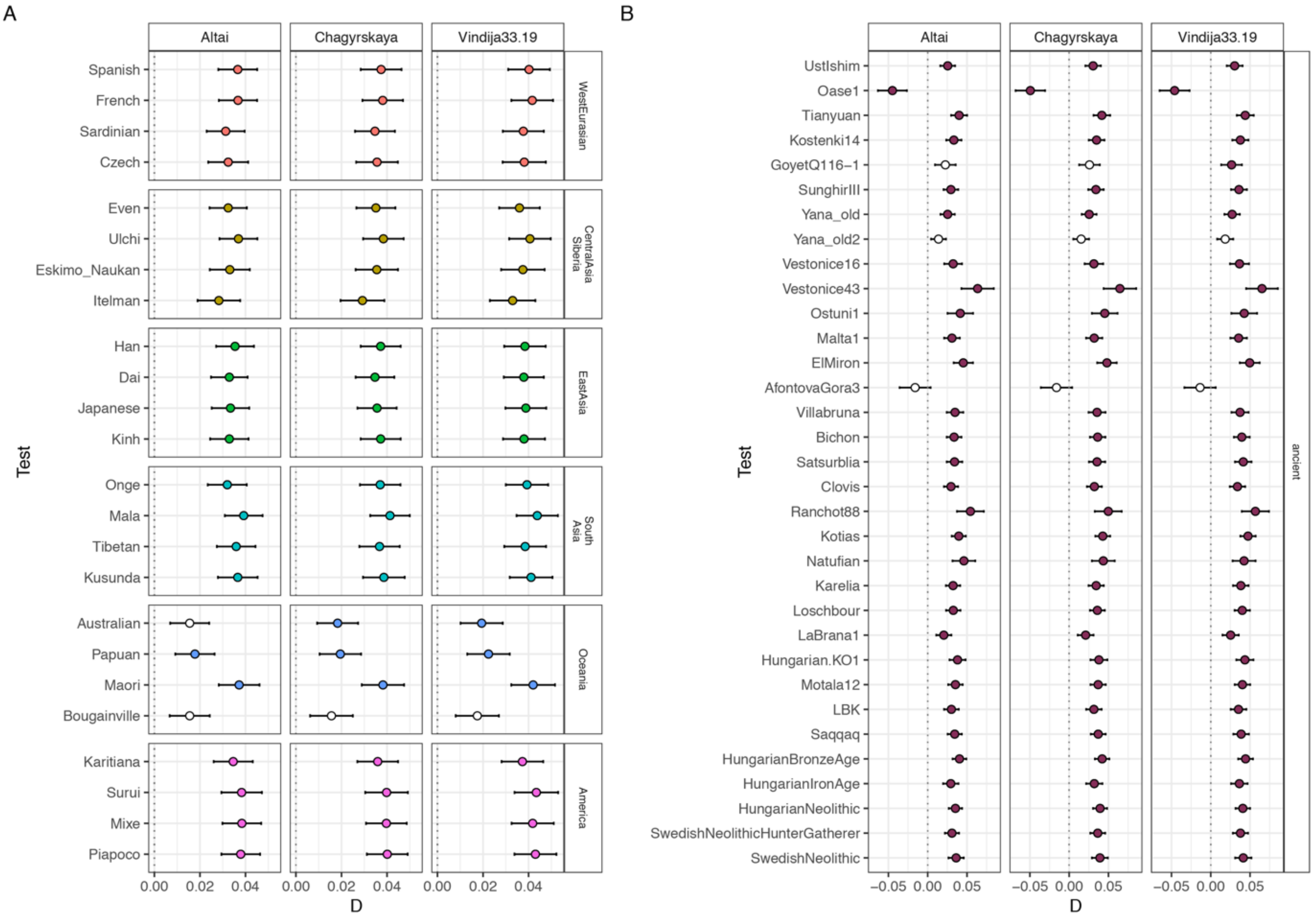


**Figure S7.8 D-statistics comparing derived allele sharing with an archaic genome between IUP Bacho Kiro F6-620 and a range of present-day and ancient modern humans.** Values for *D(Bacho Kiro F6-620, Test; Archaic, Chimpanzee)* denoted as circles are plotted on the *x*-axes where *Archaic* is a high-coverage Neandertal[2,3,11] genome and *Test* is either a present-day population from SGDP[8] (panel A) or an ancient individual (panel B). These analyses are restricted to transversion polymorphisms to mitigate the effect of ancient DNA damage. The genome of *panTro2* was used as an outgroup. *D* statistics were calculated using ADMIXTOOLS[5] as implemented in *admixr*[6]. Filled-in circles indicate a significant Z-score or $|Z| \geq 3$, and open circles indicate a non-significant Z-score or $|Z| < 3$. Whiskers on each side of the plotted *D* values correspond to one standard error calculated using a Weighted Block Jackknife[5,7] and a block size of 5 Mb across all autosomes on the "2200k" Panel (nsnps (*Bacho Kiro F6-620*) = 922,946).

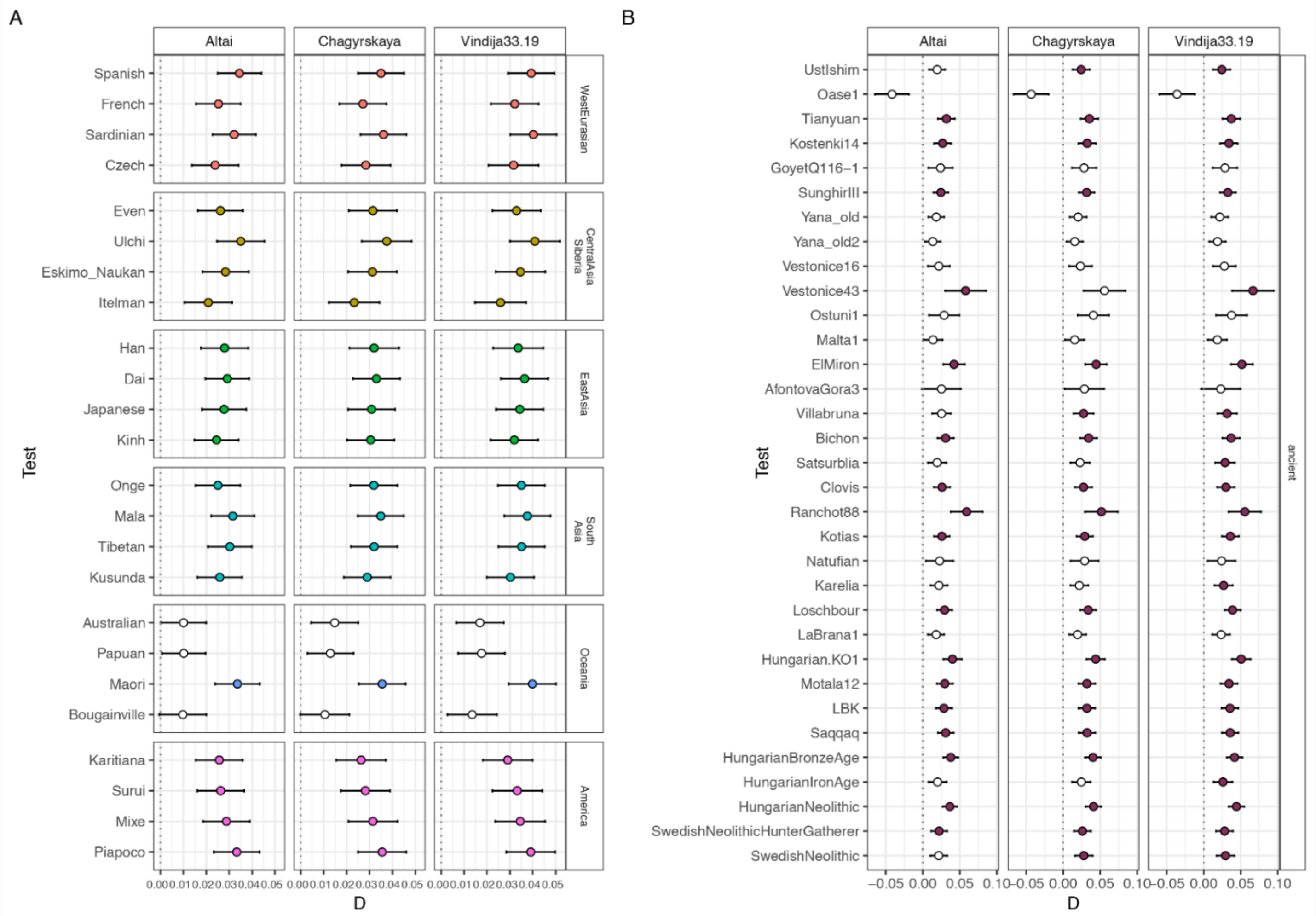


**Figure S7.9 D-statistics comparing derived allele sharing with an archaic genome between IUP Bacho Kiro CC7-335 and a range of present-day and ancient modern humans.** Values for *D(Bacho Kiro CC7-335, Test; Archaic, Chimpanzee)* denoted as circles are plotted on the *x*-axes where *Archaic* is a high-coverage Neandertal[2,3,11] genome and *Test* is either a present-day population from SGDP[8] (panel A) or an ancient individual (panel B). These analyses are restricted to transversion polymorphisms to mitigate the effect of ancient DNA damage. The genome of *panTro2* was used as an outgroup. *D* statistics were calculated using ADMIXTOOLS[5] as implemented in *admixr*[6]. Filled-in circles indicate a significant Z-score or |Z| ≥ 3, and open circles indicate a non-significant Z-score or |Z| < 3. Whiskers on each side of the plotted *D* values correspond to one standard error calculated using a Weighted Block Jackknife[5,7] and a block size of 5 Mb across all autosomes on the “2200k” Panel (nsnps (*Bacho Kiro CC7-335*) = 340,010).

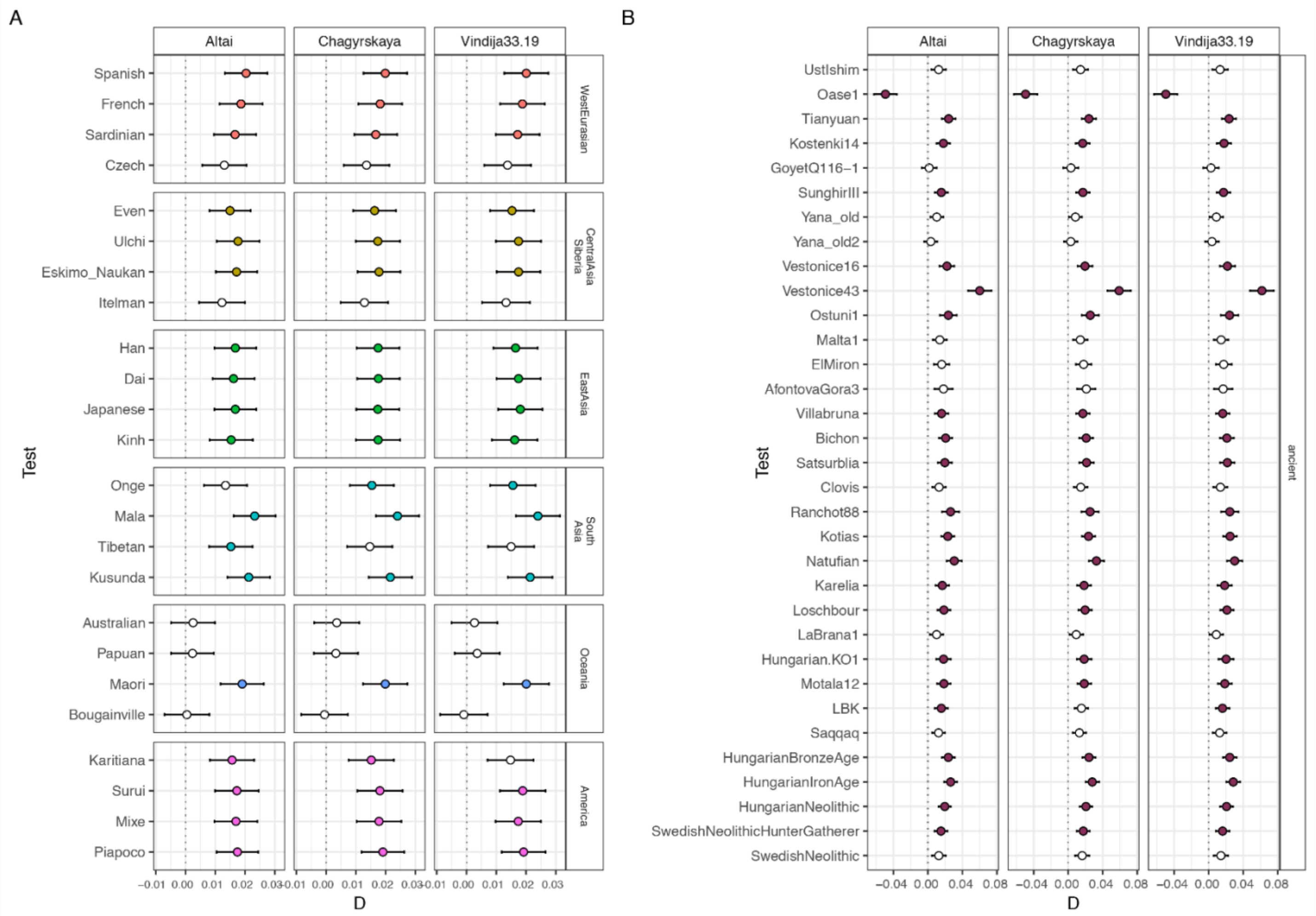


**Figure S7.10 D-statistics comparing derived allele sharing with an archaic genome between IUP Bacho Kiro BB7-240 and a range of present-day and ancient modern humans.** Values for *D(Bacho Kiro BB7-240, Test; Archaic, Chimpanzee)* denoted as circles are plotted on the *x*-axes where *Archaic* is a high-coverage Neandertal[2,3,11] genome and *Test* is either a present-day population from SGDP[8] (panel A) or an ancient individual (panel B). These analyses are restricted to transversion polymorphisms to mitigate the effect of ancient DNA damage. The genome of *panTro2* was used as an outgroup. *D* statistics were calculated using ADMIXTOOLS[5] as implemented in *admixr*[6]. Filled-in circles indicate a significant Z-score or |Z| ≥ 3, and open circles indicate a non-significant Z-score or |Z| < 3. Whiskers on each side of the plotted *D* values correspond to one standard error calculated using a Weighted Block Jackknife[5,7] and a block size of 5 Mb across all autosomes on the “2200k” Panel (nsnps (*Bacho Kiro BB7-240*) = 362,545).

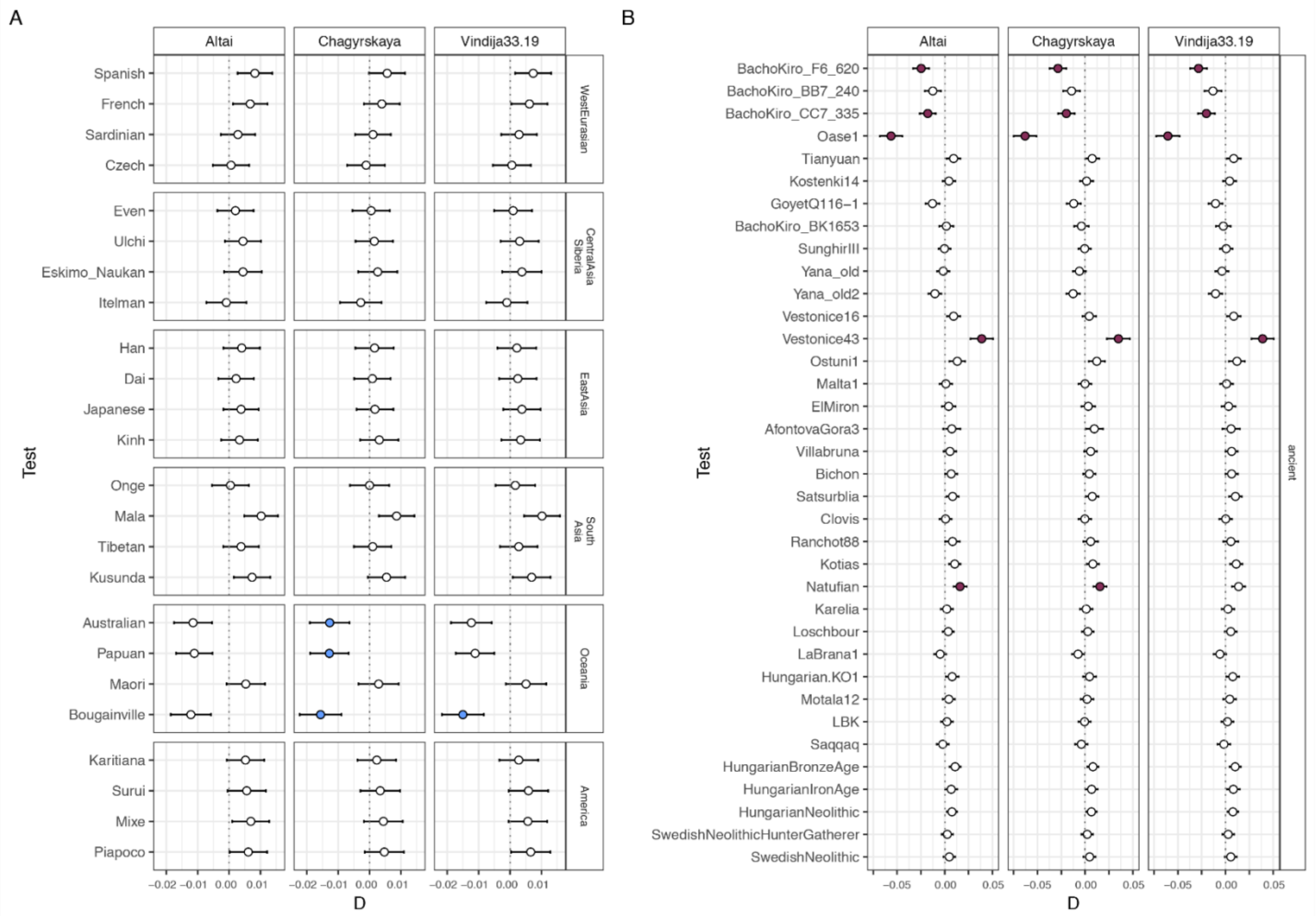


**Figure S7.11 D-statistics comparing derived allele sharing with an archaic genome between ~45,000-year-old *Ust'Ishim* from Siberia and a range of present-day and ancient modern humans.** Values for *D(Ust'Ishim, Test; Archaic, Chimpanzee)* denoted as circles are plotted on the *x*-axes where *Archaic* is a high-coverage Neandertal[2,3,11] genome and *Test* is either a present-day population from SGDP[8] (panel A) or an ancient individual (panel B). These analyses are restricted to transversion polymorphisms to mitigate the effect of ancient DNA damage. The genome of *panTro2* was used as an outgroup. *D* statistics were calculated using ADMIXTOOLS[5] as implemented in *admixr*[6]. Filled-in circles indicate a significant Z-score or $|Z| \geq 3$, and open circles indicate a non-significant Z-score or $|Z| < 3$. Whiskers on each side of the plotted *D* values correspond to one standard error calculated using a Weighted Block Jackknife[5,7] and a block size of 5 Mb across all autosomes on the "2200k" Panel (nsnps (*Ust'Ishim*) = 1,095,471).

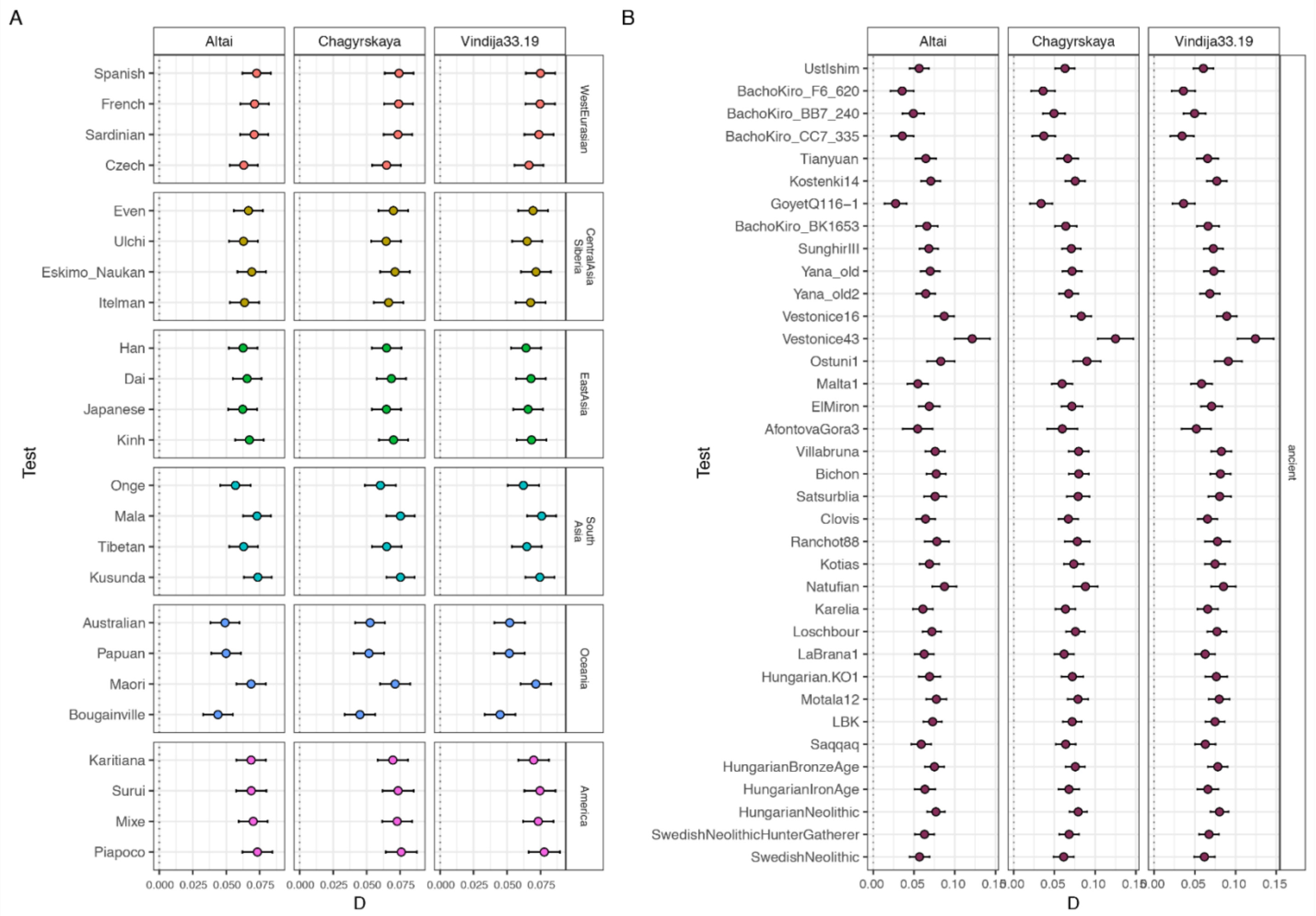


**Figure S7.12 D-statistics comparing derived allele sharing with an archaic genome between *Oase1* and a range of present-day and ancient modern humans.** Values for *D(Oase1, Test; Archaic, Chimpanzee)* denoted as circles are plotted on the *x*-axes where *Archaic* is a high-coverage Neandertal[2,3,11] genome and *Test* is either a present-day population from SGDP[8] (panel A) or an ancient individual (panel B). These analyses are restricted to transversion polymorphisms to mitigate the effect of ancient DNA damage. The genome of *panTro2* was used as an outgroup. *D* statistics were calculated using ADMIXTOOLS[5] as implemented in *admixr*[6]. Filled-in circles indicate a significant Z-score or |Z| ≥ 3, and open circles indicate a non-significant Z-score or |Z| < 3. Whiskers on each side of the plotted *D* values correspond to one standard error calculated using a Weighted Block Jackknife[5,7] and a block size of 5 Mb across all autosomes on the “2200k” Panel (nsnps (*Oase 1*) = 180,040).

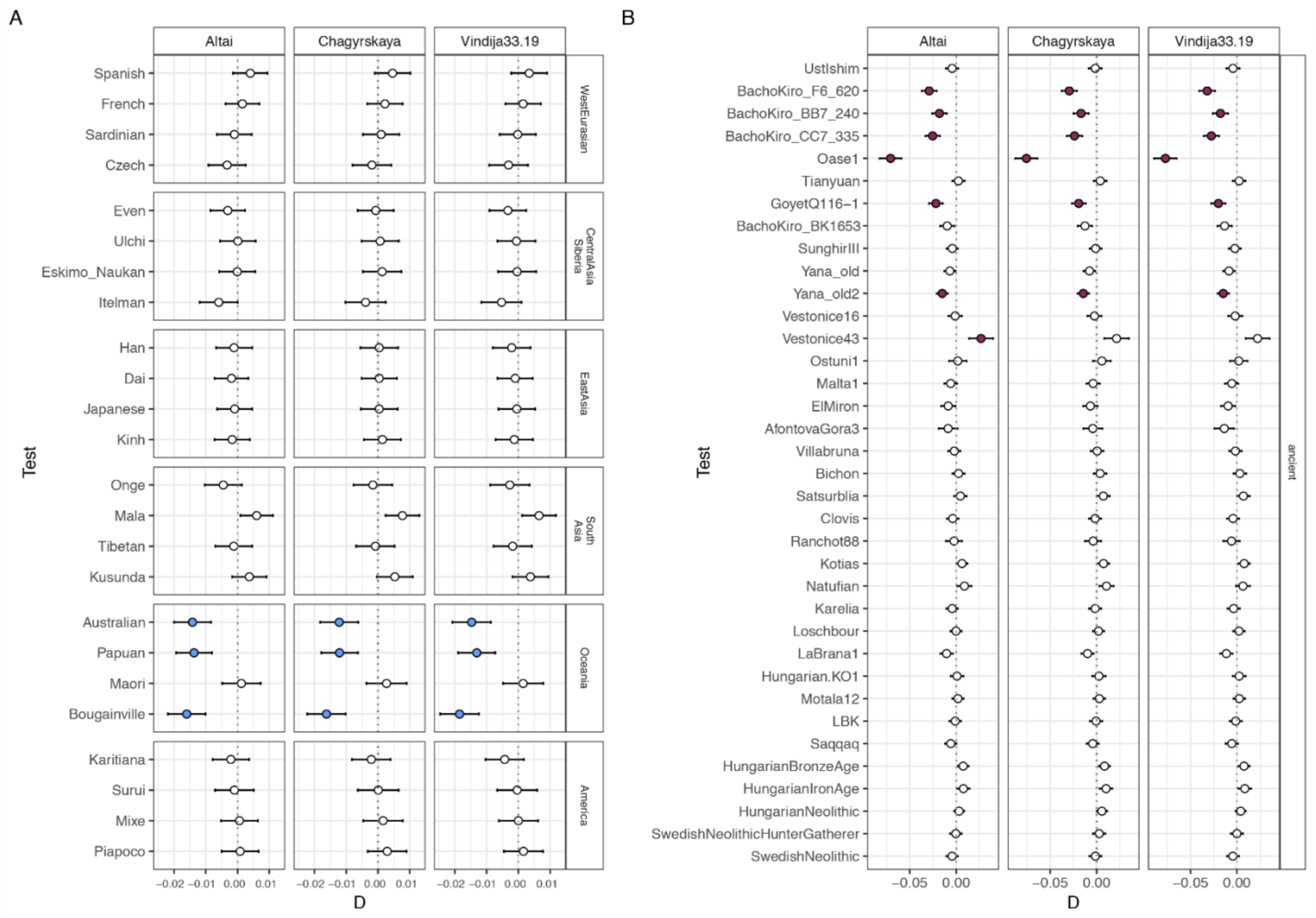


**Figure S7.13 D-statistics comparing derived allele sharing with an archaic genome between ~38,000-year-old *Kostenki14*[19] from Russia and a range of present-day and ancient modern humans.** Values for *D(Kostenki14, Test; Archaic, Chimpanzee)* denoted as circles are plotted on the *x*-axes where *Archaic* is a high-coverage Neandertal[2,3,11] genome and *Test* is either a present-day population from SGDP[8] (panel A) or an ancient individual (panel B). These analyses are restricted to transversion polymorphisms to mitigate the effect of ancient DNA damage. The genome of *panTro2* was used as an outgroup. *D* statistics were calculated using ADMIXTOOLS[5] as implemented in *admixr*[6]. Filled-in circles indicate a significant Z-score or |Z| ≥ 3, and open circles indicate a non-significant Z-score or |Z| < 3. Whiskers on each side of the plotted *D* values correspond to one standard error calculated using a Weighted Block Jackknife[5,7] and a block size of 5 Mb across all autosomes on the “2200k” Panel (nsnps (*Kostenki 14*) = 814,133).

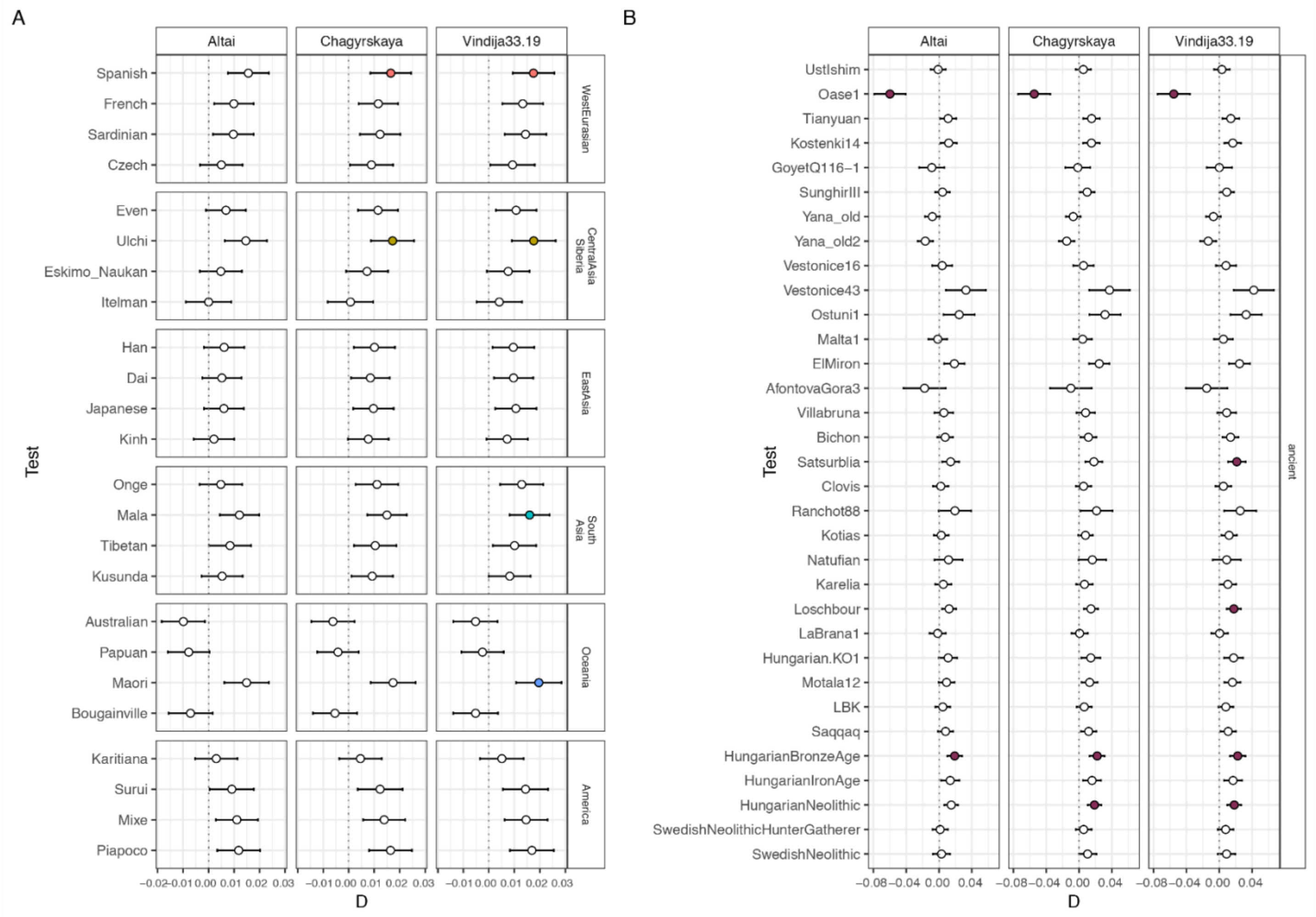


**Figure S7.14 D-statistics comparing derived allele sharing with an archaic genome between Bacho Kiro BK1653 and a range of present-day and ancient modern humans.** Values for *D(Bacho Kiro BK1653, Test; Archaic, Chimpanzee)* denoted as circles are plotted on the *x*-axes where *Archaic* is a high-coverage Neandertal[2,3,11] genome and *Test* is either a present-day population from SGDP[8] (panel A) or an ancient individual (panel B). These analyses are restricted to transversion polymorphisms to mitigate the effect of ancient DNA damage. The genome of *panTro2* was used as an outgroup. *D* statistics were calculated using ADMIXTOOLS[5] as implemented in *admixr*[6]. Filled-in circles indicate a significant Z-score or $|Z| \geq 3$, and open circles indicate a non-significant Z-score or $|Z| < 3$. Whiskers on each side of the plotted *D* values correspond to one standard error calculated using a Weighted Block Jackknife[5,7] and a block size of 5 Mb across all autosomes on the “2200k” Panel (nsnps (*Bacho Kiro BK1653*) = 382,191).

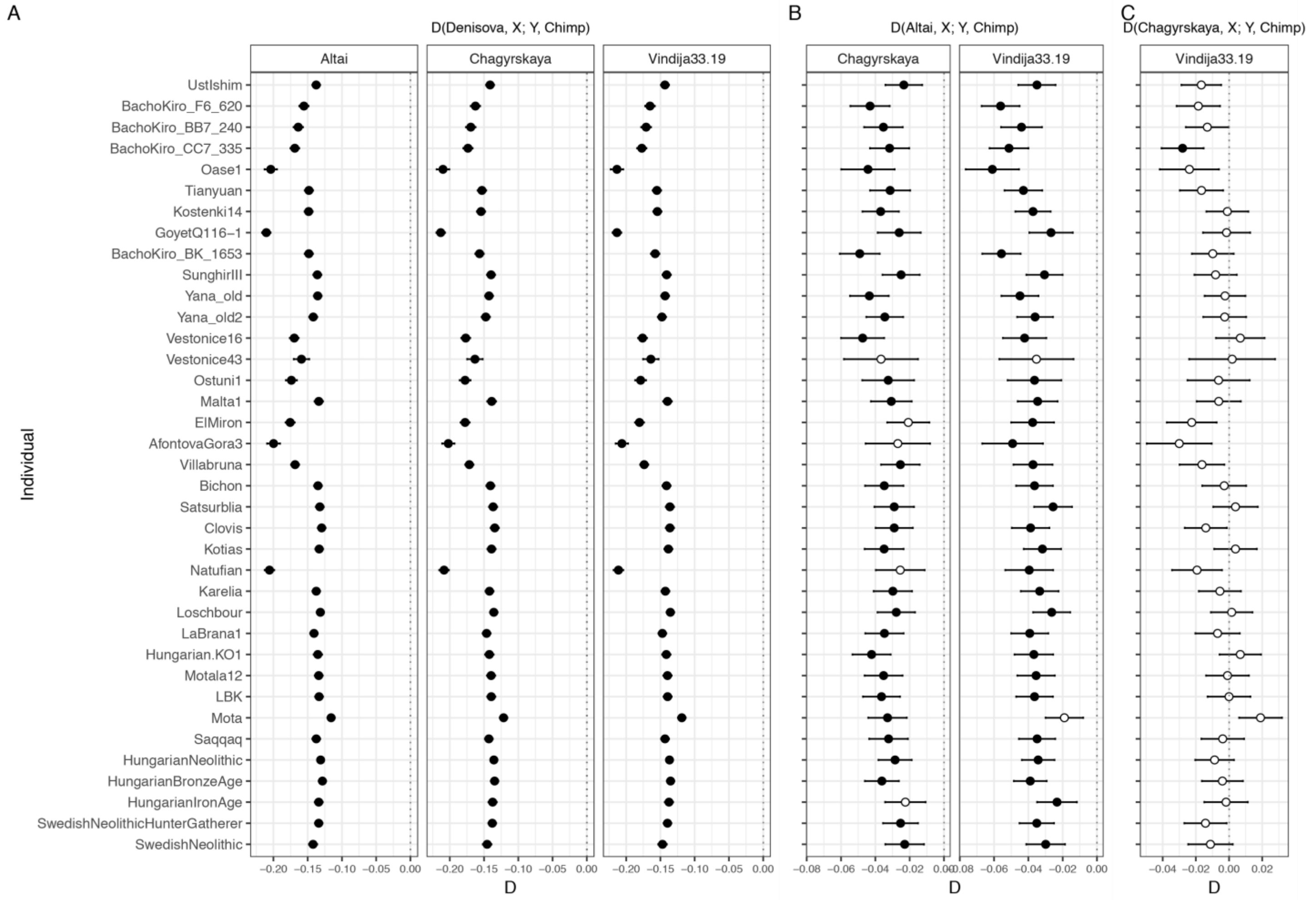


**Figure S7.15 Proximity to the introgressing archaic population in ancient humans calculated as *D($Archaic_1$, $Archaic_2$; ancient human, Chimp).*** A) All ancient modern humans are significantly closer to Neandertals[2,3,11] than to *Denisova3*, *D(Denisova3, Neandertal; ancient human, Chimp)*, B) introgressing Neandertals are significantly closer to *Chagyrskaya 8* and *Vindija 33.19* than to the *Altai* Neandertal in most ancient modern humans, *D(Altai Neandertal, $Neandertal_2$; ancient human, Chimp),* C) no significant difference in the introgressing Neandertal proximity to *Chagyrskaya 8* and *Vindija 33.19*, *D(Chagyrskaya 8, Vindija 33.19; ancient human, Chimp). D* values denoted as circles plotted on the *x*-axes were calculated using ADMIXTOOLS[5] as implemented in *admixr*[6]. The genome of *panTro2* was used as an outgroup. Filled-in circles indicate a significant Z-score or $|Z| \geq 3$, and open circles indicate a non-significant Z-score or $|Z| < 3$. Whiskers on each side of the plotted *D* values correspond to one standard error calculated using a Weighted Block Jackknife[5,7] and a block size of 5 Mb across all autosomes on the “2200k” Panel (nsnps = 2,056,573).

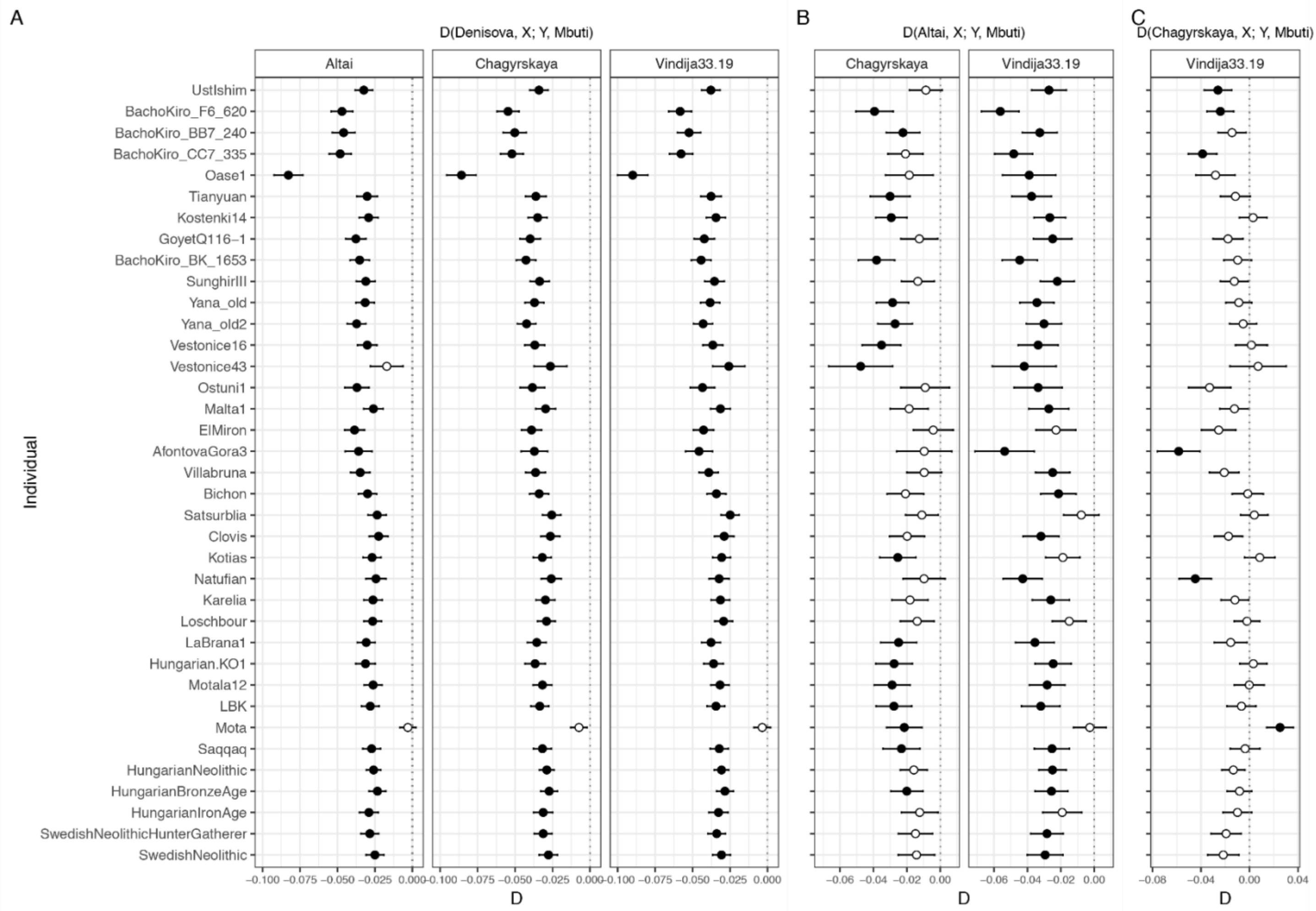


**Figure S7.16 Proximity to the introgressing archaic population in ancient humans calculated as *D(Archaic$_1$, Archaic$_2$; ancient human, Mbuti)*.** A) All ancient modern humans are significantly closer to Neandertals than to *Denisova3*[18], *D(Denisova3, Neandertal; ancient human, Mbuti)*, B) introgressing Neandertals are significantly closer to *Chagyrskaya 8*[11] and *Vindija 33.19*[3] than to the *Altai* Neandertal[2] in most ancient modern humans, *D(Altai Neandertal, Neandertal$_2$; ancient human, Mbuti)*, C) no significant difference in the introgressing Neandertal proximity to *Chagyrskaya 8*[11] and *Vindija 33.19*[3] for most ancient modern humans, *D(Chagyrskaya 8, Vindija 33.19; ancient human, Mbuti). D* values denoted as circles plotted on the *x*-axes were calculated using ADMIXTOOLS[5] as implemented in *admixr*[6]. Genomes of three Mbuti individuals from SGDP[8] are used as an outgroup. Filled-in circles indicate a significant Z-score or $|Z| \geq 3$, and open circles indicate a non-significant Z-score or $|Z| < 3$. Whiskers on each side of the plotted *D* values correspond to one standard error calculated using a Weighted Block Jackknife[5,7] and a block size of 5 Mb across all autosomes on the “2200k” Panel (nsnps = 2,056,573).

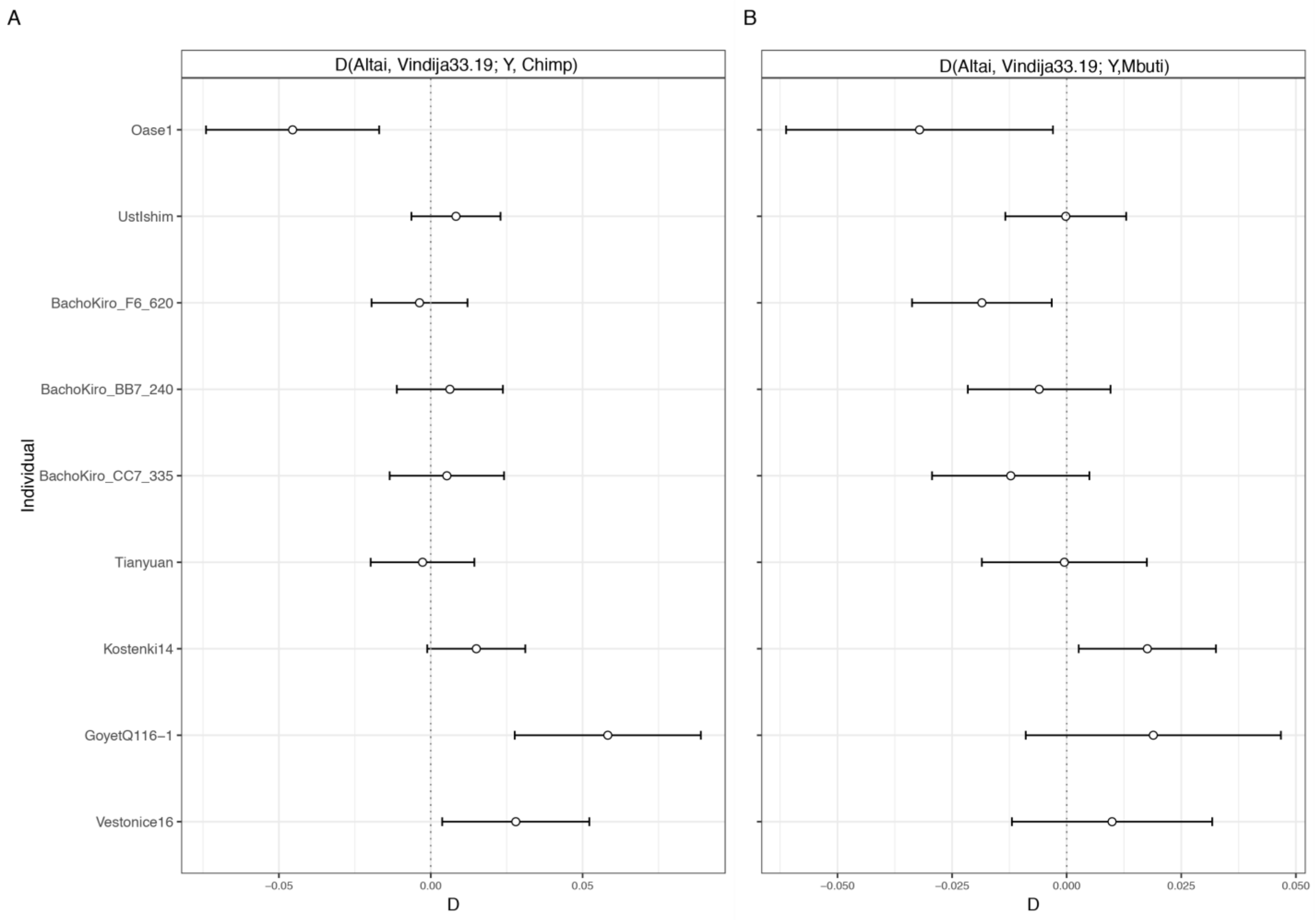


**Figure S7.17 The effect of down-sampling high-coverage genomes of the *Altai* Neandertal[2] and *Vindija 33.19*[3] on *D(Altai Neandertal, Vindija 33.19; ancient human, Outgroup)*.** Down-sampling high-coverage Neandertal genomes to the same coverage as in late Neandertals[17] (number of SNPs reported in the Tab. S7.1) on "2200k" Panel and restricting the analyses to transversion polymorphisms causes the statistics that are otherwise significant (see Fig. S7.15 and S7.16) to become not significantly different from 0. *D* values denoted as circles plotted on the *x*-axes were calculated using ADMIXTOOLS[5] as implemented in *admixr*[6]. Genomes of *panTro2* (panel A) and three Mbuti individuals from SGDP[8] (panel B) are used as outgroups. Open circles indicate a non-significant Z-score or $|Z| < 3$. Whiskers on each side of the plotted *D* values correspond to one standard error computed with a Weighted Block Jackknife[5,7] and a block size of 5 Mb across all autosomes on the "2200k" Panel.

# Supplementary Information 8

## Neandertal introgressed segments in Bacho Kiro individuals and dating of Neandertal admixture

### Distribution of Neandertal introgressed tracks in the genomes of Bacho Kiro and other ancient modern humans

We used *admixfrog*[1] (version 0.5.6, https://github.com/BenjaminPeter/admixfrog/) for detecting archaic introgressed segments in the genomes of Bacho Kiro Cave individuals, as well as in 32 ancient modern humans[2-12] and 254 present-day non-Africans from the Simons Genome Diversity Project (SGDP)[13] as a direct comparison. *Admixfrog* allows the reliable detection of introgressed segments in low-coverage ancient genomes even in the presence of substantial levels of present-day human DNA contamination[1]. In short, *admixfrog* models each individual (*Target*) as a mixture of at least two different *Sources*. As potential *Sources* we used three high-coverage Neandertal genomes[14-16] (abbreviated as NEA), the high-coverage *Denisova 3* genome[17] (abbreviated as DEN) and 44 genomes of present-day Sub-Saharan Africans from SGDP[13] (abbreviated as AFR). We used the genome of a chimpanzee (*panTro4*, abbreviated as PAN) to infer the ancestral state at each position in the "Archaic admixture" panel.

We used BAM files of 21 ancient modern humans[2-4], including six Bacho Kiro Cave specimens and *Oase 1*, that were enriched for ~1.7 million SNPs informative of Neandertal and Denisovan ancestry ("Archaic admixture"[3] panel, Supplementary Information 3). All BAM files were filtered as described in Supplementary Information 2. Briefly, all files were aligned to the human reference genome containing the decoy sequences (GRCh37/1000 Genomes release) and we used *samtools*[18] to filter for fragments equal or longer than 35 base pairs (bp) that had a mapping quality of 25 or greater (MQ≥25). We also used 16 ancient modern humans from different points in time and different geographical regions for which whole-genome shotgun data are available[5-12] as a comparison. Their respective BAM files were intersected with ~1.7 million SNPs of the "Archaic admixture" panel using *BEDTools*[19] (version: 2.24.0) after alignment to the GRCh37 and filtering for fragments of at least 35 bp with a mapping quality of at least 25. For *GoyetQ116-1*, we did not have "Archaic admixture" panel data and we therefore used "2200k" SNP Panel for inferring Neandertal introgressed segments in this individual.

For each ancient modern human (*Target*) we generated input files directly from the respective BAM files using the following command:

```
admixfrog-bam --bamfile {individual}.bam --out {individual}.in.xz --
ref {ref_archaicadmixture.csv.xz}
```

To further identify archaic introgressed segments in a genome of a given *Target*, we run:

```
admixfrog --infile {individual}.in.xz --ref
{ref_archaicadmixture.csv.xz} --out {out_individual} --states AFR NEA
DEN --cont-id AFR --ll-tol 0.01 --bin-size 5000 --est-F --est-tau
--freq-F 3 --freq-contamination 3 --e0 0.01 --ancestral PAN --run-
penalty 0.4 --max-iter 250 --n-post-replicates 200 --filter-pos 50 --
filter-map 0 –{female/male}
```

where --states AFR NEA DEN correspond to potential *Sources*, --cont-id AFR is a potential contaminant and --ancestral PAN indicates that *panTro4* is used to infer a putative ancestral allele. The window size (--bin-size) is set to 5,000 base pairs (bp) for all individuals except Bacho Kiro *CC7-2289*, where a window size is increased to 100,000 bp due to the sparse coverage of informative sites for this individual (a total of 19,703 SNPs covered by at least one fragment, see Supplementary Information 2, Tab. S2.6). The African American genetic map[20] (build hg19) was used for re-scaling physical distances (in base pairs) into genetic distances (in centimorgans [cM]). For all downstream analyses we only consider archaic fragments longer than 0.2 cM in ancient modern humans, as these are can be confidently inferred with high accuracy using *admixfrog*[1].

With the additional data produced in this study we identify a total of 496.56 cM of Neandertal segments in *Oase1*, with ten segments longer than five cM and three of them longer than 50 cM (Fig. 3 and Extended Data Fig. 8A). Interestingly, we also identify large stretches of Neandertal ancestry across autosomes of IUP Bacho Kiro *F6-620, AA7-738, CC7-335, BB7-240* and *CC7-2289* (Fig. 3, Extended Data Fig. 8A, Fig. S8.1), *i.e.* all Initial Upper Palaeolithic specimens from Bacho Kiro Cave. In total, we detect 279.59 cM of Neandertal introgressed segments *in F6-620*, 251.63 cM in *CC7-335* and 220.86 cM in *BB7-240*, with seven, six and nine of those segments longer than five cM (Tab. S8.1). The longest introgressed segment of Neandertal ancestry in *F6-620* is located on chromosome 5 (chr5:5,207,757-53,693,820) and has a recombination length of 54.26 cM (48.49 Mb). The inferred Neandertal introgressed

segment boundaries in *F6-620* and *AA7-738* are nearly identical (Fig. 3, Extended Data Fig. 8A, Fig. S8.1A), which is not expected even for the first-degree relatives, thus further supporting our conclusion based on the pairwise mismatch rate (see Supplementary Information 4) that these two specimens originate from the same individual.

The longest Neandertal segments that we identify are 25.61 cM on chromosome 19 (chr19:41,743,126-53,982,102) in *CC7-335* and 17.42 cM on chromosome 20 (chr20:39,121,854-49,228,176) in *BB7-240*. We also detect three Neandertal segments longer than 6 cM in Bacho Kiro *CC7-2289* on chromosomes 12 (chr12:71,475,600-80,845,047 and chr12:89,691,728-97,676,702) and chromosome 9 (chr9:110,903,519-115,770,224), albeit with lower confidence in the boundaries of the introgressed segments due to the limited amount of data for this individual (Fig. S8.1B). As a comparison, using *admixfrog* we identify 143.42 cM of Neandertal segments in a ~45,000-year-old *Ust'Ishim* from Siberia, with the longest segments of 6.12 cM and 5.92 cM on chromosomes 13 (chr13: 22,719,662-24,676,454) and 21 (chr21:31,552,179-35,448,101), respectively (Extended Data Fig. 7).

Neandertal ancestry is distributed in smaller segments across the genome of the ~34,000-year-old Bacho Kiro *BK1653*, with the longest segment of 2.49 cM on chromosome 6 (chr6:37,721,776- 40,410,185), an average segment length of 0.49 cM, and a total of 121.74 cM of Neandertal ancestry (Fig. 3). These numbers are similar to other Upper Palaeolithic modern humans, such as the ~38,000-year-old *Kostenki14* from Russia with an average segment length of 0.59 cM and a total of 118.62 cM of Neandertal introgressed segments, and the ~35,000-year-old *Goyet Q116-1* from Belgium with an average segment length of 0.51 cM and a total of 134.18 cM of Neandertal introgressed segments. Fig. 3 and the Extended Data Fig. 7 show the physical distribution of the inferred Neandertal introgressed segments across all autosomes in IUP Bacho Kiro individuals, *Ust'Ishim*, *Oase1*, *Tianyuan*, *Kostenki 14*, *Goyet Q116-1* and Bacho Kiro *BK1653*.

**Neandertal introgressed segments on the X chromosome**

Since the "Archaic admixture" panel does not contain SNPs of the sex chromosomes, we investigated 55,248 SNPs on the X chromosome from the "2200k" Panel for inferring the distribution of Neandertal introgressed segments on the X chromosome in Bacho Kiro Cave individuals, *Oase1*, and other ancient modern humans for which we have both panels available. We created the input files for admixfrog directly from BAM files as detailed above, with the difference of using `--ref ref_A3700k.csv.xz`.

Interestingly, we identify two long segments of Neandertal ancestry on the X chromosome of *Oase 1*, with the recombination length of 4.00 cM (chrX:12,535,839-13,850,789) and 7.37 cM (chrX:30,969,935-32,771,678) (Extended Data Fig. 8A). We also detect one Neandertal segment of ~2 cM on each of the X chromosomes of *F6-620, CC7-335* and *BB7-240* (Extended Data Fig. 8A).

**Dating of the Neandertal admixture**

In order to estimate the timing of the most recent Neandertal introgression in the Bacho Kiro individuals, we applied three different methods to the Neandertal fragments identified with *admixfrog*[1]. For all methods we calculate the genetic length of the tracts using the African-American recombination map[20] and we compare the estimates with those obtained for *Oase1*[2].

First, we calculated the likelihood of the observed Neandertal tract lengths under the assumption of an exponential decay of fragment length due to recombination, with rate of decay *recombination rate* x *number of generations since introgression*. In order to isolate fragments of recent origin and specific to Bacho Kiro Cave individuals from those introgressed in modern humans and common to all out-of-Africa human populations, we considered only fragments longer than 10cM, for which the probability of having introgressed more recently than 50 generations prior is 96% assuming an exponential decay. Uncertainty, represented in Extended Data Fig. 8B as 96% confidence intervals, is estimated assuming an asymptotic gaussian approximation of the likelihood function. Our point estimates indicate that most Bacho Kiro Cave individuals had Neandertal ancestors ~7 generations before they lived. Only the *F6-620* shows a lower point estimate, 5 generations, however the confidence intervals overlap. The point estimate for *Oase1* is 4 generations, with uncertainty between 4 and 10 generations, comparably to what was estimated previously[2]. Note that three tracts identified previously in *Oase 1*[2] are estimated by *admixfrog* to be composed of different fragments, possibly explaining the slightly higher point estimate. Despite this, our estimates of timing of introgressions are comparable to those of Fu *et al.*[2].

We then modelled the lengths of the fragments as a mixture of two exponential distributions. Specifically, we co-estimated the number of generations since a more recent and an older introgression, as well as the proportion *p* of the fragments > 0.1cM originating from the more recent introgression event. For all genomes we performed a likelihood ratio test (degrees of freedom=2) comparing the likelihood of a simple exponential model with only one introgression event, and the model with two events. In Figure S8.2 we show the proportion of fragments originating from the different introgression events for different genetic lengths. We

find support for two introgression events for both *Oase 1* and several Bacho Kiro Cave specimens (S8.5). Estimates for models with one or two introgression events are reported in Tab. S8.1.

We then fitted the complementary cumulative distribution (CCD) of the lengths of Neandertal tracts , i.e. the proportion of tracts with length equal or higher than a given length X, as in Fu *et al.*[2]. Briefly, because of the exponential relationship between timing of introgression and length of the introgressed tracts, the logarithm of the cumulative distribution of tract length is expected to the depend linearly on the tract length, i.e. ln(CCD) ~ -( n – 1) x tract_length. We thus fitted the slope of this relationship separating introgressed tracts in older (≤ 5cM) and more recent (>5cM). The threshold of 5cM (96% of a tract to be introgressed <100 generations earlier), that is lower than 10cM used for the previous method, is used here to obtain enough data points to estimate accurately the slope. Fig. S8.3 shows both the slopes for older and more recent fragments, while the estimated values and uncertainties are shown in Tab. S8.2. For all genomes, except for the *F6-620* and *Oase 1*, the point estimates appear as slightly older, although the confidence intervals overlap. Therefore, consistent with our maximum likelihood estimates we infer that the *F6-620*, like *Oase 1*, had a Neandertal ancestor less than 6 generations before he lived.

Finally, we co-estimated the number of generations since introgression and the proportion of Neandertal admixture into the ancestors of the Bacho Kiro Cave individuals via simulations, using a similar approach used in Fu *et al.*[2]. Specifically, we computed full genome simulations of introgressing events contributing on average to 0.01-12.5% of the genome, and occurring 3 to 250 generations before the Bacho Kiro Cave individuals lived, in addition to an earlier introgression occurring 350 generations earlier (~10,000 years assuming a generation time of 29 years). For each of 10,000 simulations for each parameter combination, we calculated the mean of the longest 2, 5 or 10 Neandertal tracts, obtaining an expected distribution of average length of the longest Neandertal tracts. Then, for each sample, we compared the observed values with this distribution. In Fig. S8.5 we show combinations of parameters for which the observed means fall between the 5% and the 95% of the simulations (coloured squares). Combinations of parameters for which the observed values fall out of this expected distribution are marked in grey. Proportions of admixtures corresponding to the introgression of a single Neandertal ancestor are marked as black dots for each number of generations. We can see that while estimates are consistent with the other two methods assuming a single recent Neandertal ancestors (5-10 generations for all individuals), a higher proportion of Neandertal admixture – hence more recent Neandertal ancestors than a single one

– would better explain the average length of the longest fragments for a number of generations higher than ~8, for all Bacho-Kiro genomes (Fig.S8.5 and S8.6).

**References SI8:**


1 Peter, B. M. 100,000 years of gene flow between Neandertals and Denisovans in the Altai mountains. *bioRxiv* (2020).

2 Fu, Q. *et al.* An early modern human from Romania with a recent Neanderthal ancestor. *Nature* **524**, 216-219, doi:10.1038/nature14558 (2015).

3 Fu, Q. *et al.* The genetic history of Ice Age Europe. *Nature* **534**, 200-205, doi:10.1038/nature17993 (2016).

4 Yang, M. A. *et al.* 40,000-Year-Old Individual from Asia Provides Insight into Early Population Structure in Eurasia. *Curr. Biol.* **27**, 3202-3208.e9, doi: 10.1016/j.cub.2017.09.030 (2017).

5 Fu, Q. *et al.* Genome sequence of a 45,000-year-old modern human from western Siberia. *Nature* **514**, 445-449, doi:10.1038/nature13810 (2014).

6 Sikora, M. *et al.* Ancient genomes show social and reproductive behavior of early Upper Paleolithic foragers. *Science* **358**, 659-662, doi: 10.1126/science.aao1807 (2017).

7 Sikora, M. *et al.* The population history of northeastern Siberia since the Pleistocene. *Nature* **570**, 182-188, doi: 10.1038/s41586-019-1279-z (2019).

8 Raghavan, M. *et al.* Upper Palaeolithic Siberian genome reveals dual ancestry of Native Americans. *Nature* **505**, 87-91, doi:10.1038/nature12736 (2014).

9 Lazaridis, I. *et al.* Ancient human genomes suggest three ancestral populations for present-day Europeans. *Nature* **513**, 409-413, doi:10.1038/nature13673 (2014).

10 Olalde, I. *et al.* Derived immune and ancestral pigmentation alleles in a 7,000-year-old Mesolithic European. *Nature* **507**, 225-228, doi:10.1038/nature12960 (2014).

11 Gallego Llorente, M. *et al.* Ancient Ethiopian genome reveals extensive Eurasian admixture throughout the African continent. *Science* **350**, 820-822, doi:10.1126/science.aad2879 (2015).

12 Rasmussen, M. *et al.* The genome of a Late Pleistocene human from a Clovis burial site in western Montana. *Nature* **506**, 225-229, doi:10.1038/nature13025 (2014).

13 Mallick, S. *et al.* The Simons Genome Diversity Project: 300 genomes from 142 diverse populations. *Nature* **538**, 201-206, doi:10.1038/nature18964 (2016).

14 Prüfer, K. *et al.* The complete genome sequence of a Neanderthal from the Altai Mountains. *Nature* **505**, 43-49, doi:10.1038/nature12886 (2014).

15 Prüfer, K. *et al.* A high-coverage Neandertal genome from Vindija Cave in Croatia. *Science* **358**, 655-658, doi: 10.1126/science.aao1887 (2017).

16 Mafessoni, F. *et al.* A high-coverage Neandertal genome from Chagyrskaya Cave. *Proc. Natl. Acad. Sci. U.S.A.* **117**, 15132-15136, doi:10.1073/pnas.2004944117 (2020).

17 Meyer, M. *et al.* A high-coverage genome sequence from an archaic Denisovan individual. *Science* **338**, 222-226, doi:10.1126/science.1224344 (2012).

18 Li, H. *et al.* The Sequence Alignment/Map format and SAMtools. *Bioinformatics* **25**, 2078-2079, doi:10.1093/bioinformatics/btp352 (2009).

19 Quinlan, A. R. & Hall, I. M. BEDTools: a flexible suite of utilities for comparing genomic features. *Bioinformatics* **26**, 841-842 (2010).

20 Hinch, A. G. *et al.* The landscape of recombination in African Americans. *Nature* **476**, 170-175, doi: 10.1038/nature10336 (2011).

**Table S8.1 Estimates of the number of generations before the ancient (*n.anc*) and more recent (*n.rec*) Neandertal introgression in Bacho Kiro Cave individuals and other ancient modern humans, and the proportion of Neandertal fragments originating from the most recent introgression event (*p*).** p-values obtained with a likelihood ratio tests comparing an exponential model with only one introgression occurred *n.gen1* generations before the birth of the individual and that with two events are reported as *LR*, and as LR Bonferroni after a multiple testing Bonferroni correction for the number of tested specimens (n=22). Specimens for which a model with two admixture events is supported (LR Bonferroni<0.05) are highlited in gray and an asterisk. Two-tailed 95% asymptotic confidence intervals (+/-CI) are calculated from the Hessian of the likelihood profile. Confidence intervals for the more recent introgression event are not calculated when this is not supported (n.a.).

| Specimen | n.rec | -CI | +CI | n.anc | -CI | +CI | p | -CI | +CI | n.gen 1 | LR | LR Bonferroni |
|---|---|---|---|---|---|---|---|---|---|---|---|---|
| **BK AA7_738** | 5.1 | 0.7 | 9.5 | 47.5 | 38.8 | 56.2 | 0.04 | 0.00 | 0.09 | 34.63 | 9.83E-09* | 2.16E-07* |
| **BK BB7_240** | 13.0 | 5.0 | 21.1 | 76.5 | 66.9 | 86.2 | 0.05 | 0.01 | 0.08 | 62.73 | 5.20E-07* | 1.14E-05* |
| **BK 1653** | 9.9 | n.a. | n.a. | 201.4 | 185.9 | 216.8 | 0.00 | 0.00 | 0.07 | 201.36 | 1 | 1 |
| **BK CC7_2289** | 12.0 | 5.6 | 18.5 | 393.6 | 286.2 | 500.9 | 0.17 | 0.09 | 0.26 | 60.44 | 0* | 0* |
| **BK CC7_335** | 11.5 | 6.9 | 16.1 | 91.3 | 79.0 | 103.6 | 0.10 | 0.06 | 0.15 | 53.58 | 0* | 0* |
| **BK F6-620** | 6.3 | 2.3 | 10.4 | 82.0 | 72.5 | 91.5 | 0.04 | 0.01 | 0.06 | 57.49 | 0* | 0* |
| **GoyetQ116-1** | 6.5 | n.a. | n.a. | 235.7 | 207.3 | 264.1 | 0.00 | 0.00 | 0.12 | 235.67 | 1 | 1 |
| **Kostenki 14** | 7.1 | n.a. | n.a. | 197.4 | 172.7 | 222.1 | 0.00 | 0.00 | 0.02 | 197.43 | 1 | 1 |
| **LBK** | 5.1 | n.a. | n.a. | 428.8 | 367.8 | 489.8 | 0.00 | 0.00 | 0.12 | 428.79 | 1 | 1 |
| **Loschbour** | 5.2 | n.a. | n.a. | 385.7 | 337.9 | 433.5 | 0.00 | 0.00 | 0.06 | 385.68 | 1 | 1 |
| **Malta 1** | 5.2 | n.a. | n.a. | 347.0 | 301.1 | 392.8 | 0.00 | 0.00 | 0.14 | 346.96 | 1 | 1 |
| **Oase1** | 4.0 | 1.4 | 6.7 | 82.8 | 69.8 | 95.7 | 0.06 | 0.02 | 0.09 | 39.78 | 0* | 0* |
| **Sunghir III** | 6.3 | n.a. | n.a. | 248.0 | 218.7 | 277.2 | 0.00 | 0.00 | 0.12 | 247.99 | 1 | 1 |
| **Sunghir II** | 6.0 | n.a. | n.a. | 248.9 | 218.0 | 279.7 | 0.00 | 0.00 | 0.13 | 248.86 | 1 | 1 |
| **Sunghir I** | 5.8 | n.a. | n.a. | 247.0 | 211.8 | 282.1 | 0.00 | 0.00 | 0.12 | 246.96 | 1 | 1 |
| **Sunghir IV** | 6.3 | n.a. | n.a. | 242.0 | 213.3 | 270.7 | 0.00 | 0.00 | 0.09 | 241.98 | 1 | 1 |
| **Tianyuan** | 6.3 | n.a. | n.a. | 215.2 | 186.2 | 244.2 | 0.00 | 0.00 | 0.14 | 215.19 | 1 | 1 |
| **Ust'Ishim** | 10.0 | n.a. | n.a. | 104.3 | 87.7 | 120.9 | 0.00 | 0.00 | 0.04 | 104.29 | 1 | 1 |

| | | | | | | | | | | | | |
|---|---|---|---|---|---|---|---|---|---|---|---|---|
| **Vestonice 16** | 5.4 | n.a. | n.a. | 301.1 | 264.1 | 338.1 | 0.00 | 0.00 | 0.13 | 301.08 | 1 | 1 |
| **Yana_old** | 5.9 | n.a. | n.a. | 261.0 | 229.9 | 292.1 | 0.00 | 0.00 | 0.09 | 260.99 | 1 | 1 |
| **Yana_old2** | 43.5 | 0.0 | 90.2 | 255.4 | 223.5 | 287.4 | 0.02 | 0.00 | 0.04 | 236.74 | 8.31E-03* | 1.83E-01 |

1936

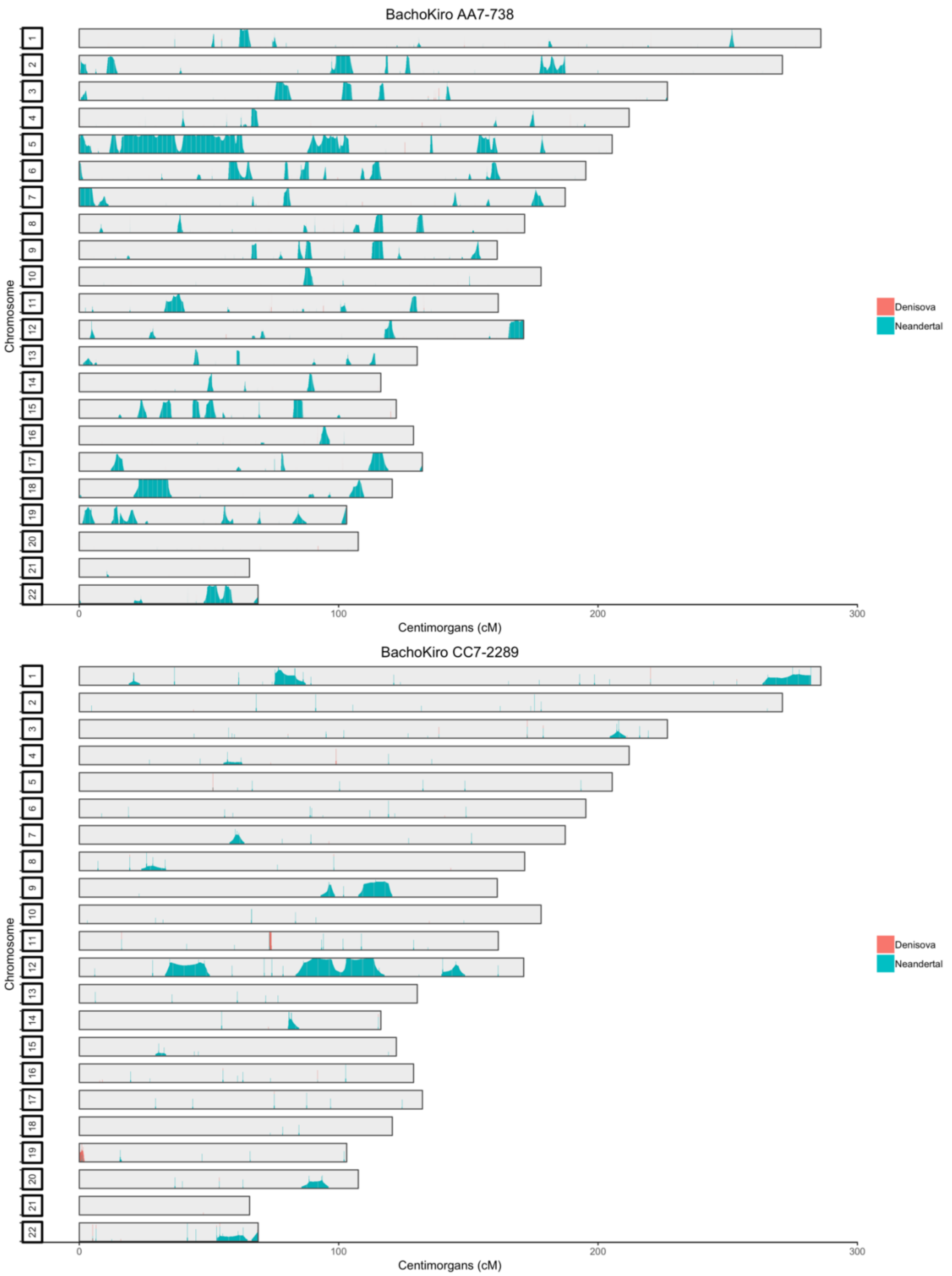


**Figure S8.1 Neandertal introgressed segments in the genomes of Bacho Kiro *AA7-738* and Bacho Kiro *CC7-2289* as inferred by admixfrog.** Neandertal segments are highlighted in blue and Denisovan segments are highlighted in orange. The height of the segment on each autosome is denoted on the y-axis and corresponds to the probability of the inferred segment.

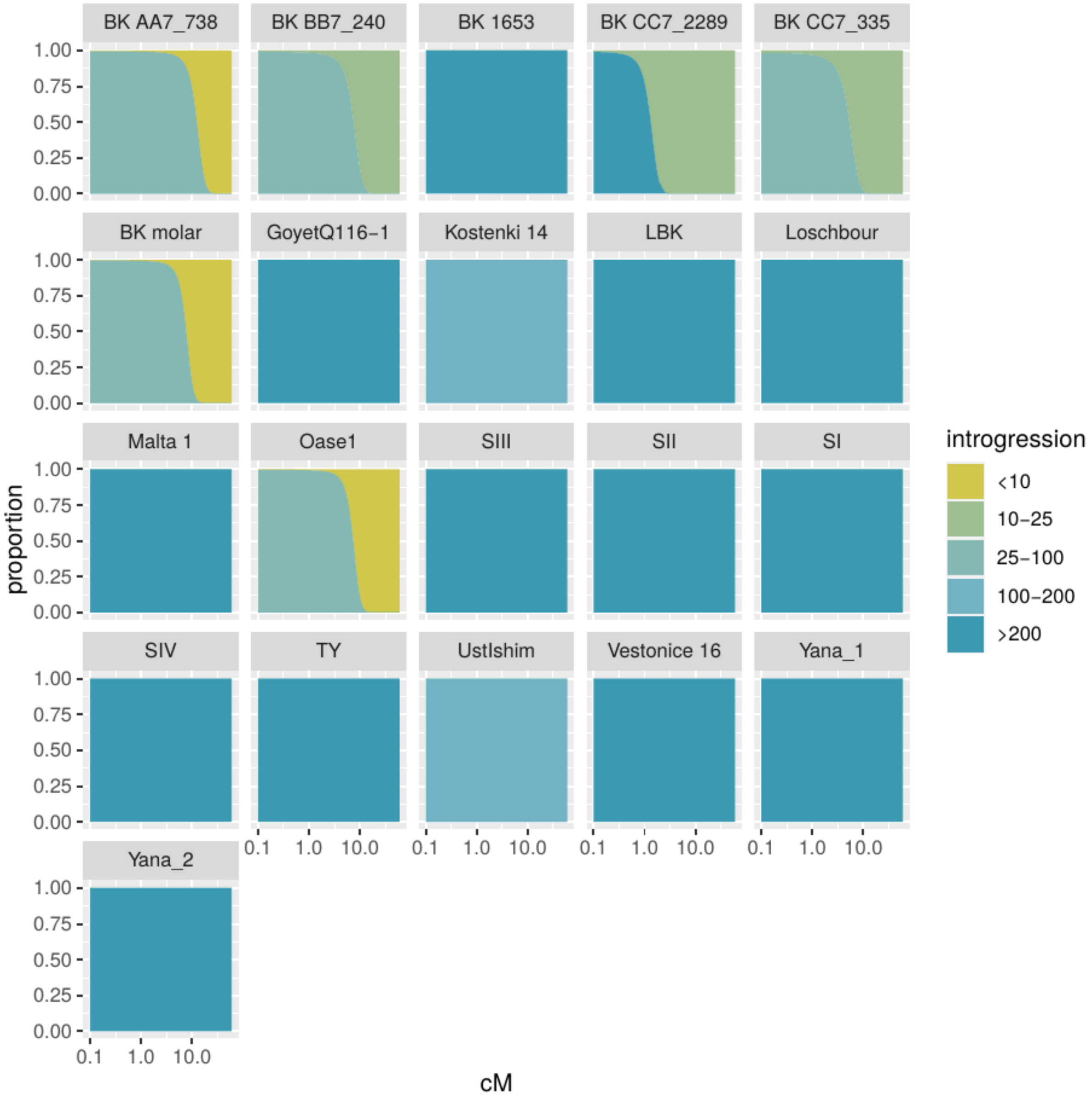


**Figure S8.2 Proportion of fragments (y-axis) originating from different introgression events (number of generation indicated by the colors in the legend).** Models with two different introgression events were plotted if supported by a likelihood ratio test <0.001 over models with only one introgression event (Table S8.1).

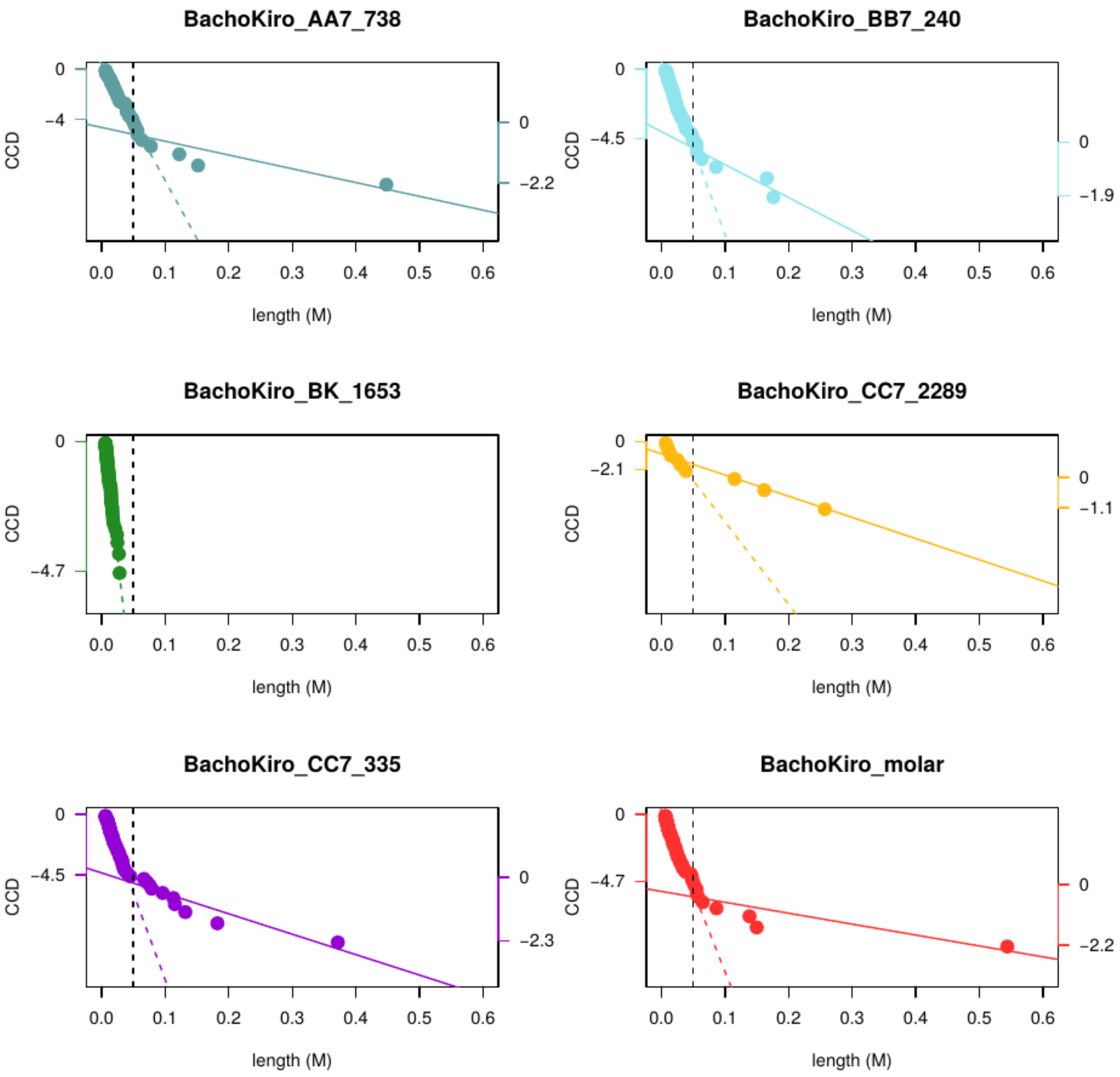


**Figure S8.3 Graphical representation of the relationship between CCD of tracts and tract lengths.** Slopes corresponding to the estimates of the number of generations before the most recent Neandertal introgression in Bacho Kiro (tracts >5cM) and older introgression events, here modelled as a single pulse (tracts $\leq$ 5cM), are shown as continuous and dashed colored lines, respectively. In order to visualize both slopes in the same plot, the CCD of all fragments with length >0.5cM is used as y-axis. The corresponding CCD for tracts lower and higher than 5cM are shown as coloured axes on the left and right of the plots, respectively. The 5cM threshold is indicated by a vertical dashed line. The x-axis represent the genetic length of the tracts in Morgans.

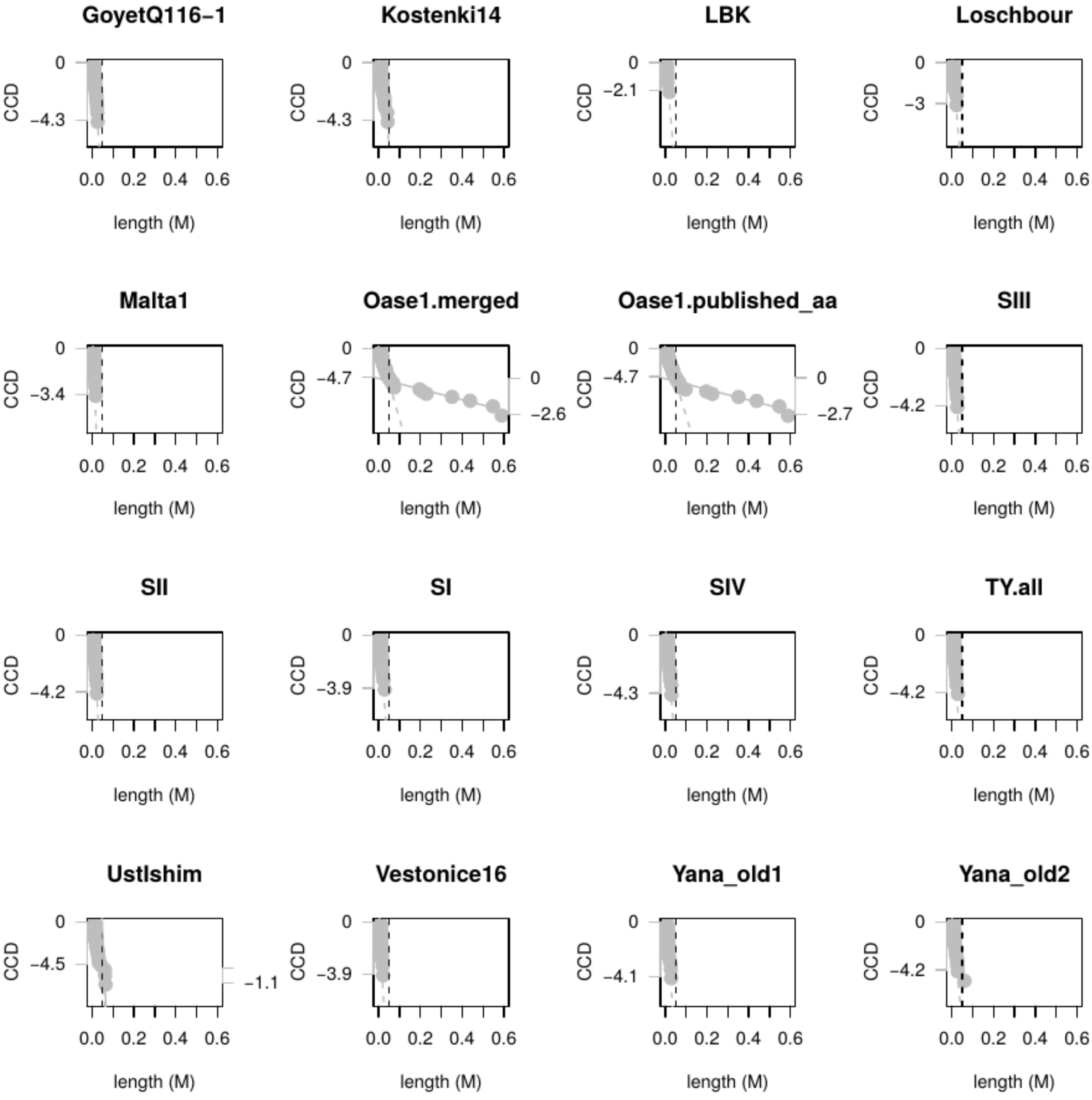


**Figure S8.4 Graphical representation of the relationship between CCD of tracts and tract lengths in other ancient modern humans.**

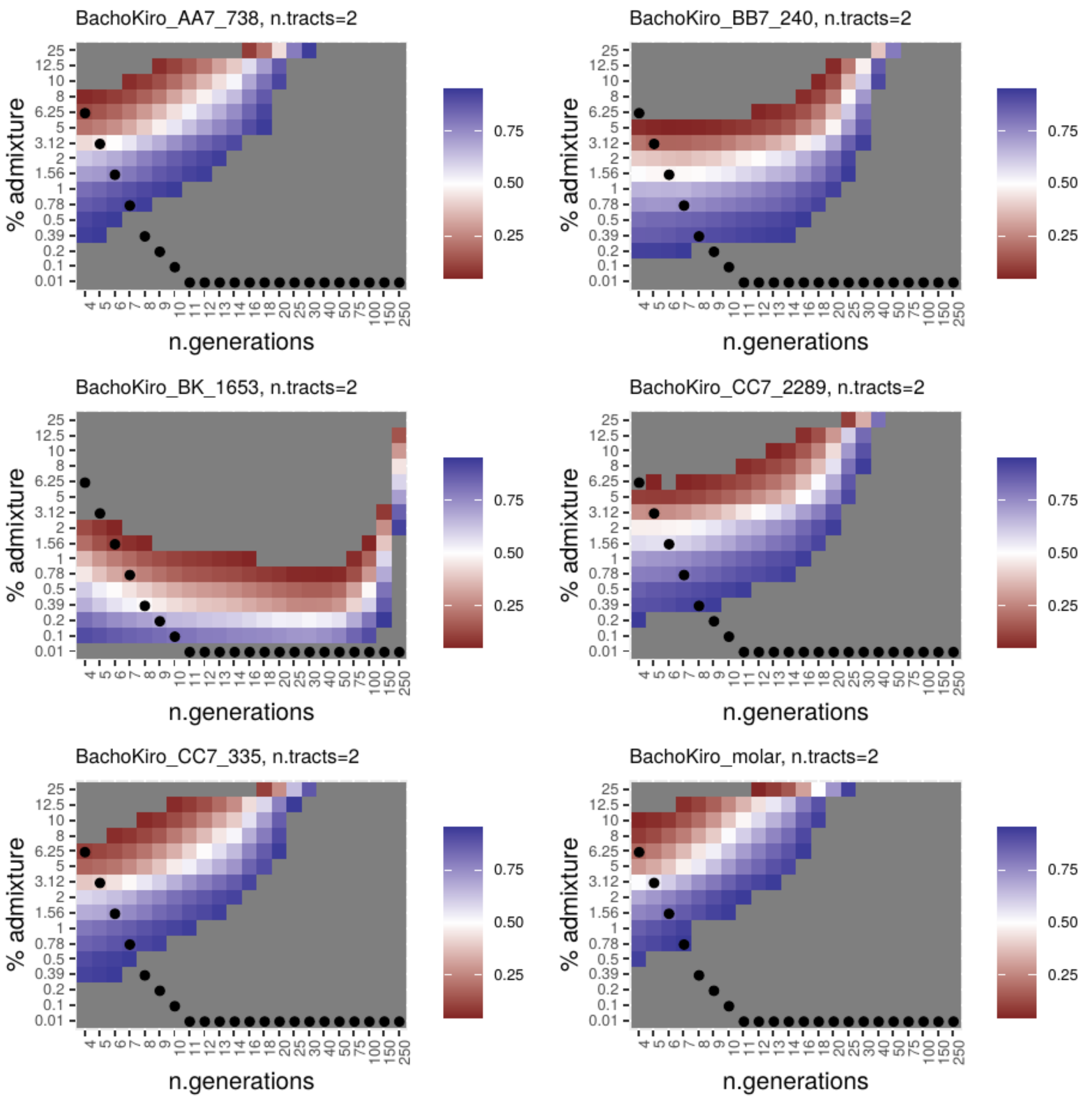


**Figure S8.5 Proportions of the simulations (q) where the two longest Neandertal tracts are shorter than for the observed tracts.** White indicates the best fitting combinations of parameters, i.e. combinations of parameters for which the observed data fall at the 50% percentile of the simulations. Combinations of parameters for which the observed data fall out of the 5 and 95 percentiles are shown in grey. Proportions of admixtures corresponding to the introgression of a single Neandertal ancestor are marked as black dots for each number of generations.

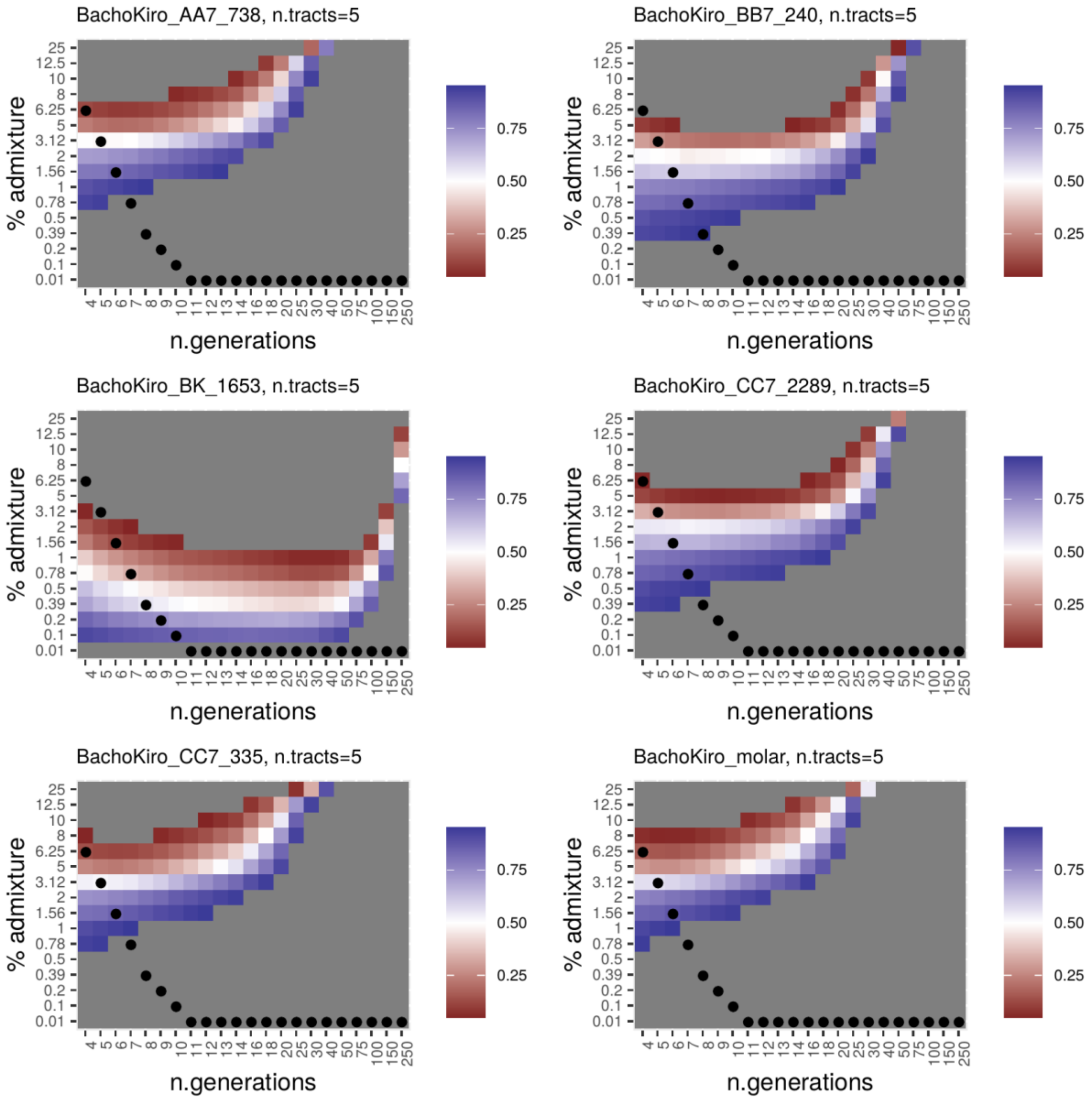


**Figure S8.6 Proportions of the simulations (q) where the five longest Neandertal tracts are shorter than for the observed tracts.** Figure description follows as Fig.S8.5.

# Supplementary information 9

## Sharing of Neandertal introgressed segments between Bacho Kiro and present-day individuals

We wanted to investigate if the Neandertal fragments we detect in Bacho Kiro individuals are shared with other ancient and present-day individuals. For ancient individuals (n=32) we consider segments longer than 0.2 cM and for modern day non-African individuals (n=229)[1] we consider segments longer than 0.05 cM[2] (see Supplementary Information 8). Furthermore, we group present-day individuals[1] by population (n=109) to increase the number of segments for comparisons. To evaluate the sharing we calculate how well the genomic positions of Neandertal segments correlate between Bacho Kiro Cave individuals vs ancient and present-day individuals, using the Pearson correlation coefficient, which we calculate in the following way:

p(X,Y) = (observed_overlap – expected_overlap)/(standard deviation of X * standard deviation of Y)

observed = overlapping sequence / callable genome length

expected_overlap = (sequence in X) / callable genome length * (sequence in Y) / callable genome length

standard deviation of X (or Y) = sqrt( overlapping sequence / callable genome length - (overlapping sequence / callable genome length)^2 )

If there is a perfect overlap, the correlation coefficient will be equal to 1 and if there is no correlation the coefficient will be equal to 0. This allows us to ask the question: "do Neandertal segments in Bacho Kiro individuals overlap more with other present-day and ancient individuals then expected by chance?". If they do, this suggests that the fragments are shared because of shared ancestry. In addition, we can evaluate the strength of the correlation to different populations. Introgressed Neandertal segments will be subject to drift the same way that allele frequencies are. Thus, populations that share more Neandertal segments share more drift.

In order to investigate if the fragment sets between Bacho Kiro individuals and other genomes overlap more than by chance we performed 500 bootstrap iterations where we randomly place the same number of archaic fragments of the same saize as in the analyzed genomes in the genomes of the Bacho Kiro individuals. If fragments overlap more than this, we conclude that the two genomes share ancestry. Furthermore, the magnitude of the correlation

coefficient will show if archaic segments in Bacho Kiro individuals are shared more with some present-day human populations than others.

Note that the amount of Neandertal segments in the genome is positively correlated with local recombination rate (Spearmans rank correlation coefficient = 0.15, pval < 4.46e-54)[3,4]. However, because recombination maps between different populations differ[5] and have changed over 35-45,000 years, we used homogeneous recombination rates for the tests.

Table S8.1 shows the mean correlation coefficient for each Bacho Kiro individual vs each "Super population", *i.e.* America, CentralAsia/Siberia, EastAsia, Oceania, SouthAsia, WestEurasia. The segments in the later Bacho Kiro Cave individual *BK1653* correlate more with Neandertal segments in West Eurasia than the Neandertal segments in IUP Bacho Kiro Cave individuals do. In contrast, the Neandertal segments in the IUP Bacho Kiro Cave individuals correlate more with Neandertal segments in present-day East Asian individuals (Fig. S9.1). The correlation coefficients when comparing Bacho Kiro individuals (segments shorter than 5 cM) with each individual SGDP population and 32 ancient individuals are shown in Fig. S9.2.

Segments longer than 5 cM found in IUP Bacho Kiro Cave individuals show very low correlation with present-day individuals (see Fig. S9.3). In fact, in all Bacho Kiro Cave individuals, except *CC7-2289* where we have little data, the genomic position of segments shorter than 5cM correlates significantly more with segments in present-day populations than segments which are longer than 5 cM (see Fig. S9.4).

**References SI9:**


1 Mallick, S. *et al.* The Simons Genome Diversity Project: 300 genomes from 142 diverse populations. *Nature* **538**, 201-206, doi:10.1038/nature18964 (2016).

2 Peter, B. M. 100,000 years of gene flow between Neandertals and Denisovans in the Altai mountains. *bioRxiv* (2020).

3 Skov, L. *et al.* The nature of Neanderthal introgression revealed by 27,566 Icelandic genomes. Nature 582, 78-83, doi: 0.1038/s41586-020-2225-9 (2020).

4 Schumer, M. *et al.* Natural selection interacts with recombination to shape the evolution of hybrid genomes. *Science* **360**, 656-660, doi: 10.1126/science.aar3684 (2018).

5 Kong, A. *et al.* Fine-scale recombination rate differences between sexes, populations and individuals. *Nature* **467**, 1099-1103, doi: 10.1038/nature09525 (2010).

**Table S9.1 Correlation coefficient of overlapping segments between Bacho Kiro Cave individuals and present-day super populations groups from SGDP[1].** The mean correlation coefficient for each super population are reported along with the 95% confidence intervals in brackets. Here we use tracts ≤ 5cM, thus representative of older introgression events. Bacho Kiro *CC7-2289* is highlighted in grey due to low amounts of data.

| Individual | America | CentralAsiaSiberia | EastAsia | Oceania | SouthAsia | WestEurasia |
|---|---|---|---|---|---|---|
| BachoKiro_AA7_738 | 0.08 (0.07-0.09) | 0.08 (0.07-0.09) | 0.09 (0.08-0.1) | 0.08 (0.05-0.11) | 0.06 (0.05-0.07) | 0.04 (0.04-0.04) |
| BachoKiro_BB7_240 | 0.09 (0.08-0.1) | 0.08 (0.07-0.09) | 0.1 (0.09-0.11) | 0.08 (0.06-0.1) | 0.07 (0.06-0.08) | 0.04 (0.04-0.04) |
| BachoKiro_BK_1653 | 0.08 (0.08-0.08) | 0.08 (0.07-0.09) | 0.07 (0.06-0.08) | 0.06 (0.04-0.08) | 0.08 (0.07-0.09) | 0.11 (0.1-0.12) |
| BachoKiro_CC7_2289 | 0 (-0.01-0.01) | 0.01 (0.01-0.01) | 0.03 (0.02-0.04) | 0.02 (0.01-0.03) | 0.03 (0.02-0.04) | 0.02 (0.02-0.02) |
| BachoKiro_CC7_335 | 0.09 (0.08-0.1) | 0.09 (0.08-0.1) | 0.12 (0.11-0.13) | 0.1 (0.08-0.12) | 0.08 (0.07-0.09) | 0.04 (0.04-0.04) |
| BachoKiro_F6_620 | 0.1 (0.09-0.11) | 0.09 (0.08-0.1) | 0.1 (0.09-0.11) | 0.09 (0.06-0.12) | 0.06 (0.05-0.07) | 0.05 (0.05-0.05) |

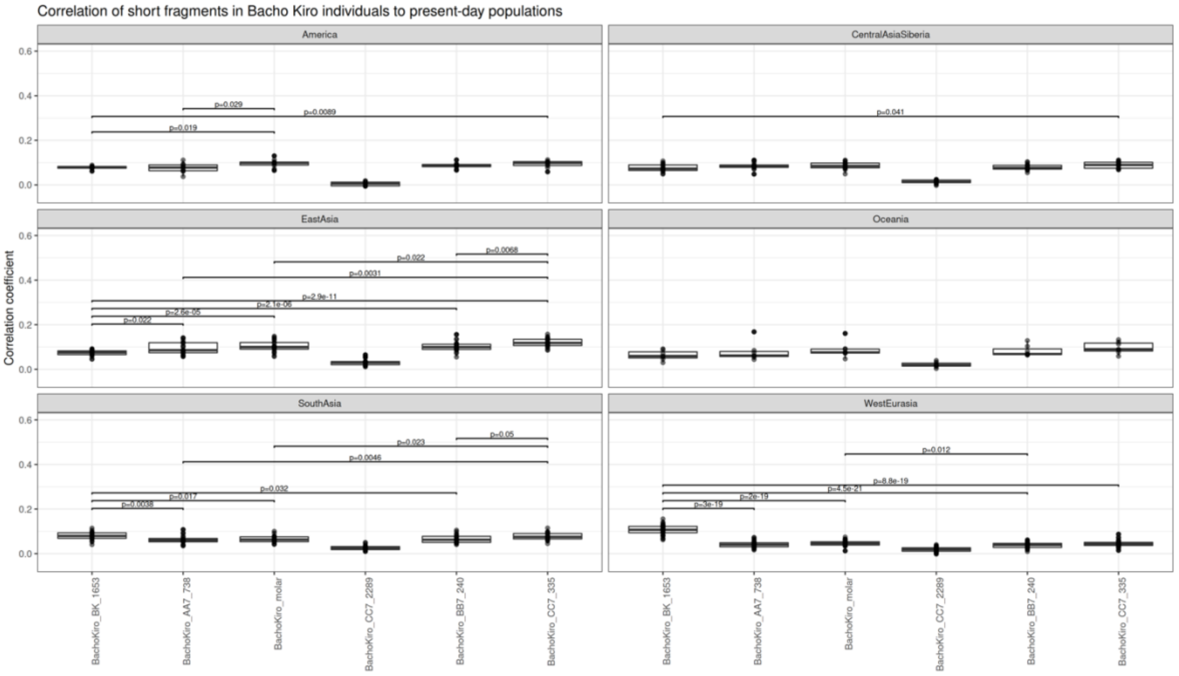


**Figure S9.1 Correlation coefficient of overlapping segments between Bacho Kiro Cave individuals and present-day super populations.** The mean correlation coefficient for each super population is reported for each Bacho Kiro Cave individual (black horizontal bar). Each point is a population from the super population (n=10 for America, n=15 for CentralAsiaSiberia, n=22 for EastAsia, n=7 for Oceania, n = 19 for SouthAsia, n = 36 for WestEurasia). The top and bottom part of the box indicates the 75% and 25% quantile respectively. We use tracts ≤ 5cM. The pvalues for each Wilcoxon signed-rank two-sided test are reported if significant (<0.05). We do not use the individual Bacho Kiro *CC7-2289* due to the low amounts of data.

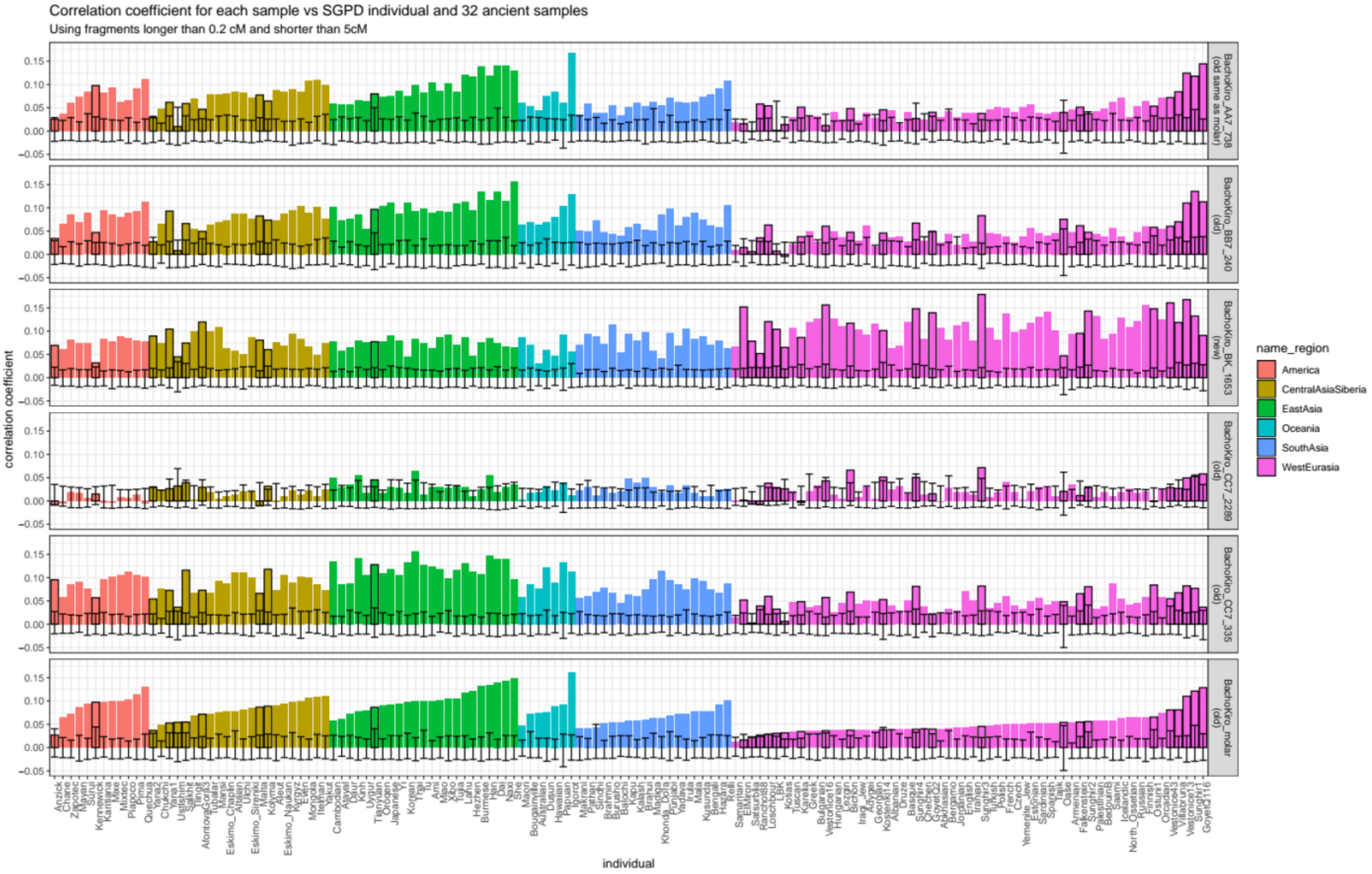


**Figure S9.2 Correlation coefficient of overlapping segments between Bacho Kiro Cave individuals and present-day population and ancient individuals.** The mean correlation coefficient between tracts ≤ 5cM in BachoKiro individuals, thus representative of older introgression events (n=138 for BachoKiro_BB7_240, n=66 for BachoKiro_AA7_738, n=153 for BachoKiro_molar, n=137 for BachoKiro_CC7_335, n=11 for BachoKiro_CC7_2289, n=257 for BachoKiro_BK_1653) and all tracts in present day populations and ancient individuals (mean n=1038, min n = 385, max n = 7535) is shown as a bar. The color of the bar indicates the super population. The black error bars show the 95% confidence intervals when we randomly place fragments in the genome (500 iterations) with the middle of the error bar being the mean correlation coefficient. Ancient individuals are highlighted with a black outline.

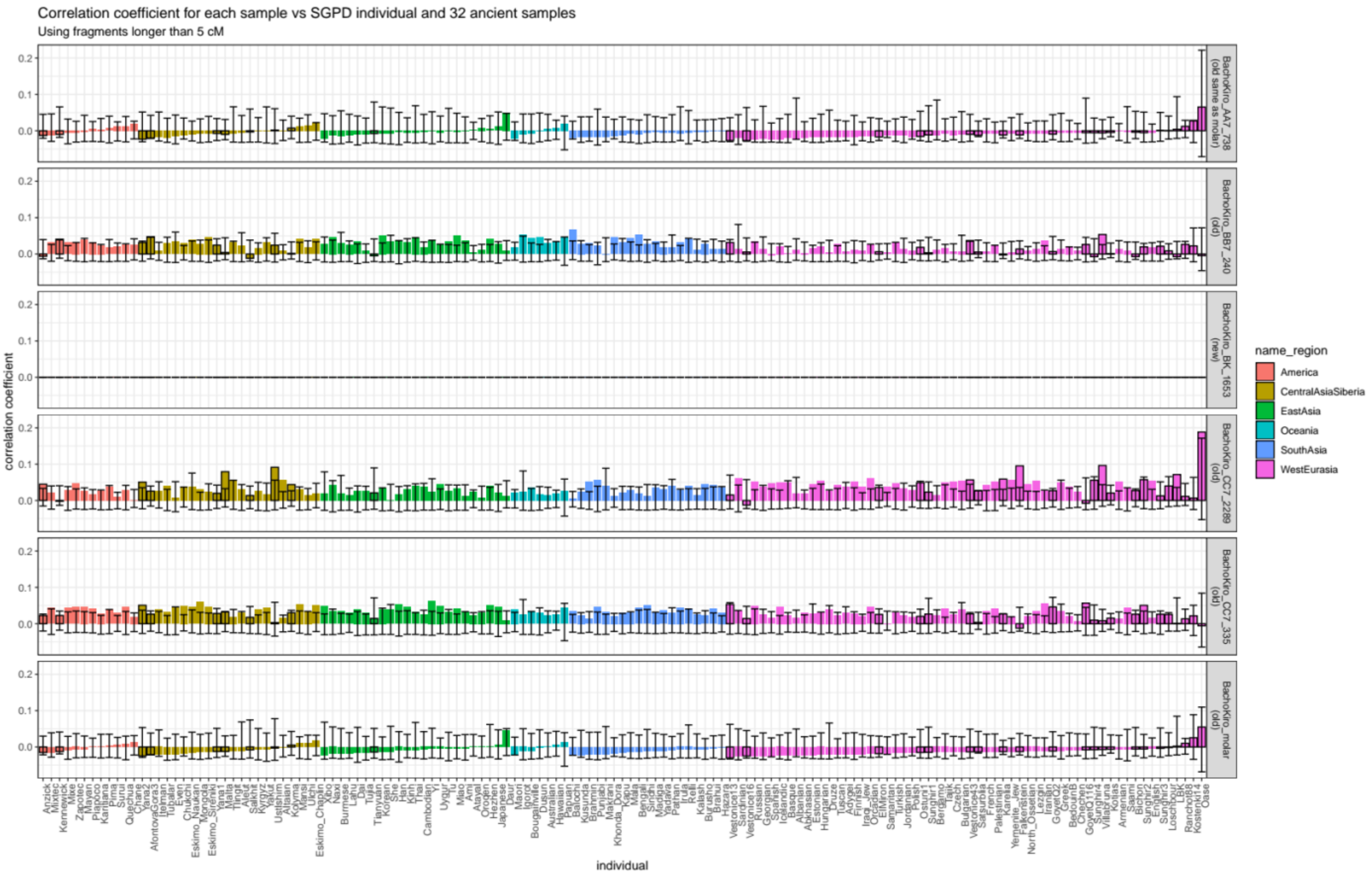


**Figure S9.3 Correlation coefficient of overlapping segments between Bacho Kiro Cave individuals and present-day population and ancient individuals.** The mean correlation coefficient between tracts $\geq$ 5cM in BachoKiro individuals, thus representative of more recent introgression events (n=8 for BachoKiro_BB7_240, n=7 for BachoKiro_AA7_738, n=9 for BachoKiro_molar, n=10 for BachoKiro_CC7_335, n=3 for BachoKiro_CC7_2289, n=0 for BachoKiro_BK_1653) and all tracts in present day populations and ancient individuals (mean n=1038, min n = 385, max n = 7535) is shown as a bar. The color of the bar indicates the super population. The black error bars show the 95% confidence intervals when we randomly place fragments in the genome (500 iterations) with the middle of the error bar being the mean correlation coefficient. Ancient individuals are highlighted with a black outline.

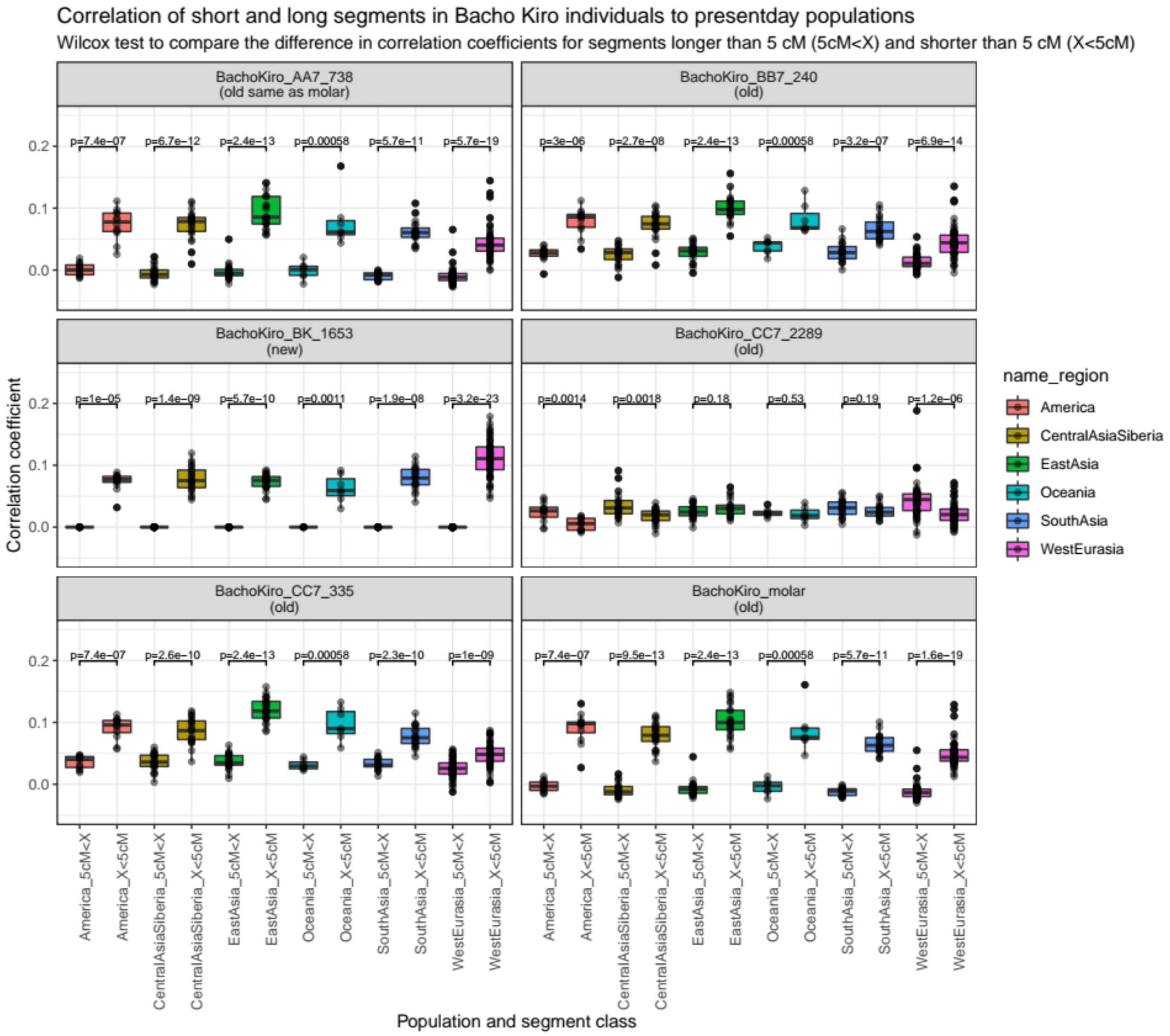


**Figure S9.4 Correlation coefficient of overlapping segments between Bacho Kiro Cave individuals and present-day population for segments longer and shorter than 5cM.** For each super population we compare the correlation coefficient of segments from Bacho Kiro individuals longer than 5cM vs the correlation coefficient of segments from Bacho Kiro individuals shorter than 5cM (n=10 for America, n=15 for CentralAsiaSiberia, n=22 for EastAsia, n=7 for Oceania, n = 19 for SouthAsia, n = 36 for WestEurasia). The top and bottom part of the box plot indicates the 75% and 25% quantile respectively. The pvalues for each Wilcoxon signed-rank two-sided test are reported if significant (<0.05).

# Supplementary Information 10

## Neandertal deserts

Previous studies of Neandertal introgression concluded that Neandertal introgressed fragments are heterogeneously distributed throughout the genome in modern day non-African populations[1,2]. Strikingly, this heterogeneity includes large regions that are nearly devoid of any Neandertal introgressed DNA. These so-called "Neandertal deserts" have been speculated to be regions where Neandertal DNA was removed by selection after introgression occurred, and thus may represent regions with important functional differences or even incompatibilities between Neandertals and modern humans. A subsequent analysis of 35 Melanesian individuals found that several of these deserts are also significantly depleted of Denisovan introgression, suggesting archaic DNA has been removed from the human genome in at least two events[3,4].

However, these analyses were performed on contemporary populations, making it difficult to resolve the timing of the formation of these Neandertal deserts; an important characteristic for considering models of selection. Genome-wide data from early modern humans may help to resolve this timing - e.g., we can ask the question "were these deserts present within 10,000 years following introgression?" In addition, the existence of very recent introgression events into such early modern humans may allow us to investigate the dynamics of desert formation on very short time scales, as little as a few generations. We therefore examined the overlap of Neandertal introgressed fragments in the three IUP Bacho Kiro Cave individuals, plus *Oase1*, with six previously identified Neandertal deserts[3]. Strikingly, these individuals carry almost no Neandertal DNA in the deserts (249 Kb out of 898 Mb of introgressed sequence). To determine if this lack of overlap could have occurred by chance, we calculated an empirical p-value by shifting the location of the deserts along the genome, eventually covering the entire genome. In 1 out of 251 such permutations, the overlap of Neandertal fragments and deserts was greater than the observed overlap, leading to an adjusted empirical p-value of 0.0079. Assuming an introgression date of 55,000 years ago[5,6] we can determine that the Neandertal deserts had already been established by approximately 10,000 years after introgression.

To examine desert formation in the more recent introgression event, we performed the same analysis on all introgressed fragments larger than 5cM. We use this threshold as described in Supplementary Information 8, since fragments longer than 5cM are unlikely to originate in the older introgression event. To maximize power, we also included fragments from the *Oase1*

individual, which experienced a similar recent introgression event. Strikingly, none of these large fragments overlap with previously identified deserts. However, when performing our permutation test, a substantial proportion of permutations (36 out of 251) also yielded no overlap, giving an adjusted empirical p-value of 0.147. Therefore, although we have no evidence of Neandertal DNA in the deserts as recently as 6-10 generations post-admixture, we also cannot reject that this occurs by chance. It is likely that the discovery of even a few additional individuals with recent introgression will allow the resolution of this question.

**References SI10:**


1 Vernot, B. & Akey, J. M. Resurrecting surviving Neandertal lineages from modern human genomes. *Science* **343**, 1017-1021, doi:10.1126/science.1245938 (2014).

2 Sankararaman, S. *et al.* The genomic landscape of Neanderthal ancestry in present-day humans. *Nature* **507**, 354-357, doi: 10.1038/nature12961 (2014).

3 Vernot, B. *et al.* Excavating Neandertal and Denisovan DNA from the genomes of Melanesian individuals. *Science* **352**, 235-239, doi:10.1126/science.aad9416 (2016).

4 Sankararaman, S., Mallick, S., Patterson, N. & Reich, D. The Combined Landscape of Denisovan and Neanderthal Ancestry in Present-Day Humans. *Curr. Biol.* **26**, 1241-1247, doi:10.1016/j.cub.2016.03.037 (2016).

5 Sankararaman, S., Patterson, N., Li, H., Paabo, S. & Reich, D. The date of interbreeding between Neandertals and modern humans. *PLoS Genet* **8**, e1002947, doi:10.1371/journal.pgen.1002947 (2012).

6 Fu, Q. *et al.* Genome sequence of a 45,000-year-old modern human from western Siberia. *Nature* **514**, 445-449, doi:10.1038/nature13810 (2014).
