# Supplementary material for: Initial Upper Palaeolithic humans in Europe had recent Neanderthal ancestry
Source: Nature. 2021 Apr 7;592(7853):253–7. doi: 10.1038/s41586-021-03335-3 (PMC8026394; doi:10.1038/s41586-021-03335-3)
Supplement: Supplementary file 2 — Reporting Summary [file 41586_2021_3335_MOESM2_ESM.pdf]

nature research

Corresponding author(s): Mateja Hajdinjak, Svante Pääbo

Last updated by author(s): Dec 18, 2020

Reporting Summary

Nature Research wishes to improve the reproducibility of the work that we publish. This form provides structure for consistency and transparency in reporting. For further information on Nature Research policies, see our Editorial Policies and the Editorial Policy Checklist.

Statistics

For all statistical analyses, confirm that the following items are present in the figure legend, table legend, main text, or Methods section.

n/a	Confirmed	
☐	☒	The exact sample size (*n*) for each experimental group/condition, given as a discrete number and unit of measurement
☐	☒	A statement on whether measurements were taken from distinct samples or whether the same sample was measured repeatedly
☐	☒	The statistical test(s) used AND whether they are one- or two-sided<br>*Only common tests should be described solely by name; describe more complex techniques in the Methods section.*
☒	☐	A description of all covariates tested
☒	☐	A description of any assumptions or corrections, such as tests of normality and adjustment for multiple comparisons
☐	☒	A full description of the statistical parameters including central tendency (e.g. means) or other basic estimates (e.g. regression coefficient) AND variation (e.g. standard deviation) or associated estimates of uncertainty (e.g. confidence intervals)
☐	☒	For null hypothesis testing, the test statistic (e.g. *F*, *t*, *r*) with confidence intervals, effect sizes, degrees of freedom and *P* value noted<br>*Give P values as exact values whenever suitable.*
☒	☐	For Bayesian analysis, information on the choice of priors and Markov chain Monte Carlo settings
☒	☐	For hierarchical and complex designs, identification of the appropriate level for tests and full reporting of outcomes
☒	☐	Estimates of effect sizes (e.g. Cohen's *d*, Pearson's *r*), indicating how they were calculated

Our web collection on statistics for biologists contains articles on many of the points above.

Software and code

Policy information about availability of computer code

Data collection	No software was used for data collection.
Data analysis	All software packages used for analysis are cited in the Online Methods section and in the Supplementary Information and all used packages are publicly available:<br>base caller: Bustard (Illumina);<br>adapter trimming: leeHom (version used for this manuscript available at https://bioinf.eva.mpg.de/leehom/);<br>mapping: Burrows-Wheeler Aligner (BWA, version 0.5.10-evan.9-1-g44db244);<br>PCR duplicate removal: bam-rmdup (version 0.6.3, https://github.com/mpieva/biohazard-tools);<br>handling BAM files: samtools (version 1.3.1), bedtools (version 2.24.0), bam-caller (https://github.com/bodkan/bam-caller, version 0.1);<br>mtDNA contamination estimates: schmutzi (version 1.5.5);<br>nuclear contamination estimates: ANGSD (version 0.929-27-ge7739a5), AuthentiCT (https://github.com/StephanePeyregne/AuthentiCT, version 1.0.0);<br>f-statistics and qpGraph: ADMIXTOOLS (version 5.1) and R package admixr (version 0.7.1);<br>genotype calls for Y-chromosome of F6-620: snpAD (version 0.3.4);<br>haplogroup calling: yHaplo (version 1.0.18);<br>PCA: smartpca from EIGENSOFT package;<br>detecting archaic introgressed segments: admixfrog (version 0.5.6, https://github.com/BenjaminPeter/admixfrog/).

For manuscripts utilizing custom algorithms or software that are central to the research but not yet described in published literature, software must be made available to editors and reviewers. We strongly encourage code deposition in a community repository (e.g. GitHub). See the Nature Research guidelines for submitting code & software for further information.

Data

Policy information about availability of data

All manuscripts must include a data availability statement. This statement should provide the following information, where applicable:

- Accession codes, unique identifiers, or web links for publicly available datasets
- A list of figures that have associated raw data
- A description of any restrictions on data availability

Newly produced sequence data of Bacho Kiro Cave specimens and Oase 1 are deposited in the European Nucleotide Archive (ENA, https://www.ebi.ac.uk/ena/browser/home) under the accession number PRJEB39134.

Comparative data of present-day human genomes from Simons Genome Diversity Project that were used in this study is available at: https://www.simonsfoundation.org/simons-genome-diversity-project/

Comparative data used in this study that includes genotypes of 2,109 ancient and 2,974 present-day individuals compiled from published studies is available in the EIGENSTRAT file format at: https://reich.hms.harvard.edu/downloadable-genotypes-present-day-and-ancient-dna-data-compiled-published-papers/ (version 37.2, released February 22, 2019).

To determine the Y chromosome haplogroups of male individuals in this study, we used the Y-haplogroup tree from the International Society of Genetic Genealogy (ISOGG, http://www.isogg.org, version: 13.38).

Field-specific reporting

Please select the one below that is the best fit for your research. If you are not sure, read the appropriate sections before making your selection.

☒ Life sciences ☐ Behavioural & social sciences ☐ Ecological, evolutionary & environmental sciences

For a reference copy of the document with all sections, see nature.com/documents/nr-reporting-summary-flat.pdf

Life sciences study design

All studies must disclose on these points even when the disclosure is negative.

Sample size	The number of genomes analysed in this study was determined by identifying those specimens that had sufficient levels of ancient DNA preservation for downstream sequencing and analysis. Human specimens from this time period in Europe are extremely scarce, and in this study we screened seven specimens excavated recently from the Bacho Kiro Cave in Bulgaria:<br>- a human lower molar (F6-620) found in the upper part of the Layer J in the Main Sector of Bacho Kiro Cave<br>- four bone fragments (AA7-738, BB7-240, CC7-2289 and CC7-335) from Layer I in the Niche 1 sector<br>- a bone fragment from the Layer B in the Main Sector (F6-597)<br>- a bone fragment that was identified among the finds from excavations in the 1970s when it was retrieved in a position corresponding to the interface of Layers B/C (BK1653).<br>The bone fragments were initially identified as hominin based on the Zooarchaeology by Mass Spectrometry (ZooMS), and we screened them for ancient DNA preservation. Therefore, the sample size was predetermined based on the availability of the identified hominin specimens.<br>We also used the remaining bone powder of Oase 1 specimen from Romania from the 2015 study (Fu et al, Nature) and treated it with 0.5% hypochlorite solution in order to remove some of the present-day human and microbial contamination.<br>Given very low proportion of endogenous DNA and high levels of present-day human DNA contamination, we excluded the specimen F6-597 from downstream in solution hybridization captures and analyses.
Data exclusions	We used pre-established criteria in ancient DNA research of excluding sequences from the sequencing data that did not map to the human genome, sequences that were shorter than 35 base pairs and sequences mapping with a low mapping quality (< 25), all of which are excluded to avoid using sequences that are not endogenous to the individual sequenced.<br>Given the high contamination estimates and low nuclear DNA content, we excluded the libraries of the specimen F6-597 from nuclear captures and further downstream analyses.
Replication	The specimens sampled in this study were sampled on three different occasions. We produced in total 35 single-stranded DNA libraries from seven extracts of Bacho Kiro Cave specimens and 6 single-stranded libraries from three Oase 1 extracts, along with respective extraction and library negative controls, and distributed over six different experiments. The results of reproducibility of the data generation and analyses are reported across the tables in the Supplementary Information Section 2.<br>To allow the reproducibility of the downstream analyses, all filtering steps and the comparative data used are detailed in the Online Methods and the Supplementary Information of this study; also all of the sequence data obtained from Bacho Kiro Cave specimens and Oase 1 needed for replication of obtained results and conclusions of this study are deposited in the European Nucleotide Archive (ENA) under the accession number PRJEB39134.
Randomization	Randomization is not relevant to this study. We first determined DNA preservation in all ancient specimens selected for this study and then proceeded to analyse generated genome-wide data of all specimens that showed evidence of endogenous DNA preservation.
Blinding	Blinding was not relevant as we sampled ancient hominin specimens that were selected for this study based on their age and provenance, thus blinding would be inappropriate given the scarcity and value of the sampled material. Blinding in downstream data analyses was not relevant given that we analysed genome-wide data of eight specimens in relation to the publicly available datasets of present-day and ancient

human genomes.

Reporting for specific materials, systems and methods

We require information from authors about some types of materials, experimental systems and methods used in many studies. Here, indicate whether each material, system or method listed is relevant to your study. If you are not sure if a list item applies to your research, read the appropriate section before selecting a response.

Materials & experimental systems

n/a	Involved in the study
☒	☐ Antibodies
☒	☐ Eukaryotic cell lines
☐	☒ Palaeontology and archaeology
☒	☐ Animals and other organisms
☒	☐ Human research participants
☒	☐ Clinical data
☒	☐ Dual use research of concern

Methods

n/a	Involved in the study
☒	☐ ChIP-seq
☒	☐ Flow cytometry
☒	☐ MRI-based neuroimaging

Palaeontology and Archaeology

Specimen provenance

Specimen Oase 1 is deposited at the "Emil Racovita" Institute of Speleology in Bucharest, Romania. Permission was granted to Svante Pääbo of the MPI-EVA in September 2009 for the sampling of the Oase 1 for genetic analyses with the specimen being sampled on the September 28, 2009 in Bucharest, Romania.
Excavation of the Bacho Kiro Cave was authorized by the Bulgarian Ministry of Culture, and delivered by NAIM-BAS: Nr124/11.05 2015; Nr225/28.04.2016; Nr47/02.05.2017; Nr99/17.04.2018/ Nr120/2019. Bacho Kiro Cave specimens were sampled in the clean room facility of the MPI-EVA in Leipzig, Germany in January and March 2018.

Specimen deposition

The Oase 1 specimen is deposited at "Emil Racovita" Institute of Speleology, Department of Geospeleology and Palaeontology, str. Frumoasa 31, Bucharest, Romania.
The palaeontological material from the Bacho Kiro Cave is deposited at the National Museum of Natural History in Sofia, Bulgaria.

Dating methods

There are no new radiocarbon dates provided in this study. However, all the previously published radiocarbon dates were newly re-calibrated using the new calibration curve IntCal20 and are provided in the Supplementary Information Section 1, Table S1.1.

☒ Tick this box to confirm that the raw and calibrated dates are available in the paper or in Supplementary Information.

Ethics oversight

All approvals for specimen handling have been obtained from the relevant institutions:
- for the Oase1 specimen, the permission was granted to Svante Pääbo of the MPI-EVA by the Emil Racovita Institute of Speleology, as national authority in caves study.
- for the Bacho Kiro Cave specimens the permission was granted by the Bulgarian Ministry of Culture and the National Museum of Natural History (Sofia, Bulgaria)

Note that full information on the approval of the study protocol must also be provided in the manuscript.
